# Performance of transient elastography and serum fibrosis biomarkers for non-invasive evaluation of recurrent fibrosis after liver transplantation: A meta-analysis

**DOI:** 10.1371/journal.pone.0185192

**Published:** 2017-09-27

**Authors:** Mamatha Bhat, Mahmood Tazari, Giada Sebastiani

**Affiliations:** 1 Multi Organ Transplant Program, University Network, Toronto, Canada; 2 Division of Gastroenterology and Hepatology, University of Toronto, Toronto, Canada; 3 Division of Gastroenterology and Hepatology, McGill University Health Centre, Montreal, Canada; Medizinische Fakultat der RWTH Aachen, GERMANY

## Abstract

Recurrent fibrosis after liver transplantation (LT) impacts on long-term graft and patient survival. We performed a meta-analysis to compare the accuracy of non-invasive methods to diagnose significant recurrent fibrosis (stage F2-F4) following LT. Studies comparing serum fibrosis biomarkers, namely AST-to-platelet ratio index (APRI), fibrosis score 4 (FIB-4), or transient elastography (TE) with liver biopsy in LT recipients were systematically identified through electronic databases. In the meta-analysis, we calculated the weighted pooled odds ratio and used a fixed effect model, as there was no significant heterogeneity between studies. Eight studies were included for APRI, four for FIB-4, and twelve for TE. The mean prevalence of significant liver fibrosis was 37.4%. The summary odds ratio was significantly higher for TE (21.17, 95% CI confidence interval 14.10–31.77, p = 1X10^-30^) as compared to APRI (9.02, 95% CI 5.79–14.07; p = 1X10^-30^) and FIB-4 (7.08, 95% CI 4.00–12.55; p = 1.93X10^-11^). In conclusion, TE performs best to diagnose recurrent fibrosis in LT recipients. APRI and FIB-4 can be used as an estimate of significant fibrosis at centres where TE is not available. Longitudinal assessment of fibrosis by means of these non-invasive tests may reduce the need for liver biopsy.

## Introduction

The development of hepatic fibrosis adversely affects the prognosis of any chronic liver disease, regardless of etiology. In the specific setting of liver transplantation (LT), recurrent liver fibrosis portends worse graft survival and need for re-transplantation, thus impacting on overall survival [1–3]. LT recipients may have multiple cofactors leading to liver fibrosis recurrence. Patients who undergo LT for hepatitis C virus (HCV) infection have universal recurrence, with development of cirrhosis in up to 30% by 5 years[4–6]. The landscape of antiviral therapy for hepatitis C has been dynamic, and has led to highly effective active, interferon-free regimens[7]. However, concerns about costs and availability in the post-transplant setting may limit the widespread use of interferon-free regimens, which highlights the need for prioritization of patients for therapy. The current AASLD/IDSA guidelines recommend that all patients with HCV be treated following LT[7]. Recurrent or *de novo* nonalcoholic fatty liver disease (NAFLD) also affects more than 20% of patients, given the high rate of metabolic syndrome triggered by rapid weight gain and immunosuppressive medications following LT[8, 9]. Various other factors, such as recipient age, donor age, type of immunosuppression, and cytomegalovirus (CMV) viremia increase the risk of recurrent fibrosis after LT[10].

Early identification of fibrosis recurrence in LT recipients is of paramount importance to permit risk stratification, ascertain prognosis and thereby provide targeted interventions and management readjustment. Liver biopsy has long been the gold standard to stage liver fibrosis. Annual protocol liver biopsies after LT were traditionally performed for hepatitis C[11], and there are certain conditions such as autoimmune hepatitis where they are considered. However, liver biopsy is impractical as a screening tool and for serial monitoring because of its invasiveness, cost and potential for sampling error[12, 13]. These are important considerations in the LT patient population, where longitudinal follow-up of recurrent fibrosis is required.

Serum biomarkers based on readily available parameters have been proposed to stage liver fibrosis in the pre-LT population, including the fibrosis score 4 (FIB-4) and aspartate aminotransferase (AST)-to-Platelet ratio index (APRI). Whereas the APRI is calculated on the basis of AST and platelet values, the FIB-4 score additionally includes age and alanine aminotransferase. An additional non-invasive tool is transient elastography (TE) (Fibroscan®), whose measurement of liver stiffness is proportional to the severity of fibrosis[3, 14, 15]. Although non-invasive methods do not enable distinction between single fibrosis stages(18), they have demonstrated good accuracy in detecting significant liver fibrosis, allowing for repeat assessment over time. Moreover, they also portend prognosis, by identifying patients at risk for liver-related complications such as death and need for LT[1, 2, 16]. As such, the routine incorporation of these non-invasive tests into the post-LT clinical setting could potentially facilitate screening and serial monitoring, thus identifying those patients in need of more immediate intervention.

APRI, FIB-4 and TE have been extensively validated in the pre-transplant clinical setting, where they have been recommended for clinical use by guidelines[17]. To our knowledge, there has been no meta-analysis of non-invasive diagnostic tests for liver fibrosis due to all etiologies of liver disease in the post-LT setting.

The objective of this study was to perform a meta-analysis of the diagnostic accuracy of simple serum biomarkers, namely APRI and FIB-4, and TE for the prediction of recurrent liver fibrosis in the post-LT setting. Although other non-invasive fibrosis measures have been studied in LT recipients, we focused on these tests due to their more extensive validation and widespread use.

## Materials and methods

### Literature search

A systematic literature search from 2003 to May 2017 was performed which included the following sources:

- Electronic databases: Pubmed, MEDLINE, EMBASE and Cochrane library databases were systematically searched for original articles and abstracts.
- Relevant websites and conference abstract books: American Association for the Study of the Liver, International Liver Transplant Society, European Association for the Study of the Liver, American Transplant Congress, Digestive Diseases Week, Asian Pacific Association for the Study of the Liver were searched for conference proceedings and abstracts.
- Manual review of reference lists of relevant articles.

A combination of relevant text words and MeSH terms were applied, namely *liver transplantation AND* one of the following: *fibrosis*, *non-invasive fibrosis markers*, *serum fibrosis markers*, *APRI*, *FIB-4*, *TE*, *and liver stiffness measurement*. For management of the searched literature, Endnote version X7 (Thomson Reuters, New York, NY) was used. We chose these three non-invasive tests as they are widely used and available, as well as the literature search revealed more studies than other scores. For example, there has been limited evaluation of the Fibrotest in the detection of significant fibrosis after LT, with only 2 prospective studies comprising 82 patients, both of which reported a low diagnostic accuracy[18, 19]. Another patented non-invasive test, the Enhanced Liver Fibrosis (ELF) Score has been evaluated in two prospective studies comprising 113 patients with all etiologies of liver disease as indication for LT, with variable accuracy reported[19, 20].

### Inclusion criteria

Studies were included if they met the following criteria: they evaluated APRI, FIB-4 or TE; they used liver biopsy as the reference standard; they employed comparable histologic staging systems: METAVIR, Ishak, Brunt, Ludwig, Knodell; they evaluated the diagnostic accuracy of the test expressed as area under the curve (AUC) for significant liver fibrosis (stage F2-4); and/or they evaluated sensitivity, specificity, positive predictive value (PPV) or negative predictive value (NPV) for the diagnosis of significant liver fibrosis based on cut-off values for APRI, FIB-4 or TE. Both adult and pediatric studies were included.

### Exclusion criteria

Studies were excluded if they met the following criteria: they were not conducted in the post-LT setting; they did not use liver biopsy as the standard of reference; they did not report data on diagnostic accuracy (AUC), sensitivity, specificity, PPV or NPV for the diagnosis of significant liver fibrosis based on cut-off values for APRI, FIB-4 or TE; they were review articles, letters to the editor or editorials not reporting own original data; they were abstracts presenting data from the same study at different meetings.

#### Data extraction

One reviewer (M.B.) searched the various databases listed above, and identified pertinent studies (both articles and abstracts) for further assessment. Both reviewers (M.B. and G.S.) reviewed the abstracts and full text versions of the articles independently, evaluating study eligibility, grading study quality, and extracting data. We took note of study design, number of participants, non-invasive tool adopted among APRI, FIB-4 and TE. From the selected studies, we extracted the following data: study characteristics such as authors, year of publication, hospital or medical school and study design; demographic and clinical characteristics of participants; characteristics of liver biopsy; non-invasive method adopted among APRI, FIB-4 and TE; correlation with liver biopsy staging of fibrosis as data categories and relative cut-off value of the non-invasive test. In case of disagreement regarding the selected studies or data appropriate for extraction, studies were refereed by a third reviewer (M.T.) and resolved by consensus. Methodological quality was assessed using the Quality Assessment of Diagnostic Accuracy Studies (QUADAS-2) Score[21]. The primary outcome was the identification of significant fibrosis, defined as stage F2–4 according to the METAVIR staging system. This threshold was chosen because it indicates a progressive liver disease eventually leading to cirrhosis and requiring interventions, such as antiviral therapy (hepatitis C) and life style modifications (NAFLD)[7, 22]. In all the included studies, APRI and FIB-4 were computed as per original formulas: APRI as [100 x (AST/upper limit of normality)/platelet count (10^9^ /L)[23]; FIB-4 as age (years) x AST /platelet count (10^9^ /L)] x ALT^1/2^[24].

### Data analysis

We estimated the diagnostic odds ratios and AUC with their corresponding 95% confidence intervals (95% CIs) for each study based on the diagnostic tests. In the meta-analysis we calculated the weighted pooled odds ratio. The heterogeneity statistics calculated the summary odds ratio under random effects model[25]. The summary receiving operating characteristics (ROC) curves (SROC) shows the overall diagnostic test including each study’s test and the sample size of each study.

Pooled measures for diagnostic performance, such as sensitivity, specificity, diagnostic odds ratios (DORs) with their corresponding 95% confidence intervals (95% CIs), and AUC were calculated. The DORs combine sensitivity and specificity into one measure for diagnostic performance. A DOR of 1 means that the test has no ability to discriminate between two outcomes. In the context of this study, the higher the DOR, the better the diagnostic accuracy of the non-invasive test for assessing significant liver fibrosis.

The forest plot was graphed for odds ratios, as a summary and for individual studies. A Chi-square-based test of homogeneity was performed, and the Cochran's *Q* inconsistency index (*I*2) statistics were determined. The heterogeneity between studies was calculated by *I*^*2*^ values. Publication bias was assessed by constructing funnel plots for DOR by Egger's test[26]. The absence of publication bias was indicated by the data points forming a symmetric funnel-shaped distribution and significance level p>0.05. We used the SAS statistical software 9.4 (SAS Institute, Cary, NC) to perform the statistical analysis at p<0.05 level of significant test and R program for SROC.

### Sensitivity analyses

Sensitivity analyses were employed to examine the impact of the following factors on test performance for identifying significant liver fibrosis: study quality; prevalence of significant fibrosis; and quality of the reference standard for assessing fibrosis. The reference standard was deemed adequate if a study excluded liver biopsies <10 mm. If sufficient data were not reported in the manuscript, the reference standard was considered inadequate.

## Results

**Fig 1:**
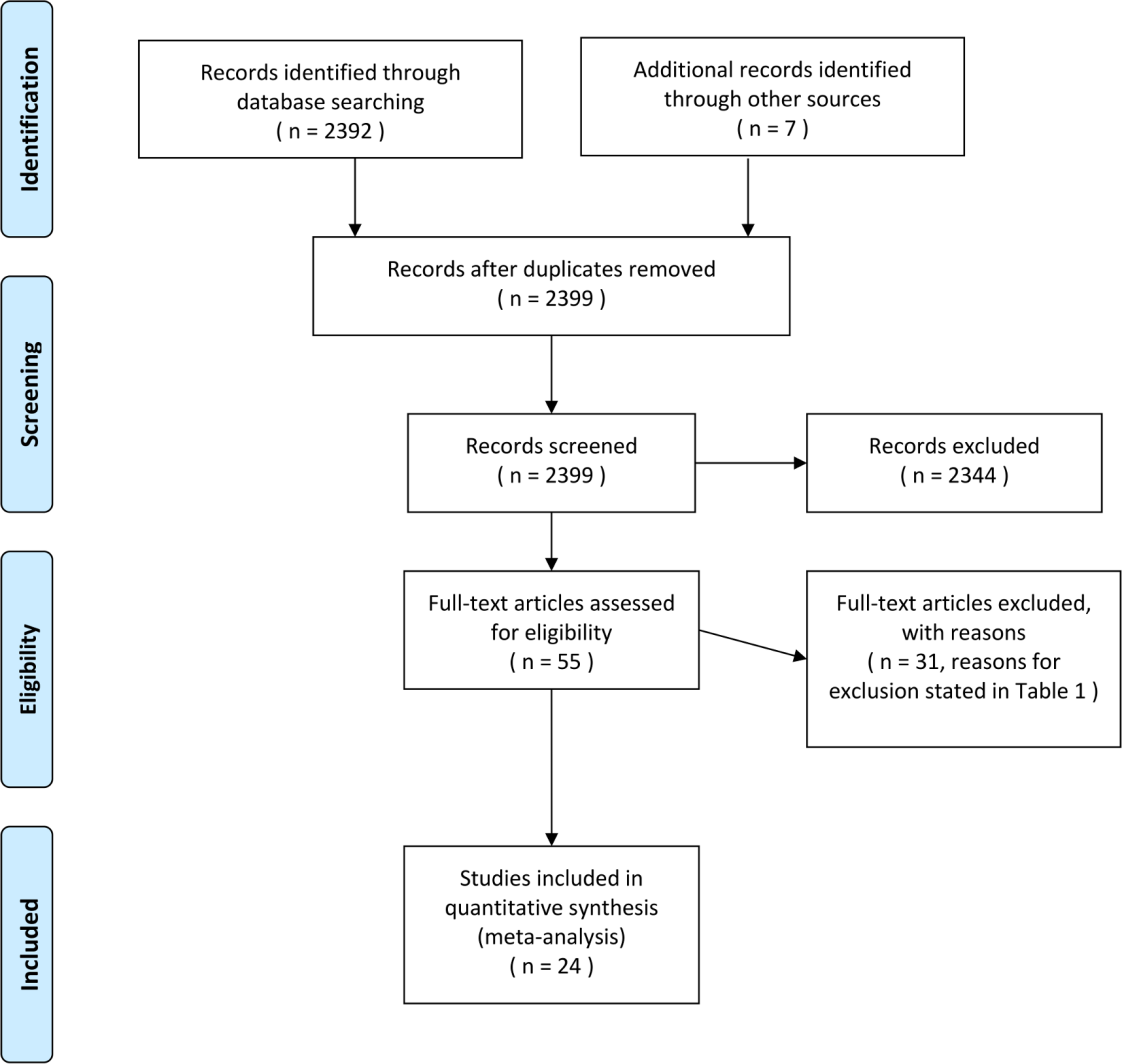
Flow diagram of the search strategy.

The literature search by the reviewers resulted in 2399 studies identified with our search terms (S1–S12 Files). Of these, 55 included original data. 48 were full-text manuscripts, and 7 studies were available in abstract form only. The systematic literature search and study selection are depicted in the flowchart of Fig 1. Ultimately, studies were excluded as they were not pertinent to non-invasive evaluation of fibrosis after LT, due to insufficient data and review articles/commentary. A total of 171 studies described TE, APRI or FIB-4, of which 33 included original data. 33 studies compared TE, APRI or FIB-4 versus liver biopsy in LT recipients. Finally, 24 studies were included in the meta-analysis. The studies excluded from the meta-analysis and reasons for exclusion are provided in Table 1. Our final data set for the meta-analysis included 8 studies assessing APRI, 4 assessing FIB-4 and 12 assessing TE (Table 2).

**Table 1:** Excluded studies and reasons for exclusion.

Non-invasive test	Author, Year	Reason for exclusion
TE	Della Guardia, 2013	Insufficient data
TE	Bellido-Munoz, 2012	Insufficient data
TE	Adebajo et al, 2012	Meta-analysis
TE	Vinciguerra et al, 2011	Insufficient data
TE	Kamphues et al., 2010	Insufficient data
TE	Masuzaki et al., 2009	Insufficient data
TE	Harada et al., 2008	Insufficient data
APRI, FIB-4	Kitajima et al, 2016	Insufficient data
APRI, FIB-4	Sheen et al, 2016	Insufficient data, and used F3-F4 as significant fibrosis
APRI	Carrion et al., 2010	Insufficient data
APRI	Corradi et al., 2009	Insufficient data

Legend: TE: transient elastography, APRI: AST-to-platelet ratio index, FIB-4: Fibrosis score 4.

**Table 2:** Characteristics of the studies included in our meta-analysis of non-invasive tests to evaluate significant fibrosis after liver transplantation.

Non-invasive test	Author/ Year	Study design	N	Time after LT (months)	Median/ mean age (male)	Etiology	Mean length biopsy, (interval biopsy & test)	Prevalence F2-4 (%)	QUADAS score
TE	Della-Guardia, 2017[36]	Prospective	267	31.7	56 (67.7%)	74.9% HCV, and others	At least 10 mm long or 10 portal tracts, mean not provided (same day)	46.1	9
TE	Crespo, 2016[37]	Retrospective	64	12	59 (73.6%)	HCV	NA (same day)	33	9
TE	Mikołajczyk-Korniak, 2016[38]	Prospective	36	29.2	54.6	HCV	20.9 mm (2.9 months)	80.5	13
TE	Lutz, 2016[39]	Prospective	48	23±2	54.4 (68.8%)	27.1% HCV, and others	NA (within 24 hours)	27.1	12
TE	Barrault, 2013	Prospective	43	55.8±4.9	58 yr (74.4%)	HCV, alcohol, other	25.1 mm (<2 months)	37.7	12
TE	Goldsmith, 2013	Prospective	26	36 (1–176)	5.6 yr (51.3%)	Biliary atresia, α1antitripsin, Wilson, other	NA (<6 months)	NA	12
TE	Crespo, 2012	Prospective	87	43	60 yr (69%)	HCV, NAFLD, alcohol, cholestasis, other	20 mm (<15 days)	42	14
TE	Sebagh, 2012	Prospective	91	240	37.3 yr (45%)	HBV, HCV, alcohol, other	28.2 ± 8.6 mm (same day)	40.4	13
TE	Beckebaum, 2010	Prospective	157	80.2±65.7	52.5 yr (61.1%)	HCV	At least 15 mm long (same day)	58.6	13
TE	Carrion, 2006	Prospective	124	24 (5–120)	60 yr (66%)	HCV	NA (<2 weeks)	43	13
APRI, TE	Corradi, 2009	Prospective	56	NA	58 yr (83.9%)	HCV	29 mm (<40 days)	32	13
TE	Rigamonti, 2008	Prospective	95	35 (6–156)	54 yr (81%)	HCV	32 mm (same day)	NA	14
APRI, FIB-4	Crespo, 2016[37]	Retrospective	72	12	59 (73.6%)	HCV	NA (same day)	33	9
APRI	D’Souza, 2016	Retrospective	39	124.68	12.52 (51.2%)	Extrahepatic biliary cirrhosis, other	NA (same day)	38.5	9
APRI	Pinto, 2014[40]	Prospective	30	60	11 (63%)	Biliary atresia, metabolic disease, α1antitripsin, Wilson, other	NA (<4 months)	20	9
APRI, FIB-4, TE	Kamphues, 2010[27]	Prospective	94	80.6	51.7 (64.9%)	HCV	1.5 cm (same day)	68.1	13
APRI, FIB-4	Pissaia, 2009[41]	Retrospective	50^1^	30.7	49.6 (62%)	All etiologies	NA (same day)	28	9
APRI	Harada, 2008[28]	Prospective	56	25.6	63.1 (53.6%)	HCV	15 mm (same day)	37.5	12
APRI	Toniutto, 2007[42]	Prospective	51^2^	24	56 (60.8%)	HCV	NA (same day)	32.4	11
FIB-4	Segovia, 2008[43]	Retrospective	219	NA	52.3 (68.95%)	All etiologies	NA (same day)	29.9	10

Legend: TE: transient elastography, APRI: AST-to-platelet ratio index, FIB-4: Fibrosis score 4; NA, not available.

^1^94 biopsies

^2^102 biopsies.

### Characteristics of the included studies

We included eight studies investigating the ability of APRI to diagnose significant fibrosis (F2-4). These studies included a total of 448 LT recipients, of whom 30 were children. Five out of the eight studies focused on HCV patients, who comprised 329 out of the 448 patients. The overall prevalence of significant liver fibrosis was 36.2% (range 20–68.1%). Biopsy quality was considered acceptable in three of these studies[18, 27, 28]. None of the studies included patients with acute rejection.

A total of 435 patients were included in the four FIB-4 studies. The overall prevalence of significant liver fibrosis was 39.8% (range 28–68.1%). Two of these studies comprised only HCV-infected patients, while two included patients of mixed etiology of liver disease. Biopsy quality was considered acceptable in one of these studies[27].

In the twelve TE studies, a total of 1,196 LT recipients were included. The overall prevalence of significant liver fibrosis was 37.4% (range 27.1–68.1%). Six of these studies comprised only HCV-infected patients, while six included patients of mixed etiology of liver disease. Biopsy quality was considered acceptable in seven of these studies[18, 20, 27, 29, 30].

According to the QUADAS scale, the methodological quality of the included studies was good. Two studies met all 14 requirements of this scale, four studies met all but one of these requirements.

### Diagnostic accuracy of APRI

**Fig 2:**
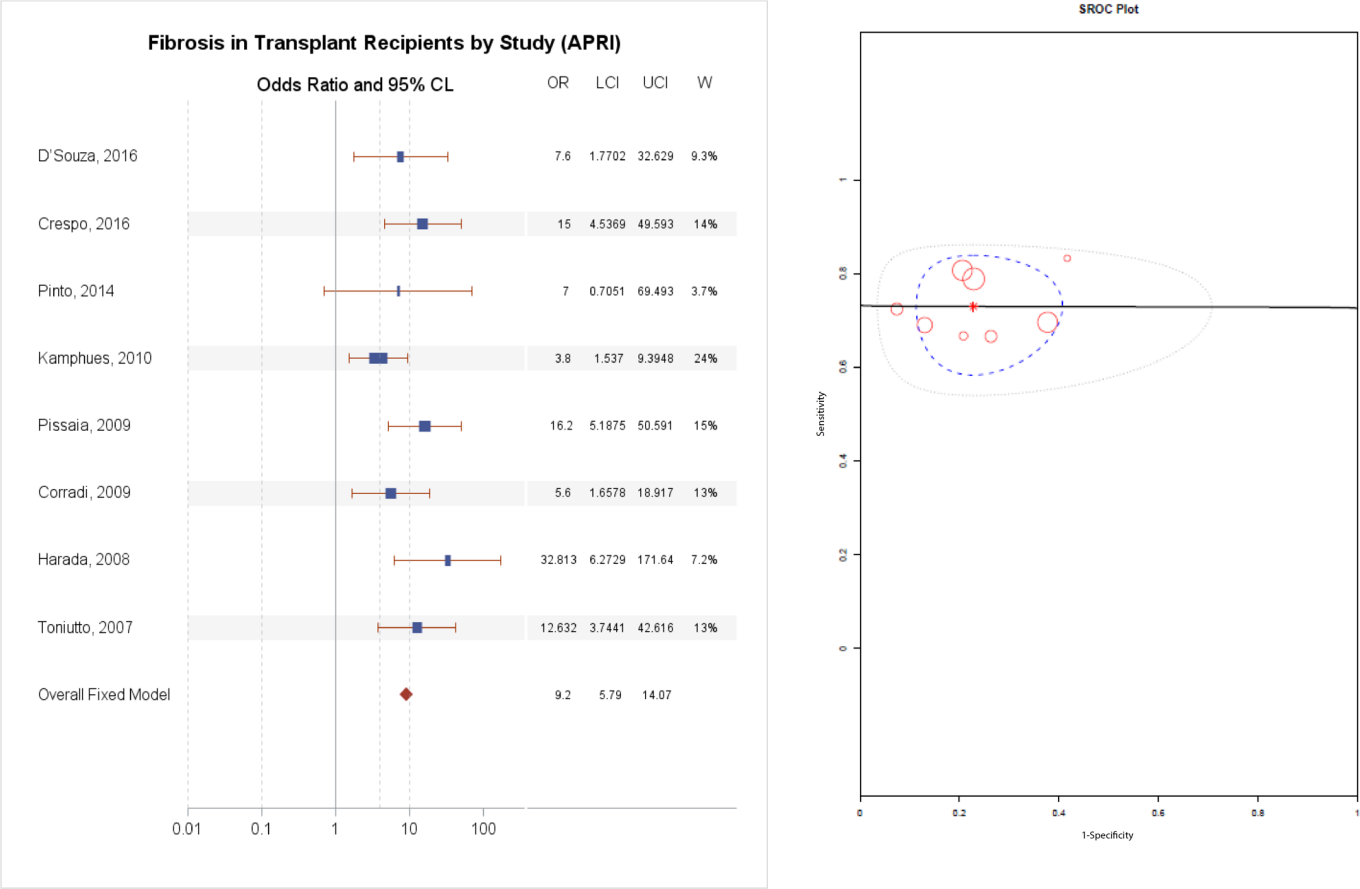
(A) Diagnostic accuracy of APRI for the prediction of F2–4 fibrosis, (B) Summary Receiver Operating Curve.

The AUC for significant fibrosis ranged from a low of 0.50 to a high of 0.83 for HCV, as reported in Table 3. The APRI test fared better in the studies of patients with all etiologies, with an AUC ranging from 0.74 to 0.87. As illustrated in Fig 2, the summary odds ratio for APRI was 9.20 (95% CI 5.79–14.07, p = 1X10^-30^).

**Table 3:** Diagnostic accuracy of non-invasive tests to predict fibrosis F2-4 in individual studies included in the meta-analysis.

Author, Year	Test	Cut-off	Sensitivity (%)	Specificity (%)	PPV (%)	NPV (%)	AUC (95% CI)
Della-Guardia, 2017	TE	12.3 kPa	43	91	72	76	0.78 (0.71–0.85)
Crespo, 2016	TE	6.8 kPa	73	71	57	83	0.76 (0.64–0.88)
Mikołajczyk-Korniak, 2016[38]	TE	4.7 kPa	93	57	90	66	0.746 (0.53–0.95)
Lutz, 2016	TE	8.35 kPa	84.6	91.4			0.89+/-0.057
Barrault, 2013	TE	7 kPa	88.2	67.9	62.5	90.5	0.83 (0.71–0.95)
Goldsmith, 2013	TE	9.3 kPa	90	81	75	93	0.93 (NA)
Crespo, 2012	TE	8.4 kPa	82	80	76	86	0.87 (NA)
Sebagh, 2012	TE	7.9 kPa	62.5	66.7	45	80	NA
Beckebaum, 2010	TE	7.3 kPa	73	100	100	52	0.86
Corradi, 2009	TE	10.1 kPa	94	89	81	94	0.94 (0.85–0.99)
Harada, 2008	TE	9.9 kPa	90	91	86	94	0.92 (NA)
Rigamonti, 2008	TE	7.9 kPa	81	76	65	88	0.85 (0.76–0.92)
Carrion, 2006	TE	8.5 kPa	90	81	79	92	0.90 (NA)
D’Souza, 2016	APRI	0.45	67	79	67	79	0.74 (0.57–0.91)
Crespo, 2016	APRI	1.36	69	87	75	83	0.83 (0.73–0.94)
Pinto, 2014	APRI	0.4	83	58	31	94	0.74 (0.54–0.95)
Kamphues, 2010	APRI	0.4845	70	63	80	80	0.68 (NA)
Carrion, 2010	APRI	-	-	-	-	-	0.50
Pissaia, 2009	APRI	0.5	81	80	62	91	0.87 (NA)
Corradi, 2010	APRI	1.3	67	74	55	82	0.82 (0.64–0.99)
Harada, 2008	APRI	0.84	73	91	63	76	0.70 (NA)
Toniutto, 2007	APRI	1.4	76	77	46	93	0.80 (NA)
Crespo, 2016	FIB-4	3.23	77	80	69	86	0.81 (0.70–0.92)
Kamphues, 2010	FIB-4	2.8	44	87	88	42	0.66 (NA)
Pissaia, 2009	FIB-4	3.25	31	94	67	77	0.78 (NA)
Segovia, 2008	FIB-4	4.09	80	60	25	94	NA

Legend: TE: transient elastography, APRI: AST-to-platelet ratio index, FIB-4: Fibrosis score 4; NA, not available.

### Diagnostic accuracy of FIB-4

**Fig 3:**
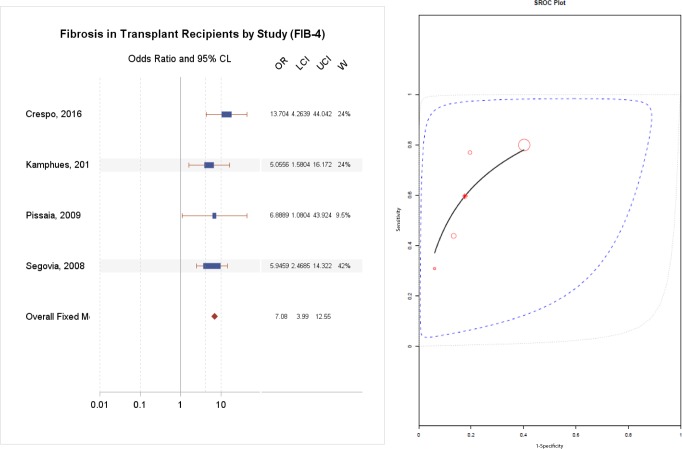
(A) Diagnostic accuracy of FIB-4 for the prediction of F2-4 fibrosis, (B) Summary Receiver Operating Curve.

The AUC for FIB-4 ranged from 0.66 to 0.81 for HCV and an AUC of 0.78 for mixed etiologies, as reported in Table 3. As demonstrated in Fig 3, the summary odds ratio for FIB-4 was 7.08 (95% CI 4.00–12.55, p = 1.93X10^-11^).

### Diagnostic accuracy of TE

**Fig 4:**
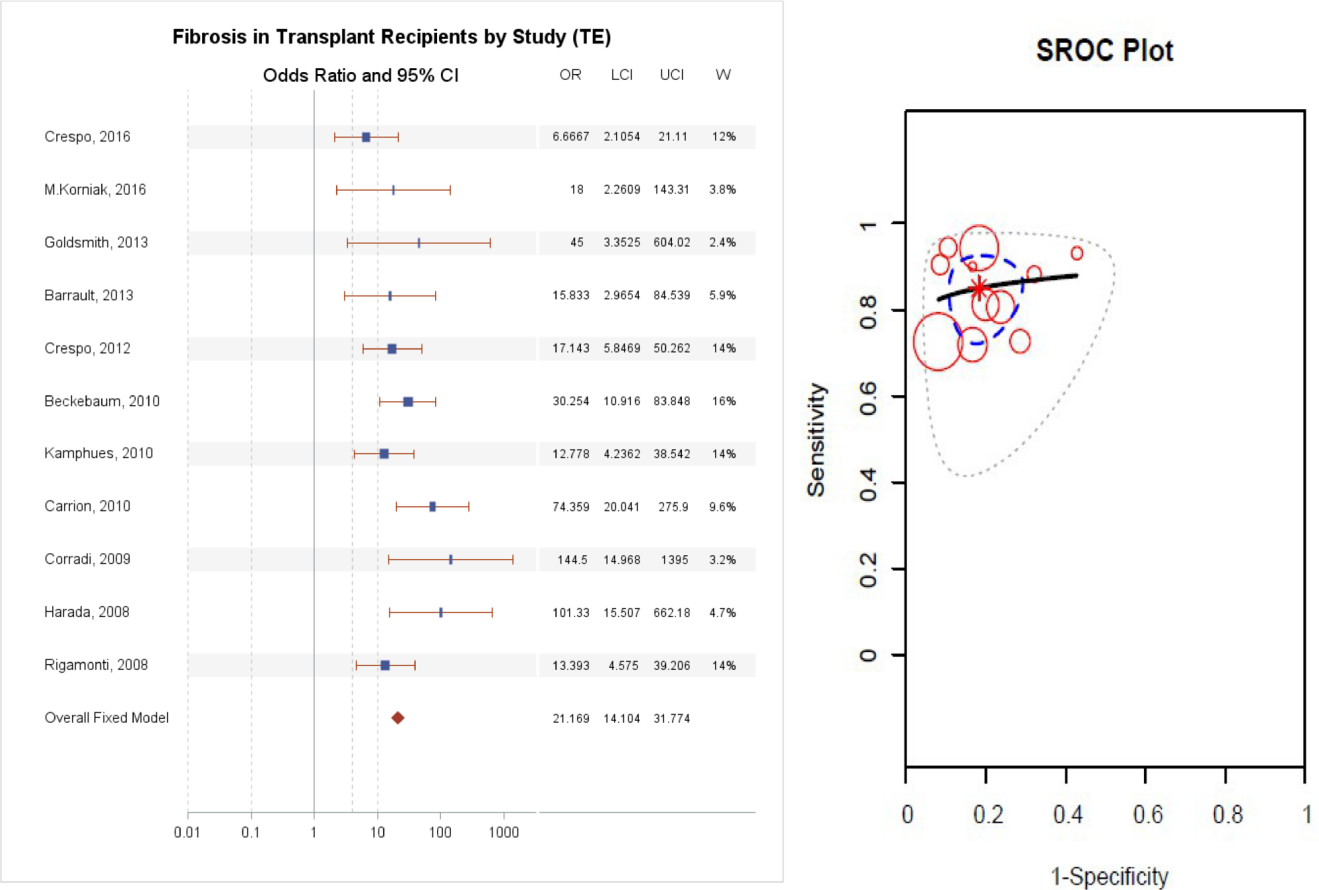
(A) Diagnostic accuracy of Fibroscan for the prediction of F2-4 fibrosis, (B) Summary Receiver Operating Curve.

**Fig 5:**
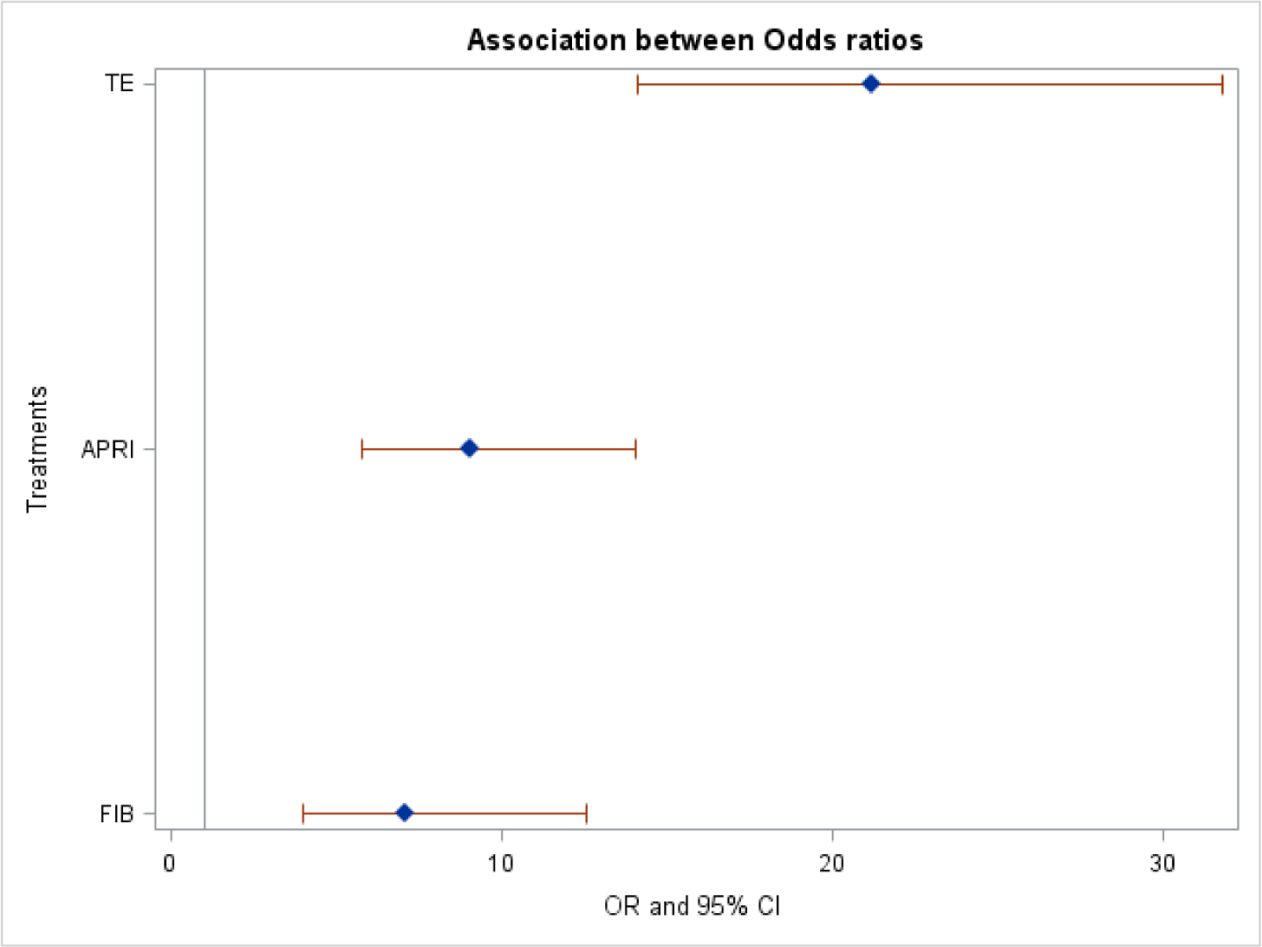
Direct comparison of diagnostic accuracy of non-invasive tests. There was no significant difference between APRI and FIB-4 (P>0.05), but there was a significant difference between TE and FIB-4 (P<0.05) and between TE and APRI (P<0.05).

TE detected significant fibrosis with an AUC ranging from 0.75 to 0.96, as reported in Table 3. Two small prospective pediatric studies comprising 50 patients with various indications for LT revealed different results: the study of 24 patients negative for viral hepatitis had an AUC of 0.71, whereas a study of 26 patients with all etiologies had an excellent AUC of 0.93. As illustrated in Fig 4, the summary odds ratio for TE was the best, at 21.17 (95% CI 14.10–31.77, p = 1X10^-30^), having excluded one study due to publication bias as elucidated below. A comparison of diagnostic accuracy of the three tests is illustrated in Fig 5. There was no significant difference between APRI and FIB4 (p>0.05), but there was a significant difference between TE and FIB4 (p<0.05) and between TE and APRI (P<0.05).

### Publication bias

**Fig 6:**
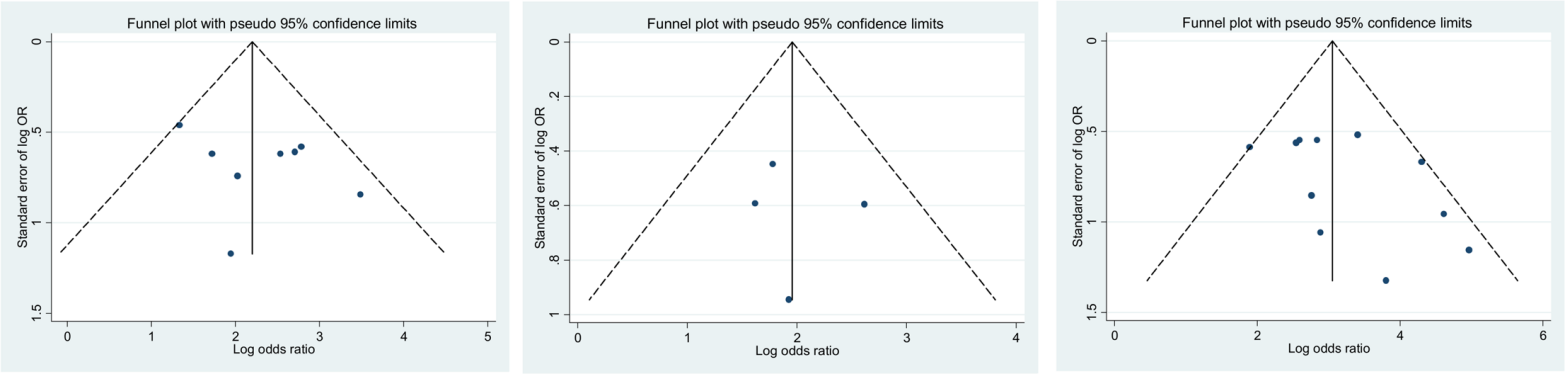
Funnel plots demonstrating the accuracy of the APRI (A), FIB-4 (B) and Fibroscan (C) versus the inverse of the square root of the effective sample size (ESS) for the prediction of F2–4 fibrosis. We derived the funnel plot and Egger test to the accuracy of meta-analysis inferences. Continuous lines represent the overall estimates of test accuracy. The Egger test did not detect a significant publication bias (p>0.05). For Fibroscan, the Egger test shows a borderline significant p-value (p = 0.0535) for Fibroscan data, which is driven by an outlier, the study by Carrion et al (2006).

Funnel plots of diagnostic accuracy versus effective sample size for the prediction of significant liver fibrosis are illustrated in Fig 6. For APRI and FIB-4 (Fig 6A and 6B), the funnel plot did not detect a significant publication bias (p>0.05). For TE (Fig 6C), there was evidence of publication bias with a single study. The Egger test for the remaining 11 studies showed no evidence of publication bias (p>0.05). The sensitivity analysis including study quality, prevalence of significant fibrosis, and quality of the reference standard, did not explain this asymmetry.

## Discussion

In this meta-analysis, we have evaluated the diagnostic accuracy of three non-invasive diagnostic tests to stage liver fibrosis in the post-LT setting. The identification of significant liver fibrosis is pivotal since it is a clear indication of recurrence of the primary disease (e.g. hepatitis C) or the occurrence of *de novo* liver disease (e.g. NAFLD) triggered by multiple risk factors related to immunosuppression or metabolic disorders which occur frequently after LT. Our data indicate that TE has good accuracy in detecting significant liver fibrosis in LT recipients and outperforms simple serum biomarkers, such as APRI and FIB-4. Our work has the strength of including all etiologies of liver disease and comparing TE with simple serum biomarkers. To our knowledge, there is only a single systematic review that included TE, APRI and FIB-4, however it was conducted only in HCV-positive recipients[31].

The development and validation of non-invasive diagnostic methods to diagnose liver fibrosis has gained much attention in recent years. These efforts have proceeded in parallel with increased evidence of the limitations and drawbacks of liver biopsy. Indeed, liver biopsy is an invasive and costly procedure, which could potentially lead to serious complications including bleeding and pneumothorax[17]. Moreover, there are intrinsic limitations in histological determination. Indeed, the quality and size of the specimen obtained through a percutaneous liver biopsy greatly affect the diagnostic reliability. Intra-observer and inter-observer variability have also been extensively studied. It has been suggested that liver biopsy is the best available standard of reference for fibrosis evaluation, although an imperfect gold standard[32].

Non-invasive methods for liver fibrosis have been extensively validated for the main etiologies of chronic liver diseases in the pre-transplant setting. TE and serum fibrosis biomarkers are the most validated tests and are recommended by recent guidelines. The recently endorsed guidelines of the European Association for the Study of the Liver (EASL) on the non-invasive diagnosis of liver fibrosis suggest that non-invasive methods could substitute liver biopsy when combined in the pre-transplant setting[17]. However, fewer data are available in LT recipients. After careful selection of available studies through a quality control check, we included 24 studies evaluating the performance of non-invasive diagnostic tools for liver fibrosis against liver histology. Although some of them included only patients with HCV, several included mixed etiologies of liver disease, such as alcoholic liver disease, NAFLD, cholestasis. There have been few studies employing other non-invasive tools, such as NAFLD fibrosis score, Magnetic Resonance Elastography (MRE) or Acoustic Radiation Force Impulse imaging (ARFI). However, we excluded those non-invasive tests either for poor quality of the studies or for the limited number of studies conducted, insufficient to perform a meta-analysis.

The primary outcome of our meta-analysis was the identification of significant liver fibrosis (stage F2-4), which indicates a progressive liver fibrosis that will eventually lead to liver cirrhosis if appropriate interventions are not implemented. In the specific setting of LT, significant liver fibrosis may mean both recurrence of primary liver disease (hepatitis C, NASH) or a *de novo* liver disease. In any case, it is a clear signal that further investigations are needed. Only a previous meta-analysis reported APRI’s performance in LT recipients and this was superior to other simple non-invasive diagnostic tests[31]. In our experience, there was no significant difference between the two simple fibrosis biomarkers APRI and FIB-4 in terms of diagnostic performance. Importantly, we did not find a significant publication bias in the studies including these two biomarkers, and this was confirmed by absence of statistically significant asymmetry on the funnel plots. APRI and FIB-4 have been employed serially in LT recipients not only for diagnostic purposes, but also for prognosis. The fact that both biomarkers and their longitudinal changes predict long-term outcomes in the recipient, including death and graft loss, confirms their pathophysiologic link with liver damage[33]. A major issue for both APRI and FIB-4 remains the wide range of cut-off values adopted in different studies to diagnose significant liver fibrosis, which may limit their applicability in clinical practice. Patented panels of fibrosis biomarkers, such as Fibrotest and ELF, have not been included in the present meta-analysis. Indeed, there are only a few studies which investigated the diagnostic accuracy of these tests in LT and they reported an unsatisfactory performance. This could be due to suboptimal adherence of the laboratory to pre-analytic recommendations to obtain reliable results[34] or on specific alterations of the individual components of the patented panels that could occur following LT due to chronic inflammation (elevation of alpha-2-macroglobulin, hyaluronan), hemolytic anemia (haptoglobin), multidrug induction (GGT) or cholestasis (bilirubin).

TE had the highest number of included studies among the three non-invasive methods we evaluated. In the pre-transplant setting, TE has been shown to outperform simple serum biomarkers to diagnose liver cirrhosis[17]. This gap in diagnostic accuracy is smaller for the detection of significant liver fibrosis. In the present study, we found that TE had higher diagnostic accuracy than APRI and FIB-4 to detect significant liver fibrosis in the post-transplant setting. Diagnostic accuracy was consistent in both HCV patients and other etiologies of liver disease. There was a tendency for a significant publication bias for TE, which was driven by only one study. However, we were not able to find any predictor of this marginally significant asymmetry with the sensitivity analysis. Interestingly, there have been several studies investigating the use of TE for serial assessment in LT recipients in order to identify liver fibrosis progression. On this topic, Rigamonti *et al* conducted two studies, one in patients transplanted for HCV and one in those transplanted for non-viral etiologies[30, 35]. In 95 patients transplanted for end-stage liver disease due to HCV and undergoing serial liver biopsies, the authors found an AUC of 0.85 for significant liver fibrosis, concluding that protocol liver biopsy could be avoided in patients with stable TE during follow-up. Along the same lines, in 69 LT recipients the same group found that TE allow accurate discrimination between presence or absence of liver graft damage, thus helping the selection of patients most in need of liver biopsy.

The EASL guidelines on non-invasive evaluation of liver fibrosis suggest combining TE with a serum biomarker in order to reduce the number of liver biopsies needed for correct clinical management[17]. The avoidance of a liver biopsy becomes a more complicated matter when dealing with the post-transplant setting. Indeed, in LT recipients, it is not only liver fibrosis stage that may cause graft damage, but also multiple liver pathologies that arise from the complex interaction between primary liver disease recurrence (hepatitis C, NAFLD), development of metabolic syndrome in the context of immunosuppression (calcineurin and mTOR inhibitors), and possible acute or chronic rejection. However, given the good accuracy we found in this meta-analysis, especially for TE, we feel that non-invasive methods for liver fibrosis could be used to reduce the number of liver biopsies in LT recipients. We acknowledge that non-invasive methods should always be used having carefully ascertained the clinical context and correlated with liver biochemistry and function tests.

Our study has certain limitations, including the higher proportion of HCV patients represented. We did also include pediatric studies, with various reasons for development of fibrosis post-transplant. Finally, the reported range of cut-off values for TE is wide (from 7.3 kPa to 12.3 kPa) in LT recipients, rendering potentially complicated the use in clinical practice for the individual patients. This finding is likely due to highly heterogeneous study populations due to etiology of liver disease, different post-LT time points for the assessment of liver fibrosis, study design and sample size and variable interval range between liver biopsy and the non-invasive assessment of liver fibrosis. We could not account for these different cut-offs for transient elastography for the various etiologies of CLD in our meta-analysis, as these were not provided in the studies. Nonetheless, the validity of our findings across this heterogeneous population suggests the usefulness of these non-invasive tools for fibrosis even in a complex context such as that of LT.

## Conclusions

In conclusion, given non-invasiveness and feasibility for serial measurements, non-invasive tests for liver fibrosis could be used in the post-transplant clinical setting as an additive tool for suspected recurrent or *de novo* liver disease. The high accuracy we found in our meta-analysis, especially for TE, suggests that these tests have similar diagnostic value as in the pre-transplant setting. It should be underlined that liver biopsy remains a cornerstone for the clinical management of LT recipients, as non-invasive tests cannot differentiate between different liver pathologies that can coexist in this setting, such as acute or chronic rejection. Nonetheless, when the etiology of recurrent or *de novo* disease has been established, these non-invasive tests are helpful in following fibrosis progression longitudinally and implementing preventive therapies in a timely manner. Further studies aimed at defining optimal cut-off values for definition of significant liver fibrosis are warranted.

## Supporting information

S1 PRISMA Checklist (DOC)

S1 File Pubmed results for “liver transplantation” AND fibrosis. (TXT)

S2 File Pubmed results for “liver transplantation” AND "serum fibrosis markers". (TXT)

S3 File Pubmed results for “liver transplantation” AND "APRI". (TXT)

S4 File Pubmed results for “liver transplantation” AND "FIB-4". (TXT)

S5 File Pubmed results for “liver transplantation” AND "transient elastography". (TXT)

S6 File Pubmed results for “liver transplantation” AND "liver stiffness measurement". (TXT)

S7 File Cochrane library database results for "Liver transplantation" AND fibrosis. (TXT)

S8 File Cochrane library database results for "Liver transplantation" AND "APRI". (TXT)

S9 File Cochrane library database results for "Liver transplantation" AND "FIB-4". (TXT)

S10 File Cochrane library database results for "Liver transplantation" AND "transient elastography". (TXT)

S11 File Cochrane library database results for "Liver transplantation" AND “liver stiffness measurement”. (TXT)

S12 File Complete EMBASE search. (DOCX)

## Abbreviations

LT: Liver transplantation
APRI: AST-to-platelet ratio index
FIB-4: Fibrosis score 4
TE: Transient elastography
AUC: Area under the curve
CI: Confidence interval
