# Supplementary material for: Performance of transient elastography and serum fibrosis biomarkers for non-invasive evaluation of recurrent fibrosis after liver transplantation: A meta-analysis
Source: PLoS One. 2017 Sep 27;12(9):e0185192. doi: 10.1371/journal.pone.0185192 (PMC5617176; doi:10.1371/journal.pone.0185192)
Supplement: S12 File — (DOCX) [file pone.0185192.s013.docx]

[Skip to Main content](http://www.sciencedirect.com/search?qs=%22Liver%20transplantation%22%20and%20APRI&show=100&sortBy=relevance&articleTypes=OR#main_content)

- [Journals](http://www.sciencedirect.com/science/journals)
- [Books](http://www.sciencedirect.com/science/bookbshsrw)
- [Register](https://www.sciencedirect.com/user/register?returnURL=http%3A%2F%2Fwww.sciencedirect.com%2Fsearch)
- [Sign in](https://www.sciencedirect.com/user/login?returnURL=http%3A%2F%2Fwww.sciencedirect.com%2Fsearch)

Top of Form


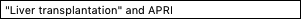


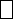


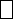


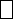


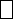


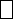


[Advanced search](http://www.sciencedirect.com/science/search)

Bottom of Form

148 results

Refine by:

Top of Form

Years

1.
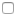
 2017 (17)
2.
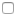
 2016 (18)
3.
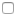
 2015 (19)
4.
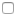
 2014 (19)
5.
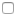
 2013 (9)
6.
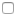
 2012 (12)
7.
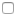
 2011 (12)
8.
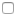
 2010 (13)
9.
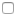
 2009 (9)
10.
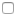
 2008 (12)

Show less

Article type

1.
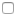
 Original research (148)

Publication title

1.
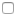
 Journal of Hepatology (24)
2.
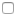
 Clinical Gastroenterology and Hepatology (15)
3.
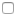
 Gastroenterology (12)
4.
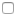
 Journal of Pediatric Surgery (9)
5.
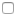
 Digestive and Liver Disease (9)
6.
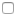
 Antiviral Research (4)
7.
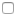
 Gastroenterología y Hepatología (4)
8.
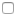
 Clinical Biochemistry (3)
9.
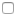
 Best Practice & Research Clinical Gastroenterology (3)
10.
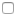
 Enfermedades Infecciosas y Microbiología Clínica (3)

Show less

[Clear all filters](http://www.sciencedirect.com/search?qs=%22Liver%20transplantation%22%20and%20APRI&show=100&sortBy=relevance&navigation=true)

Bottom of Form

sorted by *relevance* | [date](http://www.sciencedirect.com/search?qs=%22Liver%20transplantation%22%20and%20APRI&show=100&sortBy=date&articleTypes=OR&navigation=true)

[Skip to Main content](http://www.sciencedirect.com/search?qs=%22Liver%20transplantation%22%20and%20APRI&show=100&sortBy=relevance&articleTypes=OR&navigation=true&offset=0#main_content)

- [Journals](http://www.sciencedirect.com/science/journals)
- [Books](http://www.sciencedirect.com/science/bookbshsrw)
- [Register](https://www.sciencedirect.com/user/register?returnURL=http%3A%2F%2Fwww.sciencedirect.com%2Fsearch)
- [Sign in](https://www.sciencedirect.com/user/login?returnURL=http%3A%2F%2Fwww.sciencedirect.com%2Fsearch)

Top of Form


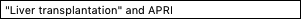


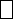


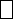


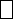


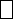


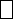


[Advanced search](http://www.sciencedirect.com/science/search)

Bottom of Form

148 results

Refine by:

Top of Form

Years

1.
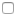
 2017 (17)
2.
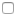
 2016 (18)
3.
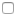
 2015 (19)
4.
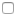
 2014 (19)
5.
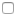
 2013 (9)
6.
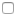
 2012 (12)
7.
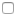
 2011 (12)
8.
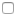
 2010 (13)
9.
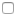
 2009 (9)
10.
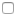
 2008 (12)

Show less

Article type

1.
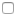
 Original research (148)

Publication title

1.
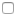
 Journal of Hepatology (24)
2.
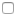
 Clinical Gastroenterology and Hepatology (15)
3.
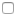
 Gastroenterology (12)
4.
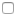
 Journal of Pediatric Surgery (9)
5.
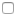
 Digestive and Liver Disease (9)
6.
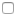
 Antiviral Research (4)
7.
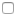
 Gastroenterología y Hepatología (4)
8.
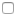
 Clinical Biochemistry (3)
9.
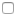
 Best Practice & Research Clinical Gastroenterology (3)
10.
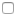
 Enfermedades Infecciosas y Microbiología Clínica (3)

Show less

[Clear all filters](http://www.sciencedirect.com/search?qs=%22Liver%20transplantation%22%20and%20APRI&show=100&sortBy=relevance&navigation=true&offset=0)

Bottom of Form

sorted by *relevance* | [date](http://www.sciencedirect.com/search?qs=%22Liver%20transplantation%22%20and%20APRI&show=100&sortBy=date&articleTypes=OR&navigation=true&offset=0)

1. [APRI and FIB-4 Scores Are Useful After Liver Transplantation Independently of Etiology](http://www.sciencedirect.com/science/article/pii/S0041134508016564)
   1. Original research article
   2. Transplantation Proceedings,
   3. Volume 41, Issue 2,
   4. March 2009,
   5. Pages 679-681
   6. A. Pissaia,
   7. D. Borderie,
   8. D. Bernard,
   9. O. Scatton,
   10. F. Conti
   11. [Purchase PDF](http://www.sciencedirect.com/science/article/pii/S0041134508016564)
2. [APRi predicts native liver survival by reflecting portal fibrogenesis and hepatic neovascularization at the time of portoenterostomy in biliary atresia](http://www.sciencedirect.com/science/article/pii/S0022346814007921)
   1. Original research article
   2. Journal of Pediatric Surgery,
   3. Volume 50, Issue 9,
   4. September 2015,
   5. Pages 1528-1531
   6. Janne S. Suominen,
   7. Hanna Lampela,
   8. Päivi Heikkilä,
   9. Jouko Lohi,
   10. Mikko P. Pakarinen
   11. [Purchase PDF](http://www.sciencedirect.com/science/article/pii/S0022346814007921)
3. Want a richer search experience?

Sign in for additional filter options, multiple article downloads, and more.

Top of Form

Sign in

Bottom of Form

1. [Diagnostic accuracy of APRI and FIB-4 for predicting hepatitis B virus-related liver fibrosis accompanied with hepatocellular carcinoma](http://www.sciencedirect.com/science/article/pii/S1590865815304278)
   1. Original research article
   2. Digestive and Liver Disease,
   3. Volume 48, Issue 10,
   4. October 2016,
   5. Pages 1220-1226
   6. Guangqin Xiao,
   7. Feng Zhu,
   8. Min Wang,
   9. Hang Zhang,
   10. Renyi Qin
   11. [Purchase PDF](http://www.sciencedirect.com/science/article/pii/S1590865815304278)
2. [Aspartate Aminotransferase-to-Platelet Ratio index (APRi) in infants with biliary atresia: Prognostic value at presentation](http://www.sciencedirect.com/science/article/pii/S0022346812007828)
   1. Original research article
   2. Journal of Pediatric Surgery,
   3. Volume 48, Issue 4,
   4. April 2013,
   5. Pages 789-795
   6. Andrew Grieve,
   7. Erica Makin,
   8. Mark Davenport
   9. [Purchase PDF](http://www.sciencedirect.com/science/article/pii/S0022346812007828)
3. [Features of Severe Liver Disease With Portal Hypertension in Patients With Cystic Fibrosis](http://www.sciencedirect.com/science/article/pii/S1542356516300167)
   1. Original research article
   2. Clinical Gastroenterology and Hepatology,
   3. Volume 14, Issue 8,
   4. August 2016,
   5. Pages 1207-1215.e3
   6. Jaclyn R. Stonebraker,
   7. Chee Y. Ooi,
   8. Rhonda G. Pace,
   9. Harriet Corvol,
   10. Simon C. Ling
   11. [Purchase PDF](http://www.sciencedirect.com/science/article/pii/S1542356516300167)
4. [Prediction on liver fibrosis using different APRI thresholds when patient age is a categorical marker in patients with chronic hepatitis B](http://www.sciencedirect.com/science/article/pii/S0009898110005292)
   1. Original research article
   2. Clinica Chimica Acta,
   3. Volume 412, Issues 1–2,
   4. 14 January 2011,
   5. Pages 33-37
   6. Hong-Bo Liu,
   7. Jian-Ping Zhou,
   8. Yong Zhang,
   9. Xiao-Hui Lv,
   10. Wei Wang
   11. [Purchase PDF](http://www.sciencedirect.com/science/article/pii/S0009898110005292)
5. [Optimising risk stratification in primary biliary cirrhosis: AST/platelet ratio index predicts outcome independent of ursodeoxycholic acid response](http://www.sciencedirect.com/science/article/pii/S0168827814001056)
   1. Original research article
   2. Journal of Hepatology,
   3. Volume 60, Issue 6,
   4. June 2014,
   5. Pages 1249-1258
   6. Palak J. Trivedi,
   7. Tony Bruns,
   8. Angela Cheung,
   9. Ka-Kit Li,
   10. Gideon M. Hirschfield
   11. [Purchase PDF](http://www.sciencedirect.com/science/article/pii/S0168827814001056)
6. [Use of noninvasive markers to predict advanced fibrosis/cirrhosis in severe obesity](http://www.sciencedirect.com/science/article/pii/S1550728915010515)
   1. Original research article
   2. Surgery for Obesity and Related Diseases,
   3. Volume 12, Issue 4,
   4. May 2016,
   5. Pages 862-867
   6. Roberto de Cleva,
   7. Livio Fiolo Duarte,
   8. Milton Roberto Furst Crenitte,
   9. Claudia Pinto Marques de Oliveira,
   10. Marco Aurelio Santo
   11. [Purchase PDF](http://www.sciencedirect.com/science/article/pii/S1550728915010515)
7. [Fibrosis index based on four factors better predicts advanced fibrosis or cirrhosis than aspartate aminotransferase/platelet ratio index in chronic hepatitis C patients](http://www.sciencedirect.com/science/article/pii/S0929664615002417)
   1. Open archive,
   2. Original research article
   3. Journal of the Formosan Medical Association,
   4. Volume 114, Issue 10,
   5. October 2015,
   6. Pages 923-928
   7. Chia-Chi Wang,
   8. Chen-Hua Liu,
   9. Chih-Lin Lin,
   10. Pin-Chao Wang,
   11. Jia-Horng Kao
   12. [Download PDF (375 KB)](http://www.sciencedirect.com/science/article/pii/S0929664615002417/pdfft?md5=68465eac6e84326a9e1d1acb9f5c709f&pid=1-s2.0-S0929664615002417-main.pdf)
8. [Preoperative inflammation-based markers predict early and late recurrence of hepatocellular carcinoma after curative hepatectomy](http://www.sciencedirect.com/science/article/pii/S1499387216600942)
   1. Original research article
   2. Hepatobiliary & Pancreatic Diseases International,
   3. Volume 15, Issue 3,
   4. June 2016,
   5. Pages 266-274
   6. Yue Liu,
   7. Zhong-Xia Wang,
   8. Yin Cao,
   9. Guang Zhang,
   10. Chun-Ping Jiang
   11. [Purchase PDF](http://www.sciencedirect.com/science/article/pii/S1499387216600942)
9. [Serum Fibrosis Markers Identify Patients With Mild and Progressive Hepatitis C Recurrence After LiverTransplantation](http://www.sciencedirect.com/science/article/pii/S0016508509016977)
   1. Original research article
   2. Gastroenterology,
   3. Volume 138, Issue 1,
   4. January 2010,
   5. Pages 147-158.e1
   6. José A. Carrión,
   7. Guillermo Fernández–Varo,
   8. Miquel Bruguera,
   9. Juan–Carlos García–Pagán,
   10. Miquel Navasa
   11. [Purchase PDF](http://www.sciencedirect.com/science/article/pii/S0016508509016977)
10. [Serum and tissue tumor growth factor β1 in children with biliary atresia](http://www.sciencedirect.com/science/article/pii/S0022346810003611)
    1. Original research article
    2. Journal of Pediatric Surgery,
    3. Volume 45, Issue 9,
    4. September 2010,
    5. Pages 1784-1790
    6. Fernanda dos Santos de Oliveira,
    7. Carlos Oscar Kieling,
    8. Jorge Luiz dos Santos,
    9. Patrícia Ponce de Leon Lima,
    10. Ursula Matte
    11. [Purchase PDF](http://www.sciencedirect.com/science/article/pii/S0022346810003611)
11. [Assessment of liver fibrosis in transplant recipients with recurrent HCV infection: Usefulness of transient elastography](http://www.sciencedirect.com/science/article/pii/S1590865808002533)
    1. Original research article
    2. Digestive and Liver Disease,
    3. Volume 41, Issue 3,
    4. March 2009,
    5. Pages 217-225
    6. F. Corradi,
    7. F. Piscaglia,
    8. S. Flori,
    9. A. D’Errico-Grigioni,
    10. The Bologna Liver Transplantation Group (BLTG)
    11. [Purchase PDF](http://www.sciencedirect.com/science/article/pii/S1590865808002533)
12. [Prediction of esophageal varices in biliary atresia: Derivation of the “varices prediction rule”, a novel noninvasive predictor](http://www.sciencedirect.com/science/article/pii/S0022346815000871)
    1. Original research article
    2. Journal of Pediatric Surgery,
    3. Volume 50, Issue 10,
    4. October 2015,
    5. Pages 1734-1738
    6. Alexander Isted,
    7. Tassos Grammatikopoulos,
    8. Mark Davenport
    9. [Purchase PDF](http://www.sciencedirect.com/science/article/pii/S0022346815000871)
13. [Use of the aspartate aminotransferase to platelet ratio index to follow liver fibrosis progression in infants with short gut](http://www.sciencedirect.com/science/article/pii/S0022346810002174)
    1. Original research article
    2. Journal of Pediatric Surgery,
    3. Volume 45, Issue 6,
    4. June 2010,
    5. Pages 1266-1273
    6. Richard S. Mangus,
    7. Michael G. O'Connor,
    8. A. Joseph Tector,
    9. Joel D. Lim,
    10. Rodrigo M. Vianna
    11. [Purchase PDF](http://www.sciencedirect.com/science/article/pii/S0022346810002174)
14. [Comparison of Circulating Endothelial Cell/Platelet Count Ratio to Aspartate Transaminase/Platelet Ratio Index for Identifying Patients with Cirrhosis](http://www.sciencedirect.com/science/article/pii/S0973688312600784)
    1. Original research article
    2. Journal of Clinical and Experimental Hepatology,
    3. Volume 2, Issue 1,
    4. March 2012,
    5. Pages 19-26
    6. Saurabh Sethi,
    7. Douglas A Simonetto,
    8. Soha S Abdelmoneim,
    9. Michael B Campion,
    10. Vijay H Shah
    11. [Purchase PDF](http://www.sciencedirect.com/science/article/pii/S0973688312600784)
15. [Marcadores séricos de fibrosis hepática en pacientes con hepatitis crónica C. Valor pronóstico de los marcadores no invasivos de fibrosis en el trasplante de hígado](http://www.sciencedirect.com/science/article/pii/S0210570512700456)
    1. Original research article
    2. Gastroenterología y Hepatología,
    3. Volume 35, Supplement 2,
    4. December 2012,
    5. Pages 17-22
    6. Gonzalo Crespo,
    7. Xavier Forns,
    8. Miquel Navasa
    9. [Purchase PDF](http://www.sciencedirect.com/science/article/pii/S0210570512700456)
16. [3D T1 relaxometry pre and post gadoxetic acid injection for the assessment of liver cirrhosis and liverfunction](http://www.sciencedirect.com/science/article/pii/S0730725X15001587)
    1. Original research article
    2. Magnetic Resonance Imaging,
    3. Volume 33, Issue 9,
    4. November 2015,
    5. Pages 1075-1082
    6. Cecilia Besa,
    7. Octavia Bane,
    8. Guido Jajamovich,
    9. Joseph Marchione,
    10. Bachir Taouli
    11. [Purchase PDF](http://www.sciencedirect.com/science/article/pii/S0730725X15001587)
17. [Surveillance for Hepatocellular Carcinoma in Patients with Cirrhosis Improves Outcome](http://www.sciencedirect.com/science/article/pii/S0002934307009928)
    1. Original research article
    2. The American Journal of Medicine,
    3. Volume 121, Issue 2,
    4. February 2008,
    5. Pages 119-126
    6. Richard Todd Stravitz,
    7. Douglas M. Heuman,
    8. Nisha Chand,
    9. Richard K. Sterling,
    10. Robert A. Fisher
    11. [Purchase PDF](http://www.sciencedirect.com/science/article/pii/S0002934307009928)
18. [Multiple approaches to assess fourteen non-invasive serum indexes for the diagnosis of liver fibrosis in chronic hepatitis C patients](http://www.sciencedirect.com/science/article/pii/S0009912016000874)
    1. Original research article
    2. Clinical Biochemistry,
    3. Volume 49, Issues 7–8,
    4. May 2016,
    5. Pages 560-565
    6. María Jesús Andrés-Otero,
    7. Ignacio De-Blas-Giral,
    8. Juan José Puente-Lanzarote,
    9. Trinidad Serrano-Aulló,
    10. José Manuel Lou-Bonafonte
    11. [Purchase PDF](http://www.sciencedirect.com/science/article/pii/S0009912016000874)
19. [Limited reliability of five non-invasive biomarkers in predicting hepatic fibrosis in chronic HCV mono-infected patients opposed to METAVIR scoring](http://www.sciencedirect.com/science/article/pii/S0344033814002076)
    1. Original research article
    2. Pathology - Research and Practice,
    3. Volume 210, Issue 12,
    4. December 2014,
    5. Pages 922-928
    6. Basem Hasan Elesawy,
    7. Amal Abd El hafez,
    8. Laila Shehata Dorgham,
    9. Ahmad El-Askary
    10. [Purchase PDF](http://www.sciencedirect.com/science/article/pii/S0344033814002076)
20. [Non-invasive tests in prediction of liver fibrosis in chronic hepatitis B and comparison with post-antiviral treatment results](http://www.sciencedirect.com/science/article/pii/S2210740112002070)
    1. Original research article
    2. Clinics and Research in Hepatology and Gastroenterology,
    3. Volume 37, Issue 2,
    4. April 2013,
    5. Pages 152-158
    6. Ömer Başar,
    7. Barış Yımaz,
    8. Fuat Ekiz,
    9. Zeynep Giniş,
    10. Osman Yüksel
    11. [Purchase PDF](http://www.sciencedirect.com/science/article/pii/S2210740112002070)
21. [Prospective comparison of two algorithms combining non-invasive methods for staging liver fibrosis in chronic hepatitis C](http://www.sciencedirect.com/science/article/pii/S016882780900734X)
    1. Original research article
    2. Journal of Hepatology,
    3. Volume 52, Issue 2,
    4. February 2010,
    5. Pages 191-198
    6. Laurent Castéra,
    7. Giada Sebastiani,
    8. Brigitte Le Bail,
    9. Victor de Lédinghen,
    10. Alfredo Alberti
    11. [Purchase PDF](http://www.sciencedirect.com/science/article/pii/S016882780900734X)
22. [Simple Noninvasive Systems Predict Long-term Outcomes of Patients With Nonalcoholic Fatty Liver Disease](http://www.sciencedirect.com/science/article/pii/S0016508513010123)
    1. Original research article
    2. Gastroenterology,
    3. Volume 145, Issue 4,
    4. October 2013,
    5. Pages 782-789.e4
    6. Paul Angulo,
    7. Elisabetta Bugianesi,
    8. Einar S. Bjornsson,
    9. Phunchai Charatcharoenwitthaya,
    10. Jacob George
    11. [Purchase PDF](http://www.sciencedirect.com/science/article/pii/S0016508513010123)
23. [Molecular signature of active fibrogenesis prevails in biliary atresia after successful portoenterostomy](http://www.sciencedirect.com/science/article/pii/S0039606017303124)
    1. Original research article
    2. Surgery,
    3. Volume 162, Issue 3,
    4. September 2017,
    5. Pages 548-556
    6. Anna Kerola,
    7. Hanna Lampela,
    8. Jouko Lohi,
    9. Päivi Heikkilä,
    10. Mikko P. Pakarinen
    11. [Purchase PDF](http://www.sciencedirect.com/science/article/pii/S0039606017303124)
24. [Serum Biomarkers Indicate Long-term Reduction in Liver Fibrosis in Patients With Sustained Virological Response to Treatment for HCV Infection](http://www.sciencedirect.com/science/article/pii/S1542356516000525)
    1. Original research article
    2. Clinical Gastroenterology and Hepatology,
    3. Volume 14, Issue 7,
    4. July 2016,
    5. Pages 1044-1055.e3
    6. Mei Lu,
    7. Jia Li,
    8. Talan Zhang,
    9. Loralee B. Rupp,
    10. Chronic Hepatitis Cohort Study Investigators
    11. [Purchase PDF](http://www.sciencedirect.com/science/article/pii/S1542356516000525)
25. [Early detection in routine clinical practice of cirrhosis and oesophageal varices in chronic hepatitis C: Comparison of transient elastography (FibroScan) with standard laboratory tests and non-invasive scores](http://www.sciencedirect.com/science/article/pii/S0168827808006387)
    1. Original research article
    2. Journal of Hepatology,
    3. Volume 50, Issue 1,
    4. January 2009,
    5. Pages 59-68
    6. Laurent Castéra,
    7. Brigitte Le Bail,
    8. Françoise Roudot-Thoraval,
    9. Pierre-Henri Bernard,
    10. Victor de Lédinghen
    11. [Purchase PDF](http://www.sciencedirect.com/science/article/pii/S0168827808006387)
26. [Evaluation of complement factor 5 variants as genetic risk factors for the development of advanced fibrosis in chronic hepatitis C infection](http://www.sciencedirect.com/science/article/pii/S0168827808003711)
    1. Original research article
    2. Journal of Hepatology,
    3. Volume 49, Issue 3,
    4. September 2008,
    5. Pages 339-345
    6. Juliane Halangk,
    7. Christoph Sarrazin,
    8. Konrad Neumann,
    9. Gero Puhl,
    10. Heiko Witt
    11. [Purchase PDF](http://www.sciencedirect.com/science/article/pii/S0168827808003711)
27. [Relating the liver damage with hepatitis C virus polymorphism in core region and human variables in HIV-1-coinfected patients](http://www.sciencedirect.com/science/article/pii/S1567134810002212)
    1. Original research article
    2. Infection, Genetics and Evolution,
    3. Volume 10, Issue 8,
    4. December 2010,
    5. Pages 1252-1261
    6. Marina Matas,
    7. Antònia Picornell,
    8. Carmen Cifuentes,
    9. Antoni Payeras,
    10. José A. Castro
    11. [Purchase PDF](http://www.sciencedirect.com/science/article/pii/S1567134810002212)
28. [Liver and spleen transient elastography and Acoustic Radiation Force Impulse Measurements. Performance and comparison of measurements in the same area concurrently assessed for liver fibrosis by biopsy](http://www.sciencedirect.com/science/article/pii/S1896112615000334)
    1. Original research article
    2. Advances in Medical Sciences,
    3. Volume 60, Issue 2,
    4. September 2015,
    5. Pages 300-306
    6. Francesca M. Trovato,
    7. Sebastiana Atzori,
    8. Giuseppe Musumeci,
    9. Vanessa Tooley,
    10. Simon D. Taylor-Robinson
    11. [Purchase PDF](http://www.sciencedirect.com/science/article/pii/S1896112615000334)
29. [Noninvasive Tests for Fibrosis and Liver Stiffness Predict 5-Year Outcomes of Patients With Chronic Hepatitis C](http://www.sciencedirect.com/science/article/pii/S0016508511002708)
    1. Original research article
    2. Gastroenterology,
    3. Volume 140, Issue 7,
    4. June 2011,
    5. Pages 1970-1979.e3
    6. Julien Vergniol,
    7. Juliette Foucher,
    8. Eric Terrebonne,
    9. Pierre–Henri Bernard,
    10. Victor de Ledinghen
    11. [Purchase PDF](http://www.sciencedirect.com/science/article/pii/S0016508511002708)
30. [Similar Effectiveness of Boceprevir and Telaprevir Treatment Regimens for Hepatitis C Virus Infection on the Basis of a Nationwide Study of Veterans](http://www.sciencedirect.com/science/article/pii/S1542356513019381)
    1. Original research article
    2. Clinical Gastroenterology and Hepatology,
    3. Volume 12, Issue 8,
    4. August 2014,
    5. Pages 1371-1380
    6. George N. Ioannou,
    7. Lauren A. Beste,
    8. Pamela K. Green
    9. [Purchase PDF](http://www.sciencedirect.com/science/article/pii/S1542356513019381)
31. [Steroids in biliary atresia: Single surgeon, single centre, prospective study](http://www.sciencedirect.com/science/article/pii/S0168827813004285)
    1. Original research article
    2. Journal of Hepatology,
    3. Volume 59, Issue 5,
    4. November 2013,
    5. Pages 1054-1058
    6. Mark Davenport,
    7. Chris Parsons,
    8. Sarah Tizzard,
    9. Nedim Hadzic
    10. [Purchase PDF](http://www.sciencedirect.com/science/article/pii/S0168827813004285)
32. [The degree of spleen stiffness measured on acoustic radiation force impulse elastography predicts the severity of portal hypertension in patients with biliary atresia after portoenterostomy](http://www.sciencedirect.com/science/article/pii/S0022346815000433)
    1. Original research article
    2. Journal of Pediatric Surgery,
    3. Volume 50, Issue 4,
    4. April 2015,
    5. Pages 559-564
    6. Hajime Uchida,
    7. Seisuke Sakamoto,
    8. Megumi Kobayashi,
    9. Takanobu Shigeta,
    10. Mureo Kasahara
    11. [Purchase PDF](http://www.sciencedirect.com/science/article/pii/S0022346815000433)
33. [Lactic acidosis in patients with hepatitis C virus cirrhosis and combined ribavirin/sofosbuvir treatment](http://www.sciencedirect.com/science/article/pii/S0168827815007928)
    1. Original research article
    2. Journal of Hepatology,
    3. Volume 64, Issue 4,
    4. April 2016,
    5. Pages 790-799
    6. Martin-Walter Welker,
    7. Stefan Luhne,
    8. Christian M. Lange,
    9. Johannes Vermehren,
    10. Christoph Sarrazin
    11. [Purchase PDF](http://www.sciencedirect.com/science/article/pii/S0168827815007928)
34. [Development and Validation of a Scoring System to Predict Outcomes of Patients With Primary Biliary Cirrhosis Receiving Ursodeoxycholic Acid Therapy](http://www.sciencedirect.com/science/article/pii/S001650851501094X)
    1. Original research article
    2. Gastroenterology,
    3. Volume 149, Issue 7,
    4. December 2015,
    5. Pages 1804-1812.e4
    6. Willem J. Lammers,
    7. Gideon M. Hirschfield,
    8. Christophe Corpechot,
    9. Frederik Nevens,
    10. Global PBC Study Group
    11. [Purchase PDF](http://www.sciencedirect.com/science/article/pii/S001650851501094X)
35. [Reaching hepatitis C virus elimination targets requires health system interventions to enhance the care cascade](http://www.sciencedirect.com/science/article/pii/S0955395917302086)
    1. Original research article
    2. International Journal of Drug Policy,
    3. In press, corrected proof,
    4. Available online 7 August 2017
    5. Nick Scott,
    6. Joseph S. Doyle,
    7. David P. Wilson,
    8. Amanda Wade,
    9. Margaret E. Hellard
    10. [Purchase PDF](http://www.sciencedirect.com/science/article/pii/S0955395917302086)
36. [Clinical effects of viral relapse after interferon plus ribavirin in patients co-infected with human immunodeficiency virus and hepatitis C virus](http://www.sciencedirect.com/science/article/pii/S0168827813000895)
    1. Original research article
    2. Journal of Hepatology,
    3. Volume 58, Issue 6,
    4. June 2013,
    5. Pages 1104-1112
    6. Juan Berenguer,
    7. Julio Alvarez-Pellicer,
    8. Ana Carrero,
    9. Miguel A. Von Wichmann,
    10. The GESIDA HIV/HCV Cohort Study Group
    11. [Purchase PDF](http://www.sciencedirect.com/science/article/pii/S0168827813000895)
37. [Performance and utility of transient elastography and noninvasive markers of liver fibrosis in primary biliary cirrhosis](http://www.sciencedirect.com/science/article/pii/S1590865811002258)
    1. Original research article
    2. Digestive and Liver Disease,
    3. Volume 43, Issue 11,
    4. November 2011,
    5. Pages 887-892
    6. Annarosa Floreani,
    7. Nora Cazzagon,
    8. Diego Martines,
    9. Luisa Cavalletto,
    10. Liliana Chemello
    11. [Purchase PDF](http://www.sciencedirect.com/science/article/pii/S1590865811002258)
38. [Non-invasive diagnosis of liver fibrosis in the transplant setting](http://www.sciencedirect.com/science/article/pii/S1594580411600211)
    1. Original research article
    2. Digestive and Liver Disease Supplements,
    3. Volume 5, Issue 1,
    4. December 2011,
    5. Pages 23-25
    6. Gonzalo Crespo,
    7. Zoe Mariño
    8. [Purchase PDF](http://www.sciencedirect.com/science/article/pii/S1594580411600211)
39. [Noninvasive evaluation of hepatic fibrosis using acoustic radiation force-based shear stiffness in patients with nonalcoholic fatty liver disease](http://www.sciencedirect.com/science/article/pii/S0168827811000079)
    1. Original research article
    2. Journal of Hepatology,
    3. Volume 55, Issue 3,
    4. September 2011,
    5. Pages 666-672
    6. Mark L. Palmeri,
    7. Michael H. Wang,
    8. Ned C. Rouze,
    9. Manal F. Abdelmalek,
    10. Kathryn R. Nightingale
    11. [Purchase PDF](http://www.sciencedirect.com/science/article/pii/S0168827811000079)
40. [Comparative clinical characteristics and natural history of three variants of sclerosing cholangitis: IgG4-related SC, PSC/AIH and PSC alone](http://www.sciencedirect.com/science/article/pii/S1568997217301428)
    1. Original research article
    2. Autoimmunity Reviews,
    3. Volume 16, Issue 8,
    4. August 2017,
    5. Pages 875-882
    6. Min Lian,
    7. Bo Li,
    8. Xiao Xiao,
    9. Yue Yang,
    10. Xiong Ma
    11. [Purchase PDF](http://www.sciencedirect.com/science/article/pii/S1568997217301428)
41. [A suggested algorithm for using serum biomarkers for the diagnosis of liver fibrosis in chronic hepatitis C infection](http://www.sciencedirect.com/science/article/pii/S168719791000119X)
    1. Original research article
    2. Arab Journal of Gastroenterology,
    3. Volume 11, Issue 4,
    4. December 2010,
    5. Pages 206-211
    6. Madiha M. El-Attar,
    7. Hebat-Allah G. Rashed,
    8. Eman M. Sewify,
    9. Howayda E. Hassan
    10. [Purchase PDF](http://www.sciencedirect.com/science/article/pii/S168719791000119X)
42. [Reproducibility of blood tests of liver fibrosis in clinical practice](http://www.sciencedirect.com/science/article/pii/S0009912007003451)
    1. Original research article
    2. Clinical Biochemistry,
    3. Volume 41, Issues 1–2,
    4. January 2008,
    5. Pages 10-18
    6. Paul Calès,
    7. Pascal Veillon,
    8. Anselme Konaté,
    9. Elisabeth Mathieu,
    10. Françoise Lunel-Fabiani
    11. [Purchase PDF](http://www.sciencedirect.com/science/article/pii/S0009912007003451)
43. [Interferon lambda-3 is not associated with clinical outcome in patients with HCV-induced compensated cirrhosis: A long-term cohort study](http://www.sciencedirect.com/science/article/pii/S016635421400312X)
    1. Original research article
    2. Antiviral Research,
    3. Volume 113,
    4. January 2015,
    5. Pages 27-32
    6. Savino Bruno,
    7. Alex J. Thompson,
    8. Rosina Critelli,
    9. Andrea Crosignani,
    10. Erica Villa
    11. [Purchase PDF](http://www.sciencedirect.com/science/article/pii/S016635421400312X)
44. [Factors Determining Bone Mineral Density in Patients with Biliary Atresia after a Successful Kasai Operation](http://www.sciencedirect.com/science/article/pii/S1875957216300687)
    1. Open access,
    2. Original research article
    3. Pediatrics & Neonatology,
    4. Volume 58, Issue 2,
    5. April 2017,
    6. Pages 128-134
    7. Ming-Huei Chen,
    8. Jiaan-Der Wang,
    9. Chia-Man Chou,
    10. Chieh-Chung Lin
    11. [Download PDF (380 KB)](http://www.sciencedirect.com/science/article/pii/S1875957216300687/pdfft?md5=576f83a2446d80aa3494442551ec318e&pid=1-s2.0-S1875957216300687-main.pdf)
45. [Assessing routine and serum markers of liver fibrosis in CHB patients using parallel and serial interpretation](http://www.sciencedirect.com/science/article/pii/S0009912007000744)
    1. Original research article
    2. Clinical Biochemistry,
    3. Volume 40, Issue 8,
    4. May 2007,
    5. Pages 562-566
    6. Liu Hongbo,
    7. Lv. Xiaohui,
    8. Kong Hong,
    9. Wang Wei,
    10. Zhang Yong
    11. [Purchase PDF](http://www.sciencedirect.com/science/article/pii/S0009912007000744)
46. [Biliary atresia: clinical aspects](http://www.sciencedirect.com/science/article/pii/S1055858612000352)
    1. Original research article
    2. Seminars in Pediatric Surgery,
    3. Volume 21, Issue 3,
    4. August 2012,
    5. Pages 175-184
    6. Mark Davenport
    7. [Purchase PDF](http://www.sciencedirect.com/science/article/pii/S1055858612000352)
47. [HCV mono-infected and HIV/HCV co-infected individuals treated with direct-acting antivirals: to what extent do they differ?](http://www.sciencedirect.com/science/article/pii/S1201971217301790)
    1. Open access,
    2. Original research article
    3. International Journal of Infectious Diseases,
    4. Volume 62,
    5. September 2017,
    6. Pages 64-71
    7. Giuseppe Bruno,
    8. Annalisa Saracino,
    9. Luigia Scudeller,
    10. Claudia Fabrizio,
    11. Gioacchino Angarano
    12. [Download PDF (632 KB)](http://www.sciencedirect.com/science/article/pii/S1201971217301790/pdfft?md5=1abc0df8b5b8e29a8e88dd3a3aea5e11&pid=1-s2.0-S1201971217301790-main.pdf)
48. [Infection With Hepatitis C Virus Genotype 3 Is an Independent Risk Factor for End-Stage Liver Disease, Hepatocellular Carcinoma, and Liver-Related Death](http://www.sciencedirect.com/science/article/pii/S1542356516309296)
    1. Original research article
    2. Clinical Gastroenterology and Hepatology,
    3. Volume 15, Issue 3,
    4. March 2017,
    5. Pages 431-437.e2
    6. Brian J. McMahon,
    7. Dana Bruden,
    8. Lisa Townshend-Bulson,
    9. Brenna Simons,
    10. Prabhu Gounder
    11. [Purchase PDF](http://www.sciencedirect.com/science/article/pii/S1542356516309296)
49. [Serum Wisteria floribunda agglutinin-positive Mac-2-binding protein expression predicts disease severity in chronic hepatitis C patients](http://www.sciencedirect.com/science/article/pii/S1607551X17300670)
    1. Open access,
    2. Original research article
    3. The Kaohsiung Journal of Medical Sciences,
    4. Volume 33, Issue 8,
    5. August 2017,
    6. Pages 394-399
    7. Ching-I Huang,
    8. Chung-Feng Huang,
    9. Ming-Lun Yeh,
    10. Yi-Hung Lin,
    11. Wan-Long Chuang
    12. [Download PDF (495 KB)](http://www.sciencedirect.com/science/article/pii/S1607551X17300670/pdfft?md5=978476b240f48b4efe7f8d3a59b744a0&pid=1-s2.0-S1607551X17300670-main.pdf)
50. [Use of Analytic Morphomics of Liver, Spleen, and Body Composition to Identify Patients at Risk for Cirrhosis](http://www.sciencedirect.com/science/article/pii/S1542356514010878)
    1. Original research article
    2. Clinical Gastroenterology and Hepatology,
    3. Volume 13, Issue 2,
    4. February 2015,
    5. Pages 360-368.e5
    6. Venkat Krishnamurthy,
    7. Peng Zhang,
    8. Sampath Ethiraj,
    9. Binu Enchakalody,
    10. Grace L. Su
    11. [Purchase PDF](http://www.sciencedirect.com/science/article/pii/S1542356514010878)
51. [Baseline Values and Changes in Liver Stiffness Measured by Transient Elastography Are Associated With Severity of Fibrosis and Outcomes of Patients With Primary Sclerosing Cholangitis](http://www.sciencedirect.com/science/article/pii/S0016508513018441)
    1. Original research article
    2. Gastroenterology,
    3. Volume 146, Issue 4,
    4. April 2014,
    5. Pages 970-979.e6
    6. Christophe Corpechot,
    7. Farid Gaouar,
    8. Ahmed El Naggar,
    9. Astrid Kemgang,
    10. Olivier Chazouillères
    11. [Purchase PDF](http://www.sciencedirect.com/science/article/pii/S0016508513018441)
52. [A Randomized Trial of Silymarin for the Treatment of Nonalcoholic Steatohepatitis](http://www.sciencedirect.com/science/article/pii/S1542356517304597)
    1. Original research article
    2. Clinical Gastroenterology and Hepatology,
    3. In press, corrected proof,
    4. Available online 15 April 2017
    5. Wah-Kheong Chan,
    6. Nik Raihan Nik Mustapha,
    7. Sanjiv Mahadeva
    8. [Purchase PDF](http://www.sciencedirect.com/science/article/pii/S1542356517304597)
53. [Early changes in dynamic biomarkers of liver fibrosis in hepatitis C virus-infected patients treated with sofosbuvir](http://www.sciencedirect.com/science/article/pii/S159086581500626X)
    1. Open access,
    2. Original research article
    3. Digestive and Liver Disease,
    4. Volume 48, Issue 3,
    5. March 2016,
    6. Pages 291-297
    7. Sebastian Bernuth,
    8. Eray Yagmur,
    9. Detlef Schuppan,
    10. Martin F. Sprinzl,
    11. Tim Zimmermann
    12. [Download PDF (794 KB)](http://www.sciencedirect.com/science/article/pii/S159086581500626X/pdfft?md5=1137242a7942ae56c781a9b9129f2cd9&pid=1-s2.0-S159086581500626X-main.pdf)
54. [Hepatitis E Virus Does Not Contribute to Hepatic Decompensation Among Patients With Advanced Chronic Hepatitis C](http://www.sciencedirect.com/science/article/pii/S1542356516000550)
    1. Original research article
    2. Clinical Gastroenterology and Hepatology,
    3. Volume 14, Issue 6,
    4. June 2016,
    5. Pages 896-902
    6. Niharika Samala,
    7. Elizabeth C. Wright,
    8. A. Gretchen Buckler,
    9. Vanessa Vargas,
    10. Marc G. Ghany
    11. [Purchase PDF](http://www.sciencedirect.com/science/article/pii/S1542356516000550)
55. [The utility of NAFLD fibrosis score for prediction of mortality among patients with nonalcoholic fatty liverdisease: A systematic review and meta-analysis of cohort study](http://www.sciencedirect.com/science/article/pii/S221074011730089X)
    1. Original research article
    2. Clinics and Research in Hepatology and Gastroenterology,
    3. In press, corrected proof,
    4. Available online 15 July 2017
    5. Veeravich Jaruvongvanich,
    6. Karn Wijarnpreecha,
    7. Patompong Ungprasert
    8. [Purchase PDF](http://www.sciencedirect.com/science/article/pii/S221074011730089X)
56. [La transplantation hépatique chez le nourrisson et l'enfant. À propos de 40 observations (mars 1991–mars 1997)](http://www.sciencedirect.com/science/article/pii/S0929693X00888314)
    1. Original research article
    2. Archives de Pédiatrie,
    3. Volume 7, Issue 4,
    4. April 2000,
    5. Pages 369-376
    6. A. Lachaux,
    7. B. Eid,
    8. D. Stamm,
    9. Y. Gillet,
    10. O. Boulot
    11. [Purchase PDF](http://www.sciencedirect.com/science/article/pii/S0929693X00888314)
57. [Clinical characteristics and outcome of hepatocellular carcinoma in alcohol related and cryptogenic cirrhosis: a prospective study](http://www.sciencedirect.com/science/article/pii/S1499387215603435)
    1. Original research article
    2. Hepatobiliary & Pancreatic Diseases International,
    3. Volume 14, Issue 4,
    4. 15 August 2015,
    5. Pages 401-405
    6. Rohan C Siriwardana,
    7. Maduni A Niriella,
    8. Anuradha S Dassanayake,
    9. Chandika Liyanage,
    10. Hithanadura J de Silva
    11. [Purchase PDF](http://www.sciencedirect.com/science/article/pii/S1499387215603435)
58. [Novel serum and bile protein markers predict primary sclerosing cholangitis disease severity and prognosis](http://www.sciencedirect.com/science/article/pii/S0168827817300521)
    1. Original research article
    2. Journal of Hepatology,
    3. Volume 66, Issue 6,
    4. June 2017,
    5. Pages 1214-1222
    6. Mette Vesterhus,
    7. Anders Holm,
    8. Johannes Roksund Hov,
    9. Ståle Nygård,
    10. Fridtjof Lund-Johansen
    11. [Purchase PDF](http://www.sciencedirect.com/science/article/pii/S0168827817300521)
59. [Clinical characteristics of hepatocellular carcinoma in Spain. Comparison with the 2008–2009 period and analysis of the causes of diagnosis out of screening programs. Analysis of 686 cases in 73 centers](http://www.sciencedirect.com/science/article/pii/S2387020617304138)
    1. Original research article
    2. Medicina Clínica (English Edition),
    3. Volume 149, Issue 2,
    4. 21 July 2017,
    5. Pages 61-71
    6. Carlos Rodríguez de Lope,
    7. María Reig,
    8. Ana Matilla,
    9. María Teresa Ferrer,
    10. on behalf of the Liver Cancer Study Group (GECH)
    11. [Purchase PDF](http://www.sciencedirect.com/science/article/pii/S2387020617304138)
60. [Limited Fibrosis Progression but Significant Mortality in Patients Ineligible for Interferon-Based Hepatitis C Therapy](http://www.sciencedirect.com/science/article/pii/S0973688316300032)
    1. Original research article
    2. Journal of Clinical and Experimental Hepatology,
    3. Volume 6, Issue 2,
    4. June 2016,
    5. Pages 100-108
    6. Manhal Izzy,
    7. Ghalib Jibara,
    8. Aws Aljanabi,
    9. Mustafa Alani,
    10. John F. Reinus
    11. [Purchase PDF](http://www.sciencedirect.com/science/article/pii/S0973688316300032)
61. [A pilot study of ultrasound elastography as a non-invasive method to monitor liver disease in children with short bowel syndrome](http://www.sciencedirect.com/science/article/pii/S0022346817301653)
    1. Original research article
    2. Journal of Pediatric Surgery,
    3. Volume 52, Issue 6,
    4. June 2017,
    5. Pages 962-965
    6. Daniel Lodwick,
    7. Molly Dienhart,
    8. Jennifer N. Cooper,
    9. Bonita Fung,
    10. Peter C. Minneci
    11. [Purchase PDF](http://www.sciencedirect.com/science/article/pii/S0022346817301653)
62. [Cytomegalovirus-associated biliary atresia: An aetiological and prognostic subgroup](http://www.sciencedirect.com/science/article/pii/S0022346815001633)
    1. Original research article
    2. Journal of Pediatric Surgery,
    3. Volume 50, Issue 10,
    4. October 2015,
    5. Pages 1739-1745
    6. Augusto Zani,
    7. Alberto Quaglia,
    8. Nedim Hadzić,
    9. Mark Zuckerman,
    10. Mark Davenport
    11. [Purchase PDF](http://www.sciencedirect.com/science/article/pii/S0022346815001633)
63. [Online combination algorithm for non-invasive assessment of chronic hepatitis B related liver fibrosis and cirrhosis in resource-limited settings](http://www.sciencedirect.com/science/article/pii/S0953620515002319)
    1. Original research article
    2. European Journal of Internal Medicine,
    3. Volume 26, Issue 8,
    4. October 2015,
    5. Pages 628-634
    6. Nermin N. Salkic,
    7. Elmir Cickusic,
    8. Predrag Jovanovic,
    9. Mirela Basic Denjagic,
    10. Sead Ahmetagic
    11. [Purchase PDF](http://www.sciencedirect.com/science/article/pii/S0953620515002319)
64. [Hepatocellular Carcinoma in the Absence of Cirrhosis in United States Veterans Is Associated With Nonalcoholic Fatty Liver Disease](http://www.sciencedirect.com/science/article/pii/S1542356515009787)
    1. Original research article
    2. Clinical Gastroenterology and Hepatology,
    3. Volume 14, Issue 1,
    4. January 2016,
    5. Pages 124-131.e1
    6. Sahil Mittal,
    7. Hashem B. El-Serag,
    8. Yvonne H. Sada,
    9. Fasiha Kanwal,
    10. Jessica A. Davila
    11. [Purchase PDF](http://www.sciencedirect.com/science/article/pii/S1542356515009787)
65. [Soluble urokinase plasminogen activator receptor levels are associated with severity of fibrosis in nonalcoholic fatty liver disease](http://www.sciencedirect.com/science/article/pii/S193152441400334X)
    1. Original research article
    2. Translational Research,
    3. Volume 165, Issue 6,
    4. June 2015,
    5. Pages 658-666
    6. Christopher Sjöwall,
    7. Klara Martinsson,
    8. Kristina Cardell,
    9. Mattias Ekstedt,
    10. Stergios Kechagias
    11. [Purchase PDF](http://www.sciencedirect.com/science/article/pii/S193152441400334X)
66. [Spleen stiffness measurement can predict clinical complications in compensated HCV-related cirrhosis: A prospective study](http://www.sciencedirect.com/science/article/pii/S0168827814001329)
    1. Original research article
    2. Journal of Hepatology,
    3. Volume 60, Issue 6,
    4. June 2014,
    5. Pages 1158-1164
    6. Antonio Colecchia,
    7. Agostino Colli,
    8. Giovanni Casazza,
    9. Daniele Mandolesi,
    10. Davide Festi
    11. [Purchase PDF](http://www.sciencedirect.com/science/article/pii/S0168827814001329)
67. [Long-term native liver fibrosis in biliary atresia: Development of a novel scoring system using histology and standard liver tests](http://www.sciencedirect.com/science/article/pii/S0168827814001044)
    1. Original research article
    2. Journal of Hepatology,
    3. Volume 60, Issue 6,
    4. June 2014,
    5. Pages 1242-1248
    6. Hirofumi Tomita,
    7. Yohei Masugi,
    8. Ken Hoshino,
    9. Yasushi Fuchimoto,
    10. Tatsuo Kuroda
    11. [Purchase PDF](http://www.sciencedirect.com/science/article/pii/S0168827814001044)
68. [Invasive and non-invasive methods for the assessment of fibrosis and disease progression in chronic liverdisease](http://www.sciencedirect.com/science/article/pii/S1521691811000291)
    1. Original research article
    2. Best Practice & Research Clinical Gastroenterology,
    3. Volume 25, Issue 2,
    4. April 2011,
    5. Pages 291-303
    6. Laurent Castera
    7. [Purchase PDF](http://www.sciencedirect.com/science/article/pii/S1521691811000291)
69. [Pain-related anxiety mediates the relationship between depressive symptoms and pain interference in veterans with hepatitis C](http://www.sciencedirect.com/science/article/pii/S0163834315001589)
    1. Original research article
    2. General Hospital Psychiatry,
    3. Volume 37, Issue 6,
    4. November–December 2015,
    5. Pages 533-537
    6. Melissa H. Adams,
    7. Travis I. Lovejoy,
    8. Dennis C. Turk,
    9. Steven K. Dobscha,
    10. Benjamin J. Morasco
    11. [Purchase PDF](http://www.sciencedirect.com/science/article/pii/S0163834315001589)
70. [High HCV cure rates for people who use drugs treated with direct acting antiviral therapy at an urban primary care clinic](http://www.sciencedirect.com/science/article/pii/S0955395917302359)
    1. Original research article
    2. International Journal of Drug Policy,
    3. In press, corrected proof,
    4. Available online 12 August 2017
    5. Brianna L. Norton,
    6. Julia Fleming,
    7. Marcus A. Bachhuber,
    8. Meredith Steinman,
    9. Alain H. Litwin
    10. [Purchase PDF](http://www.sciencedirect.com/science/article/pii/S0955395917302359)
71. [Tratamiento del paciente con hepatitis crónica por el virus de la hepatitis B](http://www.sciencedirect.com/science/article/pii/S0213005X08765200)
    1. Original research article
    2. Enfermedades Infecciosas y Microbiología Clínica,
    3. Volume 26, Supplement 7,
    4. May 2008,
    5. Pages 56-65
    6. Manuel Rodríguez,
    7. María Luisa González-Diéguez
    8. [Purchase PDF](http://www.sciencedirect.com/science/article/pii/S0213005X08765200)
72. [Fibrosis in Hepatitis C Patients Predicts Complications After Elective Total Joint Arthroplasty](http://www.sciencedirect.com/science/article/pii/S0883540313002659)
    1. Original research article
    2. The Journal of Arthroplasty,
    3. Volume 29, Issue 1,
    4. January 2014,
    5. Pages 7-10
    6. Fabio Orozco,
    7. Zachary D. Post,
    8. Omkar Baxi,
    9. Adam Miller,
    10. Alvin Ong
    11. [Purchase PDF](http://www.sciencedirect.com/science/article/pii/S0883540313002659)
73. [Primary Biliary Cholangitis: advances in management and treatment of the disease](http://www.sciencedirect.com/science/article/pii/S1590865817308575)
    1. Open access,
    2. Original research article
    3. Digestive and Liver Disease,
    4. Volume 49, Issue 8,
    5. August 2017,
    6. Pages 841-846
    7. Pietro Invernizzi,
    8. Annarosa Floreani,
    9. Marco Carbone,
    10. Marco Marzioni,
    11. Domenico Alvaro
    12. [Download PDF (559 KB)](http://www.sciencedirect.com/science/article/pii/S1590865817308575/pdfft?md5=d8b851fe4ec6231fd602d957c94499d9&pid=1-s2.0-S1590865817308575-main.pdf)
74. [Non-invasive assessment of hepatic fibrosis in a series of patients with Wilson's Disease](http://www.sciencedirect.com/science/article/pii/S1590865811004750)
    1. Original research article
    2. Digestive and Liver Disease,
    3. Volume 44, Issue 6,
    4. June 2012,
    5. Pages 487-491
    6. Margherita Sini,
    7. Orazio Sorbello,
    8. Alberto Civolani,
    9. Mauro Liggi,
    10. Luigi Demelia
    11. [Purchase PDF](http://www.sciencedirect.com/science/article/pii/S1590865811004750)
75. [Antiviral Therapy for Chronic Hepatitis B Virus Infection and Development of Hepatocellular Carcinoma in a US Population](http://www.sciencedirect.com/science/article/pii/S1542356513015115)
    1. Original research article
    2. Clinical Gastroenterology and Hepatology,
    3. Volume 12, Issue 5,
    4. May 2014,
    5. Pages 885-893
    6. Stuart C. Gordon,
    7. Lois E. Lamerato,
    8. Loralee B. Rupp,
    9. Jia Li,
    10. CHeCS Investigators
    11. [Purchase PDF](http://www.sciencedirect.com/science/article/pii/S1542356513015115)
76. [Peripheral blood mononuclear cells microRNA predicts treatment outcome of hepatitis C virus genotype 1 infection](http://www.sciencedirect.com/science/article/pii/S0166354214000771)
    1. Original research article
    2. Antiviral Research,
    3. Volume 105,
    4. May 2014,
    5. Pages 135-142
    6. Edward Hsi,
    7. Chung-Feng Huang,
    8. Chia-Yen Dai,
    9. Suh-Hang Hank Juo,
    10. Ming-Lung Yu
    11. [Purchase PDF](http://www.sciencedirect.com/science/article/pii/S0166354214000771)
77. [Correlation of FIBROSpect II With Histologic and Morphometric Evaluation of Liver Fibrosis in Chronic Hepatitis C](http://www.sciencedirect.com/science/article/pii/S1542356507011056)
    1. Original research article
    2. Clinical Gastroenterology and Hepatology,
    3. Volume 6, Issue 2,
    4. February 2008,
    5. Pages 242-247
    6. Keyur Patel,
    7. David R. Nelson,
    8. Don C. Rockey,
    9. Nezam H. Afdhal,
    10. John G. McHutchison
    11. [Purchase PDF](http://www.sciencedirect.com/science/article/pii/S1542356507011056)
78. [Long-term safety and efficacy of microRNA-targeted therapy in chronic hepatitis C patients](http://www.sciencedirect.com/science/article/pii/S0166354214002526)
    1. Original research article
    2. Antiviral Research,
    3. Volume 111,
    4. November 2014,
    5. Pages 53-59
    6. Meike H. van der Ree,
    7. Adriaan J. van der Meer,
    8. Joep de Bruijne,
    9. Raoel Maan,
    10. Hendrik W. Reesink
    11. [Purchase PDF](http://www.sciencedirect.com/science/article/pii/S0166354214002526)
79. [Safety and Efficacy of Autologous Bone Marrow Stem Cell Transplantation Through Hepatic Artery for the Treatment of Chronic Liver Failure: A Preliminary Study](http://www.sciencedirect.com/science/article/pii/S0041134508003497)
    1. Original research article
    2. Transplantation Proceedings,
    3. Volume 40, Issue 4,
    4. May 2008,
    5. Pages 1140-1144
    6. A.A. Khan,
    7. N. Parveen,
    8. V.S. Mahaboob,
    9. A. Rajendraprasad,
    10. C.M. Habibullah
    11. [Purchase PDF](http://www.sciencedirect.com/science/article/pii/S0041134508003497)
80. [Le suivi biologique avant et après transplantation hépatique](http://www.sciencedirect.com/science/article/pii/S1773035X06805022)
    1. Original research article
    2. Revue Francophone des Laboratoires,
    3. Volume 2006, Issue 387,
    4. December 2006,
    5. Pages 79-86
    6. Yvon Calmus,
    7. Filoména Conti,
    8. Michel Vaubourdolle
    9. [Purchase PDF](http://www.sciencedirect.com/science/article/pii/S1773035X06805022)
81. [Non-invasive methods can predict oesophageal varices in patients with biliary atresia after a Kasai procedure](http://www.sciencedirect.com/science/article/pii/S1590865811001460)
    1. Original research article
    2. Digestive and Liver Disease,
    3. Volume 43, Issue 8,
    4. August 2011,
    5. Pages 659-663
    6. Antonio Colecchia,
    7. Anna Rita Di Biase,
    8. Eleonora Scaioli,
    9. Barbara Predieri,
    10. Davide Festi
    11. [Purchase PDF](http://www.sciencedirect.com/science/article/pii/S1590865811001460)
82. [Efficacy and safety study of cenicriviroc for the treatment of non-alcoholic steatohepatitis in adult subjects with liver fibrosis: CENTAUR Phase 2b study design](http://www.sciencedirect.com/science/article/pii/S155171441630026X)
    1. Open access,
    2. Original research article
    3. Contemporary Clinical Trials,
    4. Volume 47,
    5. March 2016,
    6. Pages 356-365
    7. Scott Friedman,
    8. Arun Sanyal,
    9. Zachary Goodman,
    10. Eric Lefebvre,
    11. Vlad Ratziu
    12. [Download PDF (864 KB)](http://www.sciencedirect.com/science/article/pii/S155171441630026X/pdfft?md5=3dea94ae0f1dc6ecc9db50385aa2b14b&pid=1-s2.0-S155171441630026X-main.pdf)
83. [Similarities and differences between pediatric and adult nonalcoholic fatty liver disease](http://www.sciencedirect.com/science/article/pii/S0026049516000159)
    1. Original research article
    2. Metabolism,
    3. Volume 65, Issue 8,
    4. August 2016,
    5. Pages 1161-1171
    6. Maricruz Crespo,
    7. Sara Lappe,
    8. Ariel E. Feldstein,
    9. Naim Alkhouri
    10. [Purchase PDF](http://www.sciencedirect.com/science/article/pii/S0026049516000159)
84. [High hepatic and extrahepatic mortality and low treatment uptake in HCV-coinfected persons in the Swiss HIV cohort study between 2001 and 2013](http://www.sciencedirect.com/science/article/pii/S0168827815003025)
    1. Original research article
    2. Journal of Hepatology,
    3. Volume 63, Issue 3,
    4. September 2015,
    5. Pages 573-580
    6. Helen Kovari,
    7. Bruno Ledergerber,
    8. Matthias Cavassini,
    9. Juan Ambrosioni,
    10. Swiss HIV Cohort Study
    11. [Purchase PDF](http://www.sciencedirect.com/science/article/pii/S0168827815003025)
85. [Enfermedades hepáticas](http://www.sciencedirect.com/science/article/pii/S0210570508766378)
    1. Original research article
    2. Gastroenterología y Hepatología,
    3. Volume 31, Supplement 4,
    4. October 2008,
    5. Pages 93-104
    6. José Luis Calleja,
    7. Agustín Albillos,
    8. Rafael Bañares
    9. [Purchase PDF](http://www.sciencedirect.com/science/article/pii/S0210570508766378)
86. [Retention in buprenorphine treatment is associated with improved HCV care outcomes](http://www.sciencedirect.com/science/article/pii/S0740547216301489)
    1. Original research article
    2. Journal of Substance Abuse Treatment,
    3. Volume 75,
    4. April 2017,
    5. Pages 38-42
    6. B.L. Norton,
    7. A. Beitin,
    8. M. Glenn,
    9. J. DeLuca,
    10. C.O. Cunningham
    11. [Purchase PDF](http://www.sciencedirect.com/science/article/pii/S0740547216301489)
87. [Effectiveness of surveillance for hepatocellular carcinoma in clinical practice: A United States cohort](http://www.sciencedirect.com/science/article/pii/S0168827816303956)
    1. Original research article
    2. Journal of Hepatology,
    3. Volume 65, Issue 6,
    4. December 2016,
    5. Pages 1148-1154
    6. Sahil Mittal,
    7. Fasiha Kanwal,
    8. Jun Ying,
    9. Randy Chung,
    10. Hashem B. El-Serag
    11. [Purchase PDF](http://www.sciencedirect.com/science/article/pii/S0168827816303956)
88. [Onsite treatment of HCV infection with direct acting antivirals within an opioid treatment program](http://www.sciencedirect.com/science/article/pii/S074054721630321X)
    1. Original research article
    2. Journal of Substance Abuse Treatment,
    3. Volume 75,
    4. April 2017,
    5. Pages 49-53
    6. Jenna L. Butner,
    7. Neil Gupta,
    8. Chris Fabian,
    9. Susan Henry,
    10. Jeanette M. Tetrault
    11. [Purchase PDF](http://www.sciencedirect.com/science/article/pii/S074054721630321X)
89. [Host Response to Translocated Microbial Products Predicts Outcomes of Patients With HBV or HCV Infection](http://www.sciencedirect.com/science/article/pii/S0016508511009139)
    1. Original research article
    2. Gastroenterology,
    3. Volume 141, Issue 4,
    4. October 2011,
    5. Pages 1220-1230.e3
    6. Netanya G. Sandler,
    7. Christopher Koh,
    8. Annelys Roque,
    9. Jason L. Eccleston,
    10. Daniel C. Douek
    11. [Purchase PDF](http://www.sciencedirect.com/science/article/pii/S0016508511009139)
90. [Host interleukin-28B genetic variants versus viral kinetics in determining responses to standard-of-care for Asians with hepatitis C genotype 1](http://www.sciencedirect.com/science/article/pii/S0166354211005213)
    1. Original research article
    2. Antiviral Research,
    3. Volume 93, Issue 2,
    4. February 2012,
    5. Pages 239-244
    6. Chung-Feng Huang,
    7. Ming-Lun Yeh,
    8. Jee-Fu Huang,
    9. Jeng-Fu Yang,
    10. Ming-Lung Yu
    11. [Purchase PDF](http://www.sciencedirect.com/science/article/pii/S0166354211005213)
91. [Th-17 cells infiltrate the liver in human biliary atresia and are related to surgical outcome](http://www.sciencedirect.com/science/article/pii/S0022346815000858)
    1. Original research article
    2. Journal of Pediatric Surgery,
    3. Volume 50, Issue 8,
    4. August 2015,
    5. Pages 1297-1303
    6. Richard Hill,
    7. Alberto Quaglia,
    8. Munther Hussain,
    9. Nedim Hadzic,
    10. Mark Davenport
    11. [Purchase PDF](http://www.sciencedirect.com/science/article/pii/S0022346815000858)
92. [Predictors of pain intensity and pain functioning in patients with the hepatitis C virus](http://www.sciencedirect.com/science/article/pii/S0163834310000605)
    1. Original research article
    2. General Hospital Psychiatry,
    3. Volume 32, Issue 4,
    4. July–August 2010,
    5. Pages 413-418
    6. Benjamin J. Morasco,
    7. Marilyn Huckans,
    8. Jennifer M. Loftis,
    9. Jonathan Woodhouse,
    10. Peter Hauser
    11. [Purchase PDF](http://www.sciencedirect.com/science/article/pii/S0163834310000605)
93. [Characteristics of Congenital Hepatic Fibrosis in a Large Cohort of Patients With Autosomal Recessive Polycystic Kidney Disease](http://www.sciencedirect.com/science/article/pii/S0016508512014576)
    1. Original research article
    2. Gastroenterology,
    3. Volume 144, Issue 1,
    4. January 2013,
    5. Pages 112-121.e2
    6. Meral Gunay–Aygun,
    7. Esperanza Font–Montgomery,
    8. Linda Lukose,
    9. Maya Tuchman Gerstein,
    10. Theo Heller
    11. [Purchase PDF](http://www.sciencedirect.com/science/article/pii/S0016508512014576)
94. [Marcadores serológicos de fibrosis](http://www.sciencedirect.com/science/article/pii/S0210570512700444)
    1. Original research article
    2. Gastroenterología y Hepatología,
    3. Volume 35, Supplement 2,
    4. December 2012,
    5. Pages 10-16
    6. Guillermo Fernández-Varo
    7. [Purchase PDF](http://www.sciencedirect.com/science/article/pii/S0210570512700444)
95. [Virological characteristics of occult hepatitis B virus in a North American cohort of human immunodeficiency virus type 1-positive patients on dual active anti-HBV/HIV therapy](http://www.sciencedirect.com/science/article/pii/S1386653214001541)
    1. Original research article
    2. Journal of Clinical Virology,
    3. Volume 60, Issue 4,
    4. August 2014,
    5. Pages 347-353
    6. Carla S. Coffin,
    7. Patricia M. Mulrooney-Cousins,
    8. Carla Osiowy,
    9. Frank van der Meer,
    10. M. John Gill
    11. [Purchase PDF](http://www.sciencedirect.com/science/article/pii/S1386653214001541)
96. [Hepatitis por el virus C](http://www.sciencedirect.com/science/article/pii/S0304541216300294)
    1. Original research article
    2. Medicine - Programa de Formación Médica Continuada Acreditado,
    3. Volume 12, Issue 9,
    4. April 2016,
    5. Pages 484-493
    6. J. Cabezas,
    7. S. Llerena,
    8. S. Menéndez,
    9. C. Alonso Martín,
    10. J. Crespo García
    11. [Purchase PDF](http://www.sciencedirect.com/science/article/pii/S0304541216300294)
97. [Impact of Liver Disease After the Fontan Operation](http://www.sciencedirect.com/science/article/pii/S0002914914020207)
    1. Original research article
    2. The American Journal of Cardiology,
    3. Volume 115, Issue 2,
    4. 15 January 2015,
    5. Pages 249-252
    6. Ian Lindsay,
    7. Joy Johnson,
    8. Melanie D. Everitt,
    9. James Hoffman,
    10. Anji T. Yetman
    11. [Purchase PDF](http://www.sciencedirect.com/science/article/pii/S0002914914020207)
98. [Diagnostic value of magnetic resonance elastography for detecting and staging of hepatic fibrosis: A meta-analysis](http://www.sciencedirect.com/science/article/pii/S0009926014004309)
    1. Original research article
    2. Clinical Radiology,
    3. Volume 69, Issue 12,
    4. December 2014,
    5. Pages e545-e552
    6. L.-N. Su,
    7. S.-L. Guo,
    8. B.-X. Li,
    9. P. Yang
    10. [Purchase PDF](http://www.sciencedirect.com/science/article/pii/S0009926014004309)
99. [25](http://www.sciencedirect.com/search?qs=%22Liver%20transplantation%22%20and%20APRI&show=25&sortBy=relevance&articleTypes=OR&navigation=true)
100. [50](http://www.sciencedirect.com/search?qs=%22Liver%20transplantation%22%20and%20APRI&show=50&sortBy=relevance&articleTypes=OR&navigation=true)
101. 100
102. Page 1 of 2
103. [next](http://www.sciencedirect.com/search?qs=%22Liver%20transplantation%22%20and%20APRI&show=100&sortBy=relevance&articleTypes=OR&navigation=true&offset=100)

[Feedback](http://www.sciencedirect.com/search?qs=%22Liver%20transplantation%22%20and%20APRI&show=100&sortBy=relevance&articleTypes=OR&navigation=true&offset=0)

[About ScienceDirect](http://www.elsevier.com/solutions/sciencedirect)[Remote access](http://www.sciencedirect.com/science/activateaccess)[Shopping cart](http://www.sciencedirect.com/science?_ob=ShoppingCartURL&_method=display&_zone=TopNavBar&_origin=srp&md5=e5857d1631c4b1f612897ed029064507)[Contact and support](https://service.elsevier.com/app/contact/supporthub/sciencedirect/)[Terms and conditions](https://www.elsevier.com/legal/elsevier-website-terms-and-conditions)[Privacy policy](http://www.elsevier.com/legal/privacy-policy)

Cookies are used by this site. For more information, visit the [cookies page](https://www.elsevier.com/solutions/sciencedirect/support/cookies).

Copyright © 2017 Elsevier B.V. or its licensors or contributors. ScienceDirect ® is a registered trademark of Elsevier B.V.

[Skip to Main content](http://www.sciencedirect.com/search?qs=%22Liver%20transplantation%22%20and%20APRI&show=100&sortBy=relevance&articleTypes=OR&navigation=true&offset=100#main_content)

- [Journals](http://www.sciencedirect.com/science/journals)
- [Books](http://www.sciencedirect.com/science/bookbshsrw)
- [Register](https://www.sciencedirect.com/user/register?returnURL=http%3A%2F%2Fwww.sciencedirect.com%2Fsearch)
- [Sign in](https://www.sciencedirect.com/user/login?returnURL=http%3A%2F%2Fwww.sciencedirect.com%2Fsearch)

Top of Form


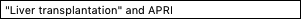


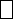


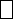


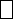


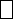


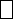


[Advanced search](http://www.sciencedirect.com/science/search)

Bottom of Form

148 results

Refine by:

Top of Form

Years

1.
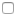
 2017 (17)
2.
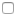
 2016 (18)
3.
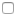
 2015 (19)
4.
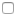
 2014 (19)
5.
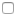
 2013 (9)
6.
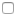
 2012 (12)
7.
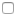
 2011 (12)
8.
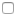
 2010 (13)
9.
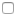
 2009 (9)
10.
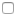
 2008 (12)

Show less

Article type

1.
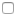
 Original research (148)

Publication title

1.
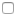
 Journal of Hepatology (24)
2.
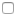
 Clinical Gastroenterology and Hepatology (15)
3.
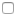
 Gastroenterology (12)
4.
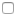
 Journal of Pediatric Surgery (9)
5.
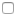
 Digestive and Liver Disease (9)
6.
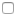
 Antiviral Research (4)
7.
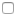
 Gastroenterología y Hepatología (4)
8.
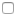
 Clinical Biochemistry (3)
9.
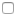
 Best Practice & Research Clinical Gastroenterology (3)
10.
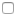
 Enfermedades Infecciosas y Microbiología Clínica (3)

Show less

[Clear all filters](http://www.sciencedirect.com/search?qs=%22Liver%20transplantation%22%20and%20APRI&show=100&sortBy=relevance&navigation=true&offset=100)

Bottom of Form

sorted by *relevance* | [date](http://www.sciencedirect.com/search?qs=%22Liver%20transplantation%22%20and%20APRI&show=100&sortBy=date&articleTypes=OR&navigation=true&offset=100)

1. [Treatment Patterns and Adherence among Patients with Chronic Hepatitis C Virus in a US Managed Care Population](http://www.sciencedirect.com/science/article/pii/S1098301510600848)
   1. Open archive,
   2. Original research article
   3. Value in Health,
   4. Volume 13, Issue 4,
   5. June–July 2010,
   6. Pages 479-486
   7. Debanjali Mitra,
   8. Keith L. Davis,
   9. Cynthia Beam,
   10. Jasmina Medjedovic,
   11. Vinod Rustgi
   12. [Download PDF (90 KB)](http://www.sciencedirect.com/science/article/pii/S1098301510600848/pdfft?md5=3bc1d10222b680e9b6ca2090e8215e97&pid=1-s2.0-S1098301510600848-main.pdf)
2. [Prediction of oesophageal varices in hepatic cirrhosis by simple serum non-invasive markers: Results of a multicenter, large-scale study](http://www.sciencedirect.com/science/article/pii/S0168827810005283)
   1. Original research article
   2. Journal of Hepatology,
   3. Volume 53, Issue 4,
   4. October 2010,
   5. Pages 630-638
   6. Giada Sebastiani,
   7. Diego Tempesta,
   8. Giovanna Fattovich,
   9. Laurent Castera,
   10. Alfredo Alberti
   11. [Purchase PDF](http://www.sciencedirect.com/science/article/pii/S0168827810005283)
3. Want a richer search experience?

Sign in for additional filter options, multiple article downloads, and more.

Top of Form

Sign in

Bottom of Form

1. [The value of gadoxetate disodium-enhanced MR imaging for predicting posthepatectomy liver failure after major hepatic resection: A preliminary study](http://www.sciencedirect.com/science/article/pii/S0720048X11006620)
   1. Original research article
   2. European Journal of Radiology,
   3. Volume 80, Issue 2,
   4. November 2011,
   5. Pages e195-e200
   6. Seung Hyun Cho,
   7. Ung Rae Kang,
   8. Joo Dong Kim,
   9. Young Seok Han,
   10. Dong Lak Choi
   11. [Purchase PDF](http://www.sciencedirect.com/science/article/pii/S0720048X11006620)
2. [Interleukin-28B genetic variants in identification of hepatitis C virus genotype 1 patients responding to 24 weeks peginterferon/ribavirin](http://www.sciencedirect.com/science/article/pii/S016882781100376X)
   1. Original research article
   2. Journal of Hepatology,
   3. Volume 56, Issue 1,
   4. January 2012,
   5. Pages 34-40
   6. Chung-Feng Huang,
   7. Jee-Fu Huang,
   8. Jeng-Fu Yang,
   9. Ming-Yen Hsieh,
   10. Ming-Lung Yu
   11. [Purchase PDF](http://www.sciencedirect.com/science/article/pii/S016882781100376X)
3. [Discordance in fibrosis staging between liver biopsy and transient elastography using the FibroScan XL probe](http://www.sciencedirect.com/science/article/pii/S0168827811007823)
   1. Original research article
   2. Journal of Hepatology,
   3. Volume 56, Issue 3,
   4. March 2012,
   5. Pages 564-570
   6. Robert P. Myers,
   7. Gilles Pomier-Layrargues,
   8. Richard Kirsch,
   9. Aaron Pollett,
   10. Magdy Elkashab
   11. [Purchase PDF](http://www.sciencedirect.com/science/article/pii/S0168827811007823)
4. [Non-invasive evaluation of liver fibrosis using transient elastography](http://www.sciencedirect.com/science/article/pii/S0168827808001232)
   1. Original research article
   2. Journal of Hepatology,
   3. Volume 48, Issue 5,
   4. May 2008,
   5. Pages 835-847
   6. Laurent Castera,
   7. Xavier Forns,
   8. Alfredo Alberti
   9. [Purchase PDF](http://www.sciencedirect.com/science/article/pii/S0168827808001232)
5. [Profound week 4 interferon responsiveness is mandatory for hepatitis C genotype 1 patients with unfavorable IL-28B genotype](http://www.sciencedirect.com/science/article/pii/S1386653212004350)
   1. Original research article
   2. Journal of Clinical Virology,
   3. Volume 56, Issue 4,
   4. April 2013,
   5. Pages 293-298
   6. Chung-Feng Huang,
   7. Ming-Lung Yu,
   8. Jia-Horng Kao,
   9. Tai-Chung Tseng,
   10. Chen-Hua Liu
   11. [Purchase PDF](http://www.sciencedirect.com/science/article/pii/S1386653212004350)
6. [Noninvasive Methods to Assess Liver Disease in Patients With Hepatitis B or C](http://www.sciencedirect.com/science/article/pii/S0016508512002302)
   1. Original research article
   2. Gastroenterology,
   3. Volume 142, Issue 6,
   4. May 2012,
   5. Pages 1293-1302.e4
   6. Laurent Castera
   7. [Purchase PDF](http://www.sciencedirect.com/science/article/pii/S0016508512002302)
7. [Non-invasive diagnosis and monitoring of liver fibrosis and cirrhosis](http://www.sciencedirect.com/science/article/pii/S1521691809000390)
   1. Original research article
   2. Best Practice & Research Clinical Gastroenterology,
   3. Volume 23, Issue 3,
   4. June 2009,
   5. Pages 453-460
   6. Ulrike W. Denzer,
   7. Stefan Lüth
   8. [Purchase PDF](http://www.sciencedirect.com/science/article/pii/S1521691809000390)
8. [Long-term follow-up of hepatitis C infection in a large cohort of patients with inherited bleeding disorders](http://www.sciencedirect.com/science/article/pii/S0168827813006041)
   1. Original research article
   2. Journal of Hepatology,
   3. Volume 60, Issue 1,
   4. January 2014,
   5. Pages 39-45
   6. Dietje Elisabeth Fransen van de Putte,
   7. Michael Makris,
   8. Kathelijn Fischer,
   9. Thynn Thynn Yee,
   10. Eveline Pauline Mauser-Bunschoten
   11. [Purchase PDF](http://www.sciencedirect.com/science/article/pii/S0168827813006041)
9. [Hepatitis B Virus–Specific and Global T-Cell Dysfunction in Chronic Hepatitis B](http://www.sciencedirect.com/science/article/pii/S0016508515017369)
   1. Original research article
   2. Gastroenterology,
   3. Volume 150, Issue 3,
   4. March 2016,
   5. Pages 684-695.e5
   6. Jang-June Park,
   7. David K. Wong,
   8. Abdus S. Wahed,
   9. William M. Lee,
   10. Hepatitis B Research Network
   11. [Purchase PDF](http://www.sciencedirect.com/science/article/pii/S0016508515017369)
10. [Incidence of Hepatocellular Carcinoma and Associated Risk Factors in Hepatitis C-Related Advanced LiverDisease](http://www.sciencedirect.com/science/article/pii/S001650850801679X)
    1. Original research article
    2. Gastroenterology,
    3. Volume 136, Issue 1,
    4. January 2009,
    5. Pages 138-148
    6. Anna S. Lok,
    7. Leonard B. Seeff,
    8. Timothy R. Morgan,
    9. Adrian M. di Bisceglie,
    10. HALT-C Trial Group
    11. [Purchase PDF](http://www.sciencedirect.com/science/article/pii/S001650850801679X)
11. [Early Virologic Responses and Hematologic Safety of Direct-Acting Antiviral Therapies in Veterans With Chronic Hepatitis C](http://www.sciencedirect.com/science/article/pii/S1542356513003856)
    1. Original research article
    2. Clinical Gastroenterology and Hepatology,
    3. Volume 11, Issue 8,
    4. August 2013,
    5. Pages 1021-1027
    6. Pamela S. Belperio,
    7. Elizabeth W. Hwang,
    8. I. Chun Thomas,
    9. Larry A. Mole,
    10. Lisa I. Backus
    11. [Purchase PDF](http://www.sciencedirect.com/science/article/pii/S1542356513003856)
12. [Hepatitis C Virus: A Critical Appraisal of Approaches to Therapy](http://www.sciencedirect.com/science/article/pii/S1542356508011609)
    1. Original research article
    2. Clinical Gastroenterology and Hepatology,
    3. Volume 7, Issue 4,
    4. April 2009,
    5. Pages 397-414
    6. David R. Nelson,
    7. Gary L. Davis,
    8. Ira Jacobson,
    9. Gregory T. Everson,
    10. Nizar Zein
    11. [Purchase PDF](http://www.sciencedirect.com/science/article/pii/S1542356508011609)
13. [Staging chronic hepatitis C in seven categories using fibrosis biomarker (FibroTest™) and transient elastography (FibroScan®)](http://www.sciencedirect.com/science/article/pii/S0168827813008179)
    1. Original research article
    2. Journal of Hepatology,
    3. Volume 60, Issue 4,
    4. April 2014,
    5. Pages 706-714
    6. Thierry Poynard,
    7. Julien Vergniol,
    8. Yen Ngo,
    9. Juliette Foucher,
    10. Bordeaux HCV Study Group
    11. [Purchase PDF](http://www.sciencedirect.com/science/article/pii/S0168827813008179)
14. [Acute kidney injury following hepatectomy for hepatocellular carcinoma: incidence, risk factors and prognostic value](http://www.sciencedirect.com/science/article/pii/S1365182X16316549)
    1. Open archive,
    2. Original research article
    3. HPB,
    4. Volume 18, Issue 6,
    5. June 2016,
    6. Pages 540-548
    7. Chetana Lim,
    8. Etienne Audureau,
    9. Chady Salloum,
    10. Eric Levesque,
    11. Daniel Azoulay
    12. [Download PDF (172 KB)](http://www.sciencedirect.com/science/article/pii/S1365182X16316549/pdfft?md5=557414b6d6143b9fbc597f6c8cd4cb2d&pid=1-s2.0-S1365182X16316549-main.pdf)
15. [Can preoperative diffusion-weighted MRI predict postoperative hepatic insufficiency after curative resection of HBV-related hepatocellular carcinoma? A pilot study](http://www.sciencedirect.com/science/article/pii/S0730725X10000810)
    1. Original research article
    2. Magnetic Resonance Imaging,
    3. Volume 28, Issue 6,
    4. July 2010,
    5. Pages 802-811
    6. Seung Up Kim,
    7. Young Chul Kim,
    8. Ji Soo Choi,
    9. Kyung Sik Kim,
    10. Myeong-Jin Kim
    11. [Purchase PDF](http://www.sciencedirect.com/science/article/pii/S0730725X10000810)
16. [Hepatic fibrosis in paediatric liver disease](http://www.sciencedirect.com/science/article/pii/S2210740112000952)
    1. Original research article
    2. Clinics and Research in Hepatology and Gastroenterology,
    3. Volume 36, Issue 3,
    4. June 2012,
    5. Pages 268-270
    6. Imeke Goldschmidt,
    7. Ulrich Baumann
    8. [Purchase PDF](http://www.sciencedirect.com/science/article/pii/S2210740112000952)
17. [Biologie et cirrhose](http://www.sciencedirect.com/science/article/pii/S1773035X06805009)
    1. Original research article
    2. Revue Francophone des Laboratoires,
    3. Volume 2006, Issue 387,
    4. December 2006,
    5. Pages 65-71
    6. Hélène Voitot
    7. [Purchase PDF](http://www.sciencedirect.com/science/article/pii/S1773035X06805009)
18. [Staging chronic hepatitis B into seven categories, defining inactive carriers and assessing treatment impact using a fibrosis biomarker (FibroTest®) and elastography (FibroScan®)](http://www.sciencedirect.com/science/article/pii/S0168827814004577)
    1. Original research article
    2. Journal of Hepatology,
    3. Volume 61, Issue 5,
    4. November 2014,
    5. Pages 994-1003
    6. Thierry Poynard,
    7. Julien Vergniol,
    8. Yen Ngo,
    9. Juliette Foucher,
    10. FibroFrance Study Group and the Bordeaux HBV Study Group
    11. [Purchase PDF](http://www.sciencedirect.com/science/article/pii/S0168827814004577)
19. [Ledipasvir-sofosbuvir with or without ribavirin to treat patients with HCV genotype 1 infection and cirrhosis non-responsive to previous protease-inhibitor therapy: a randomised, double-blind, phase 2 trial (SIRIUS)](http://www.sciencedirect.com/science/article/pii/S1473309915700502)
    1. Original research article
    2. The Lancet Infectious Diseases,
    3. Volume 15, Issue 4,
    4. April 2015,
    5. Pages 397-404
    6. Marc Bourlière,
    7. Jean-Pierre Bronowicki,
    8. Victor de Ledinghen,
    9. Christophe Hézode,
    10. Stanislas Pol
    11. [Purchase PDF](http://www.sciencedirect.com/science/article/pii/S1473309915700502)
20. [Portal Pressure Predicts Outcome and Safety of Antiviral Therapy in Cirrhotic Patients With Hepatitis C Virus Infection](http://www.sciencedirect.com/science/article/pii/S1542356511002308)
    1. Original research article
    2. Clinical Gastroenterology and Hepatology,
    3. Volume 9, Issue 7,
    4. July 2011,
    5. Pages 602-608.e1
    6. Thomas Reiberger,
    7. Karoline Rutter,
    8. Arnulf Ferlitsch,
    9. Berit Anna Payer,
    10. Markus Peck–Radosavljevic
    11. [Purchase PDF](http://www.sciencedirect.com/science/article/pii/S1542356511002308)
21. [ARFI elastography in patients with chronic autoimmune liver diseases: A preliminary study](http://www.sciencedirect.com/science/article/pii/S1971349512000653)
    1. Original research article
    2. Journal of Ultrasound,
    3. Volume 15, Issue 4,
    4. December 2012,
    5. Pages 226-231
    6. S. Righi,
    7. E. Fiorini,
    8. C. De Molo,
    9. V. Cipriano,
    10. C. Serra
    11. [Purchase PDF](http://www.sciencedirect.com/science/article/pii/S1971349512000653)
22. [Hepatitis delta-associated mortality in HIV/HBV-coinfected patients](http://www.sciencedirect.com/science/article/pii/S0168827816305694)
    1. Original research article
    2. Journal of Hepatology,
    3. Volume 66, Issue 2,
    4. February 2017,
    5. Pages 297-303
    6. Charles Béguelin,
    7. Darius Moradpour,
    8. Roland Sahli,
    9. Franziska Suter-Riniker,
    10. Swiss HIV Cohort Study
    11. [Purchase PDF](http://www.sciencedirect.com/science/article/pii/S0168827816305694)
23. [Magnetic Resonance Imaging More Accurately Classifies Steatosis and Fibrosis in Patients With Nonalcoholic Fatty Liver Disease Than Transient Elastography](http://www.sciencedirect.com/science/article/pii/S0016508515017345)
    1. Open access,
    2. Original research article
    3. Gastroenterology,
    4. Volume 150, Issue 3,
    5. March 2016,
    6. Pages 626-637.e7
    7. Kento Imajo,
    8. Takaomi Kessoku,
    9. Yasushi Honda,
    10. Wataru Tomeno,
    11. Atsushi Nakajima
    12. [Download PDF (1,580 KB)](http://www.sciencedirect.com/science/article/pii/S0016508515017345/pdfft?md5=3127e601c346bb0639a230236cdacf22&pid=1-s2.0-S0016508515017345-main.pdf)
24. [Noninvasive Assessment of Liver Fibrosis and Portal Hypertension With Transient Elastography](http://www.sciencedirect.com/science/article/pii/S0016508507021300)
    1. Original research article
    2. Gastroenterology,
    3. Volume 134, Issue 1,
    4. January 2008,
    5. Pages 8-14
    6. Don C. Rockey
    7. [Purchase PDF](http://www.sciencedirect.com/science/article/pii/S0016508507021300)
25. [Recurrence predictive models for patients with hepatocellular carcinoma after radiofrequency ablation using support vector machines with feature selection methods](http://www.sciencedirect.com/science/article/pii/S016926071400323X)
    1. Original research article
    2. Computer Methods and Programs in Biomedicine,
    3. Volume 117, Issue 3,
    4. December 2014,
    5. Pages 425-434
    6. Ja-Der Liang,
    7. Xiao-Ou Ping,
    8. Yi-Ju Tseng,
    9. Guan-Tarn Huang,
    10. Pei-Ming Yang
    11. [Purchase PDF](http://www.sciencedirect.com/science/article/pii/S016926071400323X)
26. [Changes in liver stiffness using acoustic radiation force impulse imaging in patients with obstructive cholestasis and cholangitis](http://www.sciencedirect.com/science/article/pii/S1590865814002734)
    1. Original research article
    2. Digestive and Liver Disease,
    3. Volume 46, Issue 7,
    4. July 2014,
    5. Pages 625-631
    6. Dina Attia,
    7. Sven Pischke,
    8. Ahmad A. Negm,
    9. Kinan Rifai,
    10. Andrej Potthoff
    11. [Purchase PDF](http://www.sciencedirect.com/science/article/pii/S1590865814002734)
27. [Which are the cut-off values of 2D-Shear Wave Elastography (2D-SWE) liver stiffness measurements predicting different stages of liver fibrosis, considering Transient Elastography (TE) as the reference method?](http://www.sciencedirect.com/science/article/pii/S0720048X13006542)
    1. Original research article
    2. European Journal of Radiology,
    3. Volume 83, Issue 3,
    4. March 2014,
    5. Pages e118-e122
    6. Ioan Sporea,
    7. Simona Bota,
    8. Oana Gradinaru-Taşcău,
    9. Roxana Şirli,
    10. Ana Jurchiş
    11. [Purchase PDF](http://www.sciencedirect.com/science/article/pii/S0720048X13006542)
28. [FibroTest-ActiTest as a non-invasive marker of liver fibrosis](http://www.sciencedirect.com/science/article/pii/S0399832008739915)
    1. Original research article
    2. Gastroentérologie Clinique et Biologique,
    3. Volume 32, Issue 6, Supplement 1,
    4. September 2008,
    5. Pages 22-39
    6. Philippe Halfon,
    7. Mona Munteanu,
    8. Thierry Poynard
    9. [Purchase PDF](http://www.sciencedirect.com/science/article/pii/S0399832008739915)
29. [Does transient elastography (FibroScan®) have a role in decision making in hepatocellular carcinoma?](http://www.sciencedirect.com/science/article/pii/S1365182X15305955)
    1. Open archive,
    2. Original research article
    3. HPB,
    4. Volume 14, Issue 6,
    5. June 2012,
    6. Pages 403-408
    7. Antonio Pesce,
    8. Roberto Scilletta,
    9. Angela Branca,
    10. Luciano Nigro,
    11. Stefano Puleo
    12. [Download PDF (155 KB)](http://www.sciencedirect.com/science/article/pii/S1365182X15305955/pdfft?md5=64dd0187ba44040255452a19247ab2cb&pid=1-s2.0-S1365182X15305955-main.pdf)
30. [Screening for Liver Fibrosis by Using a Noninvasive Biomarker in Patients With Diabetes](http://www.sciencedirect.com/science/article/pii/S1542356508002498)
    1. Original research article
    2. Clinical Gastroenterology and Hepatology,
    3. Volume 6, Issue 7,
    4. July 2008,
    5. Pages 828-831
    6. Sophie Jacqueminet,
    7. Pascal Lebray,
    8. Rachel Morra,
    9. Mona Munteanu,
    10. Thierry Poynard
    11. [Purchase PDF](http://www.sciencedirect.com/science/article/pii/S1542356508002498)
31. [Utilidad del Fibroscan® para evaluar la fibrosis hepática](http://www.sciencedirect.com/science/article/pii/S0210570509003562)
    1. Original research article
    2. Gastroenterología y Hepatología,
    3. Volume 32, Issue 6,
    4. June–July 2009,
    5. Pages 415-423
    6. José A. Carrión
    7. [Purchase PDF](http://www.sciencedirect.com/science/article/pii/S0210570509003562)
32. [Hepatitis C virus genotype 1b as a risk factor for hepatocellular carcinoma development: A meta-analysis](http://www.sciencedirect.com/science/article/pii/S0168827809001573)
    1. Original research article
    2. Journal of Hepatology,
    3. Volume 50, Issue 6,
    4. June 2009,
    5. Pages 1142-1154
    6. Sara Raimondi,
    7. Savino Bruno,
    8. Mario U. Mondelli,
    9. Patrick Maisonneuve
    10. [Purchase PDF](http://www.sciencedirect.com/science/article/pii/S0168827809001573)
33. [FibroScan (Vibration-Controlled Transient Elastography): Where Does It Stand in the United States Practice](http://www.sciencedirect.com/science/article/pii/S1542356514008180)
    1. Original research article
    2. Clinical Gastroenterology and Hepatology,
    3. Volume 13, Issue 1,
    4. January 2015,
    5. Pages 27-36
    6. Elliot B. Tapper,
    7. Laurent Castera,
    8. Nezam H. Afdhal
    9. [Purchase PDF](http://www.sciencedirect.com/science/article/pii/S1542356514008180)
34. [Lack of effect of tumor necrosis factor-alpha -308 G/A polymorphism on severity of liver fibrosis in Tunisian hepatitis C virus (HCV)-infected patients](http://www.sciencedirect.com/science/article/pii/S039983201000120X)
    1. Original research article
    2. Gastroentérologie Clinique et Biologique,
    3. Volume 34, Issues 4–5,
    4. April–May 2010,
    5. Pages 297-304
    6. N. Bouzgarrou,
    7. E. Hassen,
    8. S. Gabbouj,
    9. E. Schvoerer,
    10. L. Chouchane
    11. [Purchase PDF](http://www.sciencedirect.com/science/article/pii/S039983201000120X)
35. [Non-invasive assessment of liver fibrosis with impulse elastography: Comparison of Supersonic Shear Imaging with ARFI and FibroScan®](http://www.sciencedirect.com/science/article/pii/S0168827814003079)
    1. Original research article
    2. Journal of Hepatology,
    3. Volume 61, Issue 3,
    4. September 2014,
    5. Pages 550-557
    6. Christophe Cassinotto,
    7. Bruno Lapuyade,
    8. Amaury Mouries,
    9. Jean-Baptiste Hiriart,
    10. Victor De Ledinghen
    11. [Purchase PDF](http://www.sciencedirect.com/science/article/pii/S0168827814003079)
36. [Factor VII activating protease (FSAP) exerts anti-inflammatory and anti-fibrotic effects in liver fibrosis in mice and men](http://www.sciencedirect.com/science/article/pii/S0168827812006976)
    1. Original research article
    2. Journal of Hepatology,
    3. Volume 58, Issue 1,
    4. January 2013,
    5. Pages 104-111
    6. Erawan Borkham-Kamphorst,
    7. Henning W. Zimmermann,
    8. Nikolaus Gassler,
    9. Ute Bissels,
    10. Sandip M. Kanse
    11. [Purchase PDF](http://www.sciencedirect.com/science/article/pii/S0168827812006976)
37. [Diagnosis of primary biliary cirrhosis](http://www.sciencedirect.com/science/article/pii/S152169181100093X)
    1. Original research article
    2. Best Practice & Research Clinical Gastroenterology,
    3. Volume 25, Issue 6,
    4. December 2011,
    5. Pages 701-712
    6. Gideon M. Hirschfield
    7. [Purchase PDF](http://www.sciencedirect.com/science/article/pii/S152169181100093X)
38. [Use of Thrombopoietic Agents for the Thrombocytopenia of Liver Disease](http://www.sciencedirect.com/science/article/pii/S0037196310000594)
    1. Original research article
    2. Seminars in Hematology,
    3. Volume 47, Issue 3,
    4. July 2010,
    5. Pages 266-273
    6. Hans L. Tillmann,
    7. John G. McHutchison
    8. [Purchase PDF](http://www.sciencedirect.com/science/article/pii/S0037196310000594)
39. [Four years experience with the AEC residents E-Book](http://www.sciencedirect.com/science/article/pii/S2173507709700821)
    1. Original research article
    2. Cirugía Española (English Edition),
    3. Volume 86, Issue 3,
    4. 2009,
    5. Pages 147-153
    6. Xavier Serra Aracil,
    7. Salvador Navarro Soto,
    8. Óscar Aparicio Rodríguez,
    9. Judit Hermoso Bosch,
    10. Carlos Gómez Díaz
    11. [Purchase PDF](http://www.sciencedirect.com/science/article/pii/S2173507709700821)
40. [Transplantation intrapéritonéale d'hépatocytes isolés de porc: le foie bioartificiel implantable](http://www.sciencedirect.com/science/article/pii/S0001400198800378)
    1. Original research article
    2. Chirurgie,
    3. Volume 123, Issue 1,
    4. February 1998,
    5. Pages 41-46
    6. R Sarkis,
    7. L Wen,
    8. J Honiger,
    9. M Baudrimont,
    10. B Nordlinger
    11. [Purchase PDF](http://www.sciencedirect.com/science/article/pii/S0001400198800378)
41. [The role of transient elastography in patients with hepatitis B viral disease](http://www.sciencedirect.com/science/article/pii/S1590865810606895)
    1. Original research article
    2. Digestive and Liver Disease,
    3. Volume 43, Supplement 1,
    4. January 2011,
    5. Pages s25-s31
    6. Mirella Fraquelli,
    7. Federica Branchi
    8. [Purchase PDF](http://www.sciencedirect.com/science/article/pii/S1590865810606895)
42. [Historia natural y manifestaciones clínicas de la hepatitis B crónica](http://www.sciencedirect.com/science/article/pii/S0213005X08765157)
    1. Original research article
    2. Enfermedades Infecciosas y Microbiología Clínica,
    3. Volume 26, Supplement 7,
    4. May 2008,
    5. Pages 11-18
    6. Miguel Carneiro de Moura,
    7. Rui Marinho
    8. [Purchase PDF](http://www.sciencedirect.com/science/article/pii/S0213005X08765157)
43. [Coinfection VIH-VHC : Quelle prise en charge ?](http://www.sciencedirect.com/science/article/pii/S1294550104942562)
    1. Original research article
    2. Antibiotiques,
    3. Volume 6, Issue 3,
    4. September 2004,
    5. Pages 151-163
    6. H. Aumaître,
    7. E. Chauvet,
    8. M. Medus,
    9. M. Saada
    10. [Purchase PDF](http://www.sciencedirect.com/science/article/pii/S1294550104942562)
44. [Progress in Renal Transplantation for Children](http://www.sciencedirect.com/science/article/pii/S1073444900700264)
    1. Original research article
    2. Advances in Renal Replacement Therapy,
    3. Volume 7, Issue 2,
    4. April 2000,
    5. Pages 158-171
    6. Jodi M. Smith,
    7. Ruth A. McDonald
    8. [Purchase PDF](http://www.sciencedirect.com/science/article/pii/S1073444900700264)
45. [Les pneumonies à cytomégalovirus](http://www.sciencedirect.com/science/article/pii/0924420496836018)
    1. Original research article
    2. Annales de l'Institut Pasteur / Actualités,
    3. Volume 6, Issue 1,
    4. 1995,
    5. Pages 9-17
    6. Anne-Marie Fillet
    7. [Purchase PDF](http://www.sciencedirect.com/science/article/pii/0924420496836018)
46. [Indicadores de calidad asistencial de GESIDA para la atención de personas infectadas por el VIH/sida](http://www.sciencedirect.com/science/article/pii/S0213005X10700483)
    1. Original research article
    2. Enfermedades Infecciosas y Microbiología Clínica,
    3. Volume 28, Supplement 5,
    4. November 2010,
    5. Pages 6-88
    6. Miguel Ángel von Wichmann,
    7. Jaime Locutura,
    8. José Ramón Blanco,
    9. Melchor Riera,
    10. Grupo de Estudio del Sida (GESIDA)
    11. [Purchase PDF](http://www.sciencedirect.com/science/article/pii/S0213005X10700483)
47. [25](http://www.sciencedirect.com/search?qs=%22Liver%20transplantation%22%20and%20APRI&show=25&sortBy=relevance&articleTypes=OR&navigation=true)
48. [50](http://www.sciencedirect.com/search?qs=%22Liver%20transplantation%22%20and%20APRI&show=50&sortBy=relevance&articleTypes=OR&navigation=true)
49. 100
50. [previous](http://www.sciencedirect.com/search?qs=%22Liver%20transplantation%22%20and%20APRI&show=100&sortBy=relevance&articleTypes=OR&navigation=true&offset=0)
51. Page 2 of 2

[Feedback](http://www.sciencedirect.com/search?qs=%22Liver%20transplantation%22%20and%20APRI&show=100&sortBy=relevance&articleTypes=OR&navigation=true&offset=100)

[About ScienceDirect](http://www.elsevier.com/solutions/sciencedirect)[Remote access](http://www.sciencedirect.com/science/activateaccess)[Shopping cart](http://www.sciencedirect.com/science?_ob=ShoppingCartURL&_method=display&_zone=TopNavBar&_origin=srp&md5=e5857d1631c4b1f612897ed029064507)[Contact and support](https://service.elsevier.com/app/contact/supporthub/sciencedirect/)[Terms and conditions](https://www.elsevier.com/legal/elsevier-website-terms-and-conditions)[Privacy policy](http://www.elsevier.com/legal/privacy-policy)

Cookies are used by this site. For more information, visit the [cookies page](https://www.elsevier.com/solutions/sciencedirect/support/cookies).

Copyright © 2017 Elsevier B.V. or its licensors or contributors. ScienceDirect ® is a registered trademark of Elsevier B.V.

Bottom of Form

FIB-4

[Skip to Main content](http://www.sciencedirect.com/search?qs=%22Liver%20transplantation%22%20and%20FIB-4&show=100&sortBy=relevance&articleTypes=OR&lastSelectedFacet=articleTypes#main_content)

- [Journals](http://www.sciencedirect.com/science/journals)
- [Books](http://www.sciencedirect.com/science/bookbshsrw)
- [Register](https://www.sciencedirect.com/user/register?returnURL=http%3A%2F%2Fwww.sciencedirect.com%2Fsearch)
- [Sign in](https://www.sciencedirect.com/user/login?returnURL=http%3A%2F%2Fwww.sciencedirect.com%2Fsearch)

Top of Form


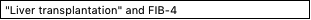


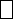


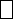


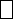


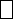


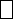


[Advanced search](http://www.sciencedirect.com/science/search)

Bottom of Form

137 results

Refine by:

Top of Form

Years

1.
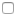
 2017 (15)
2.
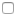
 2016 (15)
3.
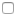
 2015 (12)
4.
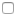
 2014 (12)
5.
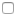
 2013 (8)
6.
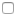
 2012 (3)
7.
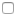
 2011 (6)
8.
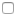
 2010 (4)
9.
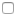
 2009 (5)
10.
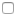
 2008 (3)

Show less

Article type

1.
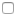
 Review articles (55)
2.
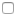
 Original research (137)
3.
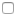
 Encyclopedia (2)
4. Book chapters (34)
5. Other (343)

Show less

Publication title

1. Journal of Hepatology (12)
2. Transplantation Proceedings (10)
3. Gastroenterology (10)
4. Digestive and Liver Disease (9)
5. Clinical Gastroenterology and Hepatology (8)
6. Journal of Cardiothoracic and Vascular Anesthesia (6)
7. Thrombosis Research (5)
8. The American Journal of Cardiology (3)
9. Human Pathology (3)
10. Transplantation Reviews (3)

Show less

[Clear all filters](http://www.sciencedirect.com/search?qs=%22Liver%20transplantation%22%20and%20FIB-4&show=100&sortBy=relevance)

Bottom of Form

sorted by *relevance* | [date](http://www.sciencedirect.com/search?qs=%22Liver%20transplantation%22%20and%20FIB-4&show=100&sortBy=date&articleTypes=OR)

1. [APRI and FIB-4 Scores Are Useful After Liver Transplantation Independently of Etiology](http://www.sciencedirect.com/science/article/pii/S0041134508016564)
   1. Original research article
   2. Transplantation Proceedings,
   3. Volume 41, Issue 2,
   4. March 2009,
   5. Pages 679-681
   6. A. Pissaia,
   7. D. Borderie,
   8. D. Bernard,
   9. O. Scatton,
   10. F. Conti
   11. [Purchase PDF](http://www.sciencedirect.com/science/article/pii/S0041134508016564)
2. [Diagnostic accuracy of APRI and FIB-4 for predicting hepatitis B virus-related liver fibrosis accompanied with hepatocellular carcinoma](http://www.sciencedirect.com/science/article/pii/S1590865815304278)
   1. Original research article
   2. Digestive and Liver Disease,
   3. Volume 48, Issue 10,
   4. October 2016,
   5. Pages 1220-1226
   6. Guangqin Xiao,
   7. Feng Zhu,
   8. Min Wang,
   9. Hang Zhang,
   10. Renyi Qin
   11. [Purchase PDF](http://www.sciencedirect.com/science/article/pii/S1590865815304278)
3. Want a richer search experience?

Sign in for additional filter options, multiple article downloads, and more.

Top of Form

Sign in

Bottom of Form

1. [Fibrinogen: A Clinical Update on Liver Transplantation](http://www.sciencedirect.com/science/article/pii/S0041134515010179)
   1. Original research article
   2. Transplantation Proceedings,
   3. Volume 47, Issue 10,
   4. December 2015,
   5. Pages 2925-2928
   6. A. Sabate,
   7. A. Dalmau
   8. [Purchase PDF](http://www.sciencedirect.com/science/article/pii/S0041134515010179)
2. [Remain recipient partial liver during liver transplant after Hassab](http://www.sciencedirect.com/science/article/pii/S0022480414001875)
   1. Original research article
   2. Journal of Surgical Research,
   3. Volume 189, Issue 2,
   4. 15 June 2014,
   5. Pages 321-325
   6. Jianhua Rao,
   7. Yong Sun,
   8. Haoming Zhou,
   9. Guoqiang Li,
   10. Ling Lu
   11. [Purchase PDF](http://www.sciencedirect.com/science/article/pii/S0022480414001875)
3. [A laboratory marker, FIB-4 index, as a predictor for long-term outcomes of hepatocellular carcinoma patients after curative hepatic resection](http://www.sciencedirect.com/science/article/pii/S0039606014007831)
   1. Original research article
   2. Surgery,
   3. Volume 157, Issue 4,
   4. April 2015,
   5. Pages 699-707
   6. Hidenori Toyoda,
   7. Takashi Kumada,
   8. Toshifumi Tada,
   9. Yuji Kaneoka,
   10. Atsuyuki Maeda
   11. [Purchase PDF](http://www.sciencedirect.com/science/article/pii/S0039606014007831)
4. [Liver transplantation: the Italian experience](http://www.sciencedirect.com/science/article/pii/S1590865802802079)
   1. Original research article
   2. Digestive and Liver Disease,
   3. Volume 34, Issue 9,
   4. September 2002,
   5. Pages 640-648
   6. S. Fagiuoli,
   7. V.G. Mirante,
   8. M. Pompili,
   9. S. Gianni,
   10. Monotematica AISF 2000-OLT Study Group
   11. [Purchase PDF](http://www.sciencedirect.com/science/article/pii/S1590865802802079)
5. [The Effects of In Vitro Hemodilution and Fibrinogen Concentrate Substitution on Thromboelastometry Analysis in Patients Qualified for Liver Transplantation – Preliminary Results](http://www.sciencedirect.com/science/article/pii/S0041134514008343)
   1. Original research article
   2. Transplantation Proceedings,
   3. Volume 46, Issue 8,
   4. October 2014,
   5. Pages 2758-2761
   6. B. Nicińska,
   7. J. Pluta,
   8. M. Kosieradzki,
   9. B. Łągiewska,
   10. J. Trzebicki
   11. [Purchase PDF](http://www.sciencedirect.com/science/article/pii/S0041134514008343)
6. [Features of Severe Liver Disease With Portal Hypertension in Patients With Cystic Fibrosis](http://www.sciencedirect.com/science/article/pii/S1542356516300167)
   1. Original research article
   2. Clinical Gastroenterology and Hepatology,
   3. Volume 14, Issue 8,
   4. August 2016,
   5. Pages 1207-1215.e3
   6. Jaclyn R. Stonebraker,
   7. Chee Y. Ooi,
   8. Rhonda G. Pace,
   9. Harriet Corvol,
   10. Simon C. Ling
   11. [Purchase PDF](http://www.sciencedirect.com/science/article/pii/S1542356516300167)
7. [Endogenous Thrombin Potential and Examination of a Further 31 Analytes in Liver Transplant Candidates](http://www.sciencedirect.com/science/article/pii/S0041134514004072)
   1. Original research article
   2. Transplantation Proceedings,
   3. Volume 46, Issue 6,
   4. July–August 2014,
   5. Pages 2171-2176
   6. F. Kovacs,
   7. Zs. Gerlei,
   8. D. Gorog,
   9. M. Varga,
   10. E. Sarvary
   11. [Purchase PDF](http://www.sciencedirect.com/science/article/pii/S0041134514004072)
8. [Hemostatic variation during perioperative period of orthotopic liver transplantation without venovenous bypass](http://www.sciencedirect.com/science/article/pii/S0049384807003854)
   1. Original research article
   2. Thrombosis Research,
   3. Volume 122, Issue 2,
   4. 2008,
   5. Pages 161-166
   6. Yuliang Wang,
   7. Yawu Liu,
   8. Ruifa Han,
   9. Zhijun Zhu,
   10. Zhongyang Shen
   11. [Purchase PDF](http://www.sciencedirect.com/science/article/pii/S0049384807003854)
9. [Quantification of Both Platelet Count and Fibrinogen Concentration Using Maximal Clot Firmness of Thromboelastometry During Liver Transplantation](http://www.sciencedirect.com/science/article/pii/S0041134515004753)
   1. Original research article
   2. Transplantation Proceedings,
   3. Volume 47, Issue 6,
   4. July–August 2015,
   5. Pages 1890-1895
   6. S.-M. Jeong,
   7. J.-G. Song,
   8. H. Seo,
   9. J.-H. Choi,
   10. G.-S. Hwang
   11. [Purchase PDF](http://www.sciencedirect.com/science/article/pii/S0041134515004753)
10. [Effectiveness of hepatitis C antiviral treatment in a USA cohort of veteran patients with hepatocellular carcinoma](http://www.sciencedirect.com/science/article/pii/S0168827817301265)
    1. Original research article
    2. Journal of Hepatology,
    3. Volume 67, Issue 1,
    4. July 2017,
    5. Pages 32-39
    6. Lauren A. Beste,
    7. Pamela K. Green,
    8. Kristin Berry,
    9. Matthew J. Kogut,
    10. George N. Ioannou
    11. [Purchase PDF](http://www.sciencedirect.com/science/article/pii/S0168827817301265)
11. [Transfusion Triggers in Orthotopic Liver Transplantation: A Comparison of the Thromboelastometry Analyzer, the Thromboelastogram, and Conventional Coagulation Tests](http://www.sciencedirect.com/science/article/pii/S1053077006000036)
    1. Original research article
    2. Journal of Cardiothoracic and Vascular Anesthesia,
    3. Volume 20, Issue 4,
    4. August 2006,
    5. Pages 548-553
    6. Margaret Coakley,
    7. Kalpana Reddy,
    8. Ian Mackie,
    9. Susan Mallett
    10. [Purchase PDF](http://www.sciencedirect.com/science/article/pii/S1053077006000036)
12. [Significance of Postoperative Changes in Hemodynamics and Biochemical Indices in Pediatric Recipients of Live-Donor Liver Transplants](http://www.sciencedirect.com/science/article/pii/S0041134513007665)
    1. Original research article
    2. Transplantation Proceedings,
    3. Volume 45, Issue 9,
    4. November 2013,
    5. Pages 3320-3324
    6. Y. Chen,
    7. F. Xu,
    8. L. Hu,
    9. C. Liu,
    10. J. Li
    11. [Purchase PDF](http://www.sciencedirect.com/science/article/pii/S0041134513007665)
13. [Fibrosis index based on four factors better predicts advanced fibrosis or cirrhosis than aspartate aminotransferase/platelet ratio index in chronic hepatitis C patients](http://www.sciencedirect.com/science/article/pii/S0929664615002417)
    1. Open archive,
    2. Original research article
    3. Journal of the Formosan Medical Association,
    4. Volume 114, Issue 10,
    5. October 2015,
    6. Pages 923-928
    7. Chia-Chi Wang,
    8. Chen-Hua Liu,
    9. Chih-Lin Lin,
    10. Pin-Chao Wang,
    11. Jia-Horng Kao
    12. [Download PDF (375 KB)](http://www.sciencedirect.com/science/article/pii/S0929664615002417/pdfft?md5=68465eac6e84326a9e1d1acb9f5c709f&pid=1-s2.0-S0929664615002417-main.pdf)
14. [Liver transplantation for primary sclerosing cholangitis: a long-term clinicopathologic study](http://www.sciencedirect.com/science/article/pii/S0046817703004854)
    1. Original research article
    2. Human Pathology,
    3. Volume 34, Issue 11,
    4. November 2003,
    5. Pages 1127-1136
    6. Urmila Khettry,
    7. Andrew Keaveny,
    8. Atoussa Goldar-Najafi,
    9. W.David Lewis,
    10. Fredric D Gordon
    11. [Purchase PDF](http://www.sciencedirect.com/science/article/pii/S0046817703004854)
15. [Successful case of adult ABO-incompatible liver transplantation: Beneficial effects of intrahepatic artery infusion therapy: A case report](http://www.sciencedirect.com/science/article/pii/S0041134504009649)
    1. Original research article
    2. Transplantation Proceedings,
    3. Volume 36, Issue 8,
    4. October 2004,
    5. Pages 2269-2273
    6. Y. Nakamura,
    7. N. Matsuno,
    8. H. Iwamoto,
    9. T. Yokoyama,
    10. T. Nagao
    11. [Purchase PDF](http://www.sciencedirect.com/science/article/pii/S0041134504009649)
16. [Myocardial ischemia after orthotopic liver transplantation](http://www.sciencedirect.com/science/article/pii/000291499490491X)
    1. Original research article
    2. The American Journal of Cardiology,
    3. Volume 74, Issue 1,
    4. 1 July 1994,
    5. Pages 53-56
    6. Daniel A. Rubin,
    7. Douglas S. Schulman,
    8. Todd D. Edwards,
    9. Thomas E. Starzl,
    10. Edward I. Curtiss
    11. [Purchase PDF](http://www.sciencedirect.com/science/article/pii/000291499490491X)
17. [Two-step Separation-free Quantitative Detection of HSA and FIB in Human Blood Plasma by a Pentaphenylpyyrrole Derivative with Aggregation-Enhanced Emission Properties](http://www.sciencedirect.com/science/article/pii/S092540051731571X)
    1. Original research article
    2. Sensors and Actuators B: Chemical,
    3. In press, accepted manuscript,
    4. Available online 24 August 2017
    5. Didi Chen,
    6. Lichao Dong,
    7. Shuai Jiang,
    8. Wangyang Li,
    9. Yuping Dong
    10. [Purchase PDF](http://www.sciencedirect.com/science/article/pii/S092540051731571X)
18. [Simple Noninvasive Systems Predict Long-term Outcomes of Patients With Nonalcoholic Fatty Liver Disease](http://www.sciencedirect.com/science/article/pii/S0016508513010123)
    1. Original research article
    2. Gastroenterology,
    3. Volume 145, Issue 4,
    4. October 2013,
    5. Pages 782-789.e4
    6. Paul Angulo,
    7. Elisabetta Bugianesi,
    8. Einar S. Bjornsson,
    9. Phunchai Charatcharoenwitthaya,
    10. Jacob George
    11. [Purchase PDF](http://www.sciencedirect.com/science/article/pii/S0016508513010123)
19. [Anticoagulant therapy in living-related liver transplantation](http://www.sciencedirect.com/science/article/pii/S0041134502034140)
    1. Original research article
    2. Transplantation Proceedings,
    3. Volume 34, Issue 7,
    4. November 2002,
    5. Pages 2788-2790
    6. N Taniai,
    7. M Onda,
    8. T Tajiri,
    9. K Akimaru,
    10. Y Mizuguchi
    11. [Purchase PDF](http://www.sciencedirect.com/science/article/pii/S0041134502034140)
20. [Recovery of bile secretion following orthotopic liver transplantation](http://www.sciencedirect.com/science/article/pii/0168827891908394)
    1. Original research article
    2. Journal of Hepatology,
    3. Volume 12, Issue 3,
    4. May 1991,
    5. Pages 351-361
    6. Mitchell L. Shiffman,
    7. Robert L. Carithers,
    8. Marc P. Posner,
    9. Edward W. Moore
    10. [Purchase PDF](http://www.sciencedirect.com/science/article/pii/0168827891908394)
21. [Transfusion support in liver transplantation](http://www.sciencedirect.com/science/article/pii/S0955388605800049)
    1. Original research article
    2. Transfusion Science,
    3. Volume 14, Issue 4,
    4. October 1993,
    5. Pages 345-352
    6. Darrell J. Triulzi,
    7. Franklin A. Bontempo,
    8. Joseph E. Kiss,
    9. Alan Winkelstein
    10. [Purchase PDF](http://www.sciencedirect.com/science/article/pii/S0955388605800049)
22. [Hepatocellular carcinoma in the absence of cirrhosis in patients with chronic hepatitis B virus infection](http://www.sciencedirect.com/science/article/pii/S0168827816305402)
    1. Original research article
    2. Journal of Hepatology,
    3. Volume 66, Issue 2,
    4. February 2017,
    5. Pages 355-362
    6. Maneerat Chayanupatkul,
    7. Ronald Omino,
    8. Sahil Mittal,
    9. Jennifer R. Kramer,
    10. Fasiha Kanwal
    11. [Purchase PDF](http://www.sciencedirect.com/science/article/pii/S0168827816305402)
23. [Surveillance for Hepatocellular Carcinoma in Patients with Cirrhosis Improves Outcome](http://www.sciencedirect.com/science/article/pii/S0002934307009928)
    1. Original research article
    2. The American Journal of Medicine,
    3. Volume 121, Issue 2,
    4. February 2008,
    5. Pages 119-126
    6. Richard Todd Stravitz,
    7. Douglas M. Heuman,
    8. Nisha Chand,
    9. Richard K. Sterling,
    10. Robert A. Fisher
    11. [Purchase PDF](http://www.sciencedirect.com/science/article/pii/S0002934307009928)
24. [Noninvasive Tests for Fibrosis and Liver Stiffness Predict 5-Year Outcomes of Patients With Chronic Hepatitis C](http://www.sciencedirect.com/science/article/pii/S0016508511002708)
    1. Original research article
    2. Gastroenterology,
    3. Volume 140, Issue 7,
    4. June 2011,
    5. Pages 1970-1979.e3
    6. Julien Vergniol,
    7. Juliette Foucher,
    8. Eric Terrebonne,
    9. Pierre–Henri Bernard,
    10. Victor de Ledinghen
    11. [Purchase PDF](http://www.sciencedirect.com/science/article/pii/S0016508511002708)
25. [Limited reliability of five non-invasive biomarkers in predicting hepatic fibrosis in chronic HCV mono-infected patients opposed to METAVIR scoring](http://www.sciencedirect.com/science/article/pii/S0344033814002076)
    1. Original research article
    2. Pathology - Research and Practice,
    3. Volume 210, Issue 12,
    4. December 2014,
    5. Pages 922-928
    6. Basem Hasan Elesawy,
    7. Amal Abd El hafez,
    8. Laila Shehata Dorgham,
    9. Ahmad El-Askary
    10. [Purchase PDF](http://www.sciencedirect.com/science/article/pii/S0344033814002076)
26. [Non-invasive tests in prediction of liver fibrosis in chronic hepatitis B and comparison with post-antiviral treatment results](http://www.sciencedirect.com/science/article/pii/S2210740112002070)
    1. Original research article
    2. Clinics and Research in Hepatology and Gastroenterology,
    3. Volume 37, Issue 2,
    4. April 2013,
    5. Pages 152-158
    6. Ömer Başar,
    7. Barış Yımaz,
    8. Fuat Ekiz,
    9. Zeynep Giniş,
    10. Osman Yüksel
    11. [Purchase PDF](http://www.sciencedirect.com/science/article/pii/S2210740112002070)
27. [Liver Fibrosis, but No Other Histologic Features, Is Associated With Long-term Outcomes of Patients With Nonalcoholic Fatty Liver Disease](http://www.sciencedirect.com/science/article/pii/S0016508515005995)
    1. Original research article
    2. Gastroenterology,
    3. Volume 149, Issue 2,
    4. August 2015,
    5. Pages 389-397.e10
    6. Paul Angulo,
    7. David E. Kleiner,
    8. Sanne Dam-Larsen,
    9. Leon A. Adams,
    10. Flemming Bendtsen
    11. [Purchase PDF](http://www.sciencedirect.com/science/article/pii/S0016508515005995)
28. [Use of quantitative liver function tests — caffeine clearance and galactose elimination capacity — after orthotopic liver transplantation](http://www.sciencedirect.com/science/article/pii/016882789090044R)
    1. Original research article
    2. Journal of Hepatology,
    3. Volume 10, Issue 2,
    4. March 1990,
    5. Pages 149-157
    6. Robyn A. Nagel,
    7. Luc Y. Dirix,
    8. Karen M. Hayllar,
    9. Rudolph Preisig,
    10. Roger Williams
    11. [Purchase PDF](http://www.sciencedirect.com/science/article/pii/016882789090044R)
29. [Statins Are Associated With a Decreased Risk of Decompensation and Death in Veterans With Hepatitis C–Related Compensated Cirrhosis](http://www.sciencedirect.com/science/article/pii/S0016508515014766)
    1. Original research article
    2. Gastroenterology,
    3. Volume 150, Issue 2,
    4. February 2016,
    5. Pages 430-440.e1
    6. Arpan Mohanty,
    7. Janet P. Tate,
    8. Guadalupe Garcia-Tsao
    9. [Purchase PDF](http://www.sciencedirect.com/science/article/pii/S0016508515014766)
30. [Relating the liver damage with hepatitis C virus polymorphism in core region and human variables in HIV-1-coinfected patients](http://www.sciencedirect.com/science/article/pii/S1567134810002212)
    1. Original research article
    2. Infection, Genetics and Evolution,
    3. Volume 10, Issue 8,
    4. December 2010,
    5. Pages 1252-1261
    6. Marina Matas,
    7. Antònia Picornell,
    8. Carmen Cifuentes,
    9. Antoni Payeras,
    10. José A. Castro
    11. [Purchase PDF](http://www.sciencedirect.com/science/article/pii/S1567134810002212)
31. [Is the aminoterminal propeptide of type III procollagen degraded in the liver?: A study of type III procollagen peptide in serum during liver transplantation in pigs](http://www.sciencedirect.com/science/article/pii/S0168827888800254)
    1. Original research article
    2. Journal of Hepatology,
    3. Volume 6, Issue 2,
    4. 1988,
    5. Pages 144-150
    6. Kirsten D. Bentsen,
    7. Steen Boesby,
    8. Preben Kirkegaard,
    9. Carsten Paln˦s Hansen,
    10. Ib Lorenzen
    11. [Purchase PDF](http://www.sciencedirect.com/science/article/pii/S0168827888800254)
32. [Baseline Values and Changes in Liver Stiffness Measured by Transient Elastography Are Associated With Severity of Fibrosis and Outcomes of Patients With Primary Sclerosing Cholangitis](http://www.sciencedirect.com/science/article/pii/S0016508513018441)
    1. Original research article
    2. Gastroenterology,
    3. Volume 146, Issue 4,
    4. April 2014,
    5. Pages 970-979.e6
    6. Christophe Corpechot,
    7. Farid Gaouar,
    8. Ahmed El Naggar,
    9. Astrid Kemgang,
    10. Olivier Chazouillères
    11. [Purchase PDF](http://www.sciencedirect.com/science/article/pii/S0016508513018441)
33. [Use of Analytic Morphomics of Liver, Spleen, and Body Composition to Identify Patients at Risk for Cirrhosis](http://www.sciencedirect.com/science/article/pii/S1542356514010878)
    1. Original research article
    2. Clinical Gastroenterology and Hepatology,
    3. Volume 13, Issue 2,
    4. February 2015,
    5. Pages 360-368.e5
    6. Venkat Krishnamurthy,
    7. Peng Zhang,
    8. Sampath Ethiraj,
    9. Binu Enchakalody,
    10. Grace L. Su
    11. [Purchase PDF](http://www.sciencedirect.com/science/article/pii/S1542356514010878)
34. [Performance and utility of transient elastography and noninvasive markers of liver fibrosis in primary biliary cirrhosis](http://www.sciencedirect.com/science/article/pii/S1590865811002258)
    1. Original research article
    2. Digestive and Liver Disease,
    3. Volume 43, Issue 11,
    4. November 2011,
    5. Pages 887-892
    6. Annarosa Floreani,
    7. Nora Cazzagon,
    8. Diego Martines,
    9. Luisa Cavalletto,
    10. Liliana Chemello
    11. [Purchase PDF](http://www.sciencedirect.com/science/article/pii/S1590865811002258)
35. [Non-invasive assessment of hepatic fibrosis in a series of patients with Wilson's Disease](http://www.sciencedirect.com/science/article/pii/S1590865811004750)
    1. Original research article
    2. Digestive and Liver Disease,
    3. Volume 44, Issue 6,
    4. June 2012,
    5. Pages 487-491
    6. Margherita Sini,
    7. Orazio Sorbello,
    8. Alberto Civolani,
    9. Mauro Liggi,
    10. Luigi Demelia
    11. [Purchase PDF](http://www.sciencedirect.com/science/article/pii/S1590865811004750)
36. [Effectiveness and safety of community-based treatment with sofosbuvir plus ribavirin for elderly patients with genotype 2 chronic hepatitis C](http://www.sciencedirect.com/science/article/pii/S1590865817308447)
    1. Original research article
    2. Digestive and Liver Disease,
    3. Volume 49, Issue 9,
    4. September 2017,
    5. Pages 1029-1035
    6. Masanori Atsukawa,
    7. Akihito Tsubota,
    8. Chisa Kondo,
    9. Noritomo Shimada,
    10. Katsuhiko Iwakiri
    11. [Purchase PDF](http://www.sciencedirect.com/science/article/pii/S1590865817308447)
37. [Comparative clinical characteristics and natural history of three variants of sclerosing cholangitis: IgG4-related SC, PSC/AIH and PSC alone](http://www.sciencedirect.com/science/article/pii/S1568997217301428)
    1. Original research article
    2. Autoimmunity Reviews,
    3. Volume 16, Issue 8,
    4. August 2017,
    5. Pages 875-882
    6. Min Lian,
    7. Bo Li,
    8. Xiao Xiao,
    9. Yue Yang,
    10. Xiong Ma
    11. [Purchase PDF](http://www.sciencedirect.com/science/article/pii/S1568997217301428)
38. [Similar Effectiveness of Boceprevir and Telaprevir Treatment Regimens for Hepatitis C Virus Infection on the Basis of a Nationwide Study of Veterans](http://www.sciencedirect.com/science/article/pii/S1542356513019381)
    1. Original research article
    2. Clinical Gastroenterology and Hepatology,
    3. Volume 12, Issue 8,
    4. August 2014,
    5. Pages 1371-1380
    6. George N. Ioannou,
    7. Lauren A. Beste,
    8. Pamela K. Green
    9. [Purchase PDF](http://www.sciencedirect.com/science/article/pii/S1542356513019381)
39. [Effectiveness of Sofosbuvir, Ledipasvir/Sofosbuvir, or Paritaprevir/Ritonavir/Ombitasvir and Dasabuvir Regimens for Treatment of Patients With Hepatitis C in the Veterans Affairs National Health Care System](http://www.sciencedirect.com/science/article/pii/S0016508516345759)
    1. Original research article
    2. Gastroenterology,
    3. Volume 151, Issue 3,
    4. September 2016,
    5. Pages 457-471.e5
    6. George N. Ioannou,
    7. Lauren A. Beste,
    8. Michael F. Chang,
    9. Pamela K. Green,
    10. Kristin Berry
    11. [Purchase PDF](http://www.sciencedirect.com/science/article/pii/S0016508516345759)
40. [Plasma exchange-centered artificial liver support system in hepatitis B virus-related acute-on-chronic liverfailure: a nationwide prospective multicenter study in China](http://www.sciencedirect.com/science/article/pii/S149938721660084X)
    1. Original research article
    2. Hepatobiliary & Pancreatic Diseases International,
    3. Volume 15, Issue 3,
    4. June 2016,
    5. Pages 275-281
    6. Jia-Jia Chen,
    7. Jian-Rong Huang,
    8. Qian Yang,
    9. Xiao-Wei Xu,
    10. Lan-Juan Li
    11. [Purchase PDF](http://www.sciencedirect.com/science/article/pii/S149938721660084X)
41. [Epidemiology and natural history of non-alcoholic fatty liver disease](http://www.sciencedirect.com/science/article/pii/S0026049516000275)
    1. Original research article
    2. Metabolism,
    3. Volume 65, Issue 8,
    4. August 2016,
    5. Pages 1017-1025
    6. Yousef Fazel,
    7. Aaron B. Koenig,
    8. Mehmet Sayiner,
    9. Zachary D. Goodman,
    10. Zobair M. Younossi
    11. [Purchase PDF](http://www.sciencedirect.com/science/article/pii/S0026049516000275)
42. [Determinants of Treatment Eligibility in Veterans With Hepatitis C Viral Infection](http://www.sciencedirect.com/science/article/pii/S0149291816308578)
    1. Original research article
    2. Clinical Therapeutics,
    3. Volume 39, Issue 1,
    4. January 2017,
    5. Pages 130-137
    6. Janice Taylor,
    7. Sian Carr-Lopez,
    8. Amy Robinson,
    9. Robert Malmstrom,
    10. Jannet M. Carmichael
    11. [Purchase PDF](http://www.sciencedirect.com/science/article/pii/S0149291816308578)
43. [Introduction of the resection severity index as independent risk factor limiting survival after resection of colorectal liver metastases](http://www.sciencedirect.com/science/article/pii/S0960740417301809)
    1. Original research article
    2. Surgical Oncology,
    3. Volume 26, Issue 4,
    4. December 2017,
    5. Pages 382-388
    6. Jill Gwiasda,
    7. Harald Schrem,
    8. Alexander Kaltenborn,
    9. Jan Mahlmann,
    10. Ulf Kulik
    11. [Purchase PDF](http://www.sciencedirect.com/science/article/pii/S0960740417301809)
44. [Alcohol use and hepatitis C virus treatment outcomes among patients receiving direct antiviral agents](http://www.sciencedirect.com/science/article/pii/S0376871616309644)
    1. Original research article
    2. Drug and Alcohol Dependence,
    3. Volume 169,
    4. 1 December 2016,
    5. Pages 101-109
    6. Judith I. Tsui,
    7. Emily C. Williams,
    8. Pamela K. Green,
    9. Kristin Berry,
    10. George N. Ioannou
    11. [Purchase PDF](http://www.sciencedirect.com/science/article/pii/S0376871616309644)
45. [AJKD Atlas of Renal Pathology: Hereditary and Other Non-AL Amyloidoses](http://www.sciencedirect.com/science/article/pii/S0272638615012469)
    1. Original research article
    2. American Journal of Kidney Diseases,
    3. Volume 66, Issue 6,
    4. December 2015,
    5. Pages e49-e51
    6. Agnes B. Fogo,
    7. Mark A. Lusco,
    8. Behzad Najafian,
    9. Charles E. Alpers
    10. [Purchase PDF](http://www.sciencedirect.com/science/article/pii/S0272638615012469)
46. [Repeated Measurements of Hepatitis B Surface Antigen Identify Carriers of Inactive HBV During Long-term Follow-up](http://www.sciencedirect.com/science/article/pii/S1542356516001221)
    1. Original research article
    2. Clinical Gastroenterology and Hepatology,
    3. Volume 14, Issue 10,
    4. October 2016,
    5. Pages 1481-1489.e5
    6. Willem P. Brouwer,
    7. Henry Lik-Yuen Chan,
    8. Maurizia R. Brunetto,
    9. Michelle Martinot-Peignoux,
    10. Good Practice in using HBsAg in Chronic Hepatitis B Study Group (GPs-CHB Study Group)
    11. [Purchase PDF](http://www.sciencedirect.com/science/article/pii/S1542356516001221)
47. [Effect of Sofosbuvir Plus Daclatasvir in Hepatitis C Virus Genotype-4 Patients: Promising Effect on LiverFibrosis](http://www.sciencedirect.com/science/article/pii/S0973688317304103)
    1. Original research article
    2. Journal of Clinical and Experimental Hepatology,
    3. In press, corrected proof,
    4. Available online 30 June 2017
    5. Asmaa M. Abdel-Aziz,
    6. Mohamed A. Ibrahim,
    7. Azza A. El-Sheikh,
    8. Maha Y. Kamel,
    9. Hisham Abdelhaleem
    10. [Purchase PDF](http://www.sciencedirect.com/science/article/pii/S0973688317304103)
48. [HCV mono-infected and HIV/HCV co-infected individuals treated with direct-acting antivirals: to what extent do they differ?](http://www.sciencedirect.com/science/article/pii/S1201971217301790)
    1. Open access,
    2. Original research article
    3. International Journal of Infectious Diseases,
    4. Volume 62,
    5. September 2017,
    6. Pages 64-71
    7. Giuseppe Bruno,
    8. Annalisa Saracino,
    9. Luigia Scudeller,
    10. Claudia Fabrizio,
    11. Gioacchino Angarano
    12. [Download PDF (632 KB)](http://www.sciencedirect.com/science/article/pii/S1201971217301790/pdfft?md5=1abc0df8b5b8e29a8e88dd3a3aea5e11&pid=1-s2.0-S1201971217301790-main.pdf)
49. [A Randomized Trial of Silymarin for the Treatment of Nonalcoholic Steatohepatitis](http://www.sciencedirect.com/science/article/pii/S1542356517304597)
    1. Original research article
    2. Clinical Gastroenterology and Hepatology,
    3. In press, corrected proof,
    4. Available online 15 April 2017
    5. Wah-Kheong Chan,
    6. Nik Raihan Nik Mustapha,
    7. Sanjiv Mahadeva
    8. [Purchase PDF](http://www.sciencedirect.com/science/article/pii/S1542356517304597)
50. [Serum Wisteria floribunda agglutinin-positive Mac-2-binding protein expression predicts disease severity in chronic hepatitis C patients](http://www.sciencedirect.com/science/article/pii/S1607551X17300670)
    1. Open access,
    2. Original research article
    3. The Kaohsiung Journal of Medical Sciences,
    4. Volume 33, Issue 8,
    5. August 2017,
    6. Pages 394-399
    7. Ching-I Huang,
    8. Chung-Feng Huang,
    9. Ming-Lun Yeh,
    10. Yi-Hung Lin,
    11. Wan-Long Chuang
    12. [Download PDF (495 KB)](http://www.sciencedirect.com/science/article/pii/S1607551X17300670/pdfft?md5=978476b240f48b4efe7f8d3a59b744a0&pid=1-s2.0-S1607551X17300670-main.pdf)
51. [Prediction of oesophageal varices in hepatic cirrhosis by simple serum non-invasive markers: Results of a multicenter, large-scale study](http://www.sciencedirect.com/science/article/pii/S0168827810005283)
    1. Original research article
    2. Journal of Hepatology,
    3. Volume 53, Issue 4,
    4. October 2010,
    5. Pages 630-638
    6. Giada Sebastiani,
    7. Diego Tempesta,
    8. Giovanna Fattovich,
    9. Laurent Castera,
    10. Alfredo Alberti
    11. [Purchase PDF](http://www.sciencedirect.com/science/article/pii/S0168827810005283)
52. [The utility of NAFLD fibrosis score for prediction of mortality among patients with nonalcoholic fatty liverdisease: A systematic review and meta-analysis of cohort study](http://www.sciencedirect.com/science/article/pii/S221074011730089X)
    1. Original research article
    2. Clinics and Research in Hepatology and Gastroenterology,
    3. In press, corrected proof,
    4. Available online 15 July 2017
    5. Veeravich Jaruvongvanich,
    6. Karn Wijarnpreecha,
    7. Patompong Ungprasert
    8. [Purchase PDF](http://www.sciencedirect.com/science/article/pii/S221074011730089X)
53. [Diffusion-weighted MRI of the transplanted liver](http://www.sciencedirect.com/science/article/pii/S0009926011001462)
    1. Original research article
    2. Clinical Radiology,
    3. Volume 66, Issue 9,
    4. September 2011,
    5. Pages 820-825
    6. K. Sandrasegaran,
    7. R. Ramaswamy,
    8. S. Ghosh,
    9. B. Tahir,
    10. P. Kwo
    11. [Purchase PDF](http://www.sciencedirect.com/science/article/pii/S0009926011001462)
54. [The vascular pathology of human hepatic allografts](http://www.sciencedirect.com/science/article/pii/004681779390298U)
    1. Original research article
    2. Human Pathology,
    3. Volume 24, Issue 2,
    4. February 1993,
    5. Pages 182-188
    6. Geoffrey Liu,
    7. Jagdish Butany,
    8. Ian R. Wanless,
    9. Ross Cameron,
    10. Gary Levy
    11. [Purchase PDF](http://www.sciencedirect.com/science/article/pii/004681779390298U)
55. [Fibrosing cholestatic hepatitis and HBV after bone marrow transplantation](http://www.sciencedirect.com/science/article/pii/0753332296826047)
    1. Original research article
    2. Biomedicine & Pharmacotherapy,
    3. Volume 49, Issue 3,
    4. 1995,
    5. Pages 117-124
    6. WGE Cooksley,
    7. CA McIvor
    8. [Purchase PDF](http://www.sciencedirect.com/science/article/pii/0753332296826047)
56. [Use of procalcitonin as indicator of nonviral infections in transplantation and related immunologic diseases](http://www.sciencedirect.com/science/article/pii/S0955470X00800105)
    1. Original research article
    2. Transplantation Reviews,
    3. Volume 14, Issue 1,
    4. January 2000,
    5. Pages 52-63
    6. Stefanie Hammer,
    7. Franz Meisner,
    8. Claus Hammer,
    9. Dietrich Seidel
    10. [Purchase PDF](http://www.sciencedirect.com/science/article/pii/S0955470X00800105)
57. [Online combination algorithm for non-invasive assessment of chronic hepatitis B related liver fibrosis and cirrhosis in resource-limited settings](http://www.sciencedirect.com/science/article/pii/S0953620515002319)
    1. Original research article
    2. European Journal of Internal Medicine,
    3. Volume 26, Issue 8,
    4. October 2015,
    5. Pages 628-634
    6. Nermin N. Salkic,
    7. Elmir Cickusic,
    8. Predrag Jovanovic,
    9. Mirela Basic Denjagic,
    10. Sead Ahmetagic
    11. [Purchase PDF](http://www.sciencedirect.com/science/article/pii/S0953620515002319)
58. [Safety and Effectiveness of Direct-Acting Antiviral Agents for Treatment of Patients With Chronic Hepatitis C Virus Infection and Cirrhosis](http://www.sciencedirect.com/science/article/pii/S1542356516303779)
    1. Original research article
    2. Clinical Gastroenterology and Hepatology,
    3. Volume 14, Issue 12,
    4. December 2016,
    5. Pages 1821-1830.e6
    6. Raoel Maan,
    7. Marjolein van Tilborg,
    8. Katja Deterding,
    9. Alnoor Ramji,
    10. Jordan J. Feld
    11. [Purchase PDF](http://www.sciencedirect.com/science/article/pii/S1542356516303779)
59. [Fibrosis in Hepatitis C Patients Predicts Complications After Elective Total Joint Arthroplasty](http://www.sciencedirect.com/science/article/pii/S0883540313002659)
    1. Original research article
    2. The Journal of Arthroplasty,
    3. Volume 29, Issue 1,
    4. January 2014,
    5. Pages 7-10
    6. Fabio Orozco,
    7. Zachary D. Post,
    8. Omkar Baxi,
    9. Adam Miller,
    10. Alvin Ong
    11. [Purchase PDF](http://www.sciencedirect.com/science/article/pii/S0883540313002659)
60. [The role of immunosuppression in recurrence of hepatitis C](http://www.sciencedirect.com/science/article/pii/S1527646503810651)
    1. Original research article
    2. Liver Transplantation,
    3. Volume 9, Issue 11,
    4. November 2003,
    5. Pages s63-s66
    6. John R. Lake
    7. [Purchase PDF](http://www.sciencedirect.com/science/article/pii/S1527646503810651)
61. [Impact of Liver Disease After the Fontan Operation](http://www.sciencedirect.com/science/article/pii/S0002914914020207)
    1. Original research article
    2. The American Journal of Cardiology,
    3. Volume 115, Issue 2,
    4. 15 January 2015,
    5. Pages 249-252
    6. Ian Lindsay,
    7. Joy Johnson,
    8. Melanie D. Everitt,
    9. James Hoffman,
    10. Anji T. Yetman
    11. [Purchase PDF](http://www.sciencedirect.com/science/article/pii/S0002914914020207)
62. [Impairment of thrombin generation in the early phases of the host response of sepsis](http://www.sciencedirect.com/science/article/pii/S0883944113003031)
    1. Original research article
    2. Journal of Critical Care,
    3. Volume 29, Issue 1,
    4. February 2014,
    5. Pages 31-36
    6. Susan K. Picoli-Quaino,
    7. Brunna E. Alves,
    8. Vanessa B. Faiotto,
    9. Silmara A.L. Montalvao,
    10. Erich V. De Paula
    11. [Purchase PDF](http://www.sciencedirect.com/science/article/pii/S0883944113003031)
63. [Invasive and non-invasive methods for the assessment of fibrosis and disease progression in chronic liverdisease](http://www.sciencedirect.com/science/article/pii/S1521691811000291)
    1. Original research article
    2. Best Practice & Research Clinical Gastroenterology,
    3. Volume 25, Issue 2,
    4. April 2011,
    5. Pages 291-303
    6. Laurent Castera
    7. [Purchase PDF](http://www.sciencedirect.com/science/article/pii/S1521691811000291)
64. [The efficacy and associated bleeding complications of recombinant antithrombin supplementation among intensive care unit patients](http://www.sciencedirect.com/science/article/pii/S0049384817303997)
    1. Open access,
    2. Original research article
    3. Thrombosis Research,
    4. Volume 157,
    5. September 2017,
    6. Pages 84-89
    7. Hiroyuki Koami,
    8. Yuichiro Sakamoto,
    9. Ryota Sakurai,
    10. Miho Ohta,
    11. Satoshi Inoue
    12. [Download PDF (354 KB)](http://www.sciencedirect.com/science/article/pii/S0049384817303997/pdfft?md5=71f58a48cc89b96b7bc9684a18786168&pid=1-s2.0-S0049384817303997-main.pdf)
65. [Acute normovolaemic haemodilution in cirrhotic patients undergoing major liver resection: Role of ROTEM](http://www.sciencedirect.com/science/article/pii/S1110184912000761)
    1. Open access,
    2. Original research article
    3. Egyptian Journal of Anaesthesia,
    4. Volume 29, Issue 1,
    5. January 2013,
    6. Pages 53-60
    7. Nirmeen A. Fayed,
    8. Emad K. Refaat,
    9. Hany A. Shoream,
    10. Sameh M. Hakim
    11. [Download PDF (341 KB)](http://www.sciencedirect.com/science/article/pii/S1110184912000761/pdfft?md5=895df44143786003fbff908136a4e3b0&pid=1-s2.0-S1110184912000761-main.pdf)
66. [High hepatic and extrahepatic mortality and low treatment uptake in HCV-coinfected persons in the Swiss HIV cohort study between 2001 and 2013](http://www.sciencedirect.com/science/article/pii/S0168827815003025)
    1. Original research article
    2. Journal of Hepatology,
    3. Volume 63, Issue 3,
    4. September 2015,
    5. Pages 573-580
    6. Helen Kovari,
    7. Bruno Ledergerber,
    8. Matthias Cavassini,
    9. Juan Ambrosioni,
    10. Swiss HIV Cohort Study
    11. [Purchase PDF](http://www.sciencedirect.com/science/article/pii/S0168827815003025)
67. [Efficacy and safety study of cenicriviroc for the treatment of non-alcoholic steatohepatitis in adult subjects with liver fibrosis: CENTAUR Phase 2b study design](http://www.sciencedirect.com/science/article/pii/S155171441630026X)
    1. Open access,
    2. Original research article
    3. Contemporary Clinical Trials,
    4. Volume 47,
    5. March 2016,
    6. Pages 356-365
    7. Scott Friedman,
    8. Arun Sanyal,
    9. Zachary Goodman,
    10. Eric Lefebvre,
    11. Vlad Ratziu
    12. [Download PDF (864 KB)](http://www.sciencedirect.com/science/article/pii/S155171441630026X/pdfft?md5=3dea94ae0f1dc6ecc9db50385aa2b14b&pid=1-s2.0-S155171441630026X-main.pdf)
68. [Prospective Observational Study of Hemostatic Alterations During Adult Extracorporeal Membrane Oxygenation (ECMO) Using Point-of-Care Thromboelastometry and Platelet Aggregometry](http://www.sciencedirect.com/science/article/pii/S1053077014002833)
    1. Original research article
    2. Journal of Cardiothoracic and Vascular Anesthesia,
    3. Volume 29, Issue 2,
    4. April 2015,
    5. Pages 288-296
    6. Priya Nair,
    7. Dominik Johannes Hoechter,
    8. Hergen Buscher,
    9. Karthik Venkatesh,
    10. Paul Jansz
    11. [Purchase PDF](http://www.sciencedirect.com/science/article/pii/S1053077014002833)
69. [Prediction on liver fibrosis using different APRI thresholds when patient age is a categorical marker in patients with chronic hepatitis B](http://www.sciencedirect.com/science/article/pii/S0009898110005292)
    1. Original research article
    2. Clinica Chimica Acta,
    3. Volume 412, Issues 1–2,
    4. 14 January 2011,
    5. Pages 33-37
    6. Hong-Bo Liu,
    7. Jian-Ping Zhou,
    8. Yong Zhang,
    9. Xiao-Hui Lv,
    10. Wei Wang
    11. [Purchase PDF](http://www.sciencedirect.com/science/article/pii/S0009898110005292)
70. [AISF position paper on nonalcoholic fatty liver disease (NAFLD): Updates and future directions](http://www.sciencedirect.com/science/article/pii/S1590865817301512)
    1. Original research article
    2. Digestive and Liver Disease,
    3. Volume 49, Issue 5,
    4. May 2017,
    5. Pages 471-483
    6. The Italian Association for the Study of the Liver (AISF)
    7. [Purchase PDF](http://www.sciencedirect.com/science/article/pii/S1590865817301512)
71. [Complete resection before development of drug resistance is essential for survival from advanced hepatoblastoma—A report from the German Cooperative Pediatric Liver Tumor Study HB-89](http://www.sciencedirect.com/science/article/pii/0022346895907629)
    1. Original research article
    2. Journal of Pediatric Surgery,
    3. Volume 30, Issue 6,
    4. June 1995,
    5. Pages 845-852
    6. D von Schweinitz,
    7. H Hecker,
    8. D Harms,
    9. U Bode,
    10. H Mildenberger
    11. [Purchase PDF](http://www.sciencedirect.com/science/article/pii/0022346895907629)
72. [Comparison of standard fibrinogen measurement methods with fibrin clot firmness assessed by thromboelastometry in patients with cirrhosis](http://www.sciencedirect.com/science/article/pii/S0049384815001589)
    1. Original research article
    2. Thrombosis Research,
    3. Volume 135, Issue 6,
    4. June 2015,
    5. Pages 1124-1130
    6. Dragica Vucelic,
    7. Rada Jesic,
    8. Snezana Jovicic,
    9. Maja Zivotic,
    10. Aleksandra Antovic
    11. [Purchase PDF](http://www.sciencedirect.com/science/article/pii/S0049384815001589)
73. [Slow regression of liver fibrosis presumed by repeated biomarkers after virological cure in patients with chronic hepatitis C](http://www.sciencedirect.com/science/article/pii/S0168827813003450)
    1. Original research article
    2. Journal of Hepatology,
    3. Volume 59, Issue 4,
    4. October 2013,
    5. Pages 675-683
    6. Thierry Poynard,
    7. Joseph Moussalli,
    8. Mona Munteanu,
    9. Dominique Thabut,
    10. FibroFrance-GHPS group
    11. [Purchase PDF](http://www.sciencedirect.com/science/article/pii/S0168827813003450)
74. [Hepatitis B Virus–Specific and Global T-Cell Dysfunction in Chronic Hepatitis B](http://www.sciencedirect.com/science/article/pii/S0016508515017369)
    1. Original research article
    2. Gastroenterology,
    3. Volume 150, Issue 3,
    4. March 2016,
    5. Pages 684-695.e5
    6. Jang-June Park,
    7. David K. Wong,
    8. Abdus S. Wahed,
    9. William M. Lee,
    10. Hepatitis B Research Network
    11. [Purchase PDF](http://www.sciencedirect.com/science/article/pii/S0016508515017369)
75. [A suggested algorithm for using serum biomarkers for the diagnosis of liver fibrosis in chronic hepatitis C infection](http://www.sciencedirect.com/science/article/pii/S168719791000119X)
    1. Original research article
    2. Arab Journal of Gastroenterology,
    3. Volume 11, Issue 4,
    4. December 2010,
    5. Pages 206-211
    6. Madiha M. El-Attar,
    7. Hebat-Allah G. Rashed,
    8. Eman M. Sewify,
    9. Howayda E. Hassan
    10. [Purchase PDF](http://www.sciencedirect.com/science/article/pii/S168719791000119X)
76. [Validity of Thromboelastometry for Rapid Assessment of Fibrinogen Levels in Heparinized Samples During Cardiac Surgery: A Retrospective, Single-center, Observational Study](http://www.sciencedirect.com/science/article/pii/S1053077015002827)
    1. Original research article
    2. Journal of Cardiothoracic and Vascular Anesthesia,
    3. Volume 30, Issue 1,
    4. January 2016,
    5. Pages 90-95
    6. Hamish Mace,
    7. Nicholas Lightfoot,
    8. Stuart McCluskey,
    9. Rita Selby,
    10. Keyvan Karkouti
    11. [Purchase PDF](http://www.sciencedirect.com/science/article/pii/S1053077015002827)
77. [Early Virologic Responses and Hematologic Safety of Direct-Acting Antiviral Therapies in Veterans With Chronic Hepatitis C](http://www.sciencedirect.com/science/article/pii/S1542356513003856)
    1. Original research article
    2. Clinical Gastroenterology and Hepatology,
    3. Volume 11, Issue 8,
    4. August 2013,
    5. Pages 1021-1027
    6. Pamela S. Belperio,
    7. Elizabeth W. Hwang,
    8. I. Chun Thomas,
    9. Larry A. Mole,
    10. Lisa I. Backus
    11. [Purchase PDF](http://www.sciencedirect.com/science/article/pii/S1542356513003856)
78. [Marcadores serológicos de fibrosis](http://www.sciencedirect.com/science/article/pii/S0210570512700444)
    1. Original research article
    2. Gastroenterología y Hepatología,
    3. Volume 35, Supplement 2,
    4. December 2012,
    5. Pages 10-16
    6. Guillermo Fernández-Varo
    7. [Purchase PDF](http://www.sciencedirect.com/science/article/pii/S0210570512700444)
79. [Surveillance de l'hémostase au cours de la transplantation hépatique : apport du thromboélastogramme](http://www.sciencedirect.com/science/article/pii/S0750765805806203)
    1. Original research article
    2. Annales Françaises d'Anesthésie et de Réanimation,
    3. Volume 12, Issue 6,
    4. 1993,
    5. Pages 544-551
    6. N. Gengenwin,
    7. A. Steib,
    8. G. Freys,
    9. S. Lévy,
    10. J.C. Otteni
    11. [Purchase PDF](http://www.sciencedirect.com/science/article/pii/S0750765805806203)
80. [Sarcopenia is an independent risk factor for non-alcoholic steatohepatitis and significant fibrosis](http://www.sciencedirect.com/science/article/pii/S0168827816304846)
    1. Original research article
    2. Journal of Hepatology,
    3. Volume 66, Issue 1,
    4. January 2017,
    5. Pages 123-131
    6. Bo Kyung Koo,
    7. Donghee Kim,
    8. Sae Kyung Joo,
    9. Jung Ho Kim,
    10. Won Kim
    11. [Purchase PDF](http://www.sciencedirect.com/science/article/pii/S0168827816304846)
81. [Microbiological features of KPC-producing Enterobacter isolates identified in a U.S. hospital system](http://www.sciencedirect.com/science/article/pii/S0732889314002557)
    1. Original research article
    2. Diagnostic Microbiology and Infectious Disease,
    3. Volume 80, Issue 2,
    4. October 2014,
    5. Pages 154-158
    6. Chulsoo Ahn,
    7. Alveena Syed,
    8. Fupin Hu,
    9. Jessica A. O’Hara,
    10. Yohei Doi
    11. [Purchase PDF](http://www.sciencedirect.com/science/article/pii/S0732889314002557)
82. [Comprehensive Preoperative Evaluation of Platelet Function in Total Knee Arthroplasty Patients Taking Diclofenac](http://www.sciencedirect.com/science/article/pii/S0883540307002331)
    1. Original research article
    2. The Journal of Arthroplasty,
    3. Volume 23, Issue 3,
    4. April 2008,
    5. Pages 424-430
    6. Kwok F.J. Ng,
    7. Jean-Claude Lawmin,
    8. Carina C.F. Li,
    9. Suk F. Tsang,
    10. Kwong Y. Chiu
    11. [Purchase PDF](http://www.sciencedirect.com/science/article/pii/S0883540307002331)
83. [Magnetic Resonance Imaging More Accurately Classifies Steatosis and Fibrosis in Patients With Nonalcoholic Fatty Liver Disease Than Transient Elastography](http://www.sciencedirect.com/science/article/pii/S0016508515017345)
    1. Open access,
    2. Original research article
    3. Gastroenterology,
    4. Volume 150, Issue 3,
    5. March 2016,
    6. Pages 626-637.e7
    7. Kento Imajo,
    8. Takaomi Kessoku,
    9. Yasushi Honda,
    10. Wataru Tomeno,
    11. Atsushi Nakajima
    12. [Download PDF (1,580 KB)](http://www.sciencedirect.com/science/article/pii/S0016508515017345/pdfft?md5=3127e601c346bb0639a230236cdacf22&pid=1-s2.0-S0016508515017345-main.pdf)
84. [Non-invasive assessment of liver fibrosis with impulse elastography: Comparison of Supersonic Shear Imaging with ARFI and FibroScan®](http://www.sciencedirect.com/science/article/pii/S0168827814003079)
    1. Original research article
    2. Journal of Hepatology,
    3. Volume 61, Issue 3,
    4. September 2014,
    5. Pages 550-557
    6. Christophe Cassinotto,
    7. Bruno Lapuyade,
    8. Amaury Mouries,
    9. Jean-Baptiste Hiriart,
    10. Victor De Ledinghen
    11. [Purchase PDF](http://www.sciencedirect.com/science/article/pii/S0168827814003079)
85. [Supraventricular tachycardia during pediatric anesthesia: a case series and qualitative analysis](http://www.sciencedirect.com/science/article/pii/S0952818014000488)
    1. Original research article
    2. Journal of Clinical Anesthesia,
    3. Volume 26, Issue 4,
    4. June 2014,
    5. Pages 257-263
    6. Chad C. Cripe,
    7. Akash R. Patel,
    8. Scott D. Markowitz,
    9. Tiffany S. Behringer,
    10. Ronald S. Litman
    11. [Purchase PDF](http://www.sciencedirect.com/science/article/pii/S0952818014000488)
86. [Noninvasive Methods to Assess Liver Disease in Patients With Hepatitis B or C](http://www.sciencedirect.com/science/article/pii/S0016508512002302)
    1. Original research article
    2. Gastroenterology,
    3. Volume 142, Issue 6,
    4. May 2012,
    5. Pages 1293-1302.e4
    6. Laurent Castera
    7. [Purchase PDF](http://www.sciencedirect.com/science/article/pii/S0016508512002302)
87. [Which are the cut-off values of 2D-Shear Wave Elastography (2D-SWE) liver stiffness measurements predicting different stages of liver fibrosis, considering Transient Elastography (TE) as the reference method?](http://www.sciencedirect.com/science/article/pii/S0720048X13006542)
    1. Original research article
    2. European Journal of Radiology,
    3. Volume 83, Issue 3,
    4. March 2014,
    5. Pages e118-e122
    6. Ioan Sporea,
    7. Simona Bota,
    8. Oana Gradinaru-Taşcău,
    9. Roxana Şirli,
    10. Ana Jurchiş
    11. [Purchase PDF](http://www.sciencedirect.com/science/article/pii/S0720048X13006542)
88. [Hepatitis por el virus C](http://www.sciencedirect.com/science/article/pii/S0304541216300294)
    1. Original research article
    2. Medicine - Programa de Formación Médica Continuada Acreditado,
    3. Volume 12, Issue 9,
    4. April 2016,
    5. Pages 484-493
    6. J. Cabezas,
    7. S. Llerena,
    8. S. Menéndez,
    9. C. Alonso Martín,
    10. J. Crespo García
    11. [Purchase PDF](http://www.sciencedirect.com/science/article/pii/S0304541216300294)
89. [Methods and outcomes of calcineurin inhibitor reduction or withdrawal in patients with chronic allograft nephropathy after the first year posttransplantation](http://www.sciencedirect.com/science/article/pii/S0041134501021133)
    1. Original research article
    2. Transplantation Proceedings,
    3. Volume 33, Issue 4, Supplement 1,
    4. June 2001,
    5. Pages s19-s28
    6. M.R Weir
    7. [Purchase PDF](http://www.sciencedirect.com/science/article/pii/S0041134501021133)
90. [Multiple approaches to assess fourteen non-invasive serum indexes for the diagnosis of liver fibrosis in chronic hepatitis C patients](http://www.sciencedirect.com/science/article/pii/S0009912016000874)
    1. Original research article
    2. Clinical Biochemistry,
    3. Volume 49, Issues 7–8,
    4. May 2016,
    5. Pages 560-565
    6. María Jesús Andrés-Otero,
    7. Ignacio De-Blas-Giral,
    8. Juan José Puente-Lanzarote,
    9. Trinidad Serrano-Aulló,
    10. José Manuel Lou-Bonafonte
    11. [Purchase PDF](http://www.sciencedirect.com/science/article/pii/S0009912016000874)
91. [Two novel POLG mutations causing hepatic mitochondrial DNA depletion with recurrent hypoketotic hypoglycaemia and fatal liver dysfunction](http://www.sciencedirect.com/science/article/pii/S1590865808007093)
    1. Original research article
    2. Digestive and Liver Disease,
    3. Volume 41, Issue 7,
    4. July 2009,
    5. Pages 494-499
    6. B. Bortot,
    7. E. Barbi,
    8. S. Biffi,
    9. G. Lunazzi,
    10. G.M. Severini
    11. [Purchase PDF](http://www.sciencedirect.com/science/article/pii/S1590865808007093)
92. [Biliary-colonic fistula caused by cholecystectomy bile duct injury](http://www.sciencedirect.com/science/article/pii/S1499387213600703)
    1. Original research article
    2. Hepatobiliary & Pancreatic Diseases International,
    3. Volume 12, Issue 4,
    4. 15 August 2013,
    5. Pages 443-445
    6. Francisco Igor B Macedo,
    7. Victor J Casillas,
    8. James S Davis,
    9. Joe U Levi,
    10. Danny Sleeman
    11. [Purchase PDF](http://www.sciencedirect.com/science/article/pii/S1499387213600703)
93. [Modifications de l'hémostase au cours de la chirurgie de résection hépatique](http://www.sciencedirect.com/science/article/pii/S0750765800884518)
    1. Original research article
    2. Annales Françaises d'Anesthésie et de Réanimation,
    3. Volume 18, Issue 7,
    4. August 1999,
    5. Pages 711-718
    6. F. Collin,
    7. C. Lehmann,
    8. S. Levy,
    9. P. Bachellier,
    10. A. Steib
    11. [Purchase PDF](http://www.sciencedirect.com/science/article/pii/S0750765800884518)
94. [Treatment of chronic hepatitis C in HIV-positive individuals: Selection of candidates](http://www.sciencedirect.com/science/article/pii/S0168827805007348)
    1. Original research article
    2. Journal of Hepatology,
    3. Volume 44, Supplement 1,
    4. 2006,
    5. Pages s44-s48
    6. Vincent Soriano
    7. [Purchase PDF](http://www.sciencedirect.com/science/article/pii/S0168827805007348)
95. [ACID MALTASE DEFICIENCY AND RELATED MYOPATHIES](http://www.sciencedirect.com/science/article/pii/S0733861905701821)
    1. Original research article
    2. Neurologic Clinics,
    3. Volume 18, Issue 1,
    4. 1 February 2000,
    5. Pages 151-165
    6. Anthony A. Amato
    7. [Purchase PDF](http://www.sciencedirect.com/science/article/pii/S0733861905701821)
96. [Symptômes cliniques ou biologiques devant amener à rechercher une amylose](http://www.sciencedirect.com/science/article/pii/S0248866300882454)
    1. Original research article
    2. La Revue de Médecine Interne,
    3. Volume 21, Issue 2,
    4. February 2000,
    5. Pages 161-166
    6. H. Roussel,
    7. C. Sauron,
    8. R. Barouky
    9. [Purchase PDF](http://www.sciencedirect.com/science/article/pii/S0248866300882454)
97. [Use of recombinant FVIIa for intraperitoneal coagulopathic bleeding in a septic patient](http://www.sciencedirect.com/science/article/pii/S0149794402006906)
    1. Original research article
    2. Current Surgery,
    3. Volume 60, Issue 4,
    4. July–August 2003,
    5. Pages 423-427
    6. John B Holcomb,
    7. Holly L Neville,
    8. Craig F Fischer,
    9. Keith Hoots
    10. [Purchase PDF](http://www.sciencedirect.com/science/article/pii/S0149794402006906)
98. [Apport de la thromboélastométrie rotative (Rotem®) pour l'exploration de l'hémostase: Intérêt en pratique clinique](http://www.sciencedirect.com/science/article/pii/S1773035X07802644)
    1. Original research article
    2. Revue Francophone des Laboratoires,
    3. Volume 2007, Issue 393,
    4. June 2007,
    5. Pages 45-50
    6. Anne Bauters,
    7. Elisabeth Mazoyer
    8. [Purchase PDF](http://www.sciencedirect.com/science/article/pii/S1773035X07802644)
99. [25](http://www.sciencedirect.com/search?qs=%22Liver%20transplantation%22%20and%20FIB-4&show=25&sortBy=relevance&articleTypes=OR)
100. [50](http://www.sciencedirect.com/search?qs=%22Liver%20transplantation%22%20and%20FIB-4&show=50&sortBy=relevance&articleTypes=OR)
101. 100
102. Page 1 of 2
103. [next](http://www.sciencedirect.com/search?qs=%22Liver%20transplantation%22%20and%20FIB-4&show=100&sortBy=relevance&articleTypes=OR&offset=100)

[Feedback](http://www.sciencedirect.com/search?qs=%22Liver%20transplantation%22%20and%20FIB-4&show=100&sortBy=relevance&articleTypes=OR&lastSelectedFacet=articleTypes)

[About ScienceDirect](http://www.elsevier.com/solutions/sciencedirect)[Remote access](http://www.sciencedirect.com/science/activateaccess)[Shopping cart](http://www.sciencedirect.com/science?_ob=ShoppingCartURL&_method=display&_zone=TopNavBar&_origin=srp&md5=e5857d1631c4b1f612897ed029064507)[Contact and support](https://service.elsevier.com/app/contact/supporthub/sciencedirect/)[Terms and conditions](https://www.elsevier.com/legal/elsevier-website-terms-and-conditions)[Privacy policy](http://www.elsevier.com/legal/privacy-policy)

Cookies are used by this site. For more information, visit the [cookies page](https://www.elsevier.com/solutions/sciencedirect/support/cookies).

Copyright © 2017 Elsevier B.V. or its licensors or contributors. ScienceDirect ® is a registered trademark of Elsevier B.V.

[Skip to Main content](http://www.sciencedirect.com/search?qs=%22Liver%20transplantation%22%20and%20FIB-4&show=100&sortBy=relevance&articleTypes=OR&offset=100#main_content)

- [Journals](http://www.sciencedirect.com/science/journals)
- [Books](http://www.sciencedirect.com/science/bookbshsrw)
- [Register](https://www.sciencedirect.com/user/register?returnURL=http%3A%2F%2Fwww.sciencedirect.com%2Fsearch)
- [Sign in](https://www.sciencedirect.com/user/login?returnURL=http%3A%2F%2Fwww.sciencedirect.com%2Fsearch)

Top of Form

[Advanced search](http://www.sciencedirect.com/science/search)

Bottom of Form

137 results

Refine by:

Top of Form

Years

1. 2017 (15)
2. 2016 (15)
3. 2015 (12)
4. 2014 (12)
5. 2013 (8)
6. 2012 (3)
7. 2011 (6)
8. 2010 (4)
9. 2009 (5)
10. 2008 (3)

Show less

Article type

1. Original research (137)

Publication title

1. Journal of Hepatology (12)
2. Transplantation Proceedings (10)
3. Gastroenterology (10)
4. Digestive and Liver Disease (9)
5. Clinical Gastroenterology and Hepatology (8)
6. Journal of Cardiothoracic and Vascular Anesthesia (6)
7. Thrombosis Research (5)
8. The American Journal of Cardiology (3)
9. Human Pathology (3)
10. Transplantation Reviews (3)

Show less

[Clear all filters](http://www.sciencedirect.com/search?qs=%22Liver%20transplantation%22%20and%20FIB-4&show=100&sortBy=relevance&offset=100&navigation=true)

Bottom of Form

sorted by *relevance* | [date](http://www.sciencedirect.com/search?qs=%22Liver%20transplantation%22%20and%20FIB-4&show=100&sortBy=date&articleTypes=OR&offset=100&navigation=true)

1. [Fibrosis in alcoholic and nonalcoholic steatohepatitis](http://www.sciencedirect.com/science/article/pii/S1521691811000369)
   1. Original research article
   2. Best Practice & Research Clinical Gastroenterology,
   3. Volume 25, Issue 2,
   4. April 2011,
   5. Pages 231-244
   6. Ramon Bataller,
   7. Krista Rombouts,
   8. José Altamirano,
   9. Fabio Marra
   10. [Purchase PDF](http://www.sciencedirect.com/science/article/pii/S1521691811000369)
2. [Management of a postpartum coagulopathy using thrombelastography](http://www.sciencedirect.com/science/article/pii/S0952818097000263)
   1. Original research article
   2. Journal of Clinical Anesthesia,
   3. Volume 9, Issue 3,
   4. May 1997,
   5. Pages 243-247
   6. Shiv K. Sharma,
   7. Richard L. Vera,
   8. William C. Stegall,
   9. Charles W. Whitten
   10. [Purchase PDF](http://www.sciencedirect.com/science/article/pii/S0952818097000263)
3. Want a richer search experience?

Sign in for additional filter options, multiple article downloads, and more.

Top of Form

Sign in

Bottom of Form

1. [Establishment of a highly differentiated immortalized human hepatocyte cell line as a source of hepatic function in the bioartificial liver](http://www.sciencedirect.com/science/article/pii/S0041134599009434)
   1. Original research article
   2. Transplantation Proceedings,
   3. Volume 32, Issue 2,
   4. March 2000,
   5. Pages 237-241
   6. N Kobayashi,
   7. M Miyazaki,
   8. K Fukaya,
   9. Y Inoue,
   10. M Namba
   11. [Purchase PDF](http://www.sciencedirect.com/science/article/pii/S0041134599009434)
2. [The contribution of fibrinolysis to postbypass bleeding](http://www.sciencedirect.com/science/article/pii/1053077091900814)
   1. Original research article
   2. Journal of Cardiothoracic and Vascular Anesthesia,
   3. Volume 5, Issue 6, Supplement 1,
   4. December 1991,
   5. Pages 13-17
   6. Bruce D. Spiess
   7. [Purchase PDF](http://www.sciencedirect.com/science/article/pii/1053077091900814)
3. [Vitamin E and vitamin C treatment improves fibrosis in patients with nonalcoholic steatohepatitis](http://www.sciencedirect.com/science/article/pii/S0002927003017866)
   1. Original research article
   2. The American Journal of Gastroenterology,
   3. Volume 98, Issue 11,
   4. November 2003,
   5. Pages 2485-2490
   6. Stephen A Harrison,
   7. Sigurd Torgerson,
   8. Paul Hayashi,
   9. John Ward,
   10. Steven Schenker
   11. [Purchase PDF](http://www.sciencedirect.com/science/article/pii/S0002927003017866)
4. [Detection and clinical significance of red cell aggregation in the human subcutaneous vein using a high-frequency transducer (10 MHz): A preliminary report](http://www.sciencedirect.com/science/article/pii/S030156299700015X)
   1. Original research article
   2. Ultrasound in Medicine & Biology,
   3. Volume 23, Issue 6,
   4. 1997,
   5. Pages 933-938
   6. Hiroshi Kitamura,
   7. Seiji Kawasaki
   8. [Purchase PDF](http://www.sciencedirect.com/science/article/pii/S030156299700015X)
5. [Adrenaline plus cyanoacrylate injection for treatment of bleeding peptic ulcers after failure of conventional endoscopic haemostasis](http://www.sciencedirect.com/science/article/pii/S1590865802801293)
   1. Original research article
   2. Digestive and Liver Disease,
   3. Volume 34, Issue 5,
   4. May 2002,
   5. Pages 349-355
   6. A. Repici,
   7. A. Ferrari,
   8. C. De Angelis,
   9. S. Caronna,
   10. G. Saracco
   11. [Purchase PDF](http://www.sciencedirect.com/science/article/pii/S1590865802801293)
6. [The surgery of “correctable” biliary atresia](http://www.sciencedirect.com/science/article/pii/S0022346887802129)
   1. Original research article
   2. Journal of Pediatric Surgery,
   3. Volume 22, Issue 6,
   4. June 1987,
   5. Pages 522-525
   6. John R. Lilly,
   7. Roberta J. Hall,
   8. Juan Vasquez-Estevez,
   9. Frederick Karrer,
   10. Robert H. Shikes
   11. [Purchase PDF](http://www.sciencedirect.com/science/article/pii/S0022346887802129)
7. [Les acides biliaires et leur utilisation thérapeutique chez l'enfant](http://www.sciencedirect.com/science/article/pii/0929693X96899234)
   1. Original research article
   2. Archives de Pédiatrie,
   3. Volume 2, Issue 12,
   4. December 1995,
   5. Pages 1200-1208
   6. F Cacaille
   7. [Purchase PDF](http://www.sciencedirect.com/science/article/pii/0929693X96899234)
8. [Hepatitis C Virus: A Critical Appraisal of Approaches to Therapy](http://www.sciencedirect.com/science/article/pii/S1542356508011609)
   1. Original research article
   2. Clinical Gastroenterology and Hepatology,
   3. Volume 7, Issue 4,
   4. April 2009,
   5. Pages 397-414
   6. David R. Nelson,
   7. Gary L. Davis,
   8. Ira Jacobson,
   9. Gregory T. Everson,
   10. Nizar Zein
   11. [Purchase PDF](http://www.sciencedirect.com/science/article/pii/S1542356508011609)
9. [Morphometric Differences in Pulmonary Lesions in Primary and Secondary ARDS: A Preliminary Study in Autopsies](http://www.sciencedirect.com/science/article/pii/S0344033804701223)
   1. Original research article
   2. Pathology - Research and Practice,
   3. Volume 197, Issue 8,
   4. 2001,
   5. Pages 521-530
   6. Cristiane Hoelz,
   7. Elnara M. Negri,
   8. Ana J.F.C. Lichtenfels,
   9. Gleice M.S. Conceição,
   10. Vera L. Capelozzi
   11. [Purchase PDF](http://www.sciencedirect.com/science/article/pii/S0344033804701223)
10. [High haematocrit in cyanotic congenital heart disease affects how fibrinogen activity is determined by rotational thromboelastometry](http://www.sciencedirect.com/science/article/pii/S0049384813002843)
    1. Original research article
    2. Thrombosis Research,
    3. Volume 132, Issue 2,
    4. August 2013,
    5. Pages e145-e151
    6. Sarah K. Westbury,
    7. Kurtis Lee,
    8. Christopher Reilly-Stitt,
    9. Robert Tulloh,
    10. Andrew D. Mumford
    11. [Purchase PDF](http://www.sciencedirect.com/science/article/pii/S0049384813002843)
11. [Postoperative changes in coagulant and anticoagulant factors following abdominal aortic surgery](http://www.sciencedirect.com/science/article/pii/1053077092900518)
    1. Original research article
    2. Journal of Cardiothoracic and Vascular Anesthesia,
    3. Volume 6, Issue 6,
    4. December 1992,
    5. Pages 680-685
    6. Neville M. Gibbs,
    7. G. Patrick,
    8. M. Crawford,
    9. Nick Michalopoulos
    10. [Purchase PDF](http://www.sciencedirect.com/science/article/pii/1053077092900518)
12. [Utilidad del Fibroscan® para evaluar la fibrosis hepática](http://www.sciencedirect.com/science/article/pii/S0210570509003562)
    1. Original research article
    2. Gastroenterología y Hepatología,
    3. Volume 32, Issue 6,
    4. June–July 2009,
    5. Pages 415-423
    6. José A. Carrión
    7. [Purchase PDF](http://www.sciencedirect.com/science/article/pii/S0210570509003562)
13. [Comparison of thromboelastography to bleeding time and standard coagulation tests in patients after cardiopulmonary bypass](http://www.sciencedirect.com/science/article/pii/105307709390161D)
    1. Original research article
    2. Journal of Cardiothoracic and Vascular Anesthesia,
    3. Volume 7, Issue 4,
    4. August 1993,
    5. Pages 410-415
    6. James H. Essell,
    7. Thomas J. Martin,
    8. John Salinas,
    9. James M. Thompson,
    10. Vernon C. Smith
    11. [Purchase PDF](http://www.sciencedirect.com/science/article/pii/105307709390161D)
14. [Hemochromatosis and alcoholic liver disease](http://www.sciencedirect.com/science/article/pii/S0741832903001289)
    1. Original research article
    2. Alcohol,
    3. Volume 30, Issue 2,
    4. June 2003,
    5. Pages 131-136
    6. Linda M Fletcher,
    7. Lawrie W Powell
    8. [Purchase PDF](http://www.sciencedirect.com/science/article/pii/S0741832903001289)
15. [Thromboelastometry-guided administration of fibrinogen concentrate for the treatment of excessive intraoperative bleeding in thoracoabdominal aortic aneurysm surgery](http://www.sciencedirect.com/science/article/pii/S002252230900350X)
    1. Open archive,
    2. Original research article
    3. The Journal of Thoracic and Cardiovascular Surgery,
    4. Volume 138, Issue 3,
    5. September 2009,
    6. Pages 694-702
    7. Niels Rahe-Meyer,
    8. Cristina Solomon,
    9. Michael Winterhalter,
    10. Siegfried Piepenbrock,
    11. Maximilian Pichlmaier
    12. [Download PDF (225 KB)](http://www.sciencedirect.com/science/article/pii/S002252230900350X/pdfft?md5=7b6a3db29b88590bee12712b91375822&pid=1-s2.0-S002252230900350X-main.pdf)
16. [Recombinant α-interferon treatment of non-A, non-B (type C) hepatitis: review of studies and recommendations for treatment](http://www.sciencedirect.com/science/article/pii/016882789090168Q)
    1. Original research article
    2. Journal of Hepatology,
    3. Volume 11, Supplement 1,
    4. 1990,
    5. Pages s72-s77
    6. G.L. Davis
    7. [Purchase PDF](http://www.sciencedirect.com/science/article/pii/016882789090168Q)
17. [Comparative effects of an erythrocyte enriched synthetic replenisher and whole blood on streptokinase activity in the isolated perfused liver](http://www.sciencedirect.com/science/article/pii/0049384879900318)
    1. Original research article
    2. Thrombosis Research,
    3. Volume 14, Issue 1,
    4. January 1979,
    5. Pages 125-130
    6. R. Losito,
    7. H. Gattiker,
    8. E. Lemieux,
    9. B. Longpré
    10. [Purchase PDF](http://www.sciencedirect.com/science/article/pii/0049384879900318)
18. [Human hepatocytes express the gene for type 1 plasminogen activator-inhibitor (PAI-1) in vivo](http://www.sciencedirect.com/science/article/pii/S0268949908800410)
    1. Original research article
    2. Fibrinolysis,
    3. Volume 9, Issue 1,
    4. January 1995,
    5. Pages 9-15
    6. A.J. Thornton,
    7. T.D. Gelehrter
    8. [Purchase PDF](http://www.sciencedirect.com/science/article/pii/S0268949908800410)
19. [Prognostic indicators of successful endoscopic sclerotherapy for prevention of rebleeding from oesophageal varices in cirrhosis: a long-term cohort study](http://www.sciencedirect.com/science/article/pii/S1590865800803564)
    1. Original research article
    2. Digestive and Liver Disease,
    3. Volume 32, Issue 9,
    4. December 2000,
    5. Pages 782-791
    6. S. Madonia,
    7. G. D'Amico,
    8. M. Traina,
    9. G. Gatto,
    10. L. Pagliaro
    11. [Purchase PDF](http://www.sciencedirect.com/science/article/pii/S1590865800803564)
20. [Mycophenolate mofetil (RS-61443): Mechanisms of action and effects in transplantation](http://www.sciencedirect.com/science/article/pii/S0955470X05800126)
    1. Original research article
    2. Transplantation Reviews,
    3. Volume 7, Issue 3,
    4. July 1993,
    5. Pages 129-139
    6. Anthony C. Allison,
    7. Elsie M. Eugui,
    8. Hans W. Sollinger
    9. [Purchase PDF](http://www.sciencedirect.com/science/article/pii/S0955470X05800126)
21. [Safety of dipyridamole testing in 73,806 patients: The Multicenter Dipyridamole Safety Study](http://www.sciencedirect.com/science/article/pii/S1071358105800030)
    1. Original research article
    2. Journal of Nuclear Cardiology,
    3. Volume 2, Issue 1,
    4. January–February 1995,
    5. Pages 3-17
    6. Jean Lette,
    7. James L. Tatum,
    8. Sheila Fraser,
    9. D. Douglas Miller,
    10. Multicenter Dipyridamole Safety Study Investigators
    11. [Purchase PDF](http://www.sciencedirect.com/science/article/pii/S1071358105800030)
22. [Mycophenolate mofetil and its mechanisms of action](http://www.sciencedirect.com/science/article/pii/S0162310900001880)
    1. Original research article
    2. Immunopharmacology,
    3. Volume 47, Issues 2–3,
    4. May 2000,
    5. Pages 85-118
    6. Anthony C Allison,
    7. Elsie M Eugui
    8. [Purchase PDF](http://www.sciencedirect.com/science/article/pii/S0162310900001880)
23. [Lymphocyte migration and tissue positioning in allograft reciepients: The role played by extracellular matrix proteins](http://www.sciencedirect.com/science/article/pii/S0955470X9580013X)
    1. Original research article
    2. Transplantation Reviews,
    3. Volume 9, Issue 1,
    4. January 1995,
    5. Pages 29-40
    6. Jerzy W. Kupiec-Weglinski,
    7. Ana J. Coito,
    8. Andrej Gorski,
    9. Maria de Sousa
    10. [Purchase PDF](http://www.sciencedirect.com/science/article/pii/S0955470X9580013X)
24. [Thromboelastogram fails to predict postoperative hemorrhage in cardiac patients](http://www.sciencedirect.com/science/article/pii/0003497592902645)
    1. Original research article
    2. The Annals of Thoracic Surgery,
    3. Volume 53, Issue 3,
    4. March 1992,
    5. Pages 435-439
    6. Jian-Sheng Wang,
    7. Chung-Yuan Lin,
    8. Wei-Te Hung,
    9. Michael F. O'Connor,
    10. Ming-Wen Yang
    11. [Purchase PDF](http://www.sciencedirect.com/science/article/pii/0003497592902645)
25. [B-cell lymphoproliferative disorders in solid-organ transplant patients: Detection of Epstein-Barr virus by in situ hybridization](http://www.sciencedirect.com/science/article/pii/004681779290237W)
    1. Original research article
    2. Human Pathology,
    3. Volume 23, Issue 2,
    4. February 1992,
    5. Pages 159-163
    6. Laurence C. Berg,
    7. Cedith M. Copenhaver,
    8. Vicki A. Morrison,
    9. Scott A. Gruber,
    10. John G. Strickler
    11. [Purchase PDF](http://www.sciencedirect.com/science/article/pii/004681779290237W)
26. [Management approaches to platelet-related microvascular bleeding in cardiothoracic surgery](http://www.sciencedirect.com/science/article/pii/S0003497500016040)
    1. Original research article
    2. The Annals of Thoracic Surgery,
    3. Volume 70, Issue 2, Supplement 1,
    4. August 2000,
    5. Pages s20-s32
    6. George J Despotis,
    7. Lawrence T Goodnough
    8. [Purchase PDF](http://www.sciencedirect.com/science/article/pii/S0003497500016040)
27. [Differential inhibition of individual human liver cytochromes P-450 by cimetidine](http://www.sciencedirect.com/science/article/pii/001650859190408D)
    1. Original research article
    2. Gastroenterology,
    3. Volume 101, Issue 6,
    4. December 1991,
    5. Pages 1680-1691
    6. Robert G. Knodell,
    7. David G. Browne,
    8. Glenn P. Gwozdz,
    9. Wiliam R. Brian,
    10. F.Peter Guengerich
    11. [Purchase PDF](http://www.sciencedirect.com/science/article/pii/001650859190408D)
28. [Weekly treatment of diet/drug-resistant hypercholesterolemia with the heparin-induced extracorporeal low-density lipoprotein precipitation (HELP) system by selective plasma low-density lipoprotein removal](http://www.sciencedirect.com/science/article/pii/0002914993908306)
    1. Original research article
    2. The American Journal of Cardiology,
    3. Volume 71, Issue 10,
    4. 1 April 1993,
    5. Pages 816-822
    6. Daniel M. Lane,
    7. Walter J. McConathy,
    8. L.O. Laughlin,
    9. Philip C. Comp,
    10. Catharine Dorrier
    11. [Purchase PDF](http://www.sciencedirect.com/science/article/pii/0002914993908306)
29. [Disturbo dell’emostasi in pronto soccorso](http://www.sciencedirect.com/science/article/pii/S1286934110700107)
    1. Original research article
    2. EMC - Urgenze,
    3. Volume 14, Issue 1,
    4. 2010,
    5. Pages 1-22
    6. N. Nathan,
    7. A. Julia
    8. [Purchase PDF](http://www.sciencedirect.com/science/article/pii/S1286934110700107)
30. [Haemostyptic preparations on the basis of collagen alone and as fixed combination with fibrin glue](http://www.sciencedirect.com/science/article/pii/026766059290097D)
    1. Original research article
    2. Clinical Materials,
    3. Volume 9, Issues 3–4,
    4. 1992,
    5. Pages 169-177
    6. Ulrich Schiele,
    7. Günter Kuntz,
    8. Anita Riegler
    9. [Purchase PDF](http://www.sciencedirect.com/science/article/pii/026766059290097D)
31. [Secondary osteoporosis](http://www.sciencedirect.com/science/article/pii/S0950351X97805216)
    1. Original research article
    2. Baillière's Clinical Endocrinology and Metabolism,
    3. Volume 11, Issue 1,
    4. April 1997,
    5. Pages 83-99
    6. David M. Reid,
    7. John Harvie
    8. [Purchase PDF](http://www.sciencedirect.com/science/article/pii/S0950351X97805216)
32. [Emerging and underrecognized respiratory infections in children](http://www.sciencedirect.com/science/article/pii/S1045187005800482)
    1. Original research article
    2. Seminars in Pediatric Infectious Diseases,
    3. Volume 6, Issue 3,
    4. July 1995,
    5. Pages 201-208
    6. Jay C. Butler
    7. [Purchase PDF](http://www.sciencedirect.com/science/article/pii/S1045187005800482)
33. [Thérapeutique des mycoses profondes (à l'exception des aspergilloses et des fusarioses)](http://www.sciencedirect.com/science/article/pii/S0338989801801244)
    1. Original research article
    2. Revue Française des Laboratoires,
    3. Volume 2001, Issue 332,
    4. April 2001,
    5. Pages 23-30
    6. Sophie Léautez,
    7. François Raffi
    8. [Purchase PDF](http://www.sciencedirect.com/science/article/pii/S0338989801801244)
34. [Enzymes as agents for the treatment of disease](http://www.sciencedirect.com/science/article/pii/000989819290007D)
    1. Original research article
    2. Clinica Chimica Acta,
    3. Volume 206, Issues 1–2,
    4. 13 March 1992,
    5. Pages 45-76
    6. David M. Goldberg
    7. [Purchase PDF](http://www.sciencedirect.com/science/article/pii/000989819290007D)
35. [Optimal Management of Bleeding and Transfusion in Patients Undergoing Cardiac Surgery](http://www.sciencedirect.com/science/article/pii/S104306799970002X)
    1. Original research article
    2. Seminars in Thoracic and Cardiovascular Surgery,
    3. Volume 11, Issue 2,
    4. April 1999,
    5. Pages 84-104
    6. George J. Despotis,
    7. Nikolaos J. Skubas,
    8. Lawrence Tim Goodnough
    9. [Purchase PDF](http://www.sciencedirect.com/science/article/pii/S104306799970002X)
36. [25](http://www.sciencedirect.com/search?qs=%22Liver%20transplantation%22%20and%20FIB-4&show=25&sortBy=relevance&articleTypes=OR&navigation=true)
37. [50](http://www.sciencedirect.com/search?qs=%22Liver%20transplantation%22%20and%20FIB-4&show=50&sortBy=relevance&articleTypes=OR&navigation=true)
38. 100
39. [previous](http://www.sciencedirect.com/search?qs=%22Liver%20transplantation%22%20and%20FIB-4&show=100&sortBy=relevance&articleTypes=OR&offset=0&navigation=true)
40. Page 2 of 2

[Feedback](http://www.sciencedirect.com/search?qs=%22Liver%20transplantation%22%20and%20FIB-4&show=100&sortBy=relevance&articleTypes=OR&offset=100)

[About ScienceDirect](http://www.elsevier.com/solutions/sciencedirect)[Remote access](http://www.sciencedirect.com/science/activateaccess)[Shopping cart](http://www.sciencedirect.com/science?_ob=ShoppingCartURL&_method=display&_zone=TopNavBar&_origin=srp&md5=e5857d1631c4b1f612897ed029064507)[Contact and support](https://service.elsevier.com/app/contact/supporthub/sciencedirect/)[Terms and conditions](https://www.elsevier.com/legal/elsevier-website-terms-and-conditions)[Privacy policy](http://www.elsevier.com/legal/privacy-policy)

Cookies are used by this site. For more information, visit the [cookies page](https://www.elsevier.com/solutions/sciencedirect/support/cookies).

Copyright © 2017 Elsevier B.V. or its licensors or contributors. ScienceDirect ® is a registered trademark of Elsevier B.V.

Transient elastography

[Skip to Main content](http://www.sciencedirect.com/search?qs=%22Liver%20transplantation%22%20and%20%22transient%20elastography%22&show=100&sortBy=relevance&lastSelectedFacet=articleTypes&articleTypes=OR#main_content)

- [Journals](http://www.sciencedirect.com/science/journals)
- [Books](http://www.sciencedirect.com/science/bookbshsrw)
- [Register](https://www.sciencedirect.com/user/register?returnURL=http%3A%2F%2Fwww.sciencedirect.com%2Fsearch)
- [Sign in](https://www.sciencedirect.com/user/login?returnURL=http%3A%2F%2Fwww.sciencedirect.com%2Fsearch)

Top of Form

[Advanced search](http://www.sciencedirect.com/science/search)

Bottom of Form

252 results

Refine by:

Top of Form

Years

1. 2017 (35)
2. 2016 (48)
3. 2015 (36)
4. 2014 (23)
5. 2013 (15)
6. 2012 (28)
7. 2011 (18)
8. 2010 (14)
9. 2009 (13)
10. 2008 (15)

Show less

Article type

1. Review articles (172)
2. Original research (252)
3. Encyclopedia (2)
4. Book chapters (60)
5. Other (636)

Show less

Publication title

1. Journal of Hepatology (53)
2. Clinical Gastroenterology and Hepatology (24)
3. Gastroenterology (22)
4. Digestive and Liver Disease (19)
5. Transplantation Proceedings (9)
6. Ultrasound in Medicine & Biology (8)
7. European Journal of Radiology (6)
8. Metabolism (6)
9. Best Practice & Research Clinical Gastroenterology (6)
10. Gastroenterología y Hepatología (5)

Show less

[Clear all filters](http://www.sciencedirect.com/search?qs=%22Liver%20transplantation%22%20and%20%22transient%20elastography%22&show=100&sortBy=relevance)

Bottom of Form

sorted by *relevance* | [date](http://www.sciencedirect.com/search?qs=%22Liver%20transplantation%22%20and%20%22transient%20elastography%22&show=100&sortBy=date&articleTypes=OR)

1. [Comparison of 2-D Shear Wave Elastography and Transient Elastography for Assessing Liver Fibrosis in Chronic Hepatitis B](http://www.sciencedirect.com/science/article/pii/S0301562917301369)
   1. Original research article
   2. Ultrasound in Medicine & Biology,
   3. Volume 43, Issue 8,
   4. August 2017,
   5. Pages 1563-1570
   6. Jie Zeng,
   7. Jian Zheng,
   8. Zeping Huang,
   9. Shigao Chen,
   10. Mingde Lu
   11. [Purchase PDF](http://www.sciencedirect.com/science/article/pii/S0301562917301369)
2. [Transient elastography in autoimmune hepatitis: Timing determines the impact of inflammation and fibrosis](http://www.sciencedirect.com/science/article/pii/S0168827816302094)
   1. Original research article
   2. Journal of Hepatology,
   3. Volume 65, Issue 4,
   4. October 2016,
   5. Pages 769-775
   6. Johannes Hartl,
   7. Ulrike Denzer,
   8. Hanno Ehlken,
   9. Roman Zenouzi,
   10. Christoph Schramm
   11. [Purchase PDF](http://www.sciencedirect.com/science/article/pii/S0168827816302094)
3. Want a richer search experience?

Sign in for additional filter options, multiple article downloads, and more.

Top of Form

Sign in

Bottom of Form

1. [Non-invasive assessment of liver graft fibrosis by transient elastography after liver transplantation](http://www.sciencedirect.com/science/article/pii/S2210740112003269)
   1. Original research article
   2. Clinics and Research in Hepatology and Gastroenterology,
   3. Volume 37, Issue 4,
   4. September 2013,
   5. Pages 347-352
   6. Camille Barrault,
   7. Françoise Roudot-Thoraval,
   8. Jeanne Tran Van Nhieu,
   9. Calina Atanasiu,
   10. Christophe Duvoux
   11. [Purchase PDF](http://www.sciencedirect.com/science/article/pii/S2210740112003269)
2. [Graft Function Measured by Transient Elastography in Living Donor Liver Transplantation: Preliminary](http://www.sciencedirect.com/science/article/pii/S004113451300794X)
   1. Original research article
   2. Transplantation Proceedings,
   3. Volume 45, Issue 8,
   4. October 2013,
   5. Pages 3028-3031
   6. S.H. Lee,
   7. D.J. Joo,
   8. S.U. Kim,
   9. M.S. Kim,
   10. S.I. Kim
   11. [Purchase PDF](http://www.sciencedirect.com/science/article/pii/S004113451300794X)
3. [Longitudinal Transient Elastography Measurements Used in Follow-up for Patients with Cystic Fibrosis](http://www.sciencedirect.com/science/article/pii/S0301562915006754)
   1. Original research article
   2. Ultrasound in Medicine & Biology,
   3. Volume 42, Issue 4,
   4. April 2016,
   5. Pages 848-854
   6. Stephanie Van Biervliet,
   7. Hugo Verdievel,
   8. Saskia Vande Velde,
   9. Ruth De Bruyne,
   10. Hans Van Vlierberghe
   11. [Purchase PDF](http://www.sciencedirect.com/science/article/pii/S0301562915006754)
4. [Monitoring the Natural Evolution and Response to Treatment of Post Liver Transplant Recurrent Hepatitis C Using Transient Elastography: Preliminary Results](http://www.sciencedirect.com/science/article/pii/S0041134512007889)
   1. Original research article
   2. Transplantation Proceedings,
   3. Volume 44, Issue 7,
   4. September 2012,
   5. Pages 2082-2086
   6. F. Bellido-Muñoz,
   7. A. Giráldez-Gallego,
   8. C. Roca-Oporto,
   9. T. García-Cayuela,
   10. J.M. Sousa-Martín
   11. [Purchase PDF](http://www.sciencedirect.com/science/article/pii/S0041134512007889)
5. [The Most Frequently Cited 100 Articles in Liver Transplantation Literature](http://www.sciencedirect.com/science/article/pii/S0041134517300520)
   1. Original research article
   2. Transplantation Proceedings,
   3. Volume 49, Issue 3,
   4. April 2017,
   5. Pages 551-561
   6. M. Özbilgin,
   7. T. Ünek,
   8. T. Egeli,
   9. C. Ağalar,
   10. İ. Astarcıoğlu
   11. [Purchase PDF](http://www.sciencedirect.com/science/article/pii/S0041134517300520)
6. [Liver stiffness measurement by transient elastography predicts late posthepatectomy outcomes in patients undergoing resection for hepatocellular carcinoma](http://www.sciencedirect.com/science/article/pii/S0039606017303963)
   1. Original research article
   2. Surgery,
   3. In press, corrected proof,
   4. Available online 12 July 2017
   5. Muthukumarassamy Rajakannu,
   6. Daniel Cherqui,
   7. Oriana Ciacio,
   8. Nicolas Golse,
   9. Eric Vibert
   10. [Purchase PDF](http://www.sciencedirect.com/science/article/pii/S0039606017303963)
7. [Efficacy and Safety of Therapy With Simeprevir and Sofosbuvir in Liver Transplant Recipients Infected by Hepatitis C Virus Genotype 4: Cohort Spanish Society of Liver Transplantation Cohort](http://www.sciencedirect.com/science/article/pii/S0041134516305309)
   1. Original research article
   2. Transplantation Proceedings,
   3. Volume 48, Issue 9,
   4. November 2016,
   5. Pages 3013-3016
   6. G. Sanchez Antolin,
   7. M. Testillano,
   8. J.M. Pascasio,
   9. I. Narvaez Rodriguez,
   10. Spanish Society of Liver Transplantation Study Group Collaborating Group
   11. [Purchase PDF](http://www.sciencedirect.com/science/article/pii/S0041134516305309)
8. [Assessment of liver fibrosis in transplant recipients with recurrent HCV infection: Usefulness of transientelastography](http://www.sciencedirect.com/science/article/pii/S1590865808002533)
   1. Original research article
   2. Digestive and Liver Disease,
   3. Volume 41, Issue 3,
   4. March 2009,
   5. Pages 217-225
   6. F. Corradi,
   7. F. Piscaglia,
   8. S. Flori,
   9. A. D’Errico-Grigioni,
   10. The Bologna Liver Transplantation Group (BLTG)
   11. [Purchase PDF](http://www.sciencedirect.com/science/article/pii/S1590865808002533)
9. [Liver and spleen transient elastography and Acoustic Radiation Force Impulse Measurements. Performance and comparison of measurements in the same area concurrently assessed for liver fibrosis by biopsy](http://www.sciencedirect.com/science/article/pii/S1896112615000334)
   1. Original research article
   2. Advances in Medical Sciences,
   3. Volume 60, Issue 2,
   4. September 2015,
   5. Pages 300-306
   6. Francesca M. Trovato,
   7. Sebastiana Atzori,
   8. Giuseppe Musumeci,
   9. Vanessa Tooley,
   10. Simon D. Taylor-Robinson
   11. [Purchase PDF](http://www.sciencedirect.com/science/article/pii/S1896112615000334)
10. [FibroScan (Vibration-Controlled Transient Elastography): Where Does It Stand in the United States Practice](http://www.sciencedirect.com/science/article/pii/S1542356514008180)
    1. Original research article
    2. Clinical Gastroenterology and Hepatology,
    3. Volume 13, Issue 1,
    4. January 2015,
    5. Pages 27-36
    6. Elliot B. Tapper,
    7. Laurent Castera,
    8. Nezam H. Afdhal
    9. [Purchase PDF](http://www.sciencedirect.com/science/article/pii/S1542356514008180)
11. [Performance and utility of transient elastography and noninvasive markers of liver fibrosis in primary biliary cirrhosis](http://www.sciencedirect.com/science/article/pii/S1590865811002258)
    1. Original research article
    2. Digestive and Liver Disease,
    3. Volume 43, Issue 11,
    4. November 2011,
    5. Pages 887-892
    6. Annarosa Floreani,
    7. Nora Cazzagon,
    8. Diego Martines,
    9. Luisa Cavalletto,
    10. Liliana Chemello
    11. [Purchase PDF](http://www.sciencedirect.com/science/article/pii/S1590865811002258)
12. [Predictors of Poor Prognosis in Recurrent Hepatitis C After Liver Transplantation](http://www.sciencedirect.com/science/article/pii/S0041134516305760)
    1. Original research article
    2. Transplantation Proceedings,
    3. Volume 48, Issue 9,
    4. November 2016,
    5. Pages 2997-2999
    6. E. Berge,
    7. E. Otón,
    8. Z. Reina,
    9. L. Díaz,
    10. F. Pérez
    11. [Purchase PDF](http://www.sciencedirect.com/science/article/pii/S0041134516305760)
13. [Non-alcoholic fatty liver disease and liver transplantation](http://www.sciencedirect.com/science/article/pii/S0026049516000524)
    1. Original research article
    2. Metabolism,
    3. Volume 65, Issue 8,
    4. August 2016,
    5. Pages 1208-1223
    6. Reenam S. Khan,
    7. Philip N. Newsome
    8. [Purchase PDF](http://www.sciencedirect.com/science/article/pii/S0026049516000524)
14. [Changes in Liver Congestion in Patients with Budd–Chiari Syndrome following Endovascular Interventions: Assessment with Transient Elastography](http://www.sciencedirect.com/science/article/pii/S1051044316308910)
    1. Original research article
    2. Journal of Vascular and Interventional Radiology,
    3. Volume 28, Issue 5,
    4. May 2017,
    5. Pages 683-687
    6. Amar Mukund,
    7. Sudheer S. Pargewar,
    8. Saloni N. Desai,
    9. S. Rajesh,
    10. Shiv K. Sarin
    11. [Purchase PDF](http://www.sciencedirect.com/science/article/pii/S1051044316308910)
15. [Sofosbuvir and Ribavirin for 24 Weeks Is An Effective Treatment Option for Recurrent Hepatitis C Infection After Living Donor Liver Transplantation](http://www.sciencedirect.com/science/article/pii/S097368831730004X)
    1. Original research article
    2. Journal of Clinical and Experimental Hepatology,
    3. In press, corrected proof,
    4. Available online 28 June 2017
    5. Anil C. Anand,
    6. Shaleen K. Agarwal,
    7. Hitendra K. Garg,
    8. Sudeep Khanna,
    9. Subhash Gupta
    10. [Purchase PDF](http://www.sciencedirect.com/science/article/pii/S097368831730004X)
16. [Factors associated with significant liver steatosis and fibrosis as assessed by transient elastography in patients with one or more components of the metabolic syndrome](http://www.sciencedirect.com/science/article/pii/S1056872716301672)
    1. Original research article
    2. Journal of Diabetes and its Complications,
    3. Volume 30, Issue 7,
    4. September–October 2016,
    5. Pages 1347-1353
    6. Ivana Mikolasevic,
    7. Sandra Milic,
    8. Lidija Orlic,
    9. Davor Stimac,
    10. Giovanni Targher
    11. [Purchase PDF](http://www.sciencedirect.com/science/article/pii/S1056872716301672)
17. [Transient elastography (FibroScan)](http://www.sciencedirect.com/science/article/pii/S0399832008739940)
    1. Original research article
    2. Gastroentérologie Clinique et Biologique,
    3. Volume 32, Issue 6, Supplement 1,
    4. September 2008,
    5. Pages 58-67
    6. V. de Lédinghen,
    7. J. Vergniol
    8. [Purchase PDF](http://www.sciencedirect.com/science/article/pii/S0399832008739940)
18. [Does transient elastography (FibroScan®) have a role in decision making in hepatocellular carcinoma?](http://www.sciencedirect.com/science/article/pii/S1365182X15305955)
    1. Open archive,
    2. Original research article
    3. HPB,
    4. Volume 14, Issue 6,
    5. June 2012,
    6. Pages 403-408
    7. Antonio Pesce,
    8. Roberto Scilletta,
    9. Angela Branca,
    10. Luciano Nigro,
    11. Stefano Puleo
    12. [Download PDF (155 KB)](http://www.sciencedirect.com/science/article/pii/S1365182X15305955/pdfft?md5=64dd0187ba44040255452a19247ab2cb&pid=1-s2.0-S1365182X15305955-main.pdf)
19. [Non-invasive evaluation of liver fibrosis using transient elastography](http://www.sciencedirect.com/science/article/pii/S0168827808001232)
    1. Original research article
    2. Journal of Hepatology,
    3. Volume 48, Issue 5,
    4. May 2008,
    5. Pages 835-847
    6. Laurent Castera,
    7. Xavier Forns,
    8. Alfredo Alberti
    9. [Purchase PDF](http://www.sciencedirect.com/science/article/pii/S0168827808001232)
20. [Immune monitoring of immunosuppression withdrawal of liver transplant recipients](http://www.sciencedirect.com/science/article/pii/S0966327415300113)
    1. Original research article
    2. Transplant Immunology,
    3. Volume 33, Issue 2,
    4. October 2015,
    5. Pages 110-116
    6. Rocío García de la Garza,
    7. Pablo Sarobe,
    8. Juana Merino,
    9. Juan J. Lasarte,
    10. J. Ignacio Herrero
    11. [Purchase PDF](http://www.sciencedirect.com/science/article/pii/S0966327415300113)
21. [Serum YKL-40 in young patients with β-thalassemia major: Relation to hepatitis C virus infection, liverstiffness by transient elastography and cardiovascular complications](http://www.sciencedirect.com/science/article/pii/S1079979615001722)
    1. Original research article
    2. Blood Cells, Molecules, and Diseases,
    3. Volume 56, Issue 1,
    4. January 2016,
    5. Pages 1-8
    6. Mohamed Abo El-Asrar,
    7. Nancy Samir Elbarbary,
    8. Eman Abdel Rahman Ismail,
    9. Ahmed Mohamed Elshenity
    10. [Purchase PDF](http://www.sciencedirect.com/science/article/pii/S1079979615001722)
22. [De Novo Donor-Specific Anti–Human Leukocyte Antigen Antibody Detection in Long-Term Adult LiverTransplantation](http://www.sciencedirect.com/science/article/pii/S0041134516305401)
    1. Original research article
    2. Transplantation Proceedings,
    3. Volume 48, Issue 9,
    4. November 2016,
    5. Pages 2980-2982
    6. D. San Segundo,
    7. C. Alonso,
    8. P. Ruiz,
    9. I. Roman,
    10. E. Fábrega
    11. [Purchase PDF](http://www.sciencedirect.com/science/article/pii/S0041134516305401)
23. [The role of transient elastography in patients with hepatitis B viral disease](http://www.sciencedirect.com/science/article/pii/S1590865810606895)
    1. Original research article
    2. Digestive and Liver Disease,
    3. Volume 43, Supplement 1,
    4. January 2011,
    5. Pages s25-s31
    6. Mirella Fraquelli,
    7. Federica Branchi
    8. [Purchase PDF](http://www.sciencedirect.com/science/article/pii/S1590865810606895)
24. [Which are the cut-off values of 2D-Shear Wave Elastography (2D-SWE) liver stiffness measurements predicting different stages of liver fibrosis, considering Transient Elastography (TE) as the reference method?](http://www.sciencedirect.com/science/article/pii/S0720048X13006542)
    1. Original research article
    2. European Journal of Radiology,
    3. Volume 83, Issue 3,
    4. March 2014,
    5. Pages e118-e122
    6. Ioan Sporea,
    7. Simona Bota,
    8. Oana Gradinaru-Taşcău,
    9. Roxana Şirli,
    10. Ana Jurchiş
    11. [Purchase PDF](http://www.sciencedirect.com/science/article/pii/S0720048X13006542)
25. [Diffusion weighted MRI and transient elastography assessment of liver fibrosis in hepatitis C patients: Validity of non invasive imaging techniques](http://www.sciencedirect.com/science/article/pii/S0378603X14000357)
    1. Open access,
    2. Original research article
    3. The Egyptian Journal of Radiology and Nuclear Medicine,
    4. Volume 45, Issue 2,
    5. June 2014,
    6. Pages 279-287
    7. Fatma Zaiton,
    8. Hitham Dawoud,
    9. Inas M. El Fiki,
    10. Khaled M. Hadhoud
    11. [Download PDF (1,234 KB)](http://www.sciencedirect.com/science/article/pii/S0378603X14000357/pdfft?md5=95c68aa1bd1316392a866c0429775938&pid=1-s2.0-S0378603X14000357-main.pdf)
26. [FibroScan Evaluation of Liver Fibrosis in Liver Transplantation](http://www.sciencedirect.com/science/article/pii/S004113450900164X)
    1. Original research article
    2. Transplantation Proceedings,
    3. Volume 41, Issue 3,
    4. April 2009,
    5. Pages 1044-1046
    6. G. Sánchez Antolin,
    7. F. Garcia Pajares,
    8. M.A. Vallecillo,
    9. P. Fernandez Orcajo,
    10. A. Caro-Patón
    11. [Purchase PDF](http://www.sciencedirect.com/science/article/pii/S004113450900164X)
27. [Staging chronic hepatitis C in seven categories using fibrosis biomarker (FibroTest™) and transientelastography (FibroScan®)](http://www.sciencedirect.com/science/article/pii/S0168827813008179)
    1. Original research article
    2. Journal of Hepatology,
    3. Volume 60, Issue 4,
    4. April 2014,
    5. Pages 706-714
    6. Thierry Poynard,
    7. Julien Vergniol,
    8. Yen Ngo,
    9. Juliette Foucher,
    10. Bordeaux HCV Study Group
    11. [Purchase PDF](http://www.sciencedirect.com/science/article/pii/S0168827813008179)
28. [Liver Transplantation for Alcoholic and Nonalcoholic Fatty Liver Disease: Pretransplant Selection and Posttransplant Management](http://www.sciencedirect.com/science/article/pii/S0016508516003036)
    1. Original research article
    2. Gastroenterology,
    3. Volume 150, Issue 8,
    4. June 2016,
    5. Pages 1849-1862
    6. M. Shadab Siddiqui,
    7. Michael Charlton
    8. [Purchase PDF](http://www.sciencedirect.com/science/article/pii/S0016508516003036)
29. [Noninvasive Assessment of Liver Fibrosis and Portal Hypertension With Transient Elastography](http://www.sciencedirect.com/science/article/pii/S0016508507021300)
    1. Original research article
    2. Gastroenterology,
    3. Volume 134, Issue 1,
    4. January 2008,
    5. Pages 8-14
    6. Don C. Rockey
    7. [Purchase PDF](http://www.sciencedirect.com/science/article/pii/S0016508507021300)
30. [Noninvasive assessment of liver fibrosis in patients with Fontan circulation using transient elastography and biochemical fibrosis markers](http://www.sciencedirect.com/science/article/pii/S0022522307016455)
    1. Open archive,
    2. Original research article
    3. The Journal of Thoracic and Cardiovascular Surgery,
    4. Volume 135, Issue 3,
    5. March 2008,
    6. Pages 560-567
    7. Mireen Friedrich-Rust,
    8. Constanze Koch,
    9. Axel Rentzsch,
    10. Christoph Sarrazin,
    11. Hashim Abdul-Khaliq
    12. [Download PDF (306 KB)](http://www.sciencedirect.com/science/article/pii/S0022522307016455/pdfft?md5=312bb4134412c0b353dd3540f537144d&pid=1-s2.0-S0022522307016455-main.pdf)
31. [Diffusion-weighted MRI versus transient elastography in quantification of liver fibrosis in patients with chronic cholestatic liver diseases](http://www.sciencedirect.com/science/article/pii/S0720048X11007674)
    1. Original research article
    2. European Journal of Radiology,
    3. Volume 81, Issue 10,
    4. October 2012,
    5. Pages 2500-2506
    6. Jelena Djokić Kovač,
    7. Marko Daković,
    8. Dejana Stanisavljević,
    9. Tamara Alempijević,
    10. Ružica Maksimović
    11. [Purchase PDF](http://www.sciencedirect.com/science/article/pii/S0720048X11007674)
32. [Discordance in fibrosis staging between liver biopsy and transient elastography using the FibroScan XL probe](http://www.sciencedirect.com/science/article/pii/S0168827811007823)
    1. Original research article
    2. Journal of Hepatology,
    3. Volume 56, Issue 3,
    4. March 2012,
    5. Pages 564-570
    6. Robert P. Myers,
    7. Gilles Pomier-Layrargues,
    8. Richard Kirsch,
    9. Aaron Pollett,
    10. Magdy Elkashab
    11. [Purchase PDF](http://www.sciencedirect.com/science/article/pii/S0168827811007823)
33. [Volatile Biomarkers in Breath Associated With Liver Cirrhosis — Comparisons of Pre- and Post-liverTransplant Breath Samples](http://www.sciencedirect.com/science/article/pii/S2352396415300797)
    1. Open access,
    2. Original research article
    3. EBioMedicine,
    4. Volume 2, Issue 9,
    5. September 2015,
    6. Pages 1243-1250
    7. R. Fernández del Río,
    8. M.E. O'Hara,
    9. A. Holt,
    10. P. Pemberton,
    11. C.A. Mayhew
    12. [Download PDF (450 KB)](http://www.sciencedirect.com/science/article/pii/S2352396415300797/pdfft?md5=b3c09d155d0ff9305ef0eb806e2f91e8&pid=1-s2.0-S2352396415300797-main.pdf)
34. [Ultrasound-Based Transient Elastography for the Detection of Hepatic Fibrosis: Systematic Review and Meta-analysis](http://www.sciencedirect.com/science/article/pii/S1542356507007537)
    1. Original research article
    2. Clinical Gastroenterology and Hepatology,
    3. Volume 5, Issue 10,
    4. October 2007,
    5. Pages 1214-1220
    6. Jayant A. Talwalkar,
    7. David M. Kurtz,
    8. Scott J. Schoenleber,
    9. Colin P. West,
    10. Victor M. Montori
    11. [Purchase PDF](http://www.sciencedirect.com/science/article/pii/S1542356507007537)
35. [Assessment of Fibrosis by Transient Elastography Compared With Liver Biopsy and Morphometry in Chronic Liver Diseases](http://www.sciencedirect.com/science/article/pii/S1542356508002000)
    1. Original research article
    2. Clinical Gastroenterology and Hepatology,
    3. Volume 6, Issue 9,
    4. September 2008,
    5. Pages 1027-1035
    6. Grace Lai–Hung Wong,
    7. Vincent Wai–Sun Wong,
    8. Paul Cheung–Lung Choi,
    9. Anthony Wing–Hung Chan,
    10. Henry Lik–Yuen Chan
    11. [Purchase PDF](http://www.sciencedirect.com/science/article/pii/S1542356508002000)
36. [MRI-guided Biopsy to Correlate Tissue Specimens with MR Elastography Stiffness Readings in LiverTransplants](http://www.sciencedirect.com/science/article/pii/S1076633212003133)
    1. Original research article
    2. Academic Radiology,
    3. Volume 19, Issue 9,
    4. September 2012,
    5. Pages 1121-1126
    6. Ryan B. Perumpail,
    7. Josh Levitsky,
    8. Yi Wang,
    9. Victoria S. Lee,
    10. Reed A. Omary
    11. [Purchase PDF](http://www.sciencedirect.com/science/article/pii/S1076633212003133)
37. [Performance of Transient Elastography for the Staging of Liver Fibrosis: A Meta-Analysis](http://www.sciencedirect.com/science/article/pii/S001650850800108X)
    1. Original research article
    2. Gastroenterology,
    3. Volume 134, Issue 4,
    4. April 2008,
    5. Pages 960-974.e8
    6. Mireen Friedrich–Rust,
    7. Mei–Fang Ong,
    8. Swantje Martens,
    9. Christoph Sarrazin,
    10. Eva Herrmann
    11. [Purchase PDF](http://www.sciencedirect.com/science/article/pii/S001650850800108X)
38. [Controversies in Liver Transplantation for Hepatitis C](http://www.sciencedirect.com/science/article/pii/S0016508508002862)
    1. Original research article
    2. Gastroenterology,
    3. Volume 134, Issue 6,
    4. May 2008,
    5. Pages 1777-1788
    6. Sandeep Mukherjee,
    7. Michael F. Sorrell
    8. [Purchase PDF](http://www.sciencedirect.com/science/article/pii/S0016508508002862)
39. [Hepatic Venous Congestion After Living Donor Liver Transplantation: Quantitative Assessment of LiverStiffness Using Shear Wave Elastography—A Case Report](http://www.sciencedirect.com/science/article/pii/S0041134512000693)
    1. Original research article
    2. Transplantation Proceedings,
    3. Volume 44, Issue 3,
    4. April 2012,
    5. Pages 814-816
    6. H.-K. Wang,
    7. Y.-C. Lai,
    8. H.-S. Tseng,
    9. R.-C. Lee,
    10. C.-Y. Chang
    11. [Purchase PDF](http://www.sciencedirect.com/science/article/pii/S0041134512000693)
40. [Magnetic Resonance Elastography vs Transient Elastography in Detection of Fibrosis and Noninvasive Measurement of Steatosis in Patients With Biopsy-Proven Nonalcoholic Fatty Liver Disease](http://www.sciencedirect.com/science/article/pii/S0016508516352738)
    1. Original research article
    2. Gastroenterology,
    3. Volume 152, Issue 3,
    4. February 2017,
    5. Pages 598-607.e2
    6. Charlie C. Park,
    7. Phirum Nguyen,
    8. Carolyn Hernandez,
    9. Ricki Bettencourt,
    10. Rohit Loomba
    11. [Purchase PDF](http://www.sciencedirect.com/science/article/pii/S0016508516352738)
41. [Viral Hepatitis in Liver Transplantation](http://www.sciencedirect.com/science/article/pii/S0016508512002235)
    1. Original research article
    2. Gastroenterology,
    3. Volume 142, Issue 6,
    4. May 2012,
    5. Pages 1373-1383.e1
    6. Gonzalo Crespo,
    7. Zoe Mariño,
    8. Miquel Navasa,
    9. Xavier Forns
    10. [Purchase PDF](http://www.sciencedirect.com/science/article/pii/S0016508512002235)
42. [The Acoustic Radiation Force Impulse Elastography Evaluation of Liver Fibrosis in Posttransplantation Dysfunction of Living Donor Liver Transplantation](http://www.sciencedirect.com/science/article/pii/S0041134513014085)
    1. Original research article
    2. Transplantation Proceedings,
    3. Volume 46, Issue 3,
    4. April 2014,
    5. Pages 876-879
    6. C.C. Liao,
    7. T.Y. Chen,
    8. L.C. Tsang,
    9. S.Y. Ou,
    10. T.L. Huang
    11. [Purchase PDF](http://www.sciencedirect.com/science/article/pii/S0041134513014085)
43. [Early detection in routine clinical practice of cirrhosis and oesophageal varices in chronic hepatitis C: Comparison of transient elastography (FibroScan) with standard laboratory tests and non-invasive scores](http://www.sciencedirect.com/science/article/pii/S0168827808006387)
    1. Original research article
    2. Journal of Hepatology,
    3. Volume 50, Issue 1,
    4. January 2009,
    5. Pages 59-68
    6. Laurent Castéra,
    7. Brigitte Le Bail,
    8. Françoise Roudot-Thoraval,
    9. Pierre-Henri Bernard,
    10. Victor de Lédinghen
    11. [Purchase PDF](http://www.sciencedirect.com/science/article/pii/S0168827808006387)
44. [Magnetic Resonance Imaging More Accurately Classifies Steatosis and Fibrosis in Patients With Nonalcoholic Fatty Liver Disease Than Transient Elastography](http://www.sciencedirect.com/science/article/pii/S0016508515017345)
    1. Open access,
    2. Original research article
    3. Gastroenterology,
    4. Volume 150, Issue 3,
    5. March 2016,
    6. Pages 626-637.e7
    7. Kento Imajo,
    8. Takaomi Kessoku,
    9. Yasushi Honda,
    10. Wataru Tomeno,
    11. Atsushi Nakajima
    12. [Download PDF (1,580 KB)](http://www.sciencedirect.com/science/article/pii/S0016508515017345/pdfft?md5=3127e601c346bb0639a230236cdacf22&pid=1-s2.0-S0016508515017345-main.pdf)
45. [Association Between Anthropometric Parameters and Measurements of Liver Stiffness by TransientElastography](http://www.sciencedirect.com/science/article/pii/S1542356512011408)
    1. Original research article
    2. Clinical Gastroenterology and Hepatology,
    3. Volume 11, Issue 3,
    4. March 2013,
    5. Pages 295-302.e3
    6. Grace Lai–Hung Wong,
    7. Henry Lik–Yuen Chan,
    8. Paul Cheung–Lung Choi,
    9. Anthony Wing–Hung Chan,
    10. Vincent Wai–Sun Wong
    11. [Purchase PDF](http://www.sciencedirect.com/science/article/pii/S1542356512011408)
46. [Association Between Level of Fibrosis, Rather Than Antiviral Regimen, and Outcomes of Patients With Chronic Hepatitis B](http://www.sciencedirect.com/science/article/pii/S1542356516303032)
    1. Original research article
    2. Clinical Gastroenterology and Hepatology,
    3. Volume 14, Issue 11,
    4. November 2016,
    5. Pages 1647-1656.e6
    6. Hye Soo Kim,
    7. Beom Kyung Kim,
    8. Seung Up Kim,
    9. Jun Yong Park,
    10. Sang Hoon Ahn
    11. [Purchase PDF](http://www.sciencedirect.com/science/article/pii/S1542356516303032)
47. [Non-invasive diagnosis of liver fibrosis in the transplant setting](http://www.sciencedirect.com/science/article/pii/S1594580411600211)
    1. Original research article
    2. Digestive and Liver Disease Supplements,
    3. Volume 5, Issue 1,
    4. December 2011,
    5. Pages 23-25
    6. Gonzalo Crespo,
    7. Zoe Mariño
    8. [Purchase PDF](http://www.sciencedirect.com/science/article/pii/S1594580411600211)
48. [Efficacy of Antiviral Therapy on Hepatitis C Recurrence After Liver Transplantation: A Randomized Controlled Study](http://www.sciencedirect.com/science/article/pii/S0016508507005653)
    1. Original research article
    2. Gastroenterology,
    3. Volume 132, Issue 5,
    4. May 2007,
    5. Pages 1746-1756
    6. José A. Carrión,
    7. Miquel Navasa,
    8. Montserrat García–Retortillo,
    9. Juan Carlos García–Pagan,
    10. Xavier Forns
    11. [Purchase PDF](http://www.sciencedirect.com/science/article/pii/S0016508507005653)
49. [Epidemiology and natural history of non-alcoholic fatty liver disease](http://www.sciencedirect.com/science/article/pii/S0026049516000275)
    1. Original research article
    2. Metabolism,
    3. Volume 65, Issue 8,
    4. August 2016,
    5. Pages 1017-1025
    6. Yousef Fazel,
    7. Aaron B. Koenig,
    8. Mehmet Sayiner,
    9. Zachary D. Goodman,
    10. Zobair M. Younossi
    11. [Purchase PDF](http://www.sciencedirect.com/science/article/pii/S0026049516000275)
50. [An association of large-fibre peripheral nerve dysfunction with non-invasive measures of liver fibrosis secondary to non-alcoholic fatty liver disease in diabetes](http://www.sciencedirect.com/science/article/pii/S1056872715002688)
    1. Original research article
    2. Journal of Diabetes and its Complications,
    3. Volume 29, Issue 8,
    4. November–December 2015,
    5. Pages 1240-1247
    6. Kathryn H. Williams,
    7. Kharis Burns,
    8. Maria Constantino,
    9. Nicholas A. Shackel,
    10. Stephen M. Twigg
    11. [Purchase PDF](http://www.sciencedirect.com/science/article/pii/S1056872715002688)
51. [Clinical effects of viral relapse after interferon plus ribavirin in patients co-infected with human immunodeficiency virus and hepatitis C virus](http://www.sciencedirect.com/science/article/pii/S0168827813000895)
    1. Original research article
    2. Journal of Hepatology,
    3. Volume 58, Issue 6,
    4. June 2013,
    5. Pages 1104-1112
    6. Juan Berenguer,
    7. Julio Alvarez-Pellicer,
    8. Ana Carrero,
    9. Miguel A. Von Wichmann,
    10. The GESIDA HIV/HCV Cohort Study Group
    11. [Purchase PDF](http://www.sciencedirect.com/science/article/pii/S0168827813000895)
52. [Baseline Values and Changes in Liver Stiffness Measured by Transient Elastography Are Associated With Severity of Fibrosis and Outcomes of Patients With Primary Sclerosing Cholangitis](http://www.sciencedirect.com/science/article/pii/S0016508513018441)
    1. Original research article
    2. Gastroenterology,
    3. Volume 146, Issue 4,
    4. April 2014,
    5. Pages 970-979.e6
    6. Christophe Corpechot,
    7. Farid Gaouar,
    8. Ahmed El Naggar,
    9. Astrid Kemgang,
    10. Olivier Chazouillères
    11. [Purchase PDF](http://www.sciencedirect.com/science/article/pii/S0016508513018441)
53. [Low Risk of Hepatocellular Carcinoma in Patients With Primary Sclerosing Cholangitis With Cirrhosis](http://www.sciencedirect.com/science/article/pii/S1542356514001980)
    1. Original research article
    2. Clinical Gastroenterology and Hepatology,
    3. Volume 12, Issue 10,
    4. October 2014,
    5. Pages 1733-1738
    6. Roman Zenouzi,
    7. Tobias J. Weismüller,
    8. Peter Hübener,
    9. Kornelius Schulze,
    10. Christoph Schramm
    11. [Purchase PDF](http://www.sciencedirect.com/science/article/pii/S1542356514001980)
54. [Features of Severe Liver Disease With Portal Hypertension in Patients With Cystic Fibrosis](http://www.sciencedirect.com/science/article/pii/S1542356516300167)
    1. Original research article
    2. Clinical Gastroenterology and Hepatology,
    3. Volume 14, Issue 8,
    4. August 2016,
    5. Pages 1207-1215.e3
    6. Jaclyn R. Stonebraker,
    7. Chee Y. Ooi,
    8. Rhonda G. Pace,
    9. Harriet Corvol,
    10. Simon C. Ling
    11. [Purchase PDF](http://www.sciencedirect.com/science/article/pii/S1542356516300167)
55. [Non-invasive assessment of hepatic fibrosis in a series of patients with Wilson's Disease](http://www.sciencedirect.com/science/article/pii/S1590865811004750)
    1. Original research article
    2. Digestive and Liver Disease,
    3. Volume 44, Issue 6,
    4. June 2012,
    5. Pages 487-491
    6. Margherita Sini,
    7. Orazio Sorbello,
    8. Alberto Civolani,
    9. Mauro Liggi,
    10. Luigi Demelia
    11. [Purchase PDF](http://www.sciencedirect.com/science/article/pii/S1590865811004750)
56. [Serum Fibrosis Markers Identify Patients With Mild and Progressive Hepatitis C Recurrence After LiverTransplantation](http://www.sciencedirect.com/science/article/pii/S0016508509016977)
    1. Original research article
    2. Gastroenterology,
    3. Volume 138, Issue 1,
    4. January 2010,
    5. Pages 147-158.e1
    6. José A. Carrión,
    7. Guillermo Fernández–Varo,
    8. Miquel Bruguera,
    9. Juan–Carlos García–Pagán,
    10. Miquel Navasa
    11. [Purchase PDF](http://www.sciencedirect.com/science/article/pii/S0016508509016977)
57. [Invasive and non-invasive methods for the assessment of fibrosis and disease progression in chronic liverdisease](http://www.sciencedirect.com/science/article/pii/S1521691811000291)
    1. Original research article
    2. Best Practice & Research Clinical Gastroenterology,
    3. Volume 25, Issue 2,
    4. April 2011,
    5. Pages 291-303
    6. Laurent Castera
    7. [Purchase PDF](http://www.sciencedirect.com/science/article/pii/S1521691811000291)
58. [Differences in liver stiffness values obtained with new ultrasound elastography machines and Fibroscan: A comparative study](http://www.sciencedirect.com/science/article/pii/S1590865817302463)
    1. Original research article
    2. Digestive and Liver Disease,
    3. Volume 49, Issue 7,
    4. July 2017,
    5. Pages 802-808
    6. Fabio Piscaglia,
    7. Veronica Salvatore,
    8. Lorenzo Mulazzani,
    9. Vito Cantisani,
    10. Luigi Bolondi
    11. [Purchase PDF](http://www.sciencedirect.com/science/article/pii/S1590865817302463)
59. [ARFI, FibroScan®, ELF, and their combinations in the assessment of liver fibrosis: A prospective study](http://www.sciencedirect.com/science/article/pii/S0168827812002711)
    1. Original research article
    2. Journal of Hepatology,
    3. Volume 57, Issue 2,
    4. August 2012,
    5. Pages 281-287
    6. Gonzalo Crespo,
    7. Guillermo Fernández-Varo,
    8. Zoe Mariño,
    9. Gregori Casals,
    10. Miquel Navasa
    11. [Purchase PDF](http://www.sciencedirect.com/science/article/pii/S0168827812002711)
60. [Hepatic steatosis progresses faster in HIV mono-infected than HIV/HCV co-infected patients and is associated with liver fibrosis](http://www.sciencedirect.com/science/article/pii/S0168827817320470)
    1. Original research article
    2. Journal of Hepatology,
    3. In press, corrected proof,
    4. Available online 18 May 2017
    5. Thomas Pembroke,
    6. Marc Deschenes,
    7. Bertrand Lebouché,
    8. Amine Benmassaoud,
    9. Giada Sebastiani
    10. [Purchase PDF](http://www.sciencedirect.com/science/article/pii/S0168827817320470)
61. [Early Acute Severe HCV Recurrence After Transplantation: From Universal Mortality to Cure](http://www.sciencedirect.com/science/article/pii/S0973688316302420)
    1. Original research article
    2. Journal of Clinical and Experimental Hepatology,
    3. Volume 7, Issue 1,
    4. March 2017,
    5. Pages 28-32
    6. Manav Wadhawan,
    7. Vivek Vij,
    8. Kausar Makki,
    9. Nalini Bansal,
    10. Ajay Kumar
    11. [Purchase PDF](http://www.sciencedirect.com/science/article/pii/S0973688316302420)
62. [Progressive Familial Intrahepatic Cholestasis (PFIC) in Indian Children: Clinical Spectrum and Outcome](http://www.sciencedirect.com/science/article/pii/S0973688316300585)
    1. Original research article
    2. Journal of Clinical and Experimental Hepatology,
    3. Volume 6, Issue 3,
    4. September 2016,
    5. Pages 203-208
    6. Sajan Agarwal,
    7. Bikrant Bihari Lal,
    8. Dinesh Rawat,
    9. Archana Rastogi,
    10. Seema Alam
    11. [Purchase PDF](http://www.sciencedirect.com/science/article/pii/S0973688316300585)
63. [Basal values and changes of liver stiffness predict the risk of disease progression in compensated advanced chronic liver disease](http://www.sciencedirect.com/science/article/pii/S1590865816304789)
    1. Original research article
    2. Digestive and Liver Disease,
    3. Volume 48, Issue 10,
    4. October 2016,
    5. Pages 1214-1219
    6. Mònica Pons,
    7. Macarena Simón-Talero,
    8. Laura Millán,
    9. Meritxell Ventura-Cots,
    10. Joan Genescà
    11. [Purchase PDF](http://www.sciencedirect.com/science/article/pii/S1590865816304789)
64. [Twenty-year protocol liver biopsies: Invasive but useful for the management of liver recipients](http://www.sciencedirect.com/science/article/pii/S0168827811008695)
    1. Original research article
    2. Journal of Hepatology,
    3. Volume 56, Issue 4,
    4. April 2012,
    5. Pages 840-847
    6. Mylène Sebagh,
    7. Didier Samuel,
    8. Teresa Maria Antonini,
    9. Audrey Coilly,
    10. Daniel Azoulay
    11. [Purchase PDF](http://www.sciencedirect.com/science/article/pii/S0168827811008695)
65. [Evaluating the risk of hepatocellular carcinoma in patients with prominently elevated liver stiffness measurements by FibroScan: a multicentre study](http://www.sciencedirect.com/science/article/pii/S1365182X16317543)
    1. Open archive,
    2. Original research article
    3. HPB,
    4. Volume 18, Issue 8,
    5. August 2016,
    6. Pages 678-683
    7. Maciej Adler,
    8. Licia Larocca,
    9. Francesca M. Trovato,
    10. Heather Marcinkowski,
    11. Simon D. Taylor-Robinson
    12. [Download PDF (372 KB)](http://www.sciencedirect.com/science/article/pii/S1365182X16317543/pdfft?md5=b6a889711465101f90555aaf0cb56179&pid=1-s2.0-S1365182X16317543-main.pdf)
66. [Etiology-related determinants of liver stiffness values in chronic viral hepatitis B or C](http://www.sciencedirect.com/science/article/pii/S0168827810008056)
    1. Original research article
    2. Journal of Hepatology,
    3. Volume 54, Issue 4,
    4. April 2011,
    5. Pages 621-628
    6. Mirella Fraquelli,
    7. Cristina Rigamonti,
    8. Giovanni Casazza,
    9. Maria Francesca Donato,
    10. Massimo Colombo
    11. [Purchase PDF](http://www.sciencedirect.com/science/article/pii/S0168827810008056)
67. [Optimising risk stratification in primary biliary cirrhosis: AST/platelet ratio index predicts outcome independent of ursodeoxycholic acid response](http://www.sciencedirect.com/science/article/pii/S0168827814001056)
    1. Original research article
    2. Journal of Hepatology,
    3. Volume 60, Issue 6,
    4. June 2014,
    5. Pages 1249-1258
    6. Palak J. Trivedi,
    7. Tony Bruns,
    8. Angela Cheung,
    9. Ka-Kit Li,
    10. Gideon M. Hirschfield
    11. [Purchase PDF](http://www.sciencedirect.com/science/article/pii/S0168827814001056)
68. [Relationship between hepatic haemodynamics assessed by Doppler ultrasound and liver stiffness](http://www.sciencedirect.com/science/article/pii/S1590865811003306)
    1. Original research article
    2. Digestive and Liver Disease,
    3. Volume 44, Issue 2,
    4. February 2012,
    5. Pages 154-159
    6. Veronica Salvatore,
    7. Alberto Borghi,
    8. Eugenia Peri,
    9. Antonio Colecchia,
    10. Luigi Bolondi
    11. [Purchase PDF](http://www.sciencedirect.com/science/article/pii/S1590865811003306)
69. [Treatment of hepatitis C in the post-transplant setting](http://www.sciencedirect.com/science/article/pii/S1594580411600223)
    1. Original research article
    2. Digestive and Liver Disease Supplements,
    3. Volume 5, Issue 1,
    4. December 2011,
    5. Pages 26-29
    6. Marina Berenguer
    7. [Purchase PDF](http://www.sciencedirect.com/science/article/pii/S1594580411600223)
70. [Ultrasound elastography and contrast-enhanced ultrasound in infants, children and adolescents](http://www.sciencedirect.com/science/article/pii/S0720048X14003258)
    1. Original research article
    2. European Journal of Radiology,
    3. Volume 83, Issue 9,
    4. September 2014,
    5. Pages 1560-1569
    6. Martin Stenzel,
    7. Hans-Joachim Mentzel
    8. [Purchase PDF](http://www.sciencedirect.com/science/article/pii/S0720048X14003258)
71. [Marcadores séricos de fibrosis hepática en pacientes con hepatitis crónica C. Valor pronóstico de los marcadores no invasivos de fibrosis en el trasplante de hígado](http://www.sciencedirect.com/science/article/pii/S0210570512700456)
    1. Original research article
    2. Gastroenterología y Hepatología,
    3. Volume 35, Supplement 2,
    4. December 2012,
    5. Pages 17-22
    6. Gonzalo Crespo,
    7. Xavier Forns,
    8. Miquel Navasa
    9. [Purchase PDF](http://www.sciencedirect.com/science/article/pii/S0210570512700456)
72. [Alcoholic steatohepatitis](http://www.sciencedirect.com/science/article/pii/S152169181000082X)
    1. Original research article
    2. Best Practice & Research Clinical Gastroenterology,
    3. Volume 24, Issue 5,
    4. October 2010,
    5. Pages 683-693
    6. Felix Stickel,
    7. Helmut K. Seitz
    8. [Purchase PDF](http://www.sciencedirect.com/science/article/pii/S152169181000082X)
73. [Non-invasive diagnosis and monitoring of liver fibrosis and cirrhosis](http://www.sciencedirect.com/science/article/pii/S1521691809000390)
    1. Original research article
    2. Best Practice & Research Clinical Gastroenterology,
    3. Volume 23, Issue 3,
    4. June 2009,
    5. Pages 453-460
    6. Ulrike W. Denzer,
    7. Stefan Lüth
    8. [Purchase PDF](http://www.sciencedirect.com/science/article/pii/S1521691809000390)
74. [Incidence of non-alcoholic fatty liver disease in Hong Kong: A population study with paired proton-magnetic resonance spectroscopy](http://www.sciencedirect.com/science/article/pii/S0168827814006291)
    1. Original research article
    2. Journal of Hepatology,
    3. Volume 62, Issue 1,
    4. January 2015,
    5. Pages 182-189
    6. Vincent Wai-Sun Wong,
    7. Grace Lai-Hung Wong,
    8. David Ka-Wai Yeung,
    9. Tina Kit-Ting Lau,
    10. Henry Lik-Yuen Chan
    11. [Purchase PDF](http://www.sciencedirect.com/science/article/pii/S0168827814006291)
75. [Shear wave elastography: An accurate technique to stage liver fibrosis in chronic liver diseases](http://www.sciencedirect.com/science/article/pii/S221156841500368X)
    1. Open archive,
    2. Original research article
    3. Diagnostic and Interventional Imaging,
    4. Volume 97, Issue 1,
    5. January 2016,
    6. Pages 91-99
    7. A. Guibal,
    8. G. Renosi,
    9. A. Rode,
    10. J.Y. Scoazec,
    11. T. Lefort
    12. [Download PDF (1,417 KB)](http://www.sciencedirect.com/science/article/pii/S221156841500368X/pdfft?md5=f08b7dfcebc937255a99dfe8b90be478&pid=1-s2.0-S221156841500368X-main.pdf)
76. [Levels of Alanine Aminotransferase Confound Use of Transient Elastography to Diagnose Fibrosis in Patients With Chronic Hepatitis C Virus Infection](http://www.sciencedirect.com/science/article/pii/S1542356512001140)
    1. Original research article
    2. Clinical Gastroenterology and Hepatology,
    3. Volume 10, Issue 8,
    4. August 2012,
    5. Pages 932-937.e1
    6. Elliot B. Tapper,
    7. Eric B. Cohen,
    8. Keyur Patel,
    9. Bruce Bacon,
    10. Nezam Afdhal
    11. [Purchase PDF](http://www.sciencedirect.com/science/article/pii/S1542356512001140)
77. [Meal ingestion markedly increases liver stiffness suggesting the need for liver stiffness determination in fasting conditions](http://www.sciencedirect.com/science/article/pii/S0210570515000291)
    1. Original research article
    2. Gastroenterología y Hepatología,
    3. Volume 38, Issue 7,
    4. August–September 2015,
    5. Pages 431-435
    6. Daniel Alvarez,
    7. Federico Orozco,
    8. José María Mella,
    9. Maria Anders,
    10. Ricardo Mastai
    11. [Purchase PDF](http://www.sciencedirect.com/science/article/pii/S0210570515000291)
78. [Efficacy and tolerability of interferon-free antiviral therapy in kidney transplant recipients with chronic hepatitis C](http://www.sciencedirect.com/science/article/pii/S016882781630753X)
    1. Original research article
    2. Journal of Hepatology,
    3. Volume 66, Issue 4,
    4. April 2017,
    5. Pages 718-723
    6. Inmaculada Fernández,
    7. Raquel Muñoz-Gómez,
    8. Juan M. Pascasio,
    9. Carme Baliellas,
    10. María-Carlota Londoño
    11. [Purchase PDF](http://www.sciencedirect.com/science/article/pii/S016882781630753X)
79. [Staging chronic hepatitis B into seven categories, defining inactive carriers and assessing treatment impact using a fibrosis biomarker (FibroTest®) and elastography (FibroScan®)](http://www.sciencedirect.com/science/article/pii/S0168827814004577)
    1. Original research article
    2. Journal of Hepatology,
    3. Volume 61, Issue 5,
    4. November 2014,
    5. Pages 994-1003
    6. Thierry Poynard,
    7. Julien Vergniol,
    8. Yen Ngo,
    9. Juliette Foucher,
    10. FibroFrance Study Group and the Bordeaux HBV Study Group
    11. [Purchase PDF](http://www.sciencedirect.com/science/article/pii/S0168827814004577)
80. [Thalassemia major between liver and heart: Where we are now](http://www.sciencedirect.com/science/article/pii/S1079979615000704)
    1. Original research article
    2. Blood Cells, Molecules, and Diseases,
    3. Volume 55, Issue 1,
    4. June 2015,
    5. Pages 82-88
    6. Carlo Dessì,
    7. GiovanBattista Leoni,
    8. Paolo Moi,
    9. Fabrice Danjou,
    10. Raffaella Origa
    11. [Purchase PDF](http://www.sciencedirect.com/science/article/pii/S1079979615000704)
81. [The long-term benefits of nucleos(t)ide analogs in compensated HBV cirrhotic patients with no or small esophageal varices: A 12-year prospective cohort study](http://www.sciencedirect.com/science/article/pii/S0168827815003979)
    1. Original research article
    2. Journal of Hepatology,
    3. Volume 63, Issue 5,
    4. November 2015,
    5. Pages 1118-1125
    6. Pietro Lampertico,
    7. Federica Invernizzi,
    8. Mauro Viganò,
    9. Alessandro Loglio,
    10. Massimo Colombo
    11. [Purchase PDF](http://www.sciencedirect.com/science/article/pii/S0168827815003979)
82. [A pilot study of autologous CD34-depleted bone marrow mononuclear cell transplantation via the hepatic artery in five patients with liver failure](http://www.sciencedirect.com/science/article/pii/S1465324913005458)
    1. Original research article
    2. Cytotherapy,
    3. Volume 15, Issue 12,
    4. December 2013,
    5. Pages 1571-1579
    6. Chung-Hwa Park,
    7. Si Hyun Bae,
    8. Hee Yeon Kim,
    9. Ja Kyung Kim,
    10. Young Sok Lee
    11. [Purchase PDF](http://www.sciencedirect.com/science/article/pii/S1465324913005458)
83. [Soluble receptor for advanced glycation end products and liver stiffness in postoperative biliary atresia](http://www.sciencedirect.com/science/article/pii/S0009912012006315)
    1. Original research article
    2. Clinical Biochemistry,
    3. Volume 46, Issue 3,
    4. February 2013,
    5. Pages 214-218
    6. Sittisak Honsawek,
    7. Paisarn Vejchapipat,
    8. Sunchai Payungporn,
    9. Apiradee Theamboonlers,
    10. Yong Poovorawan
    11. [Purchase PDF](http://www.sciencedirect.com/science/article/pii/S0009912012006315)
84. [Severity of cirrhosis should determine the operative modality for patients with early hepatocellular carcinoma and compensated liver function](http://www.sciencedirect.com/science/article/pii/S0039606015007291)
    1. Original research article
    2. Surgery,
    3. Volume 159, Issue 2,
    4. February 2016,
    5. Pages 621-631
    6. Zhi-yong Huang,
    7. Bin-yong Liang,
    8. Min Xiong,
    9. Ke-shuai Dong,
    10. Xiao-ping Chen
    11. [Purchase PDF](http://www.sciencedirect.com/science/article/pii/S0039606015007291)
85. [Real-time shear-wave elastography: Applicability, reliability and accuracy for clinically significant portal hypertension](http://www.sciencedirect.com/science/article/pii/S0168827814009258)
    1. Original research article
    2. Journal of Hepatology,
    3. Volume 62, Issue 5,
    4. May 2015,
    5. Pages 1068-1075
    6. Bogdan Procopet,
    7. Annalisa Berzigotti,
    8. Juan G. Abraldes,
    9. Fanny Turon,
    10. Jaime Bosch
    11. [Purchase PDF](http://www.sciencedirect.com/science/article/pii/S0168827814009258)
86. [Lactic acidosis in patients with hepatitis C virus cirrhosis and combined ribavirin/sofosbuvir treatment](http://www.sciencedirect.com/science/article/pii/S0168827815007928)
    1. Original research article
    2. Journal of Hepatology,
    3. Volume 64, Issue 4,
    4. April 2016,
    5. Pages 790-799
    6. Martin-Walter Welker,
    7. Stefan Luhne,
    8. Christian M. Lange,
    9. Johannes Vermehren,
    10. Christoph Sarrazin
    11. [Purchase PDF](http://www.sciencedirect.com/science/article/pii/S0168827815007928)
87. [How to Approach a Patient With Nonalcoholic Fatty Liver Disease](http://www.sciencedirect.com/science/article/pii/S0016508517358018)
    1. Original research article
    2. Gastroenterology,
    3. Volume 153, Issue 2,
    4. August 2017,
    5. Pages 345-349
    6. Herbert Tilg
    7. [Purchase PDF](http://www.sciencedirect.com/science/article/pii/S0016508517358018)
88. [Role of Nurse Practitioners in the Management of Cirrhotic Patients](http://www.sciencedirect.com/science/article/pii/S1555415512004503)
    1. Original research article
    2. The Journal for Nurse Practitioners,
    3. Volume 8, Issue 10,
    4. November–December 2012,
    5. Pages 816-821
    6. Kristina Tuesday Werner,
    7. Shari Terese Perez
    8. [Purchase PDF](http://www.sciencedirect.com/science/article/pii/S1555415512004503)
89. [A pilot study of ultrasound elastography as a non-invasive method to monitor liver disease in children with short bowel syndrome](http://www.sciencedirect.com/science/article/pii/S0022346817301653)
    1. Original research article
    2. Journal of Pediatric Surgery,
    3. Volume 52, Issue 6,
    4. June 2017,
    5. Pages 962-965
    6. Daniel Lodwick,
    7. Molly Dienhart,
    8. Jennifer N. Cooper,
    9. Bonita Fung,
    10. Peter C. Minneci
    11. [Purchase PDF](http://www.sciencedirect.com/science/article/pii/S0022346817301653)
90. [The degree of spleen stiffness measured on acoustic radiation force impulse elastography predicts the severity of portal hypertension in patients with biliary atresia after portoenterostomy](http://www.sciencedirect.com/science/article/pii/S0022346815000433)
    1. Original research article
    2. Journal of Pediatric Surgery,
    3. Volume 50, Issue 4,
    4. April 2015,
    5. Pages 559-564
    6. Hajime Uchida,
    7. Seisuke Sakamoto,
    8. Megumi Kobayashi,
    9. Takanobu Shigeta,
    10. Mureo Kasahara
    11. [Purchase PDF](http://www.sciencedirect.com/science/article/pii/S0022346815000433)
91. [Best practice guidance for the diagnosis and management of cystic fibrosis-associated liver disease](http://www.sciencedirect.com/science/article/pii/S1569199311600064)
    1. Open archive,
    2. Original research article
    3. Journal of Cystic Fibrosis,
    4. Volume 10, Supplement 2,
    5. June 2011,
    6. Pages s29-s36
    7. Dominique Debray,
    8. Deirdre Kelly,
    9. Roderick Houwen,
    10. Birgitta Strandvik,
    11. Carla Colombo
    12. [Download PDF (226 KB)](http://www.sciencedirect.com/science/article/pii/S1569199311600064/pdfft?md5=344deaae84df78163441bf4b0aa9439b&pid=1-s2.0-S1569199311600064-main.pdf)
92. [Measurement of Spleen Stiffness With Acoustic Radiation Force Impulse Imaging Predicts Mortality and Hepatic Decompensation in Patients With Liver Cirrhosis](http://www.sciencedirect.com/science/article/pii/S1542356516312344)
    1. Original research article
    2. Clinical Gastroenterology and Hepatology,
    3. In press, uncorrected proof,
    4. Available online 23 December 2016
    5. Yoshitaka Takuma,
    6. Youichi Morimoto,
    7. Hiroyuki Takabatake,
    8. Nobuyuki Toshikuni,
    9. Hiroshi Yamamoto
    10. [Purchase PDF](http://www.sciencedirect.com/science/article/pii/S1542356516312344)
93. [Early occurrence and recurrence of hepatocellular carcinoma in HCV-related cirrhosis treated with direct-acting antivirals](http://www.sciencedirect.com/science/article/pii/S0168827816303038)
    1. Original research article
    2. Journal of Hepatology,
    3. Volume 65, Issue 4,
    4. October 2016,
    5. Pages 727-733
    6. Fabio Conti,
    7. Federica Buonfiglioli,
    8. Alessandra Scuteri,
    9. Cristina Crespi,
    10. Stefano Brillanti
    11. [Purchase PDF](http://www.sciencedirect.com/science/article/pii/S0168827816303038)
94. [Lower serum fibroblast activation protein shows promise in the exclusion of clinically significant liverfibrosis due to non-alcoholic fatty liver disease in diabetes and obesity](http://www.sciencedirect.com/science/article/pii/S0168822715001242)
    1. Original research article
    2. Diabetes Research and Clinical Practice,
    3. Volume 108, Issue 3,
    4. June 2015,
    5. Pages 466-472
    6. K.H. Williams,
    7. A.J. Viera de Ribeiro,
    8. E. Prakoso,
    9. A.S. Veillard,
    10. M.D. Gorrell
    11. [Purchase PDF](http://www.sciencedirect.com/science/article/pii/S0168822715001242)
95. [Hepatitis B Virus Genotype C Is Associated With More Severe Liver Fibrosis Than Genotype B](http://www.sciencedirect.com/science/article/pii/S1542356509007617)
    1. Original research article
    2. Clinical Gastroenterology and Hepatology,
    3. Volume 7, Issue 12,
    4. December 2009,
    5. Pages 1361-1366
    6. Henry Lik–Yuen Chan,
    7. Grace Lai–Hung Wong,
    8. Chi–Hang Tse,
    9. Angel Mei–Ling Chim,
    10. Vincent Wai–Sun Wong
    11. [Purchase PDF](http://www.sciencedirect.com/science/article/pii/S1542356509007617)
96. [Alcohol intake increases the risk of HCC in hepatitis C virus-related compensated cirrhosis: A prospective study](http://www.sciencedirect.com/science/article/pii/S0168827816301842)
    1. Original research article
    2. Journal of Hepatology,
    3. Volume 65, Issue 3,
    4. September 2016,
    5. Pages 543-551
    6. Hélène Vandenbulcke,
    7. Christophe Moreno,
    8. Isabelle Colle,
    9. Jean-François Knebel,
    10. Pierre Deltenre
    11. [Purchase PDF](http://www.sciencedirect.com/science/article/pii/S0168827816301842)
97. [Practice guidelines for the diagnosis and management of nonalcoholic fatty liver disease: A decalogue from the Italian Association for the Study of the Liver (AISF) Expert Committee](http://www.sciencedirect.com/science/article/pii/S1590865810000459)
    1. Original research article
    2. Digestive and Liver Disease,
    3. Volume 42, Issue 4,
    4. April 2010,
    5. Pages 272-282
    6. P. Loria,
    7. L.E. Adinolfi,
    8. S. Bellentani,
    9. E. Bugianesi,
    10. The NAFLD Expert Committee of the Associazione Italiana per lo studio del Fegato (AISF)
    11. [Purchase PDF](http://www.sciencedirect.com/science/article/pii/S1590865810000459)
98. [Association Between Severe Portal Hypertension and Risk of Liver Decompensation in Patients With Hepatitis C, Regardless of Response to Antiviral Therapy](http://www.sciencedirect.com/science/article/pii/S1542356515004152)
    1. Original research article
    2. Clinical Gastroenterology and Hepatology,
    3. Volume 13, Issue 10,
    4. October 2015,
    5. Pages 1846-1853.e1
    6. Sabela Lens,
    7. Diego Rincón,
    8. Montserrat García-Retortillo,
    9. Agustín Albillos,
    10. Juan Carlos García-Pagán
    11. [Purchase PDF](http://www.sciencedirect.com/science/article/pii/S1542356515004152)
99. [25](http://www.sciencedirect.com/search?qs=%22Liver%20transplantation%22%20and%20%22transient%20elastography%22&show=25&sortBy=relevance&articleTypes=OR)
100. [50](http://www.sciencedirect.com/search?qs=%22Liver%20transplantation%22%20and%20%22transient%20elastography%22&show=50&sortBy=relevance&articleTypes=OR)
101. 100
102. Page 1 of 3
103. [next](http://www.sciencedirect.com/search?qs=%22Liver%20transplantation%22%20and%20%22transient%20elastography%22&show=100&sortBy=relevance&articleTypes=OR&offset=100)

[Feedback](http://www.sciencedirect.com/search?qs=%22Liver%20transplantation%22%20and%20%22transient%20elastography%22&show=100&sortBy=relevance&lastSelectedFacet=articleTypes&articleTypes=OR)

[About ScienceDirect](http://www.elsevier.com/solutions/sciencedirect)[Remote access](http://www.sciencedirect.com/science/activateaccess)[Shopping cart](http://www.sciencedirect.com/science?_ob=ShoppingCartURL&_method=display&_zone=TopNavBar&_origin=srp&md5=e5857d1631c4b1f612897ed029064507)[Contact and support](https://service.elsevier.com/app/contact/supporthub/sciencedirect/)[Terms and conditions](https://www.elsevier.com/legal/elsevier-website-terms-and-conditions)[Privacy policy](http://www.elsevier.com/legal/privacy-policy)

Cookies are used by this site. For more information, visit the [cookies page](https://www.elsevier.com/solutions/sciencedirect/support/cookies).

Copyright © 2017 Elsevier B.V. or its licensors or contributors. ScienceDirect ® is a registered trademark of Elsevier B.V.

[Advanced search](http://www.sciencedirect.com/science/search)

Bottom of Form

137 results

Refine by:

Top of Form

Years

1. 2017 (15)
2. 2016 (15)
3. 2015 (12)
4. 2014 (12)
5. 2013 (8)
6. 2012 (3)
7. 2011 (6)
8. 2010 (4)
9. 2009 (5)
10. 2008 (3)

Show less

Article type

1. Original research (137)

Publication title

1. Journal of Hepatology (12)
2. Transplantation Proceedings (10)
3. Gastroenterology (10)
4. Digestive and Liver Disease (9)
5. Clinical Gastroenterology and Hepatology (8)
6. Journal of Cardiothoracic and Vascular Anesthesia (6)
7. Thrombosis Research (5)
8. The American Journal of Cardiology (3)
9. Human Pathology (3)
10. Transplantation Reviews (3)

Show less

[Clear all filters](http://www.sciencedirect.com/search?qs=%22Liver%20transplantation%22%20and%20FIB-4&show=100&sortBy=relevance&offset=100&navigation=true)

Bottom of Form

sorted by *relevance* | [date](http://www.sciencedirect.com/search?qs=%22Liver%20transplantation%22%20and%20FIB-4&show=100&sortBy=date&articleTypes=OR&offset=100&navigation=true)

1. [Fibrosis in alcoholic and nonalcoholic steatohepatitis](http://www.sciencedirect.com/science/article/pii/S1521691811000369)
   1. Original research article
   2. Best Practice & Research Clinical Gastroenterology,
   3. Volume 25, Issue 2,
   4. April 2011,
   5. Pages 231-244
   6. Ramon Bataller,
   7. Krista Rombouts,
   8. José Altamirano,
   9. Fabio Marra
   10. [Purchase PDF](http://www.sciencedirect.com/science/article/pii/S1521691811000369)
2. [Management of a postpartum coagulopathy using thrombelastography](http://www.sciencedirect.com/science/article/pii/S0952818097000263)
   1. Original research article
   2. Journal of Clinical Anesthesia,
   3. Volume 9, Issue 3,
   4. May 1997,
   5. Pages 243-247
   6. Shiv K. Sharma,
   7. Richard L. Vera,
   8. William C. Stegall,
   9. Charles W. Whitten
   10. [Purchase PDF](http://www.sciencedirect.com/science/article/pii/S0952818097000263)
3. Want a richer search experience?

Sign in for additional filter options, multiple article downloads, and more.

Top of Form

Sign in

Bottom of Form

1. [Establishment of a highly differentiated immortalized human hepatocyte cell line as a source of hepatic function in the bioartificial liver](http://www.sciencedirect.com/science/article/pii/S0041134599009434)
   1. Original research article
   2. Transplantation Proceedings,
   3. Volume 32, Issue 2,
   4. March 2000,
   5. Pages 237-241
   6. N Kobayashi,
   7. M Miyazaki,
   8. K Fukaya,
   9. Y Inoue,
   10. M Namba
   11. [Purchase PDF](http://www.sciencedirect.com/science/article/pii/S0041134599009434)
2. [The contribution of fibrinolysis to postbypass bleeding](http://www.sciencedirect.com/science/article/pii/1053077091900814)
   1. Original research article
   2. Journal of Cardiothoracic and Vascular Anesthesia,
   3. Volume 5, Issue 6, Supplement 1,
   4. December 1991,
   5. Pages 13-17
   6. Bruce D. Spiess
   7. [Purchase PDF](http://www.sciencedirect.com/science/article/pii/1053077091900814)
3. [Vitamin E and vitamin C treatment improves fibrosis in patients with nonalcoholic steatohepatitis](http://www.sciencedirect.com/science/article/pii/S0002927003017866)
   1. Original research article
   2. The American Journal of Gastroenterology,
   3. Volume 98, Issue 11,
   4. November 2003,
   5. Pages 2485-2490
   6. Stephen A Harrison,
   7. Sigurd Torgerson,
   8. Paul Hayashi,
   9. John Ward,
   10. Steven Schenker
   11. [Purchase PDF](http://www.sciencedirect.com/science/article/pii/S0002927003017866)
4. [Detection and clinical significance of red cell aggregation in the human subcutaneous vein using a high-frequency transducer (10 MHz): A preliminary report](http://www.sciencedirect.com/science/article/pii/S030156299700015X)
   1. Original research article
   2. Ultrasound in Medicine & Biology,
   3. Volume 23, Issue 6,
   4. 1997,
   5. Pages 933-938
   6. Hiroshi Kitamura,
   7. Seiji Kawasaki
   8. [Purchase PDF](http://www.sciencedirect.com/science/article/pii/S030156299700015X)
5. [Adrenaline plus cyanoacrylate injection for treatment of bleeding peptic ulcers after failure of conventional endoscopic haemostasis](http://www.sciencedirect.com/science/article/pii/S1590865802801293)
   1. Original research article
   2. Digestive and Liver Disease,
   3. Volume 34, Issue 5,
   4. May 2002,
   5. Pages 349-355
   6. A. Repici,
   7. A. Ferrari,
   8. C. De Angelis,
   9. S. Caronna,
   10. G. Saracco
   11. [Purchase PDF](http://www.sciencedirect.com/science/article/pii/S1590865802801293)
6. [The surgery of “correctable” biliary atresia](http://www.sciencedirect.com/science/article/pii/S0022346887802129)
   1. Original research article
   2. Journal of Pediatric Surgery,
   3. Volume 22, Issue 6,
   4. June 1987,
   5. Pages 522-525
   6. John R. Lilly,
   7. Roberta J. Hall,
   8. Juan Vasquez-Estevez,
   9. Frederick Karrer,
   10. Robert H. Shikes
   11. [Purchase PDF](http://www.sciencedirect.com/science/article/pii/S0022346887802129)
7. [Les acides biliaires et leur utilisation thérapeutique chez l'enfant](http://www.sciencedirect.com/science/article/pii/0929693X96899234)
   1. Original research article
   2. Archives de Pédiatrie,
   3. Volume 2, Issue 12,
   4. December 1995,
   5. Pages 1200-1208
   6. F Cacaille
   7. [Purchase PDF](http://www.sciencedirect.com/science/article/pii/0929693X96899234)
8. [Hepatitis C Virus: A Critical Appraisal of Approaches to Therapy](http://www.sciencedirect.com/science/article/pii/S1542356508011609)
   1. Original research article
   2. Clinical Gastroenterology and Hepatology,
   3. Volume 7, Issue 4,
   4. April 2009,
   5. Pages 397-414
   6. David R. Nelson,
   7. Gary L. Davis,
   8. Ira Jacobson,
   9. Gregory T. Everson,
   10. Nizar Zein
   11. [Purchase PDF](http://www.sciencedirect.com/science/article/pii/S1542356508011609)
9. [Morphometric Differences in Pulmonary Lesions in Primary and Secondary ARDS: A Preliminary Study in Autopsies](http://www.sciencedirect.com/science/article/pii/S0344033804701223)
   1. Original research article
   2. Pathology - Research and Practice,
   3. Volume 197, Issue 8,
   4. 2001,
   5. Pages 521-530
   6. Cristiane Hoelz,
   7. Elnara M. Negri,
   8. Ana J.F.C. Lichtenfels,
   9. Gleice M.S. Conceição,
   10. Vera L. Capelozzi
   11. [Purchase PDF](http://www.sciencedirect.com/science/article/pii/S0344033804701223)
10. [High haematocrit in cyanotic congenital heart disease affects how fibrinogen activity is determined by rotational thromboelastometry](http://www.sciencedirect.com/science/article/pii/S0049384813002843)
    1. Original research article
    2. Thrombosis Research,
    3. Volume 132, Issue 2,
    4. August 2013,
    5. Pages e145-e151
    6. Sarah K. Westbury,
    7. Kurtis Lee,
    8. Christopher Reilly-Stitt,
    9. Robert Tulloh,
    10. Andrew D. Mumford
    11. [Purchase PDF](http://www.sciencedirect.com/science/article/pii/S0049384813002843)
11. [Postoperative changes in coagulant and anticoagulant factors following abdominal aortic surgery](http://www.sciencedirect.com/science/article/pii/1053077092900518)
    1. Original research article
    2. Journal of Cardiothoracic and Vascular Anesthesia,
    3. Volume 6, Issue 6,
    4. December 1992,
    5. Pages 680-685
    6. Neville M. Gibbs,
    7. G. Patrick,
    8. M. Crawford,
    9. Nick Michalopoulos
    10. [Purchase PDF](http://www.sciencedirect.com/science/article/pii/1053077092900518)
12. [Utilidad del Fibroscan® para evaluar la fibrosis hepática](http://www.sciencedirect.com/science/article/pii/S0210570509003562)
    1. Original research article
    2. Gastroenterología y Hepatología,
    3. Volume 32, Issue 6,
    4. June–July 2009,
    5. Pages 415-423
    6. José A. Carrión
    7. [Purchase PDF](http://www.sciencedirect.com/science/article/pii/S0210570509003562)
13. [Comparison of thromboelastography to bleeding time and standard coagulation tests in patients after cardiopulmonary bypass](http://www.sciencedirect.com/science/article/pii/105307709390161D)
    1. Original research article
    2. Journal of Cardiothoracic and Vascular Anesthesia,
    3. Volume 7, Issue 4,
    4. August 1993,
    5. Pages 410-415
    6. James H. Essell,
    7. Thomas J. Martin,
    8. John Salinas,
    9. James M. Thompson,
    10. Vernon C. Smith
    11. [Purchase PDF](http://www.sciencedirect.com/science/article/pii/105307709390161D)
14. [Hemochromatosis and alcoholic liver disease](http://www.sciencedirect.com/science/article/pii/S0741832903001289)
    1. Original research article
    2. Alcohol,
    3. Volume 30, Issue 2,
    4. June 2003,
    5. Pages 131-136
    6. Linda M Fletcher,
    7. Lawrie W Powell
    8. [Purchase PDF](http://www.sciencedirect.com/science/article/pii/S0741832903001289)
15. [Thromboelastometry-guided administration of fibrinogen concentrate for the treatment of excessive intraoperative bleeding in thoracoabdominal aortic aneurysm surgery](http://www.sciencedirect.com/science/article/pii/S002252230900350X)
    1. Open archive,
    2. Original research article
    3. The Journal of Thoracic and Cardiovascular Surgery,
    4. Volume 138, Issue 3,
    5. September 2009,
    6. Pages 694-702
    7. Niels Rahe-Meyer,
    8. Cristina Solomon,
    9. Michael Winterhalter,
    10. Siegfried Piepenbrock,
    11. Maximilian Pichlmaier
    12. [Download PDF (225 KB)](http://www.sciencedirect.com/science/article/pii/S002252230900350X/pdfft?md5=7b6a3db29b88590bee12712b91375822&pid=1-s2.0-S002252230900350X-main.pdf)
16. [Recombinant α-interferon treatment of non-A, non-B (type C) hepatitis: review of studies and recommendations for treatment](http://www.sciencedirect.com/science/article/pii/016882789090168Q)
    1. Original research article
    2. Journal of Hepatology,
    3. Volume 11, Supplement 1,
    4. 1990,
    5. Pages s72-s77
    6. G.L. Davis
    7. [Purchase PDF](http://www.sciencedirect.com/science/article/pii/016882789090168Q)
17. [Comparative effects of an erythrocyte enriched synthetic replenisher and whole blood on streptokinase activity in the isolated perfused liver](http://www.sciencedirect.com/science/article/pii/0049384879900318)
    1. Original research article
    2. Thrombosis Research,
    3. Volume 14, Issue 1,
    4. January 1979,
    5. Pages 125-130
    6. R. Losito,
    7. H. Gattiker,
    8. E. Lemieux,
    9. B. Longpré
    10. [Purchase PDF](http://www.sciencedirect.com/science/article/pii/0049384879900318)
18. [Human hepatocytes express the gene for type 1 plasminogen activator-inhibitor (PAI-1) in vivo](http://www.sciencedirect.com/science/article/pii/S0268949908800410)
    1. Original research article
    2. Fibrinolysis,
    3. Volume 9, Issue 1,
    4. January 1995,
    5. Pages 9-15
    6. A.J. Thornton,
    7. T.D. Gelehrter
    8. [Purchase PDF](http://www.sciencedirect.com/science/article/pii/S0268949908800410)
19. [Prognostic indicators of successful endoscopic sclerotherapy for prevention of rebleeding from oesophageal varices in cirrhosis: a long-term cohort study](http://www.sciencedirect.com/science/article/pii/S1590865800803564)
    1. Original research article
    2. Digestive and Liver Disease,
    3. Volume 32, Issue 9,
    4. December 2000,
    5. Pages 782-791
    6. S. Madonia,
    7. G. D'Amico,
    8. M. Traina,
    9. G. Gatto,
    10. L. Pagliaro
    11. [Purchase PDF](http://www.sciencedirect.com/science/article/pii/S1590865800803564)
20. [Mycophenolate mofetil (RS-61443): Mechanisms of action and effects in transplantation](http://www.sciencedirect.com/science/article/pii/S0955470X05800126)
    1. Original research article
    2. Transplantation Reviews,
    3. Volume 7, Issue 3,
    4. July 1993,
    5. Pages 129-139
    6. Anthony C. Allison,
    7. Elsie M. Eugui,
    8. Hans W. Sollinger
    9. [Purchase PDF](http://www.sciencedirect.com/science/article/pii/S0955470X05800126)
21. [Safety of dipyridamole testing in 73,806 patients: The Multicenter Dipyridamole Safety Study](http://www.sciencedirect.com/science/article/pii/S1071358105800030)
    1. Original research article
    2. Journal of Nuclear Cardiology,
    3. Volume 2, Issue 1,
    4. January–February 1995,
    5. Pages 3-17
    6. Jean Lette,
    7. James L. Tatum,
    8. Sheila Fraser,
    9. D. Douglas Miller,
    10. Multicenter Dipyridamole Safety Study Investigators
    11. [Purchase PDF](http://www.sciencedirect.com/science/article/pii/S1071358105800030)
22. [Mycophenolate mofetil and its mechanisms of action](http://www.sciencedirect.com/science/article/pii/S0162310900001880)
    1. Original research article
    2. Immunopharmacology,
    3. Volume 47, Issues 2–3,
    4. May 2000,
    5. Pages 85-118
    6. Anthony C Allison,
    7. Elsie M Eugui
    8. [Purchase PDF](http://www.sciencedirect.com/science/article/pii/S0162310900001880)
23. [Lymphocyte migration and tissue positioning in allograft reciepients: The role played by extracellular matrix proteins](http://www.sciencedirect.com/science/article/pii/S0955470X9580013X)
    1. Original research article
    2. Transplantation Reviews,
    3. Volume 9, Issue 1,
    4. January 1995,
    5. Pages 29-40
    6. Jerzy W. Kupiec-Weglinski,
    7. Ana J. Coito,
    8. Andrej Gorski,
    9. Maria de Sousa
    10. [Purchase PDF](http://www.sciencedirect.com/science/article/pii/S0955470X9580013X)
24. [Thromboelastogram fails to predict postoperative hemorrhage in cardiac patients](http://www.sciencedirect.com/science/article/pii/0003497592902645)
    1. Original research article
    2. The Annals of Thoracic Surgery,
    3. Volume 53, Issue 3,
    4. March 1992,
    5. Pages 435-439
    6. Jian-Sheng Wang,
    7. Chung-Yuan Lin,
    8. Wei-Te Hung,
    9. Michael F. O'Connor,
    10. Ming-Wen Yang
    11. [Purchase PDF](http://www.sciencedirect.com/science/article/pii/0003497592902645)
25. [B-cell lymphoproliferative disorders in solid-organ transplant patients: Detection of Epstein-Barr virus by in situ hybridization](http://www.sciencedirect.com/science/article/pii/004681779290237W)
    1. Original research article
    2. Human Pathology,
    3. Volume 23, Issue 2,
    4. February 1992,
    5. Pages 159-163
    6. Laurence C. Berg,
    7. Cedith M. Copenhaver,
    8. Vicki A. Morrison,
    9. Scott A. Gruber,
    10. John G. Strickler
    11. [Purchase PDF](http://www.sciencedirect.com/science/article/pii/004681779290237W)
26. [Management approaches to platelet-related microvascular bleeding in cardiothoracic surgery](http://www.sciencedirect.com/science/article/pii/S0003497500016040)
    1. Original research article
    2. The Annals of Thoracic Surgery,
    3. Volume 70, Issue 2, Supplement 1,
    4. August 2000,
    5. Pages s20-s32
    6. George J Despotis,
    7. Lawrence T Goodnough
    8. [Purchase PDF](http://www.sciencedirect.com/science/article/pii/S0003497500016040)
27. [Differential inhibition of individual human liver cytochromes P-450 by cimetidine](http://www.sciencedirect.com/science/article/pii/001650859190408D)
    1. Original research article
    2. Gastroenterology,
    3. Volume 101, Issue 6,
    4. December 1991,
    5. Pages 1680-1691
    6. Robert G. Knodell,
    7. David G. Browne,
    8. Glenn P. Gwozdz,
    9. Wiliam R. Brian,
    10. F.Peter Guengerich
    11. [Purchase PDF](http://www.sciencedirect.com/science/article/pii/001650859190408D)
28. [Weekly treatment of diet/drug-resistant hypercholesterolemia with the heparin-induced extracorporeal low-density lipoprotein precipitation (HELP) system by selective plasma low-density lipoprotein removal](http://www.sciencedirect.com/science/article/pii/0002914993908306)
    1. Original research article
    2. The American Journal of Cardiology,
    3. Volume 71, Issue 10,
    4. 1 April 1993,
    5. Pages 816-822
    6. Daniel M. Lane,
    7. Walter J. McConathy,
    8. L.O. Laughlin,
    9. Philip C. Comp,
    10. Catharine Dorrier
    11. [Purchase PDF](http://www.sciencedirect.com/science/article/pii/0002914993908306)
29. [Disturbo dell’emostasi in pronto soccorso](http://www.sciencedirect.com/science/article/pii/S1286934110700107)
    1. Original research article
    2. EMC - Urgenze,
    3. Volume 14, Issue 1,
    4. 2010,
    5. Pages 1-22
    6. N. Nathan,
    7. A. Julia
    8. [Purchase PDF](http://www.sciencedirect.com/science/article/pii/S1286934110700107)
30. [Haemostyptic preparations on the basis of collagen alone and as fixed combination with fibrin glue](http://www.sciencedirect.com/science/article/pii/026766059290097D)
    1. Original research article
    2. Clinical Materials,
    3. Volume 9, Issues 3–4,
    4. 1992,
    5. Pages 169-177
    6. Ulrich Schiele,
    7. Günter Kuntz,
    8. Anita Riegler
    9. [Purchase PDF](http://www.sciencedirect.com/science/article/pii/026766059290097D)
31. [Secondary osteoporosis](http://www.sciencedirect.com/science/article/pii/S0950351X97805216)
    1. Original research article
    2. Baillière's Clinical Endocrinology and Metabolism,
    3. Volume 11, Issue 1,
    4. April 1997,
    5. Pages 83-99
    6. David M. Reid,
    7. John Harvie
    8. [Purchase PDF](http://www.sciencedirect.com/science/article/pii/S0950351X97805216)
32. [Emerging and underrecognized respiratory infections in children](http://www.sciencedirect.com/science/article/pii/S1045187005800482)
    1. Original research article
    2. Seminars in Pediatric Infectious Diseases,
    3. Volume 6, Issue 3,
    4. July 1995,
    5. Pages 201-208
    6. Jay C. Butler
    7. [Purchase PDF](http://www.sciencedirect.com/science/article/pii/S1045187005800482)
33. [Thérapeutique des mycoses profondes (à l'exception des aspergilloses et des fusarioses)](http://www.sciencedirect.com/science/article/pii/S0338989801801244)
    1. Original research article
    2. Revue Française des Laboratoires,
    3. Volume 2001, Issue 332,
    4. April 2001,
    5. Pages 23-30
    6. Sophie Léautez,
    7. François Raffi
    8. [Purchase PDF](http://www.sciencedirect.com/science/article/pii/S0338989801801244)
34. [Enzymes as agents for the treatment of disease](http://www.sciencedirect.com/science/article/pii/000989819290007D)
    1. Original research article
    2. Clinica Chimica Acta,
    3. Volume 206, Issues 1–2,
    4. 13 March 1992,
    5. Pages 45-76
    6. David M. Goldberg
    7. [Purchase PDF](http://www.sciencedirect.com/science/article/pii/000989819290007D)
35. [Optimal Management of Bleeding and Transfusion in Patients Undergoing Cardiac Surgery](http://www.sciencedirect.com/science/article/pii/S104306799970002X)
    1. Original research article
    2. Seminars in Thoracic and Cardiovascular Surgery,
    3. Volume 11, Issue 2,
    4. April 1999,
    5. Pages 84-104
    6. George J. Despotis,
    7. Nikolaos J. Skubas,
    8. Lawrence Tim Goodnough
    9. [Purchase PDF](http://www.sciencedirect.com/science/article/pii/S104306799970002X)
36. [25](http://www.sciencedirect.com/search?qs=%22Liver%20transplantation%22%20and%20FIB-4&show=25&sortBy=relevance&articleTypes=OR&navigation=true)
37. [50](http://www.sciencedirect.com/search?qs=%22Liver%20transplantation%22%20and%20FIB-4&show=50&sortBy=relevance&articleTypes=OR&navigation=true)
38. 100
39. [previous](http://www.sciencedirect.com/search?qs=%22Liver%20transplantation%22%20and%20FIB-4&show=100&sortBy=relevance&articleTypes=OR&offset=0&navigation=true)
40. Page 2 of 2

[Feedback](http://www.sciencedirect.com/search?qs=%22Liver%20transplantation%22%20and%20FIB-4&show=100&sortBy=relevance&articleTypes=OR&offset=100)

[About ScienceDirect](http://www.elsevier.com/solutions/sciencedirect)[Remote access](http://www.sciencedirect.com/science/activateaccess)[Shopping cart](http://www.sciencedirect.com/science?_ob=ShoppingCartURL&_method=display&_zone=TopNavBar&_origin=srp&md5=e5857d1631c4b1f612897ed029064507)[Contact and support](https://service.elsevier.com/app/contact/supporthub/sciencedirect/)[Terms and conditions](https://www.elsevier.com/legal/elsevier-website-terms-and-conditions)[Privacy policy](http://www.elsevier.com/legal/privacy-policy)

Cookies are used by this site. For more information, visit the [cookies page](https://www.elsevier.com/solutions/sciencedirect/support/cookies).

Copyright © 2017 Elsevier B.V. or its licensors or contributors. ScienceDirect ® is a registered trademark of Elsevier B.V.

“Liver transplantation” and “Transient elastography”

[Skip to Main content](http://www.sciencedirect.com/search?qs=%22Liver%20transplantation%22%20and%20%22transient%20elastography%22&show=100&sortBy=relevance&articleTypes=OR&lastSelectedFacet=articleTypes#main_content)

- [Journals](http://www.sciencedirect.com/science/journals)
- [Books](http://www.sciencedirect.com/science/bookbshsrw)
- [Register](https://www.sciencedirect.com/user/register?returnURL=http%3A%2F%2Fwww.sciencedirect.com%2Fsearch)
- [Sign in](https://www.sciencedirect.com/user/login?returnURL=http%3A%2F%2Fwww.sciencedirect.com%2Fsearch)

Top of Form

[Advanced search](http://www.sciencedirect.com/science/search)

Bottom of Form

252 results

Refine by:

Top of Form

Years

1. 2017 (35)
2. 2016 (48)
3. 2015 (36)
4. 2014 (23)
5. 2013 (15)
6. 2012 (28)
7. 2011 (18)
8. 2010 (14)
9. 2009 (13)
10. 2008 (15)

Show less

Article type

1. Review articles (172)
2. Original research (252)
3. Encyclopedia (2)
4. Book chapters (60)
5. Other (636)

Show less

Publication title

1. Journal of Hepatology (53)
2. Clinical Gastroenterology and Hepatology (24)
3. Gastroenterology (22)
4. Digestive and Liver Disease (19)
5. Transplantation Proceedings (9)
6. Ultrasound in Medicine & Biology (8)
7. European Journal of Radiology (6)
8. Metabolism (6)
9. Best Practice & Research Clinical Gastroenterology (6)
10. Gastroenterología y Hepatología (5)

Show less

[Clear all filters](http://www.sciencedirect.com/search?qs=%22Liver%20transplantation%22%20and%20%22transient%20elastography%22&show=100&sortBy=relevance)

Bottom of Form

sorted by *relevance* | [date](http://www.sciencedirect.com/search?qs=%22Liver%20transplantation%22%20and%20%22transient%20elastography%22&show=100&sortBy=date&articleTypes=OR)

1. [Comparison of 2-D Shear Wave Elastography and Transient Elastography for Assessing Liver Fibrosis in Chronic Hepatitis B](http://www.sciencedirect.com/science/article/pii/S0301562917301369)
   1. Original research article
   2. Ultrasound in Medicine & Biology,
   3. Volume 43, Issue 8,
   4. August 2017,
   5. Pages 1563-1570
   6. Jie Zeng,
   7. Jian Zheng,
   8. Zeping Huang,
   9. Shigao Chen,
   10. Mingde Lu
   11. [Purchase PDF](http://www.sciencedirect.com/science/article/pii/S0301562917301369)
2. [Transient elastography in autoimmune hepatitis: Timing determines the impact of inflammation and fibrosis](http://www.sciencedirect.com/science/article/pii/S0168827816302094)
   1. Original research article
   2. Journal of Hepatology,
   3. Volume 65, Issue 4,
   4. October 2016,
   5. Pages 769-775
   6. Johannes Hartl,
   7. Ulrike Denzer,
   8. Hanno Ehlken,
   9. Roman Zenouzi,
   10. Christoph Schramm
   11. [Purchase PDF](http://www.sciencedirect.com/science/article/pii/S0168827816302094)
3. Want a richer search experience?

Sign in for additional filter options, multiple article downloads, and more.

Top of Form

Sign in

Bottom of Form

1. [Non-invasive assessment of liver graft fibrosis by transient elastography after liver transplantation](http://www.sciencedirect.com/science/article/pii/S2210740112003269)
   1. Original research article
   2. Clinics and Research in Hepatology and Gastroenterology,
   3. Volume 37, Issue 4,
   4. September 2013,
   5. Pages 347-352
   6. Camille Barrault,
   7. Françoise Roudot-Thoraval,
   8. Jeanne Tran Van Nhieu,
   9. Calina Atanasiu,
   10. Christophe Duvoux
   11. [Purchase PDF](http://www.sciencedirect.com/science/article/pii/S2210740112003269)
2. [Graft Function Measured by Transient Elastography in Living Donor Liver Transplantation: Preliminary](http://www.sciencedirect.com/science/article/pii/S004113451300794X)
   1. Original research article
   2. Transplantation Proceedings,
   3. Volume 45, Issue 8,
   4. October 2013,
   5. Pages 3028-3031
   6. S.H. Lee,
   7. D.J. Joo,
   8. S.U. Kim,
   9. M.S. Kim,
   10. S.I. Kim
   11. [Purchase PDF](http://www.sciencedirect.com/science/article/pii/S004113451300794X)
3. [Longitudinal Transient Elastography Measurements Used in Follow-up for Patients with Cystic Fibrosis](http://www.sciencedirect.com/science/article/pii/S0301562915006754)
   1. Original research article
   2. Ultrasound in Medicine & Biology,
   3. Volume 42, Issue 4,
   4. April 2016,
   5. Pages 848-854
   6. Stephanie Van Biervliet,
   7. Hugo Verdievel,
   8. Saskia Vande Velde,
   9. Ruth De Bruyne,
   10. Hans Van Vlierberghe
   11. [Purchase PDF](http://www.sciencedirect.com/science/article/pii/S0301562915006754)
4. [Monitoring the Natural Evolution and Response to Treatment of Post Liver Transplant Recurrent Hepatitis C Using Transient Elastography: Preliminary Results](http://www.sciencedirect.com/science/article/pii/S0041134512007889)
   1. Original research article
   2. Transplantation Proceedings,
   3. Volume 44, Issue 7,
   4. September 2012,
   5. Pages 2082-2086
   6. F. Bellido-Muñoz,
   7. A. Giráldez-Gallego,
   8. C. Roca-Oporto,
   9. T. García-Cayuela,
   10. J.M. Sousa-Martín
   11. [Purchase PDF](http://www.sciencedirect.com/science/article/pii/S0041134512007889)
5. [The Most Frequently Cited 100 Articles in Liver Transplantation Literature](http://www.sciencedirect.com/science/article/pii/S0041134517300520)
   1. Original research article
   2. Transplantation Proceedings,
   3. Volume 49, Issue 3,
   4. April 2017,
   5. Pages 551-561
   6. M. Özbilgin,
   7. T. Ünek,
   8. T. Egeli,
   9. C. Ağalar,
   10. İ. Astarcıoğlu
   11. [Purchase PDF](http://www.sciencedirect.com/science/article/pii/S0041134517300520)
6. [Liver stiffness measurement by transient elastography predicts late posthepatectomy outcomes in patients undergoing resection for hepatocellular carcinoma](http://www.sciencedirect.com/science/article/pii/S0039606017303963)
   1. Original research article
   2. Surgery,
   3. In press, corrected proof,
   4. Available online 12 July 2017
   5. Muthukumarassamy Rajakannu,
   6. Daniel Cherqui,
   7. Oriana Ciacio,
   8. Nicolas Golse,
   9. Eric Vibert
   10. [Purchase PDF](http://www.sciencedirect.com/science/article/pii/S0039606017303963)
7. [Efficacy and Safety of Therapy With Simeprevir and Sofosbuvir in Liver Transplant Recipients Infected by Hepatitis C Virus Genotype 4: Cohort Spanish Society of Liver Transplantation Cohort](http://www.sciencedirect.com/science/article/pii/S0041134516305309)
   1. Original research article
   2. Transplantation Proceedings,
   3. Volume 48, Issue 9,
   4. November 2016,
   5. Pages 3013-3016
   6. G. Sanchez Antolin,
   7. M. Testillano,
   8. J.M. Pascasio,
   9. I. Narvaez Rodriguez,
   10. Spanish Society of Liver Transplantation Study Group Collaborating Group
   11. [Purchase PDF](http://www.sciencedirect.com/science/article/pii/S0041134516305309)
8. [Assessment of liver fibrosis in transplant recipients with recurrent HCV infection: Usefulness of transientelastography](http://www.sciencedirect.com/science/article/pii/S1590865808002533)
   1. Original research article
   2. Digestive and Liver Disease,
   3. Volume 41, Issue 3,
   4. March 2009,
   5. Pages 217-225
   6. F. Corradi,
   7. F. Piscaglia,
   8. S. Flori,
   9. A. D’Errico-Grigioni,
   10. The Bologna Liver Transplantation Group (BLTG)
   11. [Purchase PDF](http://www.sciencedirect.com/science/article/pii/S1590865808002533)
9. [Liver and spleen transient elastography and Acoustic Radiation Force Impulse Measurements. Performance and comparison of measurements in the same area concurrently assessed for liver fibrosis by biopsy](http://www.sciencedirect.com/science/article/pii/S1896112615000334)
   1. Original research article
   2. Advances in Medical Sciences,
   3. Volume 60, Issue 2,
   4. September 2015,
   5. Pages 300-306
   6. Francesca M. Trovato,
   7. Sebastiana Atzori,
   8. Giuseppe Musumeci,
   9. Vanessa Tooley,
   10. Simon D. Taylor-Robinson
   11. [Purchase PDF](http://www.sciencedirect.com/science/article/pii/S1896112615000334)
10. [FibroScan (Vibration-Controlled Transient Elastography): Where Does It Stand in the United States Practice](http://www.sciencedirect.com/science/article/pii/S1542356514008180)
    1. Original research article
    2. Clinical Gastroenterology and Hepatology,
    3. Volume 13, Issue 1,
    4. January 2015,
    5. Pages 27-36
    6. Elliot B. Tapper,
    7. Laurent Castera,
    8. Nezam H. Afdhal
    9. [Purchase PDF](http://www.sciencedirect.com/science/article/pii/S1542356514008180)
11. [Performance and utility of transient elastography and noninvasive markers of liver fibrosis in primary biliary cirrhosis](http://www.sciencedirect.com/science/article/pii/S1590865811002258)
    1. Original research article
    2. Digestive and Liver Disease,
    3. Volume 43, Issue 11,
    4. November 2011,
    5. Pages 887-892
    6. Annarosa Floreani,
    7. Nora Cazzagon,
    8. Diego Martines,
    9. Luisa Cavalletto,
    10. Liliana Chemello
    11. [Purchase PDF](http://www.sciencedirect.com/science/article/pii/S1590865811002258)
12. [Predictors of Poor Prognosis in Recurrent Hepatitis C After Liver Transplantation](http://www.sciencedirect.com/science/article/pii/S0041134516305760)
    1. Original research article
    2. Transplantation Proceedings,
    3. Volume 48, Issue 9,
    4. November 2016,
    5. Pages 2997-2999
    6. E. Berge,
    7. E. Otón,
    8. Z. Reina,
    9. L. Díaz,
    10. F. Pérez
    11. [Purchase PDF](http://www.sciencedirect.com/science/article/pii/S0041134516305760)
13. [Non-alcoholic fatty liver disease and liver transplantation](http://www.sciencedirect.com/science/article/pii/S0026049516000524)
    1. Original research article
    2. Metabolism,
    3. Volume 65, Issue 8,
    4. August 2016,
    5. Pages 1208-1223
    6. Reenam S. Khan,
    7. Philip N. Newsome
    8. [Purchase PDF](http://www.sciencedirect.com/science/article/pii/S0026049516000524)
14. [Changes in Liver Congestion in Patients with Budd–Chiari Syndrome following Endovascular Interventions: Assessment with Transient Elastography](http://www.sciencedirect.com/science/article/pii/S1051044316308910)
    1. Original research article
    2. Journal of Vascular and Interventional Radiology,
    3. Volume 28, Issue 5,
    4. May 2017,
    5. Pages 683-687
    6. Amar Mukund,
    7. Sudheer S. Pargewar,
    8. Saloni N. Desai,
    9. S. Rajesh,
    10. Shiv K. Sarin
    11. [Purchase PDF](http://www.sciencedirect.com/science/article/pii/S1051044316308910)
15. [Sofosbuvir and Ribavirin for 24 Weeks Is An Effective Treatment Option for Recurrent Hepatitis C Infection After Living Donor Liver Transplantation](http://www.sciencedirect.com/science/article/pii/S097368831730004X)
    1. Original research article
    2. Journal of Clinical and Experimental Hepatology,
    3. In press, corrected proof,
    4. Available online 28 June 2017
    5. Anil C. Anand,
    6. Shaleen K. Agarwal,
    7. Hitendra K. Garg,
    8. Sudeep Khanna,
    9. Subhash Gupta
    10. [Purchase PDF](http://www.sciencedirect.com/science/article/pii/S097368831730004X)
16. [Factors associated with significant liver steatosis and fibrosis as assessed by transient elastography in patients with one or more components of the metabolic syndrome](http://www.sciencedirect.com/science/article/pii/S1056872716301672)
    1. Original research article
    2. Journal of Diabetes and its Complications,
    3. Volume 30, Issue 7,
    4. September–October 2016,
    5. Pages 1347-1353
    6. Ivana Mikolasevic,
    7. Sandra Milic,
    8. Lidija Orlic,
    9. Davor Stimac,
    10. Giovanni Targher
    11. [Purchase PDF](http://www.sciencedirect.com/science/article/pii/S1056872716301672)
17. [Transient elastography (FibroScan)](http://www.sciencedirect.com/science/article/pii/S0399832008739940)
    1. Original research article
    2. Gastroentérologie Clinique et Biologique,
    3. Volume 32, Issue 6, Supplement 1,
    4. September 2008,
    5. Pages 58-67
    6. V. de Lédinghen,
    7. J. Vergniol
    8. [Purchase PDF](http://www.sciencedirect.com/science/article/pii/S0399832008739940)
18. [Does transient elastography (FibroScan®) have a role in decision making in hepatocellular carcinoma?](http://www.sciencedirect.com/science/article/pii/S1365182X15305955)
    1. Open archive,
    2. Original research article
    3. HPB,
    4. Volume 14, Issue 6,
    5. June 2012,
    6. Pages 403-408
    7. Antonio Pesce,
    8. Roberto Scilletta,
    9. Angela Branca,
    10. Luciano Nigro,
    11. Stefano Puleo
    12. [Download PDF (155 KB)](http://www.sciencedirect.com/science/article/pii/S1365182X15305955/pdfft?md5=64dd0187ba44040255452a19247ab2cb&pid=1-s2.0-S1365182X15305955-main.pdf)
19. [Non-invasive evaluation of liver fibrosis using transient elastography](http://www.sciencedirect.com/science/article/pii/S0168827808001232)
    1. Original research article
    2. Journal of Hepatology,
    3. Volume 48, Issue 5,
    4. May 2008,
    5. Pages 835-847
    6. Laurent Castera,
    7. Xavier Forns,
    8. Alfredo Alberti
    9. [Purchase PDF](http://www.sciencedirect.com/science/article/pii/S0168827808001232)
20. [Immune monitoring of immunosuppression withdrawal of liver transplant recipients](http://www.sciencedirect.com/science/article/pii/S0966327415300113)
    1. Original research article
    2. Transplant Immunology,
    3. Volume 33, Issue 2,
    4. October 2015,
    5. Pages 110-116
    6. Rocío García de la Garza,
    7. Pablo Sarobe,
    8. Juana Merino,
    9. Juan J. Lasarte,
    10. J. Ignacio Herrero
    11. [Purchase PDF](http://www.sciencedirect.com/science/article/pii/S0966327415300113)
21. [Serum YKL-40 in young patients with β-thalassemia major: Relation to hepatitis C virus infection, liverstiffness by transient elastography and cardiovascular complications](http://www.sciencedirect.com/science/article/pii/S1079979615001722)
    1. Original research article
    2. Blood Cells, Molecules, and Diseases,
    3. Volume 56, Issue 1,
    4. January 2016,
    5. Pages 1-8
    6. Mohamed Abo El-Asrar,
    7. Nancy Samir Elbarbary,
    8. Eman Abdel Rahman Ismail,
    9. Ahmed Mohamed Elshenity
    10. [Purchase PDF](http://www.sciencedirect.com/science/article/pii/S1079979615001722)
22. [De Novo Donor-Specific Anti–Human Leukocyte Antigen Antibody Detection in Long-Term Adult LiverTransplantation](http://www.sciencedirect.com/science/article/pii/S0041134516305401)
    1. Original research article
    2. Transplantation Proceedings,
    3. Volume 48, Issue 9,
    4. November 2016,
    5. Pages 2980-2982
    6. D. San Segundo,
    7. C. Alonso,
    8. P. Ruiz,
    9. I. Roman,
    10. E. Fábrega
    11. [Purchase PDF](http://www.sciencedirect.com/science/article/pii/S0041134516305401)
23. [The role of transient elastography in patients with hepatitis B viral disease](http://www.sciencedirect.com/science/article/pii/S1590865810606895)
    1. Original research article
    2. Digestive and Liver Disease,
    3. Volume 43, Supplement 1,
    4. January 2011,
    5. Pages s25-s31
    6. Mirella Fraquelli,
    7. Federica Branchi
    8. [Purchase PDF](http://www.sciencedirect.com/science/article/pii/S1590865810606895)
24. [Which are the cut-off values of 2D-Shear Wave Elastography (2D-SWE) liver stiffness measurements predicting different stages of liver fibrosis, considering Transient Elastography (TE) as the reference method?](http://www.sciencedirect.com/science/article/pii/S0720048X13006542)
    1. Original research article
    2. European Journal of Radiology,
    3. Volume 83, Issue 3,
    4. March 2014,
    5. Pages e118-e122
    6. Ioan Sporea,
    7. Simona Bota,
    8. Oana Gradinaru-Taşcău,
    9. Roxana Şirli,
    10. Ana Jurchiş
    11. [Purchase PDF](http://www.sciencedirect.com/science/article/pii/S0720048X13006542)
25. [Diffusion weighted MRI and transient elastography assessment of liver fibrosis in hepatitis C patients: Validity of non invasive imaging techniques](http://www.sciencedirect.com/science/article/pii/S0378603X14000357)
    1. Open access,
    2. Original research article
    3. The Egyptian Journal of Radiology and Nuclear Medicine,
    4. Volume 45, Issue 2,
    5. June 2014,
    6. Pages 279-287
    7. Fatma Zaiton,
    8. Hitham Dawoud,
    9. Inas M. El Fiki,
    10. Khaled M. Hadhoud
    11. [Download PDF (1,234 KB)](http://www.sciencedirect.com/science/article/pii/S0378603X14000357/pdfft?md5=95c68aa1bd1316392a866c0429775938&pid=1-s2.0-S0378603X14000357-main.pdf)
26. [FibroScan Evaluation of Liver Fibrosis in Liver Transplantation](http://www.sciencedirect.com/science/article/pii/S004113450900164X)
    1. Original research article
    2. Transplantation Proceedings,
    3. Volume 41, Issue 3,
    4. April 2009,
    5. Pages 1044-1046
    6. G. Sánchez Antolin,
    7. F. Garcia Pajares,
    8. M.A. Vallecillo,
    9. P. Fernandez Orcajo,
    10. A. Caro-Patón
    11. [Purchase PDF](http://www.sciencedirect.com/science/article/pii/S004113450900164X)
27. [Staging chronic hepatitis C in seven categories using fibrosis biomarker (FibroTest™) and transientelastography (FibroScan®)](http://www.sciencedirect.com/science/article/pii/S0168827813008179)
    1. Original research article
    2. Journal of Hepatology,
    3. Volume 60, Issue 4,
    4. April 2014,
    5. Pages 706-714
    6. Thierry Poynard,
    7. Julien Vergniol,
    8. Yen Ngo,
    9. Juliette Foucher,
    10. Bordeaux HCV Study Group
    11. [Purchase PDF](http://www.sciencedirect.com/science/article/pii/S0168827813008179)
28. [Liver Transplantation for Alcoholic and Nonalcoholic Fatty Liver Disease: Pretransplant Selection and Posttransplant Management](http://www.sciencedirect.com/science/article/pii/S0016508516003036)
    1. Original research article
    2. Gastroenterology,
    3. Volume 150, Issue 8,
    4. June 2016,
    5. Pages 1849-1862
    6. M. Shadab Siddiqui,
    7. Michael Charlton
    8. [Purchase PDF](http://www.sciencedirect.com/science/article/pii/S0016508516003036)
29. [Noninvasive Assessment of Liver Fibrosis and Portal Hypertension With Transient Elastography](http://www.sciencedirect.com/science/article/pii/S0016508507021300)
    1. Original research article
    2. Gastroenterology,
    3. Volume 134, Issue 1,
    4. January 2008,
    5. Pages 8-14
    6. Don C. Rockey
    7. [Purchase PDF](http://www.sciencedirect.com/science/article/pii/S0016508507021300)
30. [Noninvasive assessment of liver fibrosis in patients with Fontan circulation using transient elastography and biochemical fibrosis markers](http://www.sciencedirect.com/science/article/pii/S0022522307016455)
    1. Open archive,
    2. Original research article
    3. The Journal of Thoracic and Cardiovascular Surgery,
    4. Volume 135, Issue 3,
    5. March 2008,
    6. Pages 560-567
    7. Mireen Friedrich-Rust,
    8. Constanze Koch,
    9. Axel Rentzsch,
    10. Christoph Sarrazin,
    11. Hashim Abdul-Khaliq
    12. [Download PDF (306 KB)](http://www.sciencedirect.com/science/article/pii/S0022522307016455/pdfft?md5=312bb4134412c0b353dd3540f537144d&pid=1-s2.0-S0022522307016455-main.pdf)
31. [Diffusion-weighted MRI versus transient elastography in quantification of liver fibrosis in patients with chronic cholestatic liver diseases](http://www.sciencedirect.com/science/article/pii/S0720048X11007674)
    1. Original research article
    2. European Journal of Radiology,
    3. Volume 81, Issue 10,
    4. October 2012,
    5. Pages 2500-2506
    6. Jelena Djokić Kovač,
    7. Marko Daković,
    8. Dejana Stanisavljević,
    9. Tamara Alempijević,
    10. Ružica Maksimović
    11. [Purchase PDF](http://www.sciencedirect.com/science/article/pii/S0720048X11007674)
32. [Discordance in fibrosis staging between liver biopsy and transient elastography using the FibroScan XL probe](http://www.sciencedirect.com/science/article/pii/S0168827811007823)
    1. Original research article
    2. Journal of Hepatology,
    3. Volume 56, Issue 3,
    4. March 2012,
    5. Pages 564-570
    6. Robert P. Myers,
    7. Gilles Pomier-Layrargues,
    8. Richard Kirsch,
    9. Aaron Pollett,
    10. Magdy Elkashab
    11. [Purchase PDF](http://www.sciencedirect.com/science/article/pii/S0168827811007823)
33. [Volatile Biomarkers in Breath Associated With Liver Cirrhosis — Comparisons of Pre- and Post-liverTransplant Breath Samples](http://www.sciencedirect.com/science/article/pii/S2352396415300797)
    1. Open access,
    2. Original research article
    3. EBioMedicine,
    4. Volume 2, Issue 9,
    5. September 2015,
    6. Pages 1243-1250
    7. R. Fernández del Río,
    8. M.E. O'Hara,
    9. A. Holt,
    10. P. Pemberton,
    11. C.A. Mayhew
    12. [Download PDF (450 KB)](http://www.sciencedirect.com/science/article/pii/S2352396415300797/pdfft?md5=b3c09d155d0ff9305ef0eb806e2f91e8&pid=1-s2.0-S2352396415300797-main.pdf)
34. [Ultrasound-Based Transient Elastography for the Detection of Hepatic Fibrosis: Systematic Review and Meta-analysis](http://www.sciencedirect.com/science/article/pii/S1542356507007537)
    1. Original research article
    2. Clinical Gastroenterology and Hepatology,
    3. Volume 5, Issue 10,
    4. October 2007,
    5. Pages 1214-1220
    6. Jayant A. Talwalkar,
    7. David M. Kurtz,
    8. Scott J. Schoenleber,
    9. Colin P. West,
    10. Victor M. Montori
    11. [Purchase PDF](http://www.sciencedirect.com/science/article/pii/S1542356507007537)
35. [Assessment of Fibrosis by Transient Elastography Compared With Liver Biopsy and Morphometry in Chronic Liver Diseases](http://www.sciencedirect.com/science/article/pii/S1542356508002000)
    1. Original research article
    2. Clinical Gastroenterology and Hepatology,
    3. Volume 6, Issue 9,
    4. September 2008,
    5. Pages 1027-1035
    6. Grace Lai–Hung Wong,
    7. Vincent Wai–Sun Wong,
    8. Paul Cheung–Lung Choi,
    9. Anthony Wing–Hung Chan,
    10. Henry Lik–Yuen Chan
    11. [Purchase PDF](http://www.sciencedirect.com/science/article/pii/S1542356508002000)
36. [MRI-guided Biopsy to Correlate Tissue Specimens with MR Elastography Stiffness Readings in LiverTransplants](http://www.sciencedirect.com/science/article/pii/S1076633212003133)
    1. Original research article
    2. Academic Radiology,
    3. Volume 19, Issue 9,
    4. September 2012,
    5. Pages 1121-1126
    6. Ryan B. Perumpail,
    7. Josh Levitsky,
    8. Yi Wang,
    9. Victoria S. Lee,
    10. Reed A. Omary
    11. [Purchase PDF](http://www.sciencedirect.com/science/article/pii/S1076633212003133)
37. [Performance of Transient Elastography for the Staging of Liver Fibrosis: A Meta-Analysis](http://www.sciencedirect.com/science/article/pii/S001650850800108X)
    1. Original research article
    2. Gastroenterology,
    3. Volume 134, Issue 4,
    4. April 2008,
    5. Pages 960-974.e8
    6. Mireen Friedrich–Rust,
    7. Mei–Fang Ong,
    8. Swantje Martens,
    9. Christoph Sarrazin,
    10. Eva Herrmann
    11. [Purchase PDF](http://www.sciencedirect.com/science/article/pii/S001650850800108X)
38. [Controversies in Liver Transplantation for Hepatitis C](http://www.sciencedirect.com/science/article/pii/S0016508508002862)
    1. Original research article
    2. Gastroenterology,
    3. Volume 134, Issue 6,
    4. May 2008,
    5. Pages 1777-1788
    6. Sandeep Mukherjee,
    7. Michael F. Sorrell
    8. [Purchase PDF](http://www.sciencedirect.com/science/article/pii/S0016508508002862)
39. [Hepatic Venous Congestion After Living Donor Liver Transplantation: Quantitative Assessment of LiverStiffness Using Shear Wave Elastography—A Case Report](http://www.sciencedirect.com/science/article/pii/S0041134512000693)
    1. Original research article
    2. Transplantation Proceedings,
    3. Volume 44, Issue 3,
    4. April 2012,
    5. Pages 814-816
    6. H.-K. Wang,
    7. Y.-C. Lai,
    8. H.-S. Tseng,
    9. R.-C. Lee,
    10. C.-Y. Chang
    11. [Purchase PDF](http://www.sciencedirect.com/science/article/pii/S0041134512000693)
40. [Magnetic Resonance Elastography vs Transient Elastography in Detection of Fibrosis and Noninvasive Measurement of Steatosis in Patients With Biopsy-Proven Nonalcoholic Fatty Liver Disease](http://www.sciencedirect.com/science/article/pii/S0016508516352738)
    1. Original research article
    2. Gastroenterology,
    3. Volume 152, Issue 3,
    4. February 2017,
    5. Pages 598-607.e2
    6. Charlie C. Park,
    7. Phirum Nguyen,
    8. Carolyn Hernandez,
    9. Ricki Bettencourt,
    10. Rohit Loomba
    11. [Purchase PDF](http://www.sciencedirect.com/science/article/pii/S0016508516352738)
41. [Viral Hepatitis in Liver Transplantation](http://www.sciencedirect.com/science/article/pii/S0016508512002235)
    1. Original research article
    2. Gastroenterology,
    3. Volume 142, Issue 6,
    4. May 2012,
    5. Pages 1373-1383.e1
    6. Gonzalo Crespo,
    7. Zoe Mariño,
    8. Miquel Navasa,
    9. Xavier Forns
    10. [Purchase PDF](http://www.sciencedirect.com/science/article/pii/S0016508512002235)
42. [The Acoustic Radiation Force Impulse Elastography Evaluation of Liver Fibrosis in Posttransplantation Dysfunction of Living Donor Liver Transplantation](http://www.sciencedirect.com/science/article/pii/S0041134513014085)
    1. Original research article
    2. Transplantation Proceedings,
    3. Volume 46, Issue 3,
    4. April 2014,
    5. Pages 876-879
    6. C.C. Liao,
    7. T.Y. Chen,
    8. L.C. Tsang,
    9. S.Y. Ou,
    10. T.L. Huang
    11. [Purchase PDF](http://www.sciencedirect.com/science/article/pii/S0041134513014085)
43. [Early detection in routine clinical practice of cirrhosis and oesophageal varices in chronic hepatitis C: Comparison of transient elastography (FibroScan) with standard laboratory tests and non-invasive scores](http://www.sciencedirect.com/science/article/pii/S0168827808006387)
    1. Original research article
    2. Journal of Hepatology,
    3. Volume 50, Issue 1,
    4. January 2009,
    5. Pages 59-68
    6. Laurent Castéra,
    7. Brigitte Le Bail,
    8. Françoise Roudot-Thoraval,
    9. Pierre-Henri Bernard,
    10. Victor de Lédinghen
    11. [Purchase PDF](http://www.sciencedirect.com/science/article/pii/S0168827808006387)
44. [Magnetic Resonance Imaging More Accurately Classifies Steatosis and Fibrosis in Patients With Nonalcoholic Fatty Liver Disease Than Transient Elastography](http://www.sciencedirect.com/science/article/pii/S0016508515017345)
    1. Open access,
    2. Original research article
    3. Gastroenterology,
    4. Volume 150, Issue 3,
    5. March 2016,
    6. Pages 626-637.e7
    7. Kento Imajo,
    8. Takaomi Kessoku,
    9. Yasushi Honda,
    10. Wataru Tomeno,
    11. Atsushi Nakajima
    12. [Download PDF (1,580 KB)](http://www.sciencedirect.com/science/article/pii/S0016508515017345/pdfft?md5=3127e601c346bb0639a230236cdacf22&pid=1-s2.0-S0016508515017345-main.pdf)
45. [Association Between Anthropometric Parameters and Measurements of Liver Stiffness by TransientElastography](http://www.sciencedirect.com/science/article/pii/S1542356512011408)
    1. Original research article
    2. Clinical Gastroenterology and Hepatology,
    3. Volume 11, Issue 3,
    4. March 2013,
    5. Pages 295-302.e3
    6. Grace Lai–Hung Wong,
    7. Henry Lik–Yuen Chan,
    8. Paul Cheung–Lung Choi,
    9. Anthony Wing–Hung Chan,
    10. Vincent Wai–Sun Wong
    11. [Purchase PDF](http://www.sciencedirect.com/science/article/pii/S1542356512011408)
46. [Association Between Level of Fibrosis, Rather Than Antiviral Regimen, and Outcomes of Patients With Chronic Hepatitis B](http://www.sciencedirect.com/science/article/pii/S1542356516303032)
    1. Original research article
    2. Clinical Gastroenterology and Hepatology,
    3. Volume 14, Issue 11,
    4. November 2016,
    5. Pages 1647-1656.e6
    6. Hye Soo Kim,
    7. Beom Kyung Kim,
    8. Seung Up Kim,
    9. Jun Yong Park,
    10. Sang Hoon Ahn
    11. [Purchase PDF](http://www.sciencedirect.com/science/article/pii/S1542356516303032)
47. [Non-invasive diagnosis of liver fibrosis in the transplant setting](http://www.sciencedirect.com/science/article/pii/S1594580411600211)
    1. Original research article
    2. Digestive and Liver Disease Supplements,
    3. Volume 5, Issue 1,
    4. December 2011,
    5. Pages 23-25
    6. Gonzalo Crespo,
    7. Zoe Mariño
    8. [Purchase PDF](http://www.sciencedirect.com/science/article/pii/S1594580411600211)
48. [Efficacy of Antiviral Therapy on Hepatitis C Recurrence After Liver Transplantation: A Randomized Controlled Study](http://www.sciencedirect.com/science/article/pii/S0016508507005653)
    1. Original research article
    2. Gastroenterology,
    3. Volume 132, Issue 5,
    4. May 2007,
    5. Pages 1746-1756
    6. José A. Carrión,
    7. Miquel Navasa,
    8. Montserrat García–Retortillo,
    9. Juan Carlos García–Pagan,
    10. Xavier Forns
    11. [Purchase PDF](http://www.sciencedirect.com/science/article/pii/S0016508507005653)
49. [Epidemiology and natural history of non-alcoholic fatty liver disease](http://www.sciencedirect.com/science/article/pii/S0026049516000275)
    1. Original research article
    2. Metabolism,
    3. Volume 65, Issue 8,
    4. August 2016,
    5. Pages 1017-1025
    6. Yousef Fazel,
    7. Aaron B. Koenig,
    8. Mehmet Sayiner,
    9. Zachary D. Goodman,
    10. Zobair M. Younossi
    11. [Purchase PDF](http://www.sciencedirect.com/science/article/pii/S0026049516000275)
50. [An association of large-fibre peripheral nerve dysfunction with non-invasive measures of liver fibrosis secondary to non-alcoholic fatty liver disease in diabetes](http://www.sciencedirect.com/science/article/pii/S1056872715002688)
    1. Original research article
    2. Journal of Diabetes and its Complications,
    3. Volume 29, Issue 8,
    4. November–December 2015,
    5. Pages 1240-1247
    6. Kathryn H. Williams,
    7. Kharis Burns,
    8. Maria Constantino,
    9. Nicholas A. Shackel,
    10. Stephen M. Twigg
    11. [Purchase PDF](http://www.sciencedirect.com/science/article/pii/S1056872715002688)
51. [Clinical effects of viral relapse after interferon plus ribavirin in patients co-infected with human immunodeficiency virus and hepatitis C virus](http://www.sciencedirect.com/science/article/pii/S0168827813000895)
    1. Original research article
    2. Journal of Hepatology,
    3. Volume 58, Issue 6,
    4. June 2013,
    5. Pages 1104-1112
    6. Juan Berenguer,
    7. Julio Alvarez-Pellicer,
    8. Ana Carrero,
    9. Miguel A. Von Wichmann,
    10. The GESIDA HIV/HCV Cohort Study Group
    11. [Purchase PDF](http://www.sciencedirect.com/science/article/pii/S0168827813000895)
52. [Baseline Values and Changes in Liver Stiffness Measured by Transient Elastography Are Associated With Severity of Fibrosis and Outcomes of Patients With Primary Sclerosing Cholangitis](http://www.sciencedirect.com/science/article/pii/S0016508513018441)
    1. Original research article
    2. Gastroenterology,
    3. Volume 146, Issue 4,
    4. April 2014,
    5. Pages 970-979.e6
    6. Christophe Corpechot,
    7. Farid Gaouar,
    8. Ahmed El Naggar,
    9. Astrid Kemgang,
    10. Olivier Chazouillères
    11. [Purchase PDF](http://www.sciencedirect.com/science/article/pii/S0016508513018441)
53. [Low Risk of Hepatocellular Carcinoma in Patients With Primary Sclerosing Cholangitis With Cirrhosis](http://www.sciencedirect.com/science/article/pii/S1542356514001980)
    1. Original research article
    2. Clinical Gastroenterology and Hepatology,
    3. Volume 12, Issue 10,
    4. October 2014,
    5. Pages 1733-1738
    6. Roman Zenouzi,
    7. Tobias J. Weismüller,
    8. Peter Hübener,
    9. Kornelius Schulze,
    10. Christoph Schramm
    11. [Purchase PDF](http://www.sciencedirect.com/science/article/pii/S1542356514001980)
54. [Features of Severe Liver Disease With Portal Hypertension in Patients With Cystic Fibrosis](http://www.sciencedirect.com/science/article/pii/S1542356516300167)
    1. Original research article
    2. Clinical Gastroenterology and Hepatology,
    3. Volume 14, Issue 8,
    4. August 2016,
    5. Pages 1207-1215.e3
    6. Jaclyn R. Stonebraker,
    7. Chee Y. Ooi,
    8. Rhonda G. Pace,
    9. Harriet Corvol,
    10. Simon C. Ling
    11. [Purchase PDF](http://www.sciencedirect.com/science/article/pii/S1542356516300167)
55. [Non-invasive assessment of hepatic fibrosis in a series of patients with Wilson's Disease](http://www.sciencedirect.com/science/article/pii/S1590865811004750)
    1. Original research article
    2. Digestive and Liver Disease,
    3. Volume 44, Issue 6,
    4. June 2012,
    5. Pages 487-491
    6. Margherita Sini,
    7. Orazio Sorbello,
    8. Alberto Civolani,
    9. Mauro Liggi,
    10. Luigi Demelia
    11. [Purchase PDF](http://www.sciencedirect.com/science/article/pii/S1590865811004750)
56. [Serum Fibrosis Markers Identify Patients With Mild and Progressive Hepatitis C Recurrence After LiverTransplantation](http://www.sciencedirect.com/science/article/pii/S0016508509016977)
    1. Original research article
    2. Gastroenterology,
    3. Volume 138, Issue 1,
    4. January 2010,
    5. Pages 147-158.e1
    6. José A. Carrión,
    7. Guillermo Fernández–Varo,
    8. Miquel Bruguera,
    9. Juan–Carlos García–Pagán,
    10. Miquel Navasa
    11. [Purchase PDF](http://www.sciencedirect.com/science/article/pii/S0016508509016977)
57. [Invasive and non-invasive methods for the assessment of fibrosis and disease progression in chronic liverdisease](http://www.sciencedirect.com/science/article/pii/S1521691811000291)
    1. Original research article
    2. Best Practice & Research Clinical Gastroenterology,
    3. Volume 25, Issue 2,
    4. April 2011,
    5. Pages 291-303
    6. Laurent Castera
    7. [Purchase PDF](http://www.sciencedirect.com/science/article/pii/S1521691811000291)
58. [Differences in liver stiffness values obtained with new ultrasound elastography machines and Fibroscan: A comparative study](http://www.sciencedirect.com/science/article/pii/S1590865817302463)
    1. Original research article
    2. Digestive and Liver Disease,
    3. Volume 49, Issue 7,
    4. July 2017,
    5. Pages 802-808
    6. Fabio Piscaglia,
    7. Veronica Salvatore,
    8. Lorenzo Mulazzani,
    9. Vito Cantisani,
    10. Luigi Bolondi
    11. [Purchase PDF](http://www.sciencedirect.com/science/article/pii/S1590865817302463)
59. [ARFI, FibroScan®, ELF, and their combinations in the assessment of liver fibrosis: A prospective study](http://www.sciencedirect.com/science/article/pii/S0168827812002711)
    1. Original research article
    2. Journal of Hepatology,
    3. Volume 57, Issue 2,
    4. August 2012,
    5. Pages 281-287
    6. Gonzalo Crespo,
    7. Guillermo Fernández-Varo,
    8. Zoe Mariño,
    9. Gregori Casals,
    10. Miquel Navasa
    11. [Purchase PDF](http://www.sciencedirect.com/science/article/pii/S0168827812002711)
60. [Hepatic steatosis progresses faster in HIV mono-infected than HIV/HCV co-infected patients and is associated with liver fibrosis](http://www.sciencedirect.com/science/article/pii/S0168827817320470)
    1. Original research article
    2. Journal of Hepatology,
    3. In press, corrected proof,
    4. Available online 18 May 2017
    5. Thomas Pembroke,
    6. Marc Deschenes,
    7. Bertrand Lebouché,
    8. Amine Benmassaoud,
    9. Giada Sebastiani
    10. [Purchase PDF](http://www.sciencedirect.com/science/article/pii/S0168827817320470)
61. [Early Acute Severe HCV Recurrence After Transplantation: From Universal Mortality to Cure](http://www.sciencedirect.com/science/article/pii/S0973688316302420)
    1. Original research article
    2. Journal of Clinical and Experimental Hepatology,
    3. Volume 7, Issue 1,
    4. March 2017,
    5. Pages 28-32
    6. Manav Wadhawan,
    7. Vivek Vij,
    8. Kausar Makki,
    9. Nalini Bansal,
    10. Ajay Kumar
    11. [Purchase PDF](http://www.sciencedirect.com/science/article/pii/S0973688316302420)
62. [Progressive Familial Intrahepatic Cholestasis (PFIC) in Indian Children: Clinical Spectrum and Outcome](http://www.sciencedirect.com/science/article/pii/S0973688316300585)
    1. Original research article
    2. Journal of Clinical and Experimental Hepatology,
    3. Volume 6, Issue 3,
    4. September 2016,
    5. Pages 203-208
    6. Sajan Agarwal,
    7. Bikrant Bihari Lal,
    8. Dinesh Rawat,
    9. Archana Rastogi,
    10. Seema Alam
    11. [Purchase PDF](http://www.sciencedirect.com/science/article/pii/S0973688316300585)
63. [Basal values and changes of liver stiffness predict the risk of disease progression in compensated advanced chronic liver disease](http://www.sciencedirect.com/science/article/pii/S1590865816304789)
    1. Original research article
    2. Digestive and Liver Disease,
    3. Volume 48, Issue 10,
    4. October 2016,
    5. Pages 1214-1219
    6. Mònica Pons,
    7. Macarena Simón-Talero,
    8. Laura Millán,
    9. Meritxell Ventura-Cots,
    10. Joan Genescà
    11. [Purchase PDF](http://www.sciencedirect.com/science/article/pii/S1590865816304789)
64. [Twenty-year protocol liver biopsies: Invasive but useful for the management of liver recipients](http://www.sciencedirect.com/science/article/pii/S0168827811008695)
    1. Original research article
    2. Journal of Hepatology,
    3. Volume 56, Issue 4,
    4. April 2012,
    5. Pages 840-847
    6. Mylène Sebagh,
    7. Didier Samuel,
    8. Teresa Maria Antonini,
    9. Audrey Coilly,
    10. Daniel Azoulay
    11. [Purchase PDF](http://www.sciencedirect.com/science/article/pii/S0168827811008695)
65. [Evaluating the risk of hepatocellular carcinoma in patients with prominently elevated liver stiffness measurements by FibroScan: a multicentre study](http://www.sciencedirect.com/science/article/pii/S1365182X16317543)
    1. Open archive,
    2. Original research article
    3. HPB,
    4. Volume 18, Issue 8,
    5. August 2016,
    6. Pages 678-683
    7. Maciej Adler,
    8. Licia Larocca,
    9. Francesca M. Trovato,
    10. Heather Marcinkowski,
    11. Simon D. Taylor-Robinson
    12. [Download PDF (372 KB)](http://www.sciencedirect.com/science/article/pii/S1365182X16317543/pdfft?md5=b6a889711465101f90555aaf0cb56179&pid=1-s2.0-S1365182X16317543-main.pdf)
66. [Etiology-related determinants of liver stiffness values in chronic viral hepatitis B or C](http://www.sciencedirect.com/science/article/pii/S0168827810008056)
    1. Original research article
    2. Journal of Hepatology,
    3. Volume 54, Issue 4,
    4. April 2011,
    5. Pages 621-628
    6. Mirella Fraquelli,
    7. Cristina Rigamonti,
    8. Giovanni Casazza,
    9. Maria Francesca Donato,
    10. Massimo Colombo
    11. [Purchase PDF](http://www.sciencedirect.com/science/article/pii/S0168827810008056)
67. [Optimising risk stratification in primary biliary cirrhosis: AST/platelet ratio index predicts outcome independent of ursodeoxycholic acid response](http://www.sciencedirect.com/science/article/pii/S0168827814001056)
    1. Original research article
    2. Journal of Hepatology,
    3. Volume 60, Issue 6,
    4. June 2014,
    5. Pages 1249-1258
    6. Palak J. Trivedi,
    7. Tony Bruns,
    8. Angela Cheung,
    9. Ka-Kit Li,
    10. Gideon M. Hirschfield
    11. [Purchase PDF](http://www.sciencedirect.com/science/article/pii/S0168827814001056)
68. [Relationship between hepatic haemodynamics assessed by Doppler ultrasound and liver stiffness](http://www.sciencedirect.com/science/article/pii/S1590865811003306)
    1. Original research article
    2. Digestive and Liver Disease,
    3. Volume 44, Issue 2,
    4. February 2012,
    5. Pages 154-159
    6. Veronica Salvatore,
    7. Alberto Borghi,
    8. Eugenia Peri,
    9. Antonio Colecchia,
    10. Luigi Bolondi
    11. [Purchase PDF](http://www.sciencedirect.com/science/article/pii/S1590865811003306)
69. [Treatment of hepatitis C in the post-transplant setting](http://www.sciencedirect.com/science/article/pii/S1594580411600223)
    1. Original research article
    2. Digestive and Liver Disease Supplements,
    3. Volume 5, Issue 1,
    4. December 2011,
    5. Pages 26-29
    6. Marina Berenguer
    7. [Purchase PDF](http://www.sciencedirect.com/science/article/pii/S1594580411600223)
70. [Ultrasound elastography and contrast-enhanced ultrasound in infants, children and adolescents](http://www.sciencedirect.com/science/article/pii/S0720048X14003258)
    1. Original research article
    2. European Journal of Radiology,
    3. Volume 83, Issue 9,
    4. September 2014,
    5. Pages 1560-1569
    6. Martin Stenzel,
    7. Hans-Joachim Mentzel
    8. [Purchase PDF](http://www.sciencedirect.com/science/article/pii/S0720048X14003258)
71. [Marcadores séricos de fibrosis hepática en pacientes con hepatitis crónica C. Valor pronóstico de los marcadores no invasivos de fibrosis en el trasplante de hígado](http://www.sciencedirect.com/science/article/pii/S0210570512700456)
    1. Original research article
    2. Gastroenterología y Hepatología,
    3. Volume 35, Supplement 2,
    4. December 2012,
    5. Pages 17-22
    6. Gonzalo Crespo,
    7. Xavier Forns,
    8. Miquel Navasa
    9. [Purchase PDF](http://www.sciencedirect.com/science/article/pii/S0210570512700456)
72. [Alcoholic steatohepatitis](http://www.sciencedirect.com/science/article/pii/S152169181000082X)
    1. Original research article
    2. Best Practice & Research Clinical Gastroenterology,
    3. Volume 24, Issue 5,
    4. October 2010,
    5. Pages 683-693
    6. Felix Stickel,
    7. Helmut K. Seitz
    8. [Purchase PDF](http://www.sciencedirect.com/science/article/pii/S152169181000082X)
73. [Non-invasive diagnosis and monitoring of liver fibrosis and cirrhosis](http://www.sciencedirect.com/science/article/pii/S1521691809000390)
    1. Original research article
    2. Best Practice & Research Clinical Gastroenterology,
    3. Volume 23, Issue 3,
    4. June 2009,
    5. Pages 453-460
    6. Ulrike W. Denzer,
    7. Stefan Lüth
    8. [Purchase PDF](http://www.sciencedirect.com/science/article/pii/S1521691809000390)
74. [Incidence of non-alcoholic fatty liver disease in Hong Kong: A population study with paired proton-magnetic resonance spectroscopy](http://www.sciencedirect.com/science/article/pii/S0168827814006291)
    1. Original research article
    2. Journal of Hepatology,
    3. Volume 62, Issue 1,
    4. January 2015,
    5. Pages 182-189
    6. Vincent Wai-Sun Wong,
    7. Grace Lai-Hung Wong,
    8. David Ka-Wai Yeung,
    9. Tina Kit-Ting Lau,
    10. Henry Lik-Yuen Chan
    11. [Purchase PDF](http://www.sciencedirect.com/science/article/pii/S0168827814006291)
75. [Shear wave elastography: An accurate technique to stage liver fibrosis in chronic liver diseases](http://www.sciencedirect.com/science/article/pii/S221156841500368X)
    1. Open archive,
    2. Original research article
    3. Diagnostic and Interventional Imaging,
    4. Volume 97, Issue 1,
    5. January 2016,
    6. Pages 91-99
    7. A. Guibal,
    8. G. Renosi,
    9. A. Rode,
    10. J.Y. Scoazec,
    11. T. Lefort
    12. [Download PDF (1,417 KB)](http://www.sciencedirect.com/science/article/pii/S221156841500368X/pdfft?md5=f08b7dfcebc937255a99dfe8b90be478&pid=1-s2.0-S221156841500368X-main.pdf)
76. [Levels of Alanine Aminotransferase Confound Use of Transient Elastography to Diagnose Fibrosis in Patients With Chronic Hepatitis C Virus Infection](http://www.sciencedirect.com/science/article/pii/S1542356512001140)
    1. Original research article
    2. Clinical Gastroenterology and Hepatology,
    3. Volume 10, Issue 8,
    4. August 2012,
    5. Pages 932-937.e1
    6. Elliot B. Tapper,
    7. Eric B. Cohen,
    8. Keyur Patel,
    9. Bruce Bacon,
    10. Nezam Afdhal
    11. [Purchase PDF](http://www.sciencedirect.com/science/article/pii/S1542356512001140)
77. [Meal ingestion markedly increases liver stiffness suggesting the need for liver stiffness determination in fasting conditions](http://www.sciencedirect.com/science/article/pii/S0210570515000291)
    1. Original research article
    2. Gastroenterología y Hepatología,
    3. Volume 38, Issue 7,
    4. August–September 2015,
    5. Pages 431-435
    6. Daniel Alvarez,
    7. Federico Orozco,
    8. José María Mella,
    9. Maria Anders,
    10. Ricardo Mastai
    11. [Purchase PDF](http://www.sciencedirect.com/science/article/pii/S0210570515000291)
78. [Efficacy and tolerability of interferon-free antiviral therapy in kidney transplant recipients with chronic hepatitis C](http://www.sciencedirect.com/science/article/pii/S016882781630753X)
    1. Original research article
    2. Journal of Hepatology,
    3. Volume 66, Issue 4,
    4. April 2017,
    5. Pages 718-723
    6. Inmaculada Fernández,
    7. Raquel Muñoz-Gómez,
    8. Juan M. Pascasio,
    9. Carme Baliellas,
    10. María-Carlota Londoño
    11. [Purchase PDF](http://www.sciencedirect.com/science/article/pii/S016882781630753X)
79. [Staging chronic hepatitis B into seven categories, defining inactive carriers and assessing treatment impact using a fibrosis biomarker (FibroTest®) and elastography (FibroScan®)](http://www.sciencedirect.com/science/article/pii/S0168827814004577)
    1. Original research article
    2. Journal of Hepatology,
    3. Volume 61, Issue 5,
    4. November 2014,
    5. Pages 994-1003
    6. Thierry Poynard,
    7. Julien Vergniol,
    8. Yen Ngo,
    9. Juliette Foucher,
    10. FibroFrance Study Group and the Bordeaux HBV Study Group
    11. [Purchase PDF](http://www.sciencedirect.com/science/article/pii/S0168827814004577)
80. [Thalassemia major between liver and heart: Where we are now](http://www.sciencedirect.com/science/article/pii/S1079979615000704)
    1. Original research article
    2. Blood Cells, Molecules, and Diseases,
    3. Volume 55, Issue 1,
    4. June 2015,
    5. Pages 82-88
    6. Carlo Dessì,
    7. GiovanBattista Leoni,
    8. Paolo Moi,
    9. Fabrice Danjou,
    10. Raffaella Origa
    11. [Purchase PDF](http://www.sciencedirect.com/science/article/pii/S1079979615000704)
81. [The long-term benefits of nucleos(t)ide analogs in compensated HBV cirrhotic patients with no or small esophageal varices: A 12-year prospective cohort study](http://www.sciencedirect.com/science/article/pii/S0168827815003979)
    1. Original research article
    2. Journal of Hepatology,
    3. Volume 63, Issue 5,
    4. November 2015,
    5. Pages 1118-1125
    6. Pietro Lampertico,
    7. Federica Invernizzi,
    8. Mauro Viganò,
    9. Alessandro Loglio,
    10. Massimo Colombo
    11. [Purchase PDF](http://www.sciencedirect.com/science/article/pii/S0168827815003979)
82. [A pilot study of autologous CD34-depleted bone marrow mononuclear cell transplantation via the hepatic artery in five patients with liver failure](http://www.sciencedirect.com/science/article/pii/S1465324913005458)
    1. Original research article
    2. Cytotherapy,
    3. Volume 15, Issue 12,
    4. December 2013,
    5. Pages 1571-1579
    6. Chung-Hwa Park,
    7. Si Hyun Bae,
    8. Hee Yeon Kim,
    9. Ja Kyung Kim,
    10. Young Sok Lee
    11. [Purchase PDF](http://www.sciencedirect.com/science/article/pii/S1465324913005458)
83. [Soluble receptor for advanced glycation end products and liver stiffness in postoperative biliary atresia](http://www.sciencedirect.com/science/article/pii/S0009912012006315)
    1. Original research article
    2. Clinical Biochemistry,
    3. Volume 46, Issue 3,
    4. February 2013,
    5. Pages 214-218
    6. Sittisak Honsawek,
    7. Paisarn Vejchapipat,
    8. Sunchai Payungporn,
    9. Apiradee Theamboonlers,
    10. Yong Poovorawan
    11. [Purchase PDF](http://www.sciencedirect.com/science/article/pii/S0009912012006315)
84. [Severity of cirrhosis should determine the operative modality for patients with early hepatocellular carcinoma and compensated liver function](http://www.sciencedirect.com/science/article/pii/S0039606015007291)
    1. Original research article
    2. Surgery,
    3. Volume 159, Issue 2,
    4. February 2016,
    5. Pages 621-631
    6. Zhi-yong Huang,
    7. Bin-yong Liang,
    8. Min Xiong,
    9. Ke-shuai Dong,
    10. Xiao-ping Chen
    11. [Purchase PDF](http://www.sciencedirect.com/science/article/pii/S0039606015007291)
85. [Real-time shear-wave elastography: Applicability, reliability and accuracy for clinically significant portal hypertension](http://www.sciencedirect.com/science/article/pii/S0168827814009258)
    1. Original research article
    2. Journal of Hepatology,
    3. Volume 62, Issue 5,
    4. May 2015,
    5. Pages 1068-1075
    6. Bogdan Procopet,
    7. Annalisa Berzigotti,
    8. Juan G. Abraldes,
    9. Fanny Turon,
    10. Jaime Bosch
    11. [Purchase PDF](http://www.sciencedirect.com/science/article/pii/S0168827814009258)
86. [Lactic acidosis in patients with hepatitis C virus cirrhosis and combined ribavirin/sofosbuvir treatment](http://www.sciencedirect.com/science/article/pii/S0168827815007928)
    1. Original research article
    2. Journal of Hepatology,
    3. Volume 64, Issue 4,
    4. April 2016,
    5. Pages 790-799
    6. Martin-Walter Welker,
    7. Stefan Luhne,
    8. Christian M. Lange,
    9. Johannes Vermehren,
    10. Christoph Sarrazin
    11. [Purchase PDF](http://www.sciencedirect.com/science/article/pii/S0168827815007928)
87. [How to Approach a Patient With Nonalcoholic Fatty Liver Disease](http://www.sciencedirect.com/science/article/pii/S0016508517358018)
    1. Original research article
    2. Gastroenterology,
    3. Volume 153, Issue 2,
    4. August 2017,
    5. Pages 345-349
    6. Herbert Tilg
    7. [Purchase PDF](http://www.sciencedirect.com/science/article/pii/S0016508517358018)
88. [Role of Nurse Practitioners in the Management of Cirrhotic Patients](http://www.sciencedirect.com/science/article/pii/S1555415512004503)
    1. Original research article
    2. The Journal for Nurse Practitioners,
    3. Volume 8, Issue 10,
    4. November–December 2012,
    5. Pages 816-821
    6. Kristina Tuesday Werner,
    7. Shari Terese Perez
    8. [Purchase PDF](http://www.sciencedirect.com/science/article/pii/S1555415512004503)
89. [A pilot study of ultrasound elastography as a non-invasive method to monitor liver disease in children with short bowel syndrome](http://www.sciencedirect.com/science/article/pii/S0022346817301653)
    1. Original research article
    2. Journal of Pediatric Surgery,
    3. Volume 52, Issue 6,
    4. June 2017,
    5. Pages 962-965
    6. Daniel Lodwick,
    7. Molly Dienhart,
    8. Jennifer N. Cooper,
    9. Bonita Fung,
    10. Peter C. Minneci
    11. [Purchase PDF](http://www.sciencedirect.com/science/article/pii/S0022346817301653)
90. [The degree of spleen stiffness measured on acoustic radiation force impulse elastography predicts the severity of portal hypertension in patients with biliary atresia after portoenterostomy](http://www.sciencedirect.com/science/article/pii/S0022346815000433)
    1. Original research article
    2. Journal of Pediatric Surgery,
    3. Volume 50, Issue 4,
    4. April 2015,
    5. Pages 559-564
    6. Hajime Uchida,
    7. Seisuke Sakamoto,
    8. Megumi Kobayashi,
    9. Takanobu Shigeta,
    10. Mureo Kasahara
    11. [Purchase PDF](http://www.sciencedirect.com/science/article/pii/S0022346815000433)
91. [Best practice guidance for the diagnosis and management of cystic fibrosis-associated liver disease](http://www.sciencedirect.com/science/article/pii/S1569199311600064)
    1. Open archive,
    2. Original research article
    3. Journal of Cystic Fibrosis,
    4. Volume 10, Supplement 2,
    5. June 2011,
    6. Pages s29-s36
    7. Dominique Debray,
    8. Deirdre Kelly,
    9. Roderick Houwen,
    10. Birgitta Strandvik,
    11. Carla Colombo
    12. [Download PDF (226 KB)](http://www.sciencedirect.com/science/article/pii/S1569199311600064/pdfft?md5=344deaae84df78163441bf4b0aa9439b&pid=1-s2.0-S1569199311600064-main.pdf)
92. [Measurement of Spleen Stiffness With Acoustic Radiation Force Impulse Imaging Predicts Mortality and Hepatic Decompensation in Patients With Liver Cirrhosis](http://www.sciencedirect.com/science/article/pii/S1542356516312344)
    1. Original research article
    2. Clinical Gastroenterology and Hepatology,
    3. In press, uncorrected proof,
    4. Available online 23 December 2016
    5. Yoshitaka Takuma,
    6. Youichi Morimoto,
    7. Hiroyuki Takabatake,
    8. Nobuyuki Toshikuni,
    9. Hiroshi Yamamoto
    10. [Purchase PDF](http://www.sciencedirect.com/science/article/pii/S1542356516312344)
93. [Early occurrence and recurrence of hepatocellular carcinoma in HCV-related cirrhosis treated with direct-acting antivirals](http://www.sciencedirect.com/science/article/pii/S0168827816303038)
    1. Original research article
    2. Journal of Hepatology,
    3. Volume 65, Issue 4,
    4. October 2016,
    5. Pages 727-733
    6. Fabio Conti,
    7. Federica Buonfiglioli,
    8. Alessandra Scuteri,
    9. Cristina Crespi,
    10. Stefano Brillanti
    11. [Purchase PDF](http://www.sciencedirect.com/science/article/pii/S0168827816303038)
94. [Lower serum fibroblast activation protein shows promise in the exclusion of clinically significant liverfibrosis due to non-alcoholic fatty liver disease in diabetes and obesity](http://www.sciencedirect.com/science/article/pii/S0168822715001242)
    1. Original research article
    2. Diabetes Research and Clinical Practice,
    3. Volume 108, Issue 3,
    4. June 2015,
    5. Pages 466-472
    6. K.H. Williams,
    7. A.J. Viera de Ribeiro,
    8. E. Prakoso,
    9. A.S. Veillard,
    10. M.D. Gorrell
    11. [Purchase PDF](http://www.sciencedirect.com/science/article/pii/S0168822715001242)
95. [Hepatitis B Virus Genotype C Is Associated With More Severe Liver Fibrosis Than Genotype B](http://www.sciencedirect.com/science/article/pii/S1542356509007617)
    1. Original research article
    2. Clinical Gastroenterology and Hepatology,
    3. Volume 7, Issue 12,
    4. December 2009,
    5. Pages 1361-1366
    6. Henry Lik–Yuen Chan,
    7. Grace Lai–Hung Wong,
    8. Chi–Hang Tse,
    9. Angel Mei–Ling Chim,
    10. Vincent Wai–Sun Wong
    11. [Purchase PDF](http://www.sciencedirect.com/science/article/pii/S1542356509007617)
96. [Alcohol intake increases the risk of HCC in hepatitis C virus-related compensated cirrhosis: A prospective study](http://www.sciencedirect.com/science/article/pii/S0168827816301842)
    1. Original research article
    2. Journal of Hepatology,
    3. Volume 65, Issue 3,
    4. September 2016,
    5. Pages 543-551
    6. Hélène Vandenbulcke,
    7. Christophe Moreno,
    8. Isabelle Colle,
    9. Jean-François Knebel,
    10. Pierre Deltenre
    11. [Purchase PDF](http://www.sciencedirect.com/science/article/pii/S0168827816301842)
97. [Practice guidelines for the diagnosis and management of nonalcoholic fatty liver disease: A decalogue from the Italian Association for the Study of the Liver (AISF) Expert Committee](http://www.sciencedirect.com/science/article/pii/S1590865810000459)
    1. Original research article
    2. Digestive and Liver Disease,
    3. Volume 42, Issue 4,
    4. April 2010,
    5. Pages 272-282
    6. P. Loria,
    7. L.E. Adinolfi,
    8. S. Bellentani,
    9. E. Bugianesi,
    10. The NAFLD Expert Committee of the Associazione Italiana per lo studio del Fegato (AISF)
    11. [Purchase PDF](http://www.sciencedirect.com/science/article/pii/S1590865810000459)
98. [Association Between Severe Portal Hypertension and Risk of Liver Decompensation in Patients With Hepatitis C, Regardless of Response to Antiviral Therapy](http://www.sciencedirect.com/science/article/pii/S1542356515004152)
    1. Original research article
    2. Clinical Gastroenterology and Hepatology,
    3. Volume 13, Issue 10,
    4. October 2015,
    5. Pages 1846-1853.e1
    6. Sabela Lens,
    7. Diego Rincón,
    8. Montserrat García-Retortillo,
    9. Agustín Albillos,
    10. Juan Carlos García-Pagán
    11. [Purchase PDF](http://www.sciencedirect.com/science/article/pii/S1542356515004152)
99. [25](http://www.sciencedirect.com/search?qs=%22Liver%20transplantation%22%20and%20%22transient%20elastography%22&show=25&sortBy=relevance&articleTypes=OR)
100. [50](http://www.sciencedirect.com/search?qs=%22Liver%20transplantation%22%20and%20%22transient%20elastography%22&show=50&sortBy=relevance&articleTypes=OR)
101. 100
102. Page 1 of 3
103. [next](http://www.sciencedirect.com/search?qs=%22Liver%20transplantation%22%20and%20%22transient%20elastography%22&show=100&sortBy=relevance&articleTypes=OR&offset=100)

[Feedback](http://www.sciencedirect.com/search?qs=%22Liver%20transplantation%22%20and%20%22transient%20elastography%22&show=100&sortBy=relevance&articleTypes=OR&lastSelectedFacet=articleTypes)

[About ScienceDirect](http://www.elsevier.com/solutions/sciencedirect)[Remote access](http://www.sciencedirect.com/science/activateaccess)[Shopping cart](http://www.sciencedirect.com/science?_ob=ShoppingCartURL&_method=display&_zone=TopNavBar&_origin=srp&md5=e5857d1631c4b1f612897ed029064507)[Contact and support](https://service.elsevier.com/app/contact/supporthub/sciencedirect/)[Terms and conditions](https://www.elsevier.com/legal/elsevier-website-terms-and-conditions)[Privacy policy](http://www.elsevier.com/legal/privacy-policy)

Cookies are used by this site. For more information, visit the [cookies page](https://www.elsevier.com/solutions/sciencedirect/support/cookies).

Copyright © 2017 Elsevier B.V. or its licensors or contributors. ScienceDirect ® is a registered trademark of Elsevier B.V.

[Skip to Main content](http://www.sciencedirect.com/search?qs=%22Liver%20transplantation%22%20and%20%22transient%20elastography%22&show=100&sortBy=relevance&articleTypes=OR&offset=100#main_content)

- [Journals](http://www.sciencedirect.com/science/journals)
- [Books](http://www.sciencedirect.com/science/bookbshsrw)
- [Register](https://www.sciencedirect.com/user/register?returnURL=http%3A%2F%2Fwww.sciencedirect.com%2Fsearch)
- [Sign in](https://www.sciencedirect.com/user/login?returnURL=http%3A%2F%2Fwww.sciencedirect.com%2Fsearch)

Top of Form

[Advanced search](http://www.sciencedirect.com/science/search)

Bottom of Form

252 results

Refine by:

Top of Form

Years

1. 2017 (35)
2. 2016 (48)
3. 2015 (36)
4. 2014 (23)
5. 2013 (15)
6. 2012 (28)
7. 2011 (18)
8. 2010 (14)
9. 2009 (13)
10. 2008 (15)

Show less

Article type

1. Original research (252)

Publication title

1. Journal of Hepatology (53)
2. Clinical Gastroenterology and Hepatology (24)
3. Gastroenterology (22)
4. Digestive and Liver Disease (19)
5. Transplantation Proceedings (9)
6. Ultrasound in Medicine & Biology (8)
7. European Journal of Radiology (6)
8. Metabolism (6)
9. Best Practice & Research Clinical Gastroenterology (6)
10. Gastroenterología y Hepatología (5)

Show less

[Clear all filters](http://www.sciencedirect.com/search?qs=%22Liver%20transplantation%22%20and%20%22transient%20elastography%22&show=100&sortBy=relevance&offset=100&navigation=true)

Bottom of Form

sorted by *relevance* | [date](http://www.sciencedirect.com/search?qs=%22Liver%20transplantation%22%20and%20%22transient%20elastography%22&show=100&sortBy=date&articleTypes=OR&offset=100&navigation=true)

1. [Clinical Factors Associated With Liver Stiffness in Hepatitis B e Antigen–Positive Chronic Hepatitis B Patients](http://www.sciencedirect.com/science/article/pii/S1542356508011075)
   1. Original research article
   2. Clinical Gastroenterology and Hepatology,
   3. Volume 7, Issue 2,
   4. February 2009,
   5. Pages 227-233
   6. Grace Lai–Hung Wong,
   7. Vincent Wai–Sun Wong,
   8. Paul Cheung–Lung Choi,
   9. Anthony Wing–Hung Chan,
   10. Henry Lik–Yuen Chan
   11. [Purchase PDF](http://www.sciencedirect.com/science/article/pii/S1542356508011075)
2. [Exercise and diet in the management of nonalcoholic fatty liver disease](http://www.sciencedirect.com/science/article/pii/S002604951500339X)
   1. Original research article
   2. Metabolism,
   3. Volume 65, Issue 8,
   4. August 2016,
   5. Pages 1172-1182
   6. Suzanne E. Mahady,
   7. Jacob George
   8. [Purchase PDF](http://www.sciencedirect.com/science/article/pii/S002604951500339X)
3. Want a richer search experience?

Sign in for additional filter options, multiple article downloads, and more.

Top of Form

Sign in

Bottom of Form

1. [Fibrosis index based on four factors better predicts advanced fibrosis or cirrhosis than aspartate aminotransferase/platelet ratio index in chronic hepatitis C patients](http://www.sciencedirect.com/science/article/pii/S0929664615002417)
   1. Open archive,
   2. Original research article
   3. Journal of the Formosan Medical Association,
   4. Volume 114, Issue 10,
   5. October 2015,
   6. Pages 923-928
   7. Chia-Chi Wang,
   8. Chen-Hua Liu,
   9. Chih-Lin Lin,
   10. Pin-Chao Wang,
   11. Jia-Horng Kao
   12. [Download PDF (375 KB)](http://www.sciencedirect.com/science/article/pii/S0929664615002417/pdfft?md5=68465eac6e84326a9e1d1acb9f5c709f&pid=1-s2.0-S0929664615002417-main.pdf)
2. [Liver stiffness accurately predicts portal hypertension related complications in patients with chronic liverdisease: A prospective study](http://www.sciencedirect.com/science/article/pii/S0168827811002017)
   1. Original research article
   2. Journal of Hepatology,
   3. Volume 55, Issue 5,
   4. November 2011,
   5. Pages 1017-1024
   6. Marie Angèle Robic,
   7. Bogdan Procopet,
   8. Sophie Métivier,
   9. Jean Marie Péron,
   10. Christophe Bureau
   11. [Purchase PDF](http://www.sciencedirect.com/science/article/pii/S0168827811002017)
3. [Development and Validation of a Scoring System to Predict Outcomes of Patients With Primary Biliary Cirrhosis Receiving Ursodeoxycholic Acid Therapy](http://www.sciencedirect.com/science/article/pii/S001650851501094X)
   1. Original research article
   2. Gastroenterology,
   3. Volume 149, Issue 7,
   4. December 2015,
   5. Pages 1804-1812.e4
   6. Willem J. Lammers,
   7. Gideon M. Hirschfield,
   8. Christophe Corpechot,
   9. Frederik Nevens,
   10. Global PBC Study Group
   11. [Purchase PDF](http://www.sciencedirect.com/science/article/pii/S001650851501094X)
4. [Non invasive diagnosis of portal hypertension in cirrhotic patients](http://www.sciencedirect.com/science/article/pii/S0399832008739976)
   1. Original research article
   2. Gastroentérologie Clinique et Biologique,
   3. Volume 32, Issue 6, Supplement 1,
   4. September 2008,
   5. Pages 80-87
   6. F. Vizzutti,
   7. U. Arena,
   8. L. Rega,
   9. M. Pinzani
   10. [Purchase PDF](http://www.sciencedirect.com/science/article/pii/S0399832008739976)
5. [Prospective screening increases the detection of potentially curable hepatocellular carcinoma: results in 8900 high-risk patients](http://www.sciencedirect.com/science/article/pii/S1365182X15313514)
   1. Open archive,
   2. Original research article
   3. HPB,
   4. Volume 15, Issue 12,
   5. December 2013,
   6. Pages 985-990
   7. Francesco Izzo,
   8. Mauro Piccirillo,
   9. Vittorio Albino,
   10. Raffaele Palaia,
   11. Steven A. Curley
   12. [Download PDF (90 KB)](http://www.sciencedirect.com/science/article/pii/S1365182X15313514/pdfft?md5=3338d76390dde4a4974dae0c9c9d60bb&pid=1-s2.0-S1365182X15313514-main.pdf)
6. [Benefits associated with antiviral treatment in kidney allograft recipients with chronic hepatitis B virus infection](http://www.sciencedirect.com/science/article/pii/S0168827812002073)
   1. Original research article
   2. Journal of Hepatology,
   3. Volume 57, Issue 1,
   4. July 2012,
   5. Pages 55-60
   6. Simona Cosconea,
   7. Hélène Fontaine,
   8. Jean-François Méritet,
   9. Marion Corouge,
   10. Stanislas Pol
   11. [Purchase PDF](http://www.sciencedirect.com/science/article/pii/S0168827812002073)
7. [Primary biliary cirrhosis and bile acids](http://www.sciencedirect.com/science/article/pii/S2210740112700165)
   1. Original research article
   2. Clinics and Research in Hepatology and Gastroenterology,
   3. Volume 36, Supplement 1,
   4. September 2012,
   5. Pages s13-s20
   6. Christophe Corpechot
   7. [Purchase PDF](http://www.sciencedirect.com/science/article/pii/S2210740112700165)
8. [Noninvasive diagnosis of nonalcoholic fatty liver disease: Are we there yet?](http://www.sciencedirect.com/science/article/pii/S0026049516000287)
   1. Original research article
   2. Metabolism,
   3. Volume 65, Issue 8,
   4. August 2016,
   5. Pages 1087-1095
   6. Naim Alkhouri,
   7. Ariel E. Feldstein
   8. [Purchase PDF](http://www.sciencedirect.com/science/article/pii/S0026049516000287)
9. [Prospective comparison of two algorithms combining non-invasive methods for staging liver fibrosis in chronic hepatitis C](http://www.sciencedirect.com/science/article/pii/S016882780900734X)
   1. Original research article
   2. Journal of Hepatology,
   3. Volume 52, Issue 2,
   4. February 2010,
   5. Pages 191-198
   6. Laurent Castéra,
   7. Giada Sebastiani,
   8. Brigitte Le Bail,
   9. Victor de Lédinghen,
   10. Alfredo Alberti
   11. [Purchase PDF](http://www.sciencedirect.com/science/article/pii/S016882780900734X)
10. [Pediatric Nonalcoholic Fatty Liver Disease: A Report from the Expert Committee on Nonalcoholic FattyLiver Disease (ECON)](http://www.sciencedirect.com/science/article/pii/S0022347615015267)
    1. Original research article
    2. The Journal of Pediatrics,
    3. Volume 172,
    4. May 2016,
    5. Pages 9-13
    6. Rohit Kohli,
    7. Shikha Sunduram,
    8. Marialena Mouzaki,
    9. Sabina Ali,
    10. Jeffrey B. Schwimmer
    11. [Purchase PDF](http://www.sciencedirect.com/science/article/pii/S0022347615015267)
11. [Slow regression of liver fibrosis presumed by repeated biomarkers after virological cure in patients with chronic hepatitis C](http://www.sciencedirect.com/science/article/pii/S0168827813003450)
    1. Original research article
    2. Journal of Hepatology,
    3. Volume 59, Issue 4,
    4. October 2013,
    5. Pages 675-683
    6. Thierry Poynard,
    7. Joseph Moussalli,
    8. Mona Munteanu,
    9. Dominique Thabut,
    10. FibroFrance-GHPS group
    11. [Purchase PDF](http://www.sciencedirect.com/science/article/pii/S0168827813003450)
12. [A 28-Year Study of the Course of Hepatitis Δ Infection: A Risk Factor for Cirrhosis and Hepatocellular Carcinoma](http://www.sciencedirect.com/science/article/pii/S0016508509001541)
    1. Original research article
    2. Gastroenterology,
    3. Volume 136, Issue 5,
    4. May 2009,
    5. Pages 1629-1638
    6. Raffaella Romeo,
    7. Ersilio Del Ninno,
    8. Mariagrazia Rumi,
    9. Antonio Russo,
    10. Massimo Colombo
    11. [Purchase PDF](http://www.sciencedirect.com/science/article/pii/S0016508509001541)
13. [norUrsodeoxycholic acid improves cholestasis in primary sclerosing cholangitis](http://www.sciencedirect.com/science/article/pii/S0168827817320457)
    1. Open access,
    2. Original research article
    3. Journal of Hepatology,
    4. Volume 67, Issue 3,
    5. September 2017,
    6. Pages 549-558
    7. Peter Fickert,
    8. Gideon M. Hirschfield,
    9. Gerald Denk,
    10. Hanns-Ulrich Marschall,
    11. European PSC norUDCA Study Group
    12. [Download PDF (1,068 KB)](http://www.sciencedirect.com/science/article/pii/S0168827817320457/pdfft?md5=f6957ade07fd48af39d689631da50588&pid=1-s2.0-S0168827817320457-main.pdf)
14. [The CRP level and STATE score predict survival in cirrhotic patients with hepatocellular carcinoma treated by transarterial embolization](http://www.sciencedirect.com/science/article/pii/S1590865816304649)
    1. Original research article
    2. Digestive and Liver Disease,
    3. Volume 48, Issue 9,
    4. September 2016,
    5. Pages 1088-1092
    6. Samia Rekik,
    7. Erwan Guyot,
    8. Mohannad Bhais,
    9. Yves Ajavon,
    10. Jean-Charles Nault
    11. [Purchase PDF](http://www.sciencedirect.com/science/article/pii/S1590865816304649)
15. [Similarities and differences between pediatric and adult nonalcoholic fatty liver disease](http://www.sciencedirect.com/science/article/pii/S0026049516000159)
    1. Original research article
    2. Metabolism,
    3. Volume 65, Issue 8,
    4. August 2016,
    5. Pages 1161-1171
    6. Maricruz Crespo,
    7. Sara Lappe,
    8. Ariel E. Feldstein,
    9. Naim Alkhouri
    10. [Purchase PDF](http://www.sciencedirect.com/science/article/pii/S0026049516000159)
16. [Spleen stiffness measurement can predict clinical complications in compensated HCV-related cirrhosis: A prospective study](http://www.sciencedirect.com/science/article/pii/S0168827814001329)
    1. Original research article
    2. Journal of Hepatology,
    3. Volume 60, Issue 6,
    4. June 2014,
    5. Pages 1158-1164
    6. Antonio Colecchia,
    7. Agostino Colli,
    8. Giovanni Casazza,
    9. Daniele Mandolesi,
    10. Davide Festi
    11. [Purchase PDF](http://www.sciencedirect.com/science/article/pii/S0168827814001329)
17. [Nonalcoholic fatty liver and the severity of acute pancreatitis](http://www.sciencedirect.com/science/article/pii/S0953620516303740)
    1. Original research article
    2. European Journal of Internal Medicine,
    3. Volume 38,
    4. March 2017,
    5. Pages 73-78
    6. I. Mikolasevic,
    7. L. Orlic,
    8. G. Poropat,
    9. I. Jakopcic,
    10. S. Milic
    11. [Purchase PDF](http://www.sciencedirect.com/science/article/pii/S0953620516303740)
18. [Non-invasive assessment of liver fibrosis with impulse elastography: Comparison of Supersonic Shear Imaging with ARFI and FibroScan®](http://www.sciencedirect.com/science/article/pii/S0168827814003079)
    1. Original research article
    2. Journal of Hepatology,
    3. Volume 61, Issue 3,
    4. September 2014,
    5. Pages 550-557
    6. Christophe Cassinotto,
    7. Bruno Lapuyade,
    8. Amaury Mouries,
    9. Jean-Baptiste Hiriart,
    10. Victor De Ledinghen
    11. [Purchase PDF](http://www.sciencedirect.com/science/article/pii/S0168827814003079)
19. [Role of magnetic resonance elastography in compensated and decompensated liver disease](http://www.sciencedirect.com/science/article/pii/S0168827813008842)
    1. Original research article
    2. Journal of Hepatology,
    3. Volume 60, Issue 5,
    4. May 2014,
    5. Pages 934-939
    6. Sumeet K. Asrani,
    7. Jayant A. Talwalkar,
    8. Patrick S. Kamath,
    9. Vijay H. Shah,
    10. Richard L. Ehman
    11. [Purchase PDF](http://www.sciencedirect.com/science/article/pii/S0168827813008842)
20. [Enfermedades hepáticas](http://www.sciencedirect.com/science/article/pii/S0210570508766378)
    1. Original research article
    2. Gastroenterología y Hepatología,
    3. Volume 31, Supplement 4,
    4. October 2008,
    5. Pages 93-104
    6. José Luis Calleja,
    7. Agustín Albillos,
    8. Rafael Bañares
    9. [Purchase PDF](http://www.sciencedirect.com/science/article/pii/S0210570508766378)
21. [Compartmental HBV evolution and replication in liver and extrahepatic sites after nucleos/tide analogue therapy in chronic hepatitis B carriers](http://www.sciencedirect.com/science/article/pii/S1386653217301853)
    1. Original research article
    2. Journal of Clinical Virology,
    3. Volume 94,
    4. September 2017,
    5. Pages 8-14
    6. Shan Gao,
    7. Zhong-Ping Duan,
    8. Yu Chen,
    9. Frank van der Meer,
    10. Carla S. Coffin
    11. [Purchase PDF](http://www.sciencedirect.com/science/article/pii/S1386653217301853)
22. [Safety and Effectiveness of Direct-Acting Antiviral Agents for Treatment of Patients With Chronic Hepatitis C Virus Infection and Cirrhosis](http://www.sciencedirect.com/science/article/pii/S1542356516303779)
    1. Original research article
    2. Clinical Gastroenterology and Hepatology,
    3. Volume 14, Issue 12,
    4. December 2016,
    5. Pages 1821-1830.e6
    6. Raoel Maan,
    7. Marjolein van Tilborg,
    8. Katja Deterding,
    9. Alnoor Ramji,
    10. Jordan J. Feld
    11. [Purchase PDF](http://www.sciencedirect.com/science/article/pii/S1542356516303779)
23. [Risk of cirrhosis-related complications in patients with advanced fibrosis following hepatitis C virus eradication](http://www.sciencedirect.com/science/article/pii/S0168827816305827)
    1. Original research article
    2. Journal of Hepatology,
    3. Volume 66, Issue 3,
    4. March 2017,
    5. Pages 485-493
    6. Adriaan J. van der Meer,
    7. Jordan J. Feld,
    8. Harald Hofer,
    9. Piero L. Almasio,
    10. Harry L.A. Janssen
    11. [Purchase PDF](http://www.sciencedirect.com/science/article/pii/S0168827816305827)
24. [Sofosbuvir plus daclatasvir with or without ribavirin for chronic hepatitis C infection: Impact of drug concentration on viral load decay](http://www.sciencedirect.com/science/article/pii/S1590865816305266)
    1. Original research article
    2. Digestive and Liver Disease,
    3. Volume 48, Issue 11,
    4. November 2016,
    5. Pages 1351-1356
    6. Victor Virlogeux,
    7. Laure Choupeaux,
    8. Pierre Pradat,
    9. Marianne Maynard,
    10. Fabien Zoulim
    11. [Purchase PDF](http://www.sciencedirect.com/science/article/pii/S1590865816305266)
25. [Optimising the clinical strategy for autoimmune liver diseases: Principles of value-based medicine](http://www.sciencedirect.com/science/article/pii/S0925443917303071)
    1. Original research article
    2. Biochimica et Biophysica Acta (BBA) - Molecular Basis of Disease,
    3. In press, accepted manuscript,
    4. Available online 24 August 2017
    5. Marco Carbone,
    6. Laura Cristoferi,
    7. Paolo Angelo Cortesi,
    8. Matteo Rota,
    9. Mario Strazzabosco
    10. [Purchase PDF](http://www.sciencedirect.com/science/article/pii/S0925443917303071)
26. [Tratamiento del paciente con hepatitis crónica por el virus de la hepatitis B](http://www.sciencedirect.com/science/article/pii/S0213005X08765200)
    1. Original research article
    2. Enfermedades Infecciosas y Microbiología Clínica,
    3. Volume 26, Supplement 7,
    4. May 2008,
    5. Pages 56-65
    6. Manuel Rodríguez,
    7. María Luisa González-Diéguez
    8. [Purchase PDF](http://www.sciencedirect.com/science/article/pii/S0213005X08765200)
27. [Effectiveness, safety and clinical outcomes of direct-acting antiviral therapy in HCV genotype 1 infection: Results from a Spanish real-world cohort](http://www.sciencedirect.com/science/article/pii/S0168827817300636)
    1. Original research article
    2. Journal of Hepatology,
    3. Volume 66, Issue 6,
    4. June 2017,
    5. Pages 1138-1148
    6. Jose Luis Calleja,
    7. Javier Crespo,
    8. Diego Rincón,
    9. Belén Ruiz-Antorán,
    10. The Spanish Group for the Study of the Use of Direct-acting Drugs Hepatitis C Collaborating Group
    11. [Purchase PDF](http://www.sciencedirect.com/science/article/pii/S0168827817300636)
28. [Effectiveness of Ledipasvir-Sofosbuvir Combination in Patients With Hepatitis C Virus Infection and Factors Associated With Sustained Virologic Response](http://www.sciencedirect.com/science/article/pii/S0016508516349277)
    1. Original research article
    2. Gastroenterology,
    3. Volume 151, Issue 6,
    4. December 2016,
    5. Pages 1131-1140.e5
    6. Norah A. Terrault,
    7. Stefan Zeuzem,
    8. Adrian M. Di Bisceglie,
    9. Joseph K. Lim,
    10. HCV-TARGET Study Group
    11. [Purchase PDF](http://www.sciencedirect.com/science/article/pii/S0016508516349277)
29. [Outbreak of hepatitis C virus infection during sclerotherapy of varicose veins: Long-term follow-up of 196 patients (4535 patient-years)](http://www.sciencedirect.com/science/article/pii/S0168827806004636)
    1. Original research article
    2. Journal of Hepatology,
    3. Volume 46, Issue 1,
    4. January 2007,
    5. Pages 19-25
    6. Victor de Lédinghen,
    7. Pascale Trimoulet,
    8. Paul-Régis Mannant,
    9. Francis Dumas,
    10. Pierre-Henri Bernard
    11. [Purchase PDF](http://www.sciencedirect.com/science/article/pii/S0168827806004636)
30. [Care of hepatitis C virus infection in human immunodeficiency virus-infected patients: Modifications in three consecutive large surveys between 2004 and 2009](http://www.sciencedirect.com/science/article/pii/S0168827810003260)
    1. Original research article
    2. Journal of Hepatology,
    3. Volume 53, Issue 2,
    4. August 2010,
    5. Pages 230-237
    6. Patrice Cacoub,
    7. Philippe Halfon,
    8. Eric Rosenthal,
    9. François Bailly,
    10. Stanislas Pol
    11. [Purchase PDF](http://www.sciencedirect.com/science/article/pii/S0168827810003260)
31. [Primary Biliary Cholangitis: advances in management and treatment of the disease](http://www.sciencedirect.com/science/article/pii/S1590865817308575)
    1. Open access,
    2. Original research article
    3. Digestive and Liver Disease,
    4. Volume 49, Issue 8,
    5. August 2017,
    6. Pages 841-846
    7. Pietro Invernizzi,
    8. Annarosa Floreani,
    9. Marco Carbone,
    10. Marco Marzioni,
    11. Domenico Alvaro
    12. [Download PDF (559 KB)](http://www.sciencedirect.com/science/article/pii/S1590865817308575/pdfft?md5=d8b851fe4ec6231fd602d957c94499d9&pid=1-s2.0-S1590865817308575-main.pdf)
32. [Virologic, Clinical, and Immune Response Outcomes of Patients With Hepatitis C Virus–Associated Cryoglobulinemia Treated With Direct-Acting Antivirals](http://www.sciencedirect.com/science/article/pii/S1542356516308783)
    1. Original research article
    2. Clinical Gastroenterology and Hepatology,
    3. Volume 15, Issue 4,
    4. April 2017,
    5. Pages 575-583.e1
    6. Martín Bonacci,
    7. Sabela Lens,
    8. María-Carlota Londoño,
    9. Zoe Mariño,
    10. José Hernández-Rodríguez
    11. [Purchase PDF](http://www.sciencedirect.com/science/article/pii/S1542356516308783)
33. [Sofosbuvir and ribavirin for genotype 2 HCV infected patients with cirrhosis: A real life experience](http://www.sciencedirect.com/science/article/pii/S0168827816307073)
    1. Original research article
    2. Journal of Hepatology,
    3. Volume 66, Issue 4,
    4. April 2017,
    5. Pages 711-717
    6. Alessandra Mangia,
    7. Simone Susser,
    8. Valeria Piazzolla,
    9. Ernesto Agostinacchio,
    10. Massimiliano Copetti
    11. [Purchase PDF](http://www.sciencedirect.com/science/article/pii/S0168827816307073)
34. [Ultrasound Elastography](http://www.sciencedirect.com/science/article/pii/S0976001611601117)
    1. Original research article
    2. Apollo Medicine,
    3. Volume 7, Issue 3,
    4. September 2010,
    5. Pages 224-226
    6. Meera Krishnakumar
    7. [Purchase PDF](http://www.sciencedirect.com/science/article/pii/S0976001611601117)
35. [The management of chronic hepatitis B in the immunocompromised patient: Recommendations from a single topic meeting](http://www.sciencedirect.com/science/article/pii/S1386653207004362)
    1. Original research article
    2. Journal of Clinical Virology,
    3. Volume 41, Issue 4,
    4. April 2008,
    5. Pages 243-254
    6. Stephen Barclay,
    7. Stanislas Pol,
    8. David Mutimer,
    9. Yves Benhamou,
    10. William Cameron
    11. [Purchase PDF](http://www.sciencedirect.com/science/article/pii/S1386653207004362)
36. [Validation of intimate correlation between visceral fat and hepatic steatosis: Quantitative measurement techniques using CT for area of fat and MR for hepatic steatosis](http://www.sciencedirect.com/science/article/pii/S0261561416313474)
    1. Original research article
    2. Clinical Nutrition,
    3. In press, corrected proof,
    4. Available online 18 December 2016
    5. Moon Hyung Choi,
    6. Joon-Il Choi,
    7. Michael Yong Park,
    8. Sung Eun Rha,
    9. Yohan Son
    10. [Purchase PDF](http://www.sciencedirect.com/science/article/pii/S0261561416313474)
37. [Marcadores serológicos de fibrosis](http://www.sciencedirect.com/science/article/pii/S0210570512700444)
    1. Original research article
    2. Gastroenterología y Hepatología,
    3. Volume 35, Supplement 2,
    4. December 2012,
    5. Pages 10-16
    6. Guillermo Fernández-Varo
    7. [Purchase PDF](http://www.sciencedirect.com/science/article/pii/S0210570512700444)
38. [Multiparametric magnetic resonance imaging predicts clinical outcomes in patients with chronic liverdisease](http://www.sciencedirect.com/science/article/pii/S0168827815006807)
    1. Open access,
    2. Original research article
    3. Journal of Hepatology,
    4. Volume 64, Issue 2,
    5. February 2016,
    6. Pages 308-315
    7. Michael Pavlides,
    8. Rajarshi Banerjee,
    9. Joanne Sellwood,
    10. Catherine J. Kelly,
    11. Eleanor Barnes
    12. [Download PDF (1,382 KB)](http://www.sciencedirect.com/science/article/pii/S0168827815006807/pdfft?md5=d3fc874466ad485ea0db88bce1795279&pid=1-s2.0-S0168827815006807-main.pdf)
39. [Detection of Risky Esophageal Varices by Two-Dimensional Ultrasound: When to Perform Endoscopy](http://www.sciencedirect.com/science/article/pii/S000296291530433X)
    1. Original research article
    2. The American Journal of the Medical Sciences,
    3. Volume 347, Issue 1,
    4. January 2014,
    5. Pages 28-33
    6. Abd El razek Mohammad Ali,
    7. Hamdy Mahfouz,
    8. Abd El wahhab Fathy,
    9. Khaled Abd El azeem,
    10. Magdy Mansour
    11. [Purchase PDF](http://www.sciencedirect.com/science/article/pii/S000296291530433X)
40. [Phase 1–2 pilot clinical trial in patients with decompensated liver cirrhosis treated with bone marrow–derived endothelial progenitor cells](http://www.sciencedirect.com/science/article/pii/S1931524416000633)
    1. Original research article
    2. Translational Research,
    3. In press, corrected proof,
    4. Available online 24 February 2016
    5. Delia D'Avola,
    6. Verónica Fernández-Ruiz,
    7. Francisco Carmona-Torre,
    8. Miriam Méndez,
    9. Jorge Quiroga
    10. [Purchase PDF](http://www.sciencedirect.com/science/article/pii/S1931524416000633)
41. [Development, functional characterization and validation of methodology for GMP-compliant manufacture of phagocytic macrophages: A novel cellular therapeutic for liver cirrhosis](http://www.sciencedirect.com/science/article/pii/S1465324917305923)
    1. Open access,
    2. Original research article
    3. Cytotherapy,
    4. Volume 19, Issue 9,
    5. September 2017,
    6. Pages 1113-1124
    7. Alasdair R. Fraser,
    8. Chloe Pass,
    9. Paul Burgoyne,
    10. Anne Atkinson,
    11. John D.M. Campbell
    12. [Download PDF (1,600 KB)](http://www.sciencedirect.com/science/article/pii/S1465324917305923/pdfft?md5=0adb9258eea5673b8739ef4f6f859b08&pid=1-s2.0-S1465324917305923-main.pdf)
42. [Portal Pressure Predicts Outcome and Safety of Antiviral Therapy in Cirrhotic Patients With Hepatitis C Virus Infection](http://www.sciencedirect.com/science/article/pii/S1542356511002308)
    1. Original research article
    2. Clinical Gastroenterology and Hepatology,
    3. Volume 9, Issue 7,
    4. July 2011,
    5. Pages 602-608.e1
    6. Thomas Reiberger,
    7. Karoline Rutter,
    8. Arnulf Ferlitsch,
    9. Berit Anna Payer,
    10. Markus Peck–Radosavljevic
    11. [Purchase PDF](http://www.sciencedirect.com/science/article/pii/S1542356511002308)
43. [High hepatic and extrahepatic mortality and low treatment uptake in HCV-coinfected persons in the Swiss HIV cohort study between 2001 and 2013](http://www.sciencedirect.com/science/article/pii/S0168827815003025)
    1. Original research article
    2. Journal of Hepatology,
    3. Volume 63, Issue 3,
    4. September 2015,
    5. Pages 573-580
    6. Helen Kovari,
    7. Bruno Ledergerber,
    8. Matthias Cavassini,
    9. Juan Ambrosioni,
    10. Swiss HIV Cohort Study
    11. [Purchase PDF](http://www.sciencedirect.com/science/article/pii/S0168827815003025)
44. [The stiffness of the liver and spleen on ARFI Imaging pre and post TIPS placement: a preliminary observation](http://www.sciencedirect.com/science/article/pii/S0899707111002579)
    1. Original research article
    2. Clinical Imaging,
    3. Volume 36, Issue 2,
    4. March–April 2012,
    5. Pages 135-141
    6. Jing Gao,
    7. Hai-Tao Ran,
    8. Xiao-Ping Ye,
    9. Yuan-Yi Zheng,
    10. Zhi-Gang Wang
    11. [Purchase PDF](http://www.sciencedirect.com/science/article/pii/S0899707111002579)
45. [Epidemiology, Screening, and Pretreatment Evaluation of the Patient With Chronic Hepatitis C Infection](http://www.sciencedirect.com/science/article/pii/S1555415514007417)
    1. Original research article
    2. The Journal for Nurse Practitioners,
    3. Volume 11, Issue 1,
    4. January 2015,
    5. Pages 109-115
    6. Donald Gardenier,
    7. Jeffrey Kwong,
    8. Mary C. Olson,
    9. Rachel Epstein
    10. [Purchase PDF](http://www.sciencedirect.com/science/article/pii/S1555415514007417)
46. [Determinants of Treatment Eligibility in Veterans With Hepatitis C Viral Infection](http://www.sciencedirect.com/science/article/pii/S0149291816308578)
    1. Original research article
    2. Clinical Therapeutics,
    3. Volume 39, Issue 1,
    4. January 2017,
    5. Pages 130-137
    6. Janice Taylor,
    7. Sian Carr-Lopez,
    8. Amy Robinson,
    9. Robert Malmstrom,
    10. Jannet M. Carmichael
    11. [Purchase PDF](http://www.sciencedirect.com/science/article/pii/S0149291816308578)
47. [Role of HCV-RNA decay and IP-10 levels after 48hours of standard HCV therapy as predictors of rapid virological response](http://www.sciencedirect.com/science/article/pii/S2210740115000844)
    1. Original research article
    2. Clinics and Research in Hepatology and Gastroenterology,
    3. Volume 39, Issue 6,
    4. December 2015,
    5. Pages 705-710
    6. Roberta Prinapori,
    7. Laura Sticchi,
    8. Cristiano Alicino,
    9. Filippo Del Puente,
    10. Antonio Di Biagio
    11. [Purchase PDF](http://www.sciencedirect.com/science/article/pii/S2210740115000844)
48. [AISF position paper on nonalcoholic fatty liver disease (NAFLD): Updates and future directions](http://www.sciencedirect.com/science/article/pii/S1590865817301512)
    1. Original research article
    2. Digestive and Liver Disease,
    3. Volume 49, Issue 5,
    4. May 2017,
    5. Pages 471-483
    6. The Italian Association for the Study of the Liver (AISF)
    7. [Purchase PDF](http://www.sciencedirect.com/science/article/pii/S1590865817301512)
49. [Comparison of the Phenotype and Approach to Pediatric vs Adult Patients With Nonalcoholic Fatty LiverDisease](http://www.sciencedirect.com/science/article/pii/S0016508516003553)
    1. Original research article
    2. Gastroenterology,
    3. Volume 150, Issue 8,
    4. June 2016,
    5. Pages 1798-1810
    6. Valerio Nobili,
    7. Anna Alisi,
    8. Kimberly P. Newton,
    9. Jeffrey B. Schwimmer
    10. [Purchase PDF](http://www.sciencedirect.com/science/article/pii/S0016508516003553)
50. [Automatic Assessment of Shear Wave Elastography Quality and Measurement Reliability in the Liver](http://www.sciencedirect.com/science/article/pii/S0301562914007352)
    1. Original research article
    2. Ultrasound in Medicine & Biology,
    3. Volume 41, Issue 4,
    4. April 2015,
    5. Pages 936-943
    6. Claire Pellot-Barakat,
    7. Muriel Lefort,
    8. Linda Chami,
    9. Mickaël Labit,
    10. Olivier Lucidarme
    11. [Purchase PDF](http://www.sciencedirect.com/science/article/pii/S0301562914007352)
51. [Reaching hepatitis C virus elimination targets requires health system interventions to enhance the care cascade](http://www.sciencedirect.com/science/article/pii/S0955395917302086)
    1. Original research article
    2. International Journal of Drug Policy,
    3. In press, corrected proof,
    4. Available online 7 August 2017
    5. Nick Scott,
    6. Joseph S. Doyle,
    7. David P. Wilson,
    8. Amanda Wade,
    9. Margaret E. Hellard
    10. [Purchase PDF](http://www.sciencedirect.com/science/article/pii/S0955395917302086)
52. [Ledipasvir-sofosbuvir with or without ribavirin to treat patients with HCV genotype 1 infection and cirrhosis non-responsive to previous protease-inhibitor therapy: a randomised, double-blind, phase 2 trial (SIRIUS)](http://www.sciencedirect.com/science/article/pii/S1473309915700502)
    1. Original research article
    2. The Lancet Infectious Diseases,
    3. Volume 15, Issue 4,
    4. April 2015,
    5. Pages 397-404
    6. Marc Bourlière,
    7. Jean-Pierre Bronowicki,
    8. Victor de Ledinghen,
    9. Christophe Hézode,
    10. Stanislas Pol
    11. [Purchase PDF](http://www.sciencedirect.com/science/article/pii/S1473309915700502)
53. [Diagnostic value of magnetic resonance elastography for detecting and staging of hepatic fibrosis: A meta-analysis](http://www.sciencedirect.com/science/article/pii/S0009926014004309)
    1. Original research article
    2. Clinical Radiology,
    3. Volume 69, Issue 12,
    4. December 2014,
    5. Pages e545-e552
    6. L.-N. Su,
    7. S.-L. Guo,
    8. B.-X. Li,
    9. P. Yang
    10. [Purchase PDF](http://www.sciencedirect.com/science/article/pii/S0009926014004309)
54. [Prospective evaluation of FibroTest®, FibroMeter®, and HepaScore® for staging liver fibrosis in chronic hepatitis B: Comparison with hepatitis C](http://www.sciencedirect.com/science/article/pii/S0168827814001378)
    1. Original research article
    2. Journal of Hepatology,
    3. Volume 61, Issue 1,
    4. July 2014,
    5. Pages 28-34
    6. Vincent Leroy,
    7. Nathalie Sturm,
    8. Patrice Faure,
    9. Candice Trocme,
    10. Jean-Pierre Zarski
    11. [Purchase PDF](http://www.sciencedirect.com/science/article/pii/S0168827814001378)
55. [The changing role of liver biopsy in diagnosis and management of haemochromatosis](http://www.sciencedirect.com/science/article/pii/S0031302516329749)
    1. Original research article
    2. Pathology,
    3. Volume 43, Issue 5,
    4. August 2011,
    5. Pages 433-439
    6. Mark L. Bassett,
    7. Peter E. Hickman,
    8. Jane E. Dahlstrom
    9. [Purchase PDF](http://www.sciencedirect.com/science/article/pii/S0031302516329749)
56. [Efficacy and safety study of cenicriviroc for the treatment of non-alcoholic steatohepatitis in adult subjects with liver fibrosis: CENTAUR Phase 2b study design](http://www.sciencedirect.com/science/article/pii/S155171441630026X)
    1. Open access,
    2. Original research article
    3. Contemporary Clinical Trials,
    4. Volume 47,
    5. March 2016,
    6. Pages 356-365
    7. Scott Friedman,
    8. Arun Sanyal,
    9. Zachary Goodman,
    10. Eric Lefebvre,
    11. Vlad Ratziu
    12. [Download PDF (864 KB)](http://www.sciencedirect.com/science/article/pii/S155171441630026X/pdfft?md5=3dea94ae0f1dc6ecc9db50385aa2b14b&pid=1-s2.0-S155171441630026X-main.pdf)
57. [Shear Wave Dispersion Measures Liver Steatosis](http://www.sciencedirect.com/science/article/pii/S0301562911014268)
    1. Original research article
    2. Ultrasound in Medicine & Biology,
    3. Volume 38, Issue 2,
    4. February 2012,
    5. Pages 175-182
    6. Christopher T. Barry,
    7. Bradley Mills,
    8. Zaegyoo Hah,
    9. Robert A. Mooney,
    10. Kevin J. Parker
    11. [Purchase PDF](http://www.sciencedirect.com/science/article/pii/S0301562911014268)
58. [Incidence of hepatocellular carcinoma after HBsAg seroclearance in chronic hepatitis B patients: A need for surveillance](http://www.sciencedirect.com/science/article/pii/S0168827814008800)
    1. Original research article
    2. Journal of Hepatology,
    3. Volume 62, Issue 5,
    4. May 2015,
    5. Pages 1092-1099
    6. Gi-Ae Kim,
    7. Han Chu Lee,
    8. Min-Ju Kim,
    9. Yeonjung Ha,
    10. Young-Suk Lim
    11. [Purchase PDF](http://www.sciencedirect.com/science/article/pii/S0168827814008800)
59. [Does motion affect liver stiffness estimates in shear wave elastography? Phantom and clinical study](http://www.sciencedirect.com/science/article/pii/S0720048X1630211X)
    1. Original research article
    2. European Journal of Radiology,
    3. Volume 85, Issue 9,
    4. September 2016,
    5. Pages 1645-1650
    6. Claire Pellot-Barakat,
    7. Linda Chami,
    8. Jean Michel Correas,
    9. Muriel Lefort,
    10. Olivier Lucidarme
    11. [Purchase PDF](http://www.sciencedirect.com/science/article/pii/S0720048X1630211X)
60. [Changes of HBsAg and interferon-inducible protein 10 serum levels in naive HBeAg-negative chronic hepatitis B patients under 4-year entecavir therapy](http://www.sciencedirect.com/science/article/pii/S0168827813006466)
    1. Original research article
    2. Journal of Hepatology,
    3. Volume 60, Issue 1,
    4. January 2014,
    5. Pages 62-68
    6. George Papatheodoridis,
    7. John Goulis,
    8. Spilios Manolakopoulos,
    9. Aikaterini Margariti,
    10. Evangelos Akriviadis
    11. [Purchase PDF](http://www.sciencedirect.com/science/article/pii/S0168827813006466)
61. [Use of the aspartate aminotransferase to platelet ratio index to follow liver fibrosis progression in infants with short gut](http://www.sciencedirect.com/science/article/pii/S0022346810002174)
    1. Original research article
    2. Journal of Pediatric Surgery,
    3. Volume 45, Issue 6,
    4. June 2010,
    5. Pages 1266-1273
    6. Richard S. Mangus,
    7. Michael G. O'Connor,
    8. A. Joseph Tector,
    9. Joel D. Lim,
    10. Rodrigo M. Vianna
    11. [Purchase PDF](http://www.sciencedirect.com/science/article/pii/S0022346810002174)
62. [Aspartate Aminotransferase-to-Platelet Ratio index (APRi) in infants with biliary atresia: Prognostic value at presentation](http://www.sciencedirect.com/science/article/pii/S0022346812007828)
    1. Original research article
    2. Journal of Pediatric Surgery,
    3. Volume 48, Issue 4,
    4. April 2013,
    5. Pages 789-795
    6. Andrew Grieve,
    7. Erica Makin,
    8. Mark Davenport
    9. [Purchase PDF](http://www.sciencedirect.com/science/article/pii/S0022346812007828)
63. [The utility of repeat liver biopsy in autoimmune hepatitis: a series of 20 consecutive cases](http://www.sciencedirect.com/science/article/pii/S0031302516400772)
    1. Original research article
    2. Pathology,
    3. Volume 48, Issue 5,
    4. August 2016,
    5. Pages 449-453
    6. Juan Putra,
    7. Arifa Toor,
    8. Arief A. Suriawinata
    9. [Purchase PDF](http://www.sciencedirect.com/science/article/pii/S0031302516400772)
64. [Noninvasive evaluation of hepatic fibrosis using acoustic radiation force-based shear stiffness in patients with nonalcoholic fatty liver disease](http://www.sciencedirect.com/science/article/pii/S0168827811000079)
    1. Original research article
    2. Journal of Hepatology,
    3. Volume 55, Issue 3,
    4. September 2011,
    5. Pages 666-672
    6. Mark L. Palmeri,
    7. Michael H. Wang,
    8. Ned C. Rouze,
    9. Manal F. Abdelmalek,
    10. Kathryn R. Nightingale
    11. [Purchase PDF](http://www.sciencedirect.com/science/article/pii/S0168827811000079)
65. [The ALBI grade provides objective hepatic reserve estimation across each BCLC stage of hepatocellular carcinoma](http://www.sciencedirect.com/science/article/pii/S0168827816305359)
    1. Original research article
    2. Journal of Hepatology,
    3. Volume 66, Issue 2,
    4. February 2017,
    5. Pages 338-346
    6. David J. Pinato,
    7. Rohini Sharma,
    8. Elias Allara,
    9. Clarence Yen,
    10. Brian I. Carr
    11. [Purchase PDF](http://www.sciencedirect.com/science/article/pii/S0168827816305359)
66. [Collagen proportionate area is superior to other histological methods for sub-classifying cirrhosis and determining prognosis](http://www.sciencedirect.com/science/article/pii/S0168827814000038)
    1. Open access,
    2. Original research article
    3. Journal of Hepatology,
    4. Volume 60, Issue 5,
    5. May 2014,
    6. Pages 948-954
    7. Emmanuel Tsochatzis,
    8. Sara Bruno,
    9. Graziella Isgro,
    10. Andrew Hall,
    11. Tu Vinh Luong
    12. [Download PDF (1,688 KB)](http://www.sciencedirect.com/science/article/pii/S0168827814000038/pdfft?md5=ab9c1ec586a087c21434a217920ca34b&pid=1-s2.0-S0168827814000038-main.pdf)
67. [Testosterone therapy increases muscle mass in men with cirrhosis and low testosterone: A randomised controlled trial](http://www.sciencedirect.com/science/article/pii/S0168827816302690)
    1. Original research article
    2. Journal of Hepatology,
    3. Volume 65, Issue 5,
    4. November 2016,
    5. Pages 906-913
    6. Marie Sinclair,
    7. Mathis Grossmann,
    8. Rudolf Hoermann,
    9. Peter W. Angus,
    10. Paul J. Gow
    11. [Purchase PDF](http://www.sciencedirect.com/science/article/pii/S0168827816302690)
68. [The Laennec staging system for histological sub-classification of cirrhosis is useful for stratification of prognosis in patients with liver cirrhosis](http://www.sciencedirect.com/science/article/pii/S0168827812003510)
    1. Original research article
    2. Journal of Hepatology,
    3. Volume 57, Issue 3,
    4. September 2012,
    5. Pages 556-563
    6. Seung Up Kim,
    7. Hyun Jung Oh,
    8. Ian R. Wanless,
    9. Sarah Lee,
    10. Young Nyun Park
    11. [Purchase PDF](http://www.sciencedirect.com/science/article/pii/S0168827812003510)
69. [Introduction](http://www.sciencedirect.com/science/article/pii/S1594580409600073)
    1. Original research article
    2. Digestive and Liver Disease Supplements,
    3. Volume 3, Issue 1,
    4. April 2009,
    5. Pages 1-2
    6. Piero L. Almasio,
    7. Gamal Esmat
    8. [Purchase PDF](http://www.sciencedirect.com/science/article/pii/S1594580409600073)
70. [Online combination algorithm for non-invasive assessment of chronic hepatitis B related liver fibrosis and cirrhosis in resource-limited settings](http://www.sciencedirect.com/science/article/pii/S0953620515002319)
    1. Original research article
    2. European Journal of Internal Medicine,
    3. Volume 26, Issue 8,
    4. October 2015,
    5. Pages 628-634
    6. Nermin N. Salkic,
    7. Elmir Cickusic,
    8. Predrag Jovanovic,
    9. Mirela Basic Denjagic,
    10. Sead Ahmetagic
    11. [Purchase PDF](http://www.sciencedirect.com/science/article/pii/S0953620515002319)
71. [Noninvasive Methods to Assess Liver Disease in Patients With Hepatitis B or C](http://www.sciencedirect.com/science/article/pii/S0016508512002302)
    1. Original research article
    2. Gastroenterology,
    3. Volume 142, Issue 6,
    4. May 2012,
    5. Pages 1293-1302.e4
    6. Laurent Castera
    7. [Purchase PDF](http://www.sciencedirect.com/science/article/pii/S0016508512002302)
72. [Real-time elastography as a noninvasive technique for quantification of fibrosis in patients with chronic viral liver disease: Preliminary findings](http://www.sciencedirect.com/science/article/pii/S1971349512000641)
    1. Original research article
    2. Journal of Ultrasound,
    3. Volume 15, Issue 4,
    4. December 2012,
    5. Pages 220-225
    6. E. Fiorini,
    7. V. Cipriano,
    8. C. De Molo,
    9. S. Righi,
    10. C. Serra
    11. [Purchase PDF](http://www.sciencedirect.com/science/article/pii/S1971349512000641)
73. [Nonalcoholic fatty liver disease is associated with an increased prevalence of distal symmetric polyneuropathy in adult patients with type 1 diabetes](http://www.sciencedirect.com/science/article/pii/S105687271630544X)
    1. Original research article
    2. Journal of Diabetes and its Complications,
    3. Volume 31, Issue 6,
    4. June 2017,
    5. Pages 1021-1026
    6. Alessandro Mantovani,
    7. Riccardo Rigolon,
    8. Lucia Mingolla,
    9. Isabella Pichiri,
    10. Giovanni Targher
    11. [Purchase PDF](http://www.sciencedirect.com/science/article/pii/S105687271630544X)
74. [Hepatic fibrosis in paediatric liver disease](http://www.sciencedirect.com/science/article/pii/S2210740112000952)
    1. Original research article
    2. Clinics and Research in Hepatology and Gastroenterology,
    3. Volume 36, Issue 3,
    4. June 2012,
    5. Pages 268-270
    6. Imeke Goldschmidt,
    7. Ulrich Baumann
    8. [Purchase PDF](http://www.sciencedirect.com/science/article/pii/S2210740112000952)
75. [Elastographic assessment of liver fibrosis in children: A prospective single center experience](http://www.sciencedirect.com/science/article/pii/S0720048X12001878)
    1. Original research article
    2. European Journal of Radiology,
    3. Volume 81, Issue 8,
    4. August 2012,
    5. Pages e870-e874
    6. Cristina Oana Marginean,
    7. Claudiu Marginean
    8. [Purchase PDF](http://www.sciencedirect.com/science/article/pii/S0720048X12001878)
76. [Indications of Liver Biopsy in the Era of Noninvasive Assessment of Liver Fibrosis](http://www.sciencedirect.com/science/article/pii/S097368831500420X)
    1. Original research article
    2. Journal of Clinical and Experimental Hepatology,
    3. Volume 5, Issue 4,
    4. December 2015,
    5. Pages 314-319
    6. Deepak Amarapurkar,
    7. Anjali Amarapurkar
    8. [Purchase PDF](http://www.sciencedirect.com/science/article/pii/S097368831500420X)
77. [A Machine-Learning Algorithm Toward Color Analysis for Chronic Liver Disease Classification, Employing Ultrasound Shear Wave Elastography](http://www.sciencedirect.com/science/article/pii/S0301562917301977)
    1. Original research article
    2. Ultrasound in Medicine & Biology,
    3. Volume 43, Issue 9,
    4. September 2017,
    5. Pages 1797-1810
    6. Ilias Gatos,
    7. Stavros Tsantis,
    8. Stavros Spiliopoulos,
    9. Dimitris Karnabatidis,
    10. George C. Kagadis
    11. [Purchase PDF](http://www.sciencedirect.com/science/article/pii/S0301562917301977)
78. [HCV mono-infected and HIV/HCV co-infected individuals treated with direct-acting antivirals: to what extent do they differ?](http://www.sciencedirect.com/science/article/pii/S1201971217301790)
    1. Open access,
    2. Original research article
    3. International Journal of Infectious Diseases,
    4. Volume 62,
    5. September 2017,
    6. Pages 64-71
    7. Giuseppe Bruno,
    8. Annalisa Saracino,
    9. Luigia Scudeller,
    10. Claudia Fabrizio,
    11. Gioacchino Angarano
    12. [Download PDF (632 KB)](http://www.sciencedirect.com/science/article/pii/S1201971217301790/pdfft?md5=1abc0df8b5b8e29a8e88dd3a3aea5e11&pid=1-s2.0-S1201971217301790-main.pdf)
79. [Natural History and Clinical Impact of Cryoglobulins in Chronic Hepatitis C: 10-Year Prospective Study of 343 Patients](http://www.sciencedirect.com/science/article/pii/S0016508507012966)
    1. Original research article
    2. Gastroenterology,
    3. Volume 133, Issue 3,
    4. September 2007,
    5. Pages 835-842
    6. Mauro Viganò,
    7. Pietro Lampertico,
    8. Maria Grazia Rumi,
    9. Christian Folli,
    10. Massimo Colombo
    11. [Purchase PDF](http://www.sciencedirect.com/science/article/pii/S0016508507012966)
80. [The clinical significance of HCV core antigen detection during Telaprevir/Peg-Interferon/Ribavirin therapy in patients with HCV 1 genotype infection](http://www.sciencedirect.com/science/article/pii/S1386653215001869)
    1. Original research article
    2. Journal of Clinical Virology,
    3. Volume 69,
    4. August 2015,
    5. Pages 68-73
    6. Anna Rosa Garbuglia,
    7. Raffaella Lionetti,
    8. Daniele Lapa,
    9. Chiara Taibi,
    10. Paola Paci
    11. [Purchase PDF](http://www.sciencedirect.com/science/article/pii/S1386653215001869)
81. [The addition of a protease inhibitor increases the risk of infections in patients with hepatitis C-related cirrhosis](http://www.sciencedirect.com/science/article/pii/S0168827814007168)
    1. Original research article
    2. Journal of Hepatology,
    3. Volume 62, Issue 2,
    4. February 2015,
    5. Pages 311-316
    6. Maria-Carlota Londoño,
    7. Christie Perelló,
    8. Joaquín Cabezas,
    9. Nuria Cañete,
    10. Xavier Forns
    11. [Purchase PDF](http://www.sciencedirect.com/science/article/pii/S0168827814007168)
82. [Histological parameters and alcohol abstinence determine long-term prognosis in patients with alcoholicliver disease](http://www.sciencedirect.com/science/article/pii/S0168827816306857)
    1. Original research article
    2. Journal of Hepatology,
    3. Volume 66, Issue 3,
    4. March 2017,
    5. Pages 610-618
    6. Carolin Lackner,
    7. Walter Spindelboeck,
    8. Johannes Haybaeck,
    9. Philipp Douschan,
    10. Rudolf E. Stauber
    11. [Purchase PDF](http://www.sciencedirect.com/science/article/pii/S0168827816306857)
83. [Hepatitis B virus: From diagnosis to treatment](http://www.sciencedirect.com/science/article/pii/S0369811410000635)
    1. Original research article
    2. Pathologie Biologie,
    3. Volume 58, Issue 4,
    4. August 2010,
    5. Pages 245-253
    6. P. Dény,
    7. F. Zoulim
    8. [Purchase PDF](http://www.sciencedirect.com/science/article/pii/S0369811410000635)
84. [Diagnostic Performance of Magnetic Resonance Elastography in Staging Liver Fibrosis: A Systematic Review and Meta-analysis of Individual Participant Data](http://www.sciencedirect.com/science/article/pii/S1542356514013950)
    1. Original research article
    2. Clinical Gastroenterology and Hepatology,
    3. Volume 13, Issue 3,
    4. March 2015,
    5. Pages 440-451.e6
    6. Siddharth Singh,
    7. Sudhakar K. Venkatesh,
    8. Zhen Wang,
    9. Frank H. Miller,
    10. Richard L. Ehman
    11. [Purchase PDF](http://www.sciencedirect.com/science/article/pii/S1542356514013950)
85. [Acoustic Radiation Force Impulse Elastography of the Liver in Healthy Patients: Test Location, Reference Range and Influence of Gender and Body Mass Index](http://www.sciencedirect.com/science/article/pii/S0301562914006656)
    1. Original research article
    2. Ultrasound in Medicine & Biology,
    3. Volume 41, Issue 3,
    4. March 2015,
    5. Pages 698-704
    6. Li-Ying Liao,
    7. Kuan-Liang Kuo,
    8. Huei-Shin Chiang,
    9. Chong-Zong Lin,
    10. Chih-Lin Lin
    11. [Purchase PDF](http://www.sciencedirect.com/science/article/pii/S0301562914006656)
86. [Clinical and Biochemical Profile of Tuberculosis in Patients with Liver Cirrhosis](http://www.sciencedirect.com/science/article/pii/S0973688315000092)
    1. Original research article
    2. Journal of Clinical and Experimental Hepatology,
    3. Volume 5, Issue 1,
    4. March 2015,
    5. Pages 8-13
    6. Praveen Sharma,
    7. Pankaj Tyagi,
    8. Vikas Singla,
    9. Naresh Bansal,
    10. Anil Arora
    11. [Purchase PDF](http://www.sciencedirect.com/science/article/pii/S0973688315000092)
87. [Virologic response at week 8 of combined treatment as a predictor of sustained virologic response in non rapid virologic response, chronic HCV genotype 4 infected patients](http://www.sciencedirect.com/science/article/pii/S1110863012000237)
    1. Open access,
    2. Original research article
    3. Egyptian Journal of Medical Human Genetics,
    4. Volume 13, Issue 3,
    5. October 2012,
    6. Pages 331-335
    7. Ali Monis,
    8. Ahmed Ali Monis,
    9. Reham Al Swaff
    10. [Download PDF (289 KB)](http://www.sciencedirect.com/science/article/pii/S1110863012000237/pdfft?md5=37d80c0708de4816b81ba84c1009c470&pid=1-s2.0-S1110863012000237-main.pdf)
88. [Pilot study on the use of acoustic radiation force impulse imaging in the staging of cystic fibrosis associatedliver disease](http://www.sciencedirect.com/science/article/pii/S1569199312000513)
    1. Open archive,
    2. Original research article
    3. Journal of Cystic Fibrosis,
    4. Volume 11, Issue 5,
    5. September 2012,
    6. Pages 427-432
    7. Melania Manco,
    8. Cristina Lo Zupone,
    9. Federico Alghisi,
    10. Maria Luisa D'Andrea,
    11. Lidia Monti
    12. [Download PDF (165 KB)](http://www.sciencedirect.com/science/article/pii/S1569199312000513/pdfft?md5=de90df636bfd8f0429223f8519a72321&pid=1-s2.0-S1569199312000513-main.pdf)
89. [Sarcopenia is an independent risk factor for non-alcoholic steatohepatitis and significant fibrosis](http://www.sciencedirect.com/science/article/pii/S0168827816304846)
    1. Original research article
    2. Journal of Hepatology,
    3. Volume 66, Issue 1,
    4. January 2017,
    5. Pages 123-131
    6. Bo Kyung Koo,
    7. Donghee Kim,
    8. Sae Kyung Joo,
    9. Jung Ho Kim,
    10. Won Kim
    11. [Purchase PDF](http://www.sciencedirect.com/science/article/pii/S0168827816304846)
90. [Exosomes mediate intercellular transfer of pro-fibrogenic connective tissue growth factor (CCN2) between hepatic stellate cells, the principal fibrotic cells in the liver](http://www.sciencedirect.com/science/article/pii/S0039606014001846)
    1. Original research article
    2. Surgery,
    3. Volume 156, Issue 3,
    4. September 2014,
    5. Pages 548-555
    6. Alyssa Charrier,
    7. Ruju Chen,
    8. Li Chen,
    9. Sherri Kemper,
    10. David R. Brigstock
    11. [Purchase PDF](http://www.sciencedirect.com/science/article/pii/S0039606014001846)
91. [Gastrointestinal Complications of Cystic Fibrosis](http://www.sciencedirect.com/science/article/pii/S1542356512013080)
    1. Original research article
    2. Clinical Gastroenterology and Hepatology,
    3. Volume 11, Issue 4,
    4. April 2013,
    5. Pages 333-342
    6. Daniel Gelfond,
    7. Drucy Borowitz
    8. [Purchase PDF](http://www.sciencedirect.com/science/article/pii/S1542356512013080)
92. [Prevalence and characterization of fibrosis in surveillance liver biopsies of patients with Fontan circulation](http://www.sciencedirect.com/science/article/pii/S0046817716301575)
    1. Original research article
    2. Human Pathology,
    3. Volume 57,
    4. November 2016,
    5. Pages 106-115
    6. Lea F. Surrey,
    7. Pierre Russo,
    8. Jack Rychik,
    9. David J. Goldberg,
    10. Henry C. Lin
    11. [Purchase PDF](http://www.sciencedirect.com/science/article/pii/S0046817716301575)
93. [Changes in liver stiffness using acoustic radiation force impulse imaging in patients with obstructive cholestasis and cholangitis](http://www.sciencedirect.com/science/article/pii/S1590865814002734)
    1. Original research article
    2. Digestive and Liver Disease,
    3. Volume 46, Issue 7,
    4. July 2014,
    5. Pages 625-631
    6. Dina Attia,
    7. Sven Pischke,
    8. Ahmad A. Negm,
    9. Kinan Rifai,
    10. Andrej Potthoff
    11. [Purchase PDF](http://www.sciencedirect.com/science/article/pii/S1590865814002734)
94. [Identification of naïve HVC-4 patients who may be treated with pegylated-interferon and ribavirin according to IL28B polymorphisms](http://www.sciencedirect.com/science/article/pii/S0166354214000904)
    1. Original research article
    2. Antiviral Research,
    3. Volume 106,
    4. June 2014,
    5. Pages 105-110
    6. Lucio Boglione,
    7. Jessica Cusato,
    8. Amedeo De Nicolò,
    9. Giuseppe Cariti,
    10. Antonio D’Avolio
    11. [Purchase PDF](http://www.sciencedirect.com/science/article/pii/S0166354214000904)
95. [Efficacy of the Combination of Sofosbuvir, Velpatasvir, and the NS3/4A Protease Inhibitor GS-9857 in Treatment-Naïve or Previously Treated Patients With Hepatitis C Virus Genotype 1 or 3 Infections](http://www.sciencedirect.com/science/article/pii/S0016508516345139)
    1. Original research article
    2. Gastroenterology,
    3. Volume 151, Issue 3,
    4. September 2016,
    5. Pages 448-456.e1
    6. Edward J. Gane,
    7. Christian Schwabe,
    8. Robert H. Hyland,
    9. Yin Yang,
    10. Catherine A. Stedman
    11. [Purchase PDF](http://www.sciencedirect.com/science/article/pii/S0016508516345139)
96. [Hepatocellular Carcinoma in Chronic Hepatitis B Patients on Third Generation Nucleos(t)ides Analogs: Risk Factors and Performance of a Risk Score](http://www.sciencedirect.com/science/article/pii/S2341454516000302)
    1. Open access,
    2. Original research article
    3. GE Portuguese Journal of Gastroenterology,
    4. Volume 23, Issue 5,
    5. September–October 2016,
    6. Pages 233-242
    7. Pedro Magalhães-Costa,
    8. Luís Lebre,
    9. Paula Peixe,
    10. Sofia Santos,
    11. Cristina Chagas
    12. [Download PDF (958 KB)](http://www.sciencedirect.com/science/article/pii/S2341454516000302/pdfft?md5=f6a1cf02214bdfe0325546f4ab85f0cb&pid=1-s2.0-S2341454516000302-main.pdf)
97. [Predicting the severity of liver cirrhosis through clinical parameters](http://www.sciencedirect.com/science/article/pii/S0022480416300397)
    1. Original research article
    2. Journal of Surgical Research,
    3. Volume 204, Issue 2,
    4. August 2016,
    5. Pages 274-281
    6. Er-lei Zhang,
    7. Zun-yi Zhang,
    8. Shu-ping Wang,
    9. Zhen-yu Xiao,
    10. Zhi-yong Huang
    11. [Purchase PDF](http://www.sciencedirect.com/science/article/pii/S0022480416300397)
98. [Pharmacological management of nonalcoholic fatty liver disease](http://www.sciencedirect.com/science/article/pii/S0026049516300087)
    1. Open access,
    2. Original research article
    3. Metabolism,
    4. Volume 65, Issue 8,
    5. August 2016,
    6. Pages 1183-1195
    7. Diana Barb,
    8. Paola Portillo-Sanchez,
    9. Kenneth Cusi
    10. [Download PDF (567 KB)](http://www.sciencedirect.com/science/article/pii/S0026049516300087/pdfft?md5=f0ab35dff143aab12624d1b011ff6ca2&pid=1-s2.0-S0026049516300087-main.pdf)
99. [25](http://www.sciencedirect.com/search?qs=%22Liver%20transplantation%22%20and%20%22transient%20elastography%22&show=25&sortBy=relevance&articleTypes=OR&navigation=true)
100. [50](http://www.sciencedirect.com/search?qs=%22Liver%20transplantation%22%20and%20%22transient%20elastography%22&show=50&sortBy=relevance&articleTypes=OR&navigation=true)
101. 100
102. [previous](http://www.sciencedirect.com/search?qs=%22Liver%20transplantation%22%20and%20%22transient%20elastography%22&show=100&sortBy=relevance&articleTypes=OR&offset=0&navigation=true)
103. Page 2 of 3
104. [next](http://www.sciencedirect.com/search?qs=%22Liver%20transplantation%22%20and%20%22transient%20elastography%22&show=100&sortBy=relevance&articleTypes=OR&offset=200&navigation=true)

[Feedback](http://www.sciencedirect.com/search?qs=%22Liver%20transplantation%22%20and%20%22transient%20elastography%22&show=100&sortBy=relevance&articleTypes=OR&offset=100)

[About ScienceDirect](http://www.elsevier.com/solutions/sciencedirect)[Remote access](http://www.sciencedirect.com/science/activateaccess)[Shopping cart](http://www.sciencedirect.com/science?_ob=ShoppingCartURL&_method=display&_zone=TopNavBar&_origin=srp&md5=e5857d1631c4b1f612897ed029064507)[Contact and support](https://service.elsevier.com/app/contact/supporthub/sciencedirect/)[Terms and conditions](https://www.elsevier.com/legal/elsevier-website-terms-and-conditions)[Privacy policy](http://www.elsevier.com/legal/privacy-policy)

Cookies are used by this site. For more information, visit the [cookies page](https://www.elsevier.com/solutions/sciencedirect/support/cookies).

Copyright © 2017 Elsevier B.V. or its licensors or contributors. ScienceDirect ® is a registered trademark of Elsevier B.V.

[Skip to Main content](http://www.sciencedirect.com/search?qs=%22Liver%20transplantation%22%20and%20%22transient%20elastography%22&show=100&sortBy=relevance&articleTypes=OR&offset=200&navigation=true#main_content)

- [Journals](http://www.sciencedirect.com/science/journals)
- [Books](http://www.sciencedirect.com/science/bookbshsrw)
- [Register](https://www.sciencedirect.com/user/register?returnURL=http%3A%2F%2Fwww.sciencedirect.com%2Fsearch)
- [Sign in](https://www.sciencedirect.com/user/login?returnURL=http%3A%2F%2Fwww.sciencedirect.com%2Fsearch)

Top of Form

[Advanced search](http://www.sciencedirect.com/science/search)

Bottom of Form

252 results

Refine by:

Top of Form

Years

1. 2017 (35)
2. 2016 (48)
3. 2015 (36)
4. 2014 (23)
5. 2013 (15)
6. 2012 (28)
7. 2011 (18)
8. 2010 (14)
9. 2009 (13)
10. 2008 (15)

Show less

Article type

1. Original research (252)

Publication title

1. Journal of Hepatology (53)
2. Clinical Gastroenterology and Hepatology (24)
3. Gastroenterology (22)
4. Digestive and Liver Disease (19)
5. Transplantation Proceedings (9)
6. Ultrasound in Medicine & Biology (8)
7. European Journal of Radiology (6)
8. Metabolism (6)
9. Best Practice & Research Clinical Gastroenterology (6)
10. Gastroenterología y Hepatología (5)

Show less

[Clear all filters](http://www.sciencedirect.com/search?qs=%22Liver%20transplantation%22%20and%20%22transient%20elastography%22&show=100&sortBy=relevance&offset=200&navigation=true)

Bottom of Form

sorted by *relevance* | [date](http://www.sciencedirect.com/search?qs=%22Liver%20transplantation%22%20and%20%22transient%20elastography%22&show=100&sortBy=date&articleTypes=OR&offset=200&navigation=true)

1. [Correlation of FIBROSpect II With Histologic and Morphometric Evaluation of Liver Fibrosis in Chronic Hepatitis C](http://www.sciencedirect.com/science/article/pii/S1542356507011056)
   1. Original research article
   2. Clinical Gastroenterology and Hepatology,
   3. Volume 6, Issue 2,
   4. February 2008,
   5. Pages 242-247
   6. Keyur Patel,
   7. David R. Nelson,
   8. Don C. Rockey,
   9. Nezam H. Afdhal,
   10. John G. McHutchison
   11. [Purchase PDF](http://www.sciencedirect.com/science/article/pii/S1542356507011056)
2. [Serum Biomarkers Indicate Long-term Reduction in Liver Fibrosis in Patients With Sustained Virological Response to Treatment for HCV Infection](http://www.sciencedirect.com/science/article/pii/S1542356516000525)
   1. Original research article
   2. Clinical Gastroenterology and Hepatology,
   3. Volume 14, Issue 7,
   4. July 2016,
   5. Pages 1044-1055.e3
   6. Mei Lu,
   7. Jia Li,
   8. Talan Zhang,
   9. Loralee B. Rupp,
   10. Chronic Hepatitis Cohort Study Investigators
   11. [Purchase PDF](http://www.sciencedirect.com/science/article/pii/S1542356516000525)
3. Want a richer search experience?

Sign in for additional filter options, multiple article downloads, and more.

Top of Form

Sign in

Bottom of Form

1. [Hyaluronic acid predicts hepatic fibrosis in children with nonalcoholic fatty liver disease](http://www.sciencedirect.com/science/article/pii/S1931524410001015)
   1. Original research article
   2. Translational Research,
   3. Volume 156, Issue 4,
   4. October 2010,
   5. Pages 229-234
   6. Valerio Nobili,
   7. Anna Alisi,
   8. Giuliano Torre,
   9. Rita De Vito,
   10. Massimo Pinzani
   11. [Purchase PDF](http://www.sciencedirect.com/science/article/pii/S1931524410001015)
2. [Triple therapy with ursodeoxycholic acid, budesonide and mycophenolate mofetil in patients with features of severe primary biliary cirrhosis not responding to ursodeoxycholic acid alone](http://www.sciencedirect.com/science/article/pii/S0399832010000709)
   1. Original research article
   2. Gastroentérologie Clinique et Biologique,
   3. Volume 34, Issues 4–5,
   4. April–May 2010,
   5. Pages 283-287
   6. N. Rabahi,
   7. Y. Chrétien,
   8. F. Gaouar,
   9. D. Wendum,
   10. R. Poupon
   11. [Purchase PDF](http://www.sciencedirect.com/science/article/pii/S0399832010000709)
3. [A Randomized Trial of Silymarin for the Treatment of Nonalcoholic Steatohepatitis](http://www.sciencedirect.com/science/article/pii/S1542356517304597)
   1. Original research article
   2. Clinical Gastroenterology and Hepatology,
   3. In press, corrected proof,
   4. Available online 15 April 2017
   5. Wah-Kheong Chan,
   6. Nik Raihan Nik Mustapha,
   7. Sanjiv Mahadeva
   8. [Purchase PDF](http://www.sciencedirect.com/science/article/pii/S1542356517304597)
4. [Multiple approaches to assess fourteen non-invasive serum indexes for the diagnosis of liver fibrosis in chronic hepatitis C patients](http://www.sciencedirect.com/science/article/pii/S0009912016000874)
   1. Original research article
   2. Clinical Biochemistry,
   3. Volume 49, Issues 7–8,
   4. May 2016,
   5. Pages 560-565
   6. María Jesús Andrés-Otero,
   7. Ignacio De-Blas-Giral,
   8. Juan José Puente-Lanzarote,
   9. Trinidad Serrano-Aulló,
   10. José Manuel Lou-Bonafonte
   11. [Purchase PDF](http://www.sciencedirect.com/science/article/pii/S0009912016000874)
5. [Management of Patients Coinfected With HCV and HIV: A Close Look at the Role for Direct-Acting Antivirals](http://www.sciencedirect.com/science/article/pii/S0016508512002247)
   1. Original research article
   2. Gastroenterology,
   3. Volume 142, Issue 6,
   4. May 2012,
   5. Pages 1324-1334.e3
   6. Susanna Naggie,
   7. Mark S. Sulkowski
   8. [Purchase PDF](http://www.sciencedirect.com/science/article/pii/S0016508512002247)
6. [Nonalcoholic fatty liver disease and bariatric surgery in adolescents](http://www.sciencedirect.com/science/article/pii/S1055858613000681)
   1. Original research article
   2. Seminars in Pediatric Surgery,
   3. Volume 23, Issue 1,
   4. February 2014,
   5. Pages 49-57
   6. AiXuan Holterman,
   7. Juan Gurria,
   8. Smita Tanpure,
   9. Nerina DiSomma
   10. [Purchase PDF](http://www.sciencedirect.com/science/article/pii/S1055858613000681)
7. [Early changes in dynamic biomarkers of liver fibrosis in hepatitis C virus-infected patients treated with sofosbuvir](http://www.sciencedirect.com/science/article/pii/S159086581500626X)
   1. Open access,
   2. Original research article
   3. Digestive and Liver Disease,
   4. Volume 48, Issue 3,
   5. March 2016,
   6. Pages 291-297
   7. Sebastian Bernuth,
   8. Eray Yagmur,
   9. Detlef Schuppan,
   10. Martin F. Sprinzl,
   11. Tim Zimmermann
   12. [Download PDF (794 KB)](http://www.sciencedirect.com/science/article/pii/S159086581500626X/pdfft?md5=1137242a7942ae56c781a9b9129f2cd9&pid=1-s2.0-S159086581500626X-main.pdf)
8. [Hepatitis C Virus: A Critical Appraisal of Approaches to Therapy](http://www.sciencedirect.com/science/article/pii/S1542356508011609)
   1. Original research article
   2. Clinical Gastroenterology and Hepatology,
   3. Volume 7, Issue 4,
   4. April 2009,
   5. Pages 397-414
   6. David R. Nelson,
   7. Gary L. Davis,
   8. Ira Jacobson,
   9. Gregory T. Everson,
   10. Nizar Zein
   11. [Purchase PDF](http://www.sciencedirect.com/science/article/pii/S1542356508011609)
9. [Sustained virologic response of 100% in HCV genotype 1b patients with cirrhosis receiving ombitasvir/paritaprevir/r and dasabuvir for 12weeks](http://www.sciencedirect.com/science/article/pii/S0168827815006765)
   1. Open access,
   2. Original research article
   3. Journal of Hepatology,
   4. Volume 64, Issue 2,
   5. February 2016,
   6. Pages 301-307
   7. Jordan J. Feld,
   8. Christophe Moreno,
   9. Roger Trinh,
   10. Edward Tam,
   11. Fred Poordad
   12. [Download PDF (717 KB)](http://www.sciencedirect.com/science/article/pii/S0168827815006765/pdfft?md5=d3fe0c3554b9403a5690c1fdf4eebbb1&pid=1-s2.0-S0168827815006765-main.pdf)
10. [Management of Hepatitis B](http://www.sciencedirect.com/science/article/pii/S1542356510012796)
    1. Original research article
    2. Clinical Gastroenterology and Hepatology,
    3. Volume 9, Issue 5,
    4. May 2011,
    5. Pages 385-391
    6. Hin Hin Ko,
    7. David K.H. Wong,
    8. Jenny Heathcote
    9. [Purchase PDF](http://www.sciencedirect.com/science/article/pii/S1542356510012796)
11. [Biologie et cirrhose](http://www.sciencedirect.com/science/article/pii/S1773035X06805009)
    1. Original research article
    2. Revue Francophone des Laboratoires,
    3. Volume 2006, Issue 387,
    4. December 2006,
    5. Pages 65-71
    6. Hélène Voitot
    7. [Purchase PDF](http://www.sciencedirect.com/science/article/pii/S1773035X06805009)
12. [Phenotypic and functional characterization of macrophages with therapeutic potential generated from human cirrhotic monocytes in a cohort study](http://www.sciencedirect.com/science/article/pii/S1465324915010014)
    1. Open access,
    2. Original research article
    3. Cytotherapy,
    4. Volume 17, Issue 11,
    5. November 2015,
    6. Pages 1604-1616
    7. Joanna K. Moore,
    8. Alison C. Mackinnon,
    9. Dvina Wojtacha,
    10. Caroline Pope,
    11. Stuart J. Forbes
    12. [Download PDF (2,165 KB)](http://www.sciencedirect.com/science/article/pii/S1465324915010014/pdfft?md5=0de815148d38c01652fe3a1f077e8580&pid=1-s2.0-S1465324915010014-main.pdf)
13. [3D T1 relaxometry pre and post gadoxetic acid injection for the assessment of liver cirrhosis and liverfunction](http://www.sciencedirect.com/science/article/pii/S0730725X15001587)
    1. Original research article
    2. Magnetic Resonance Imaging,
    3. Volume 33, Issue 9,
    4. November 2015,
    5. Pages 1075-1082
    6. Cecilia Besa,
    7. Octavia Bane,
    8. Guido Jajamovich,
    9. Joseph Marchione,
    10. Bachir Taouli
    11. [Purchase PDF](http://www.sciencedirect.com/science/article/pii/S0730725X15001587)
14. [Prediction of oesophageal varices in hepatic cirrhosis by simple serum non-invasive markers: Results of a multicenter, large-scale study](http://www.sciencedirect.com/science/article/pii/S0168827810005283)
    1. Original research article
    2. Journal of Hepatology,
    3. Volume 53, Issue 4,
    4. October 2010,
    5. Pages 630-638
    6. Giada Sebastiani,
    7. Diego Tempesta,
    8. Giovanna Fattovich,
    9. Laurent Castera,
    10. Alfredo Alberti
    11. [Purchase PDF](http://www.sciencedirect.com/science/article/pii/S0168827810005283)
15. [Efficacy of pegylated interferon alpha-2b and ribavirin treatment on the risk of hepatocellular carcinoma in patients with chronic hepatitis C: A prospective, multicenter study](http://www.sciencedirect.com/science/article/pii/S0168827812008161)
    1. Original research article
    2. Journal of Hepatology,
    3. Volume 58, Issue 3,
    4. March 2013,
    5. Pages 495-501
    6. Eiichi Ogawa,
    7. Norihiro Furusyo,
    8. Eiji Kajiwara,
    9. Kazuhiro Takahashi,
    10. The Kyushu University Liver Disease Study (KULDS) Group
    11. [Purchase PDF](http://www.sciencedirect.com/science/article/pii/S0168827812008161)
16. [Non-invasive methods can predict oesophageal varices in patients with biliary atresia after a Kasai procedure](http://www.sciencedirect.com/science/article/pii/S1590865811001460)
    1. Original research article
    2. Digestive and Liver Disease,
    3. Volume 43, Issue 8,
    4. August 2011,
    5. Pages 659-663
    6. Antonio Colecchia,
    7. Anna Rita Di Biase,
    8. Eleonora Scaioli,
    9. Barbara Predieri,
    10. Davide Festi
    11. [Purchase PDF](http://www.sciencedirect.com/science/article/pii/S1590865811001460)
17. [Soluble urokinase plasminogen activator receptor levels are associated with severity of fibrosis in nonalcoholic fatty liver disease](http://www.sciencedirect.com/science/article/pii/S193152441400334X)
    1. Original research article
    2. Translational Research,
    3. Volume 165, Issue 6,
    4. June 2015,
    5. Pages 658-666
    6. Christopher Sjöwall,
    7. Klara Martinsson,
    8. Kristina Cardell,
    9. Mattias Ekstedt,
    10. Stergios Kechagias
    11. [Purchase PDF](http://www.sciencedirect.com/science/article/pii/S193152441400334X)
18. [Is magnetic resonance imaging of hepatic hemangioma any different in liver fibrosis and cirrhosis compared to normal liver?](http://www.sciencedirect.com/science/article/pii/S0720048X15000558)
    1. Original research article
    2. European Journal of Radiology,
    3. Volume 84, Issue 5,
    4. May 2015,
    5. Pages 816-822
    6. Rafael Duran,
    7. Maxime Ronot,
    8. Sara Di Renzo,
    9. Bettina Gregoli,
    10. Valérie Vilgrain
    11. [Purchase PDF](http://www.sciencedirect.com/science/article/pii/S0720048X15000558)
19. [The importance of fatigue cognitions in chronic hepatitis C infection](http://www.sciencedirect.com/science/article/pii/S0022399914003912)
    1. Original research article
    2. Journal of Psychosomatic Research,
    3. Volume 78, Issue 2,
    4. February 2015,
    5. Pages 193-198
    6. Dora Zalai,
    7. Morris Sherman,
    8. Kelly McShane,
    9. Colin M. Shapiro,
    10. Colleen E. Carney
    11. [Purchase PDF](http://www.sciencedirect.com/science/article/pii/S0022399914003912)
20. [The safety and efficacy of vitamin K antagonist in patients with atrial fibrillation and liver cirrhosis](http://www.sciencedirect.com/science/article/pii/S016752731402364X)
    1. Original research article
    2. International Journal of Cardiology,
    3. Volume 180,
    4. 1 February 2015,
    5. Pages 185-191
    6. Seung-Jun Lee,
    7. Jae-Sun Uhm,
    8. Jong-Youn Kim,
    9. Hui-Nam Pak,
    10. Boyoung Joung
    11. [Purchase PDF](http://www.sciencedirect.com/science/article/pii/S016752731402364X)
21. [Treatment indication and response to standard of care with peginterferon and ribavirin in acute and chronic HCV infection](http://www.sciencedirect.com/science/article/pii/S1521691812000960)
    1. Original research article
    2. Best Practice & Research Clinical Gastroenterology,
    3. Volume 26, Issue 4,
    4. August 2012,
    5. Pages 429-444
    6. Jordan J. Feld
    7. [Purchase PDF](http://www.sciencedirect.com/science/article/pii/S1521691812000960)
22. [Clinical testing of a dendritic cell targeted therapeutic vaccine in patients with chronic hepatitis C virus infection](http://www.sciencedirect.com/science/article/pii/S2329050116300183)
    1. Open access,
    2. Original research article
    3. Molecular Therapy - Methods & Clinical Development,
    4. Volume 2,
    5. 2015,
    6. Article 15006
    7. Aintzane Zabaleta,
    8. Delia D'Avola,
    9. Itziar Echeverria,
    10. Diana Llopiz,
    11. Pablo Sarobe
    12. [Download PDF (815 KB)](http://www.sciencedirect.com/science/article/pii/S2329050116300183/pdfft?md5=eb17ee6380ef3995dc7288cecdf73d63&pid=1-s2.0-S2329050116300183-main.pdf)
23. [EASL clinical practice guidelines for HFE hemochromatosis](http://www.sciencedirect.com/science/article/pii/S0168827810001972)
    1. Original research article
    2. Journal of Hepatology,
    3. Volume 53, Issue 1,
    4. July 2010,
    5. Pages 3-22
    6. European Association for the Study of the Liver
    7. [Purchase PDF](http://www.sciencedirect.com/science/article/pii/S0168827810001972)
24. [Treatment of Hepatitis C virus infection in Italy: A consensus report from an expert panel](http://www.sciencedirect.com/science/article/pii/S1590865817308071)
    1. Original research article
    2. Digestive and Liver Disease,
    3. Volume 49, Issue 7,
    4. July 2017,
    5. Pages 731-741
    6. Mauro Viganò,
    7. Carlo Federico Perno,
    8. Antonio Craxì,
    9. The AdHoc (Advancing Hepatitis C for the Optimization of Cure) Working Party
    10. [Purchase PDF](http://www.sciencedirect.com/science/article/pii/S1590865817308071)
25. [Virological characteristics of occult hepatitis B virus in a North American cohort of human immunodeficiency virus type 1-positive patients on dual active anti-HBV/HIV therapy](http://www.sciencedirect.com/science/article/pii/S1386653214001541)
    1. Original research article
    2. Journal of Clinical Virology,
    3. Volume 60, Issue 4,
    4. August 2014,
    5. Pages 347-353
    6. Carla S. Coffin,
    7. Patricia M. Mulrooney-Cousins,
    8. Carla Osiowy,
    9. Frank van der Meer,
    10. M. John Gill
    11. [Purchase PDF](http://www.sciencedirect.com/science/article/pii/S1386653214001541)
26. [Fibrosis in alcoholic and nonalcoholic steatohepatitis](http://www.sciencedirect.com/science/article/pii/S1521691811000369)
    1. Original research article
    2. Best Practice & Research Clinical Gastroenterology,
    3. Volume 25, Issue 2,
    4. April 2011,
    5. Pages 231-244
    6. Ramon Bataller,
    7. Krista Rombouts,
    8. José Altamirano,
    9. Fabio Marra
    10. [Purchase PDF](http://www.sciencedirect.com/science/article/pii/S1521691811000369)
27. [Assessment of new hyaluronic acid assays and their impact on FibroMeter scores](http://www.sciencedirect.com/science/article/pii/S0009898110006911)
    1. Original research article
    2. Clinica Chimica Acta,
    3. Volume 412, Issues 3–4,
    4. 30 January 2011,
    5. Pages 347-352
    6. Pascal Veillon,
    7. Yves Gallois,
    8. Valérie Moal,
    9. Isabelle Fouchard-Hubert,
    10. Françoise Lunel-Fabiani
    11. [Purchase PDF](http://www.sciencedirect.com/science/article/pii/S0009898110006911)
28. [Mouse Liver Dispersion for the Diagnosis of Early-Stage Fatty Liver Disease: A 70-Sample Study](http://www.sciencedirect.com/science/article/pii/S0301562913011095)
    1. Original research article
    2. Ultrasound in Medicine & Biology,
    3. Volume 40, Issue 4,
    4. April 2014,
    5. Pages 704-713
    6. Christopher T. Barry,
    7. Zaegyoo Hah,
    8. Alexander Partin,
    9. Robert A. Mooney,
    10. Kevin J. Parker
    11. [Purchase PDF](http://www.sciencedirect.com/science/article/pii/S0301562913011095)
29. [Telaprevir Is Effective Given Every 8 or 12 Hours With Ribavirin and Peginterferon Alfa-2a or -2b to Patients With Chronic Hepatitis C](http://www.sciencedirect.com/science/article/pii/S0016508510015842)
    1. Original research article
    2. Gastroenterology,
    3. Volume 140, Issue 2,
    4. February 2011,
    5. Pages 459-468.e1
    6. Patrick Marcellin,
    7. Xavier Forns,
    8. Tobias Goeser,
    9. Peter Ferenci,
    10. Maria Beumont
    11. [Purchase PDF](http://www.sciencedirect.com/science/article/pii/S0016508510015842)
30. [Utilidad del Fibroscan® para evaluar la fibrosis hepática](http://www.sciencedirect.com/science/article/pii/S0210570509003562)
    1. Original research article
    2. Gastroenterología y Hepatología,
    3. Volume 32, Issue 6,
    4. June–July 2009,
    5. Pages 415-423
    6. José A. Carrión
    7. [Purchase PDF](http://www.sciencedirect.com/science/article/pii/S0210570509003562)
31. [High Serum Levels of the Interleukin-33 Receptor Soluble ST2 as a Negative Prognostic Factor in Hepatocellular Carcinoma](http://www.sciencedirect.com/science/article/pii/S1936523313800824)
    1. Open access,
    2. Original research article
    3. Translational Oncology,
    4. Volume 6, Issue 3,
    5. June 2013,
    6. Pages 311-318
    7. Dominik Bergis,
    8. Valentin Kassis,
    9. Annika Ranglack,
    10. Verena Koeberle,
    11. Heinfried H Radeke
    12. [Download PDF (319 KB)](http://www.sciencedirect.com/science/article/pii/S1936523313800824/pdfft?md5=0e45085f98b4085417a1e8a3e75649fe&pid=1-s2.0-S1936523313800824-main.pdf)
32. [Quantification of Liver Viscoelasticity with Acoustic Radiation Force: A Study of Hepatic Fibrosis in a Rat Model](http://www.sciencedirect.com/science/article/pii/S0301562913008107)
    1. Original research article
    2. Ultrasound in Medicine & Biology,
    3. Volume 39, Issue 11,
    4. November 2013,
    5. Pages 2091-2102
    6. Xin Chen,
    7. Yuanyuan Shen,
    8. Yi Zheng,
    9. Haoming Lin,
    10. Siping Chen
    11. [Purchase PDF](http://www.sciencedirect.com/science/article/pii/S0301562913008107)
33. [A validated clinical tool for the prediction of varices in PBC: The Newcastle Varices in PBC Score](http://www.sciencedirect.com/science/article/pii/S0168827813002638)
    1. Original research article
    2. Journal of Hepatology,
    3. Volume 59, Issue 2,
    4. August 2013,
    5. Pages 327-335
    6. Imran Patanwala,
    7. Peter McMeekin,
    8. Ruth Walters,
    9. George Mells,
    10. Dave Jones
    11. [Purchase PDF](http://www.sciencedirect.com/science/article/pii/S0168827813002638)
34. [Severe hepatotoxicity following ingestion of Herbalife® nutritional supplements contaminated with Bacillus subtilis](http://www.sciencedirect.com/science/article/pii/S016882780800634X)
    1. Original research article
    2. Journal of Hepatology,
    3. Volume 50, Issue 1,
    4. January 2009,
    5. Pages 111-117
    6. Felix Stickel,
    7. Sara Droz,
    8. Eleonora Patsenker,
    9. Katja Bögli-Stuber,
    10. Stephen L. Leib
    11. [Purchase PDF](http://www.sciencedirect.com/science/article/pii/S016882780800634X)
35. [Role of liver biopsy in disorders of iron metabolism](http://www.sciencedirect.com/science/article/pii/S1756231708001680)
    1. Original research article
    2. Diagnostic Histopathology,
    3. Volume 14, Issue 12,
    4. December 2008,
    5. Pages 577-585
    6. Stefan G. Hübscher
    7. [Purchase PDF](http://www.sciencedirect.com/science/article/pii/S1756231708001680)
36. [Treatment of chronic hepatitis B: Recommendations from an Italian workshop](http://www.sciencedirect.com/science/article/pii/S1590865808001126)
    1. Original research article
    2. Digestive and Liver Disease,
    3. Volume 40, Issue 8,
    4. August 2008,
    5. Pages 603-617
    6. G. Carosi,
    7. M. Rizzetto
    8. [Purchase PDF](http://www.sciencedirect.com/science/article/pii/S1590865808001126)
37. [Role of pharmacogenetic in ribavirin outcome prediction and pharmacokinetics in an Italian cohort of HCV-1 and 4 patients](http://www.sciencedirect.com/science/article/pii/S0753332214001590)
    1. Original research article
    2. Biomedicine & Pharmacotherapy,
    3. Volume 69,
    4. February 2015,
    5. Pages 47-55
    6. Sarah Allegra,
    7. Jessica Cusato,
    8. Amedeo De Nicolò,
    9. Lucio Boglione,
    10. Antonio D’Avolio
    11. [Purchase PDF](http://www.sciencedirect.com/science/article/pii/S0753332214001590)
38. [A Clinical Prediction Rule and Platelet Count Predict Esophageal Varices in Children](http://www.sciencedirect.com/science/article/pii/S0016508511012467)
    1. Original research article
    2. Gastroenterology,
    3. Volume 141, Issue 6,
    4. December 2011,
    5. Pages 2009-2016
    6. Juan Cristóbal Gana,
    7. Dan Turner,
    8. Giorgina Mieli–Vergani,
    9. Mark Davenport,
    10. Simon C. Ling
    11. [Purchase PDF](http://www.sciencedirect.com/science/article/pii/S0016508511012467)
39. [The role of thiazolidinediones in non-alcoholic steatohepatitis – A systematic review and meta analysis](http://www.sciencedirect.com/science/article/pii/S016882781100290X)
    1. Original research article
    2. Journal of Hepatology,
    3. Volume 55, Issue 6,
    4. December 2011,
    5. Pages 1383-1390
    6. Suzanne E. Mahady,
    7. Angela C. Webster,
    8. Sarah Walker,
    9. Arun Sanyal,
    10. Jacob George
    11. [Purchase PDF](http://www.sciencedirect.com/science/article/pii/S016882781100290X)
40. [EASL Clinical Practice Guidelines: Management of chronic hepatitis B](http://www.sciencedirect.com/science/article/pii/S0168827808006375)
    1. Original research article
    2. Journal of Hepatology,
    3. Volume 50, Issue 2,
    4. February 2009,
    5. Pages 227-242
    6. European Association for the Study of the Liver
    7. [Purchase PDF](http://www.sciencedirect.com/science/article/pii/S0168827808006375)
41. [Non-invasive diagnosis for differentiating non-alcoholic steatohepatitis from simple steatosis: a meta-analysis and systematic review](http://www.sciencedirect.com/science/article/pii/S1542356517309904)
    1. Original research article
    2. Clinical Gastroenterology and Hepatology,
    3. In press, accepted manuscript,
    4. Available online 22 August 2017
    5. Pauline Verhaegh,
    6. Roisin Bavalia,
    7. Bjorn Winkens,
    8. Ad Masclee,
    9. Ger Koek
    10. [Purchase PDF](http://www.sciencedirect.com/science/article/pii/S1542356517309904)
42. [Historia natural y manifestaciones clínicas de la hepatitis B crónica](http://www.sciencedirect.com/science/article/pii/S0213005X08765157)
    1. Original research article
    2. Enfermedades Infecciosas y Microbiología Clínica,
    3. Volume 26, Supplement 7,
    4. May 2008,
    5. Pages 11-18
    6. Miguel Carneiro de Moura,
    7. Rui Marinho
    8. [Purchase PDF](http://www.sciencedirect.com/science/article/pii/S0213005X08765157)
43. [Congenital fibrocystic liver diseases](http://www.sciencedirect.com/science/article/pii/S1521691810001034)
    1. Original research article
    2. Best Practice & Research Clinical Gastroenterology,
    3. Volume 24, Issue 5,
    4. October 2010,
    5. Pages 573-584
    6. Joost P.H. Drenth,
    7. Melissa Chrispijn,
    8. Carsten Bergmann
    9. [Purchase PDF](http://www.sciencedirect.com/science/article/pii/S1521691810001034)
44. [Liver Stiffness Is Associated With Risk of Decompensation, Liver Cancer, and Death in Patients With Chronic Liver Diseases: A Systematic Review and Meta-analysis](http://www.sciencedirect.com/science/article/pii/S1542356513011671)
    1. Original research article
    2. Clinical Gastroenterology and Hepatology,
    3. Volume 11, Issue 12,
    4. December 2013,
    5. Pages 1573-1584.e2
    6. Siddharth Singh,
    7. Larissa L. Fujii,
    8. Mohammad Hassan Murad,
    9. Zhen Wang,
    10. Jayant A. Talwalkar
    11. [Purchase PDF](http://www.sciencedirect.com/science/article/pii/S1542356513011671)
45. [Performance of ELF Serum Markers in Predicting Fibrosis Stage in Pediatric Non-Alcoholic Fatty LiverDisease](http://www.sciencedirect.com/science/article/pii/S0016508508016788)
    1. Original research article
    2. Gastroenterology,
    3. Volume 136, Issue 1,
    4. January 2009,
    5. Pages 160-167
    6. Valerio Nobili,
    7. Julie Parkes,
    8. Gianfranco Bottazzo,
    9. Matilde Marcellini,
    10. William M. Rosenberg
    11. [Purchase PDF](http://www.sciencedirect.com/science/article/pii/S0016508508016788)
46. [Can preoperative diffusion-weighted MRI predict postoperative hepatic insufficiency after curative resection of HBV-related hepatocellular carcinoma? A pilot study](http://www.sciencedirect.com/science/article/pii/S0730725X10000810)
    1. Original research article
    2. Magnetic Resonance Imaging,
    3. Volume 28, Issue 6,
    4. July 2010,
    5. Pages 802-811
    6. Seung Up Kim,
    7. Young Chul Kim,
    8. Ji Soo Choi,
    9. Kyung Sik Kim,
    10. Myeong-Jin Kim
    11. [Purchase PDF](http://www.sciencedirect.com/science/article/pii/S0730725X10000810)
47. [Safety of treatments for inflammatory bowel disease: Clinical practice guidelines of the Italian Group for the Study of Inflammatory Bowel Disease (IG-IBD)](http://www.sciencedirect.com/science/article/pii/S1590865817301457)
    1. Original research article
    2. Digestive and Liver Disease,
    3. Volume 49, Issue 4,
    4. April 2017,
    5. Pages 338-358
    6. Livia Biancone,
    7. Vito Annese,
    8. Sandro Ardizzone,
    9. Alessandro Armuzzi,
    10. List of attendees:
    11. [Purchase PDF](http://www.sciencedirect.com/science/article/pii/S1590865817301457)
48. [ESPEN guidelines on chronic intestinal failure in adults](http://www.sciencedirect.com/science/article/pii/S0261561416000479)
    1. Original research article
    2. Clinical Nutrition,
    3. Volume 35, Issue 2,
    4. April 2016,
    5. Pages 247-307
    6. Loris Pironi,
    7. Jann Arends,
    8. Federico Bozzetti,
    9. Cristina Cuerda,
    10. the Home Artificial Nutrition & Chronic Intestinal Failure Special Interest Group of ESPEN
    11. [Purchase PDF](http://www.sciencedirect.com/science/article/pii/S0261561416000479)
49. [Numerical modeling of multiphase plasma/soil flow and heat transfer in an electric arc furnace](http://www.sciencedirect.com/science/article/pii/001793109400248T)
    1. Original research article
    2. International Journal of Heat and Mass Transfer,
    3. Volume 38, Issue 7,
    4. May 1995,
    5. Pages 1161-1171
    6. Seungho Paik,
    7. Hoa D. Nguyen
    8. [Purchase PDF](http://www.sciencedirect.com/science/article/pii/001793109400248T)
50. [XIII National Congress of Digestive Diseases, Italian Federation of Digestive Diseases – FIMAD Palermo, 29 September – 3 October 2007](http://www.sciencedirect.com/science/article/pii/S159086580780911X)
    1. Original research article
    2. Digestive and Liver Disease,
    3. Volume 39, Supplement 2,
    4. September 2007,
    5. Pages s139-s343
    6. No authors available
    7. [Purchase PDF](http://www.sciencedirect.com/science/article/pii/S159086580780911X)
51. [25](http://www.sciencedirect.com/search?qs=%22Liver%20transplantation%22%20and%20%22transient%20elastography%22&show=25&sortBy=relevance&articleTypes=OR&navigation=true)
52. [50](http://www.sciencedirect.com/search?qs=%22Liver%20transplantation%22%20and%20%22transient%20elastography%22&show=50&sortBy=relevance&articleTypes=OR&navigation=true)
53. 100
54. [previous](http://www.sciencedirect.com/search?qs=%22Liver%20transplantation%22%20and%20%22transient%20elastography%22&show=100&sortBy=relevance&articleTypes=OR&offset=100&navigation=true)
55. Page 3 of 3

[Feedback](http://www.sciencedirect.com/search?qs=%22Liver%20transplantation%22%20and%20%22transient%20elastography%22&show=100&sortBy=relevance&articleTypes=OR&offset=200&navigation=true)

[About ScienceDirect](http://www.elsevier.com/solutions/sciencedirect)[Remote access](http://www.sciencedirect.com/science/activateaccess)[Shopping cart](http://www.sciencedirect.com/science?_ob=ShoppingCartURL&_method=display&_zone=TopNavBar&_origin=srp&md5=e5857d1631c4b1f612897ed029064507)[Contact and support](https://service.elsevier.com/app/contact/supporthub/sciencedirect/)[Terms and conditions](https://www.elsevier.com/legal/elsevier-website-terms-and-conditions)[Privacy policy](http://www.elsevier.com/legal/privacy-policy)

Cookies are used by this site. For more information, visit the [cookies page](https://www.elsevier.com/solutions/sciencedirect/support/cookies).

Copyright © 2017 Elsevier B.V. or its licensors or contributors. ScienceDirect ® is a registered trademark of Elsevier B.V.

“Liver transplantation” and “serum fibrosis markers”

[Skip to Main content](http://www.sciencedirect.com/search?qs=%22Liver%20transplantation%22%20and%20%22serum%20fibrosis%20markers%22&show=100&sortBy=relevance&articleTypes=OR&lastSelectedFacet=articleTypes#main_content)

- [Journals](http://www.sciencedirect.com/science/journals)
- [Books](http://www.sciencedirect.com/science/bookbshsrw)
- [Register](https://www.sciencedirect.com/user/register?returnURL=http%3A%2F%2Fwww.sciencedirect.com%2Fsearch)
- [Sign in](https://www.sciencedirect.com/user/login?returnURL=http%3A%2F%2Fwww.sciencedirect.com%2Fsearch)

Top of Form

[Advanced search](http://www.sciencedirect.com/science/search)

Bottom of Form

40 results

Refine by:

Top of Form

Years

1. 2017 (2)
2. 2016 (4)
3. 2015 (1)
4. 2014 (5)
5. 2012 (7)
6. 2011 (4)
7. 2010 (4)
8. 2009 (3)
9. 2008 (4)
10. 2007 (2)

Show less

Article type

1. Review articles (2,871)
2. Original research (6,957)
3. Encyclopedia (250)
4. Book chapters (2,031)
5. Other (8,661)

Show less

Publication title

1. Journal of Hepatology (7)
2. Clinical Gastroenterology and Hepatology (6)
3. Gastroenterology (3)
4. Transplantation Proceedings (2)
5. The Journal of Thoracic and Cardiovascular Surgery (2)
6. Digestive and Liver Disease (2)
7. Gastroenterología y Hepatología (2)
8. Journal of Clinical and Experimental Hepatology (2)
9. Controlled Clinical Trials (1)
10. European Journal of Radiology (1)

Show less

[Clear all filters](http://www.sciencedirect.com/search?qs=%22Liver%20transplantation%22%20and%20%22serum%20fibrosis%20markers%22&show=100&sortBy=relevance)

Bottom of Form

sorted by *relevance* | [date](http://www.sciencedirect.com/search?qs=%22Liver%20transplantation%22%20and%20%22serum%20fibrosis%20markers%22&show=100&sortBy=date&articleTypes=OR)

1. [Serum Fibrosis Markers Identify Patients With Mild and Progressive Hepatitis C Recurrence After LiverTransplantation](http://www.sciencedirect.com/science/article/pii/S0016508509016977)
   1. Original research article
   2. Gastroenterology,
   3. Volume 138, Issue 1,
   4. January 2010,
   5. Pages 147-158.e1
   6. José A. Carrión,
   7. Guillermo Fernández–Varo,
   8. Miquel Bruguera,
   9. Juan–Carlos García–Pagán,
   10. Miquel Navasa
   11. [Purchase PDF](http://www.sciencedirect.com/science/article/pii/S0016508509016977)
2. [Liver transplantation in the setting of chronic HCV](http://www.sciencedirect.com/science/article/pii/S1521691812000935)
   1. Original research article
   2. Best Practice & Research Clinical Gastroenterology,
   3. Volume 26, Issue 4,
   4. August 2012,
   5. Pages 531-548
   6. Norah Terrault
   7. [Purchase PDF](http://www.sciencedirect.com/science/article/pii/S1521691812000935)
3. Want a richer search experience?

Sign in for additional filter options, multiple article downloads, and more.

Top of Form

Sign in

Bottom of Form

1. [FibroScan Evaluation of Liver Fibrosis in Liver Transplantation](http://www.sciencedirect.com/science/article/pii/S004113450900164X)
   1. Original research article
   2. Transplantation Proceedings,
   3. Volume 41, Issue 3,
   4. April 2009,
   5. Pages 1044-1046
   6. G. Sánchez Antolin,
   7. F. Garcia Pajares,
   8. M.A. Vallecillo,
   9. P. Fernandez Orcajo,
   10. A. Caro-Patón
   11. [Purchase PDF](http://www.sciencedirect.com/science/article/pii/S004113450900164X)
2. [Protective Effects of Norursodeoxycholic Acid Versus Ursodeoxycholic Acid on Thioacetamide-induced RatLiver Fibrosis](http://www.sciencedirect.com/science/article/pii/S0973688314000073)
   1. Original research article
   2. Journal of Clinical and Experimental Hepatology,
   3. Volume 4, Issue 4,
   4. December 2014,
   5. Pages 293-301
   6. Vyacheslav U. Buko,
   7. Oxana Y. Lukivskaya,
   8. Elena E. Naruta,
   9. Elena B. Belonovskaya,
   10. Horst-Dietmar Tauschel
   11. [Purchase PDF](http://www.sciencedirect.com/science/article/pii/S0973688314000073)
[truncated: 87,930 more chars]
